# Cu(I)-Catalyzed Stereoselective Glycosylation of “Electron-Deficient” Glycals

**DOI:** 10.1021/acs.joc.5c00172

**Published:** 2025-05-20

**Authors:** Mukul Mahanti, Carla M. Saunders, Nicholas Walker, Natalie Fey, M. Carmen Galan

**Affiliations:** School of Chemistry, 1980 University of Bristol, Cantock’s Close, Bristol BS8 1TS, U.K.

## Abstract

An efficient and mild Cu­(I)-catalyzed Michael-type conjugate addition for 2-nitro glycals to access *O-*, *S-*, and *C*-glycosides with high stereoselectivity is reported. Under optimized conditions, nitrogalactals achieved high α-selectivity, whereas nitroglucal predominantly gave β-selective glycosides. The method is further demonstrated with other Michael-type substrates, including 2-formyl glycals and 3-keto glycals. Initial mechanistic investigations using NMR and supported by DFT calculations suggest that the reaction proceeds via a preorganized complex that positions the nucleophile close to the double bond to promote the Michael-type addition, in a manner analogous to enzyme-catalyzed processes. Moreover, the versatility of this synthetic approach was exemplified in the stereoselective synthesis of a mucin-type glycopeptide and the chemoselective one-pot synthesis of a trisaccharide.

## Graphical abstract

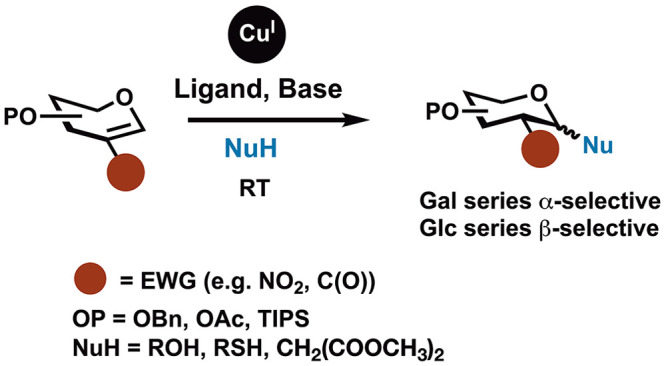

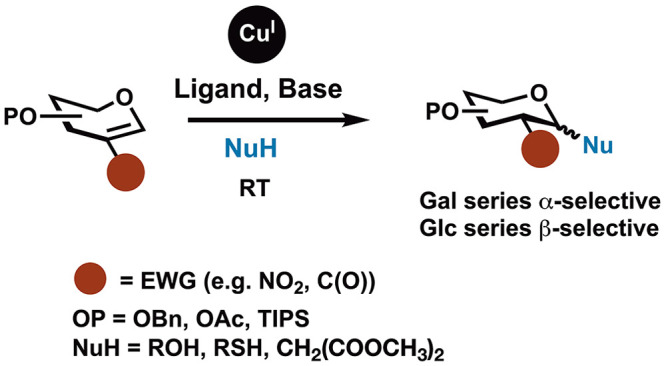

Carbohydrates and their glycoconjugates play a multitude of roles in biology and, in order to determine the nature of their interactions, structurally defined carbohydrate-based synthetic tools are needed.^1,2^ However, despite advancements in the field, achieving the stereoselective synthesis of glycosides remains a formidable challenge.^3,4^

2-Amino-2-deoxyglycosides represent an important class of glycans commonly found as components of natural products such as peptidoglycan, glycoproteins, polysaccharides, e.g., chitin, heparan sulfate, nucleosides, and aminoglycoside antibiotics, e.g., streptomycin, kanamycin B, neomycins, tunicamycin V, lividomycin, among others.^2,5^ Moreover, these molecules assume vital functions in many biological processes including cancer metastasis, inflammation, immune defense, fertilization, signal transduction, cell growth, cell–cell adhesion, and cell recognition,^2,6^ which makes them an attractive synthetic target.

While the synthesis of 1,2-*trans* aminoglycosides has been extensively researched, most strategies reported to date require the use of amides or carbamates as *N*-protecting groups that exhibit 1,2-*trans*-directing behavior during the glycosylation reaction.^7^ However, there are still challenges associated with the use of the naturally occurring 2-acetamido glycosyl donors due to the formation of stable oxazoline intermediates which often result in low yields.^8^ Conversely, access to 1,2-*cis* aminoglycosides still remains particularly difficult with regards to stereocontrol, even in the presence of nonparticipating groups, e.g., 2-azido glycosyl donors.^9^

The utility of 2-nitroglycals as glycosyl donors, where the nitro group acts as a latent amino functionality that does not actively participate in the reaction, has been demonstrated in the synthesis of aminoglycosides.^10^ The Schmidt group reported pioneering work on the strong base-catalyzed concatenation reaction of 2-nitroglycals to access α- and β-linked 2-amino-2-deoxy-*O*-glycosides;^11^ however, the method is limited to base-stable protecting groups.^6,12,13^ Other milder organocatalytic strategies have subsequently been reported for nitroglycal activation in glycosylation: Sun and Yu reported a DMAP or PPY-catalyzed Michael-type addition of nucleophiles to 2-nitroglycals to access β-glycosides.^13^ Our group disclosed the use of cinchona thiourea-catalyzed activation of 2-nitrogalactals,^14^ while almost in parallel, Yoshida, Takao, and co-workers developed the amino-thiourea-catalyzed glycosylation of 2-nitrogalactal with phenols to give α-galactopyranosides as the major products.^15^ More recent examples of stereoselective 2-nitroglycal activation include Michael-type reactions facilitated by *N*-heterocyclic carbenes^16^ or superbase-catalysis using P_4_^t^Bu as the catalyst (Scheme 1).^17^

**Scheme 1:**
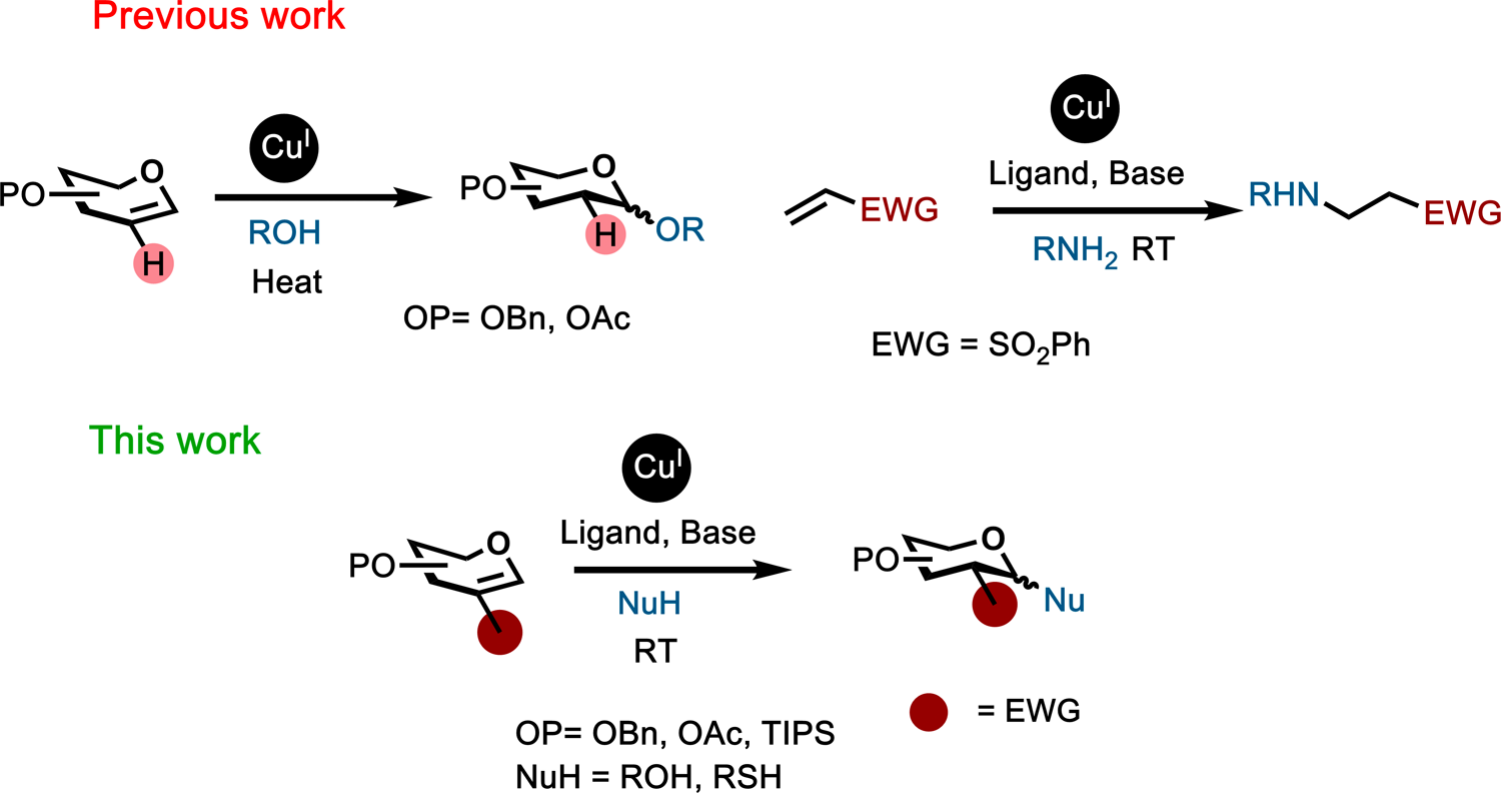
Cu­(I)-Catalyzed Activation of Glycals (Top Left) and Michael-Type Addition on Olefins (Top Right) and Activation of Electron-Deficient Glycals (Bottom)

Despite the elegance of such approaches, these organocatalytic methods either incur high costs, lack stability at room temperature, or require complex synthetic procedures to access them. Hence, there is a demand for sustainable, practical, and robust approaches that offer both high yield and stereoselectivity. First-row transition metals have garnered substantial attention as replacements to more precious transition metals in catalytic processes, including examples in carbohydrate chemistry.^3,18–20^ Among these, copper (Cu) stands out as an affordable, abundantly available, and environmentally friendly alternative. Additionally, copper complexes exhibit versatile and unique chemistry, displaying excellent functional group tolerance. Moreover, the reactivity of Cu can be tuned depending on its oxidation state and coordination sphere, and as a result, this metal can efficiently catalyze reactions involving one or two-electron mechanisms.^18,21^

Remarkably, despite its accessibility and vast potential for catalysis, copper remains relatively underexplored in glycosylation chemistry.^22,23^ Previously, we disclosed the Cu­(I)-catalyzed glycal-type glycosylation, which included examples of “disarmed” peracetylated galactals which could not be activated by other reported mild catalytic systems.^23^ Although the reported catalytic system could not activate nitroglycals, recent examples of a copper-catalyzed Michael-type addition to α,β-unsaturated olefins^24^ motivated us to investigate the scope of Cu­(I) catalysis for the glycosylation of Michael-type glycoside donors, such as nitroglycals.

Initial experiments started by screening a series of Cu­(I) catalysts in the presence and absence of different inorganic bases in CH_2_Cl_2_ for the glycosylation of perbenzylated 2-deoxy nitrogalactal **1a** with BnOH **2a** (Table 1).

**Table 1:**
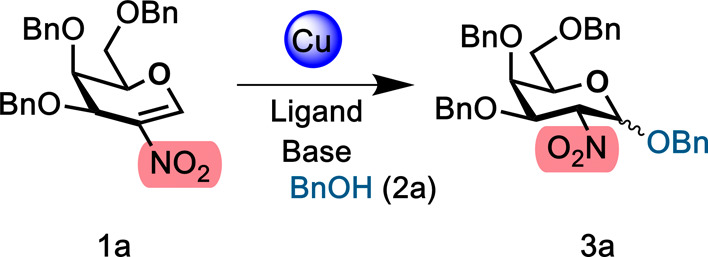
Catalyst Optimization in the Model Reaction of Glycal **1a** with BnOH **2a**

entry	catalyst (0.1)	ligand (0.1)	additive (0.2)	*t*	yield (%)^a^	α/β^a^
1	CuCl		Cs_2_CO_3_	4 h	70	1:0
2	CuBr		Cs_2_CO_3_	4 h	72	1:0
3	CuBr·SMe_2_		Cs_2_CO_3_	4 h	81	1:0
4	CuI		Cs_2_CO_3_	4 h	65	1:0
5	(CuOTf)_2_·C_6_H_6_		Cs_2_CO_3_	4 h	<15	1:0
6	(CuOTf)_2_·C_6_H_6_			8 h	NR	ND
7	CuBr·SMe_2_			8 h	<20	1:0
8			Cs_2_CO_3_	6 h	55	16:1
9			Cs_2_CO_3_ ^b^	3 h	70	16:1
10	CuBr·SMe_2_	XPhos	Cs_2_CO_3_	4 h	91	1:0
11	CuBr·SMe_2_	BINAP	Cs_2_CO_3_	4 h	81	1:0
12	CuBr·SMe_2_	Tol BINAP	Cs_2_CO_3_	4 h	80	1:0
13	CuBr·SMe_2_	XPhos	K_2_CO_3_	6 h	69	1:0
14	CuBr·SMe_2_	XPhos	Na_2_CO_3_	6 h	45	1:0
15	CuBr·SMe_2_	XPhos	KOH	6 h	35	10:1
16	CuBr·SMe_2_	XPhos	Li_2_CO_3_	6 h	<5	ND
17	CuBr·SMe_2_	XPhos	Cs_2_CO_3_	4 h	72^c^	1:0
18	CuBr·SMe_2_	XPhos	Cs_2_CO_3_	4 h	57^d^	>30:1
19	CuBr·SMe_2_	XPhos	Cs_2_CO_3_	4 h	62^e^	1:0

a Determined from crude NMR, and reactions are carried out with catalyst (0.1), ligand (0.1), and base (0.2) in DCM at rt, unless otherwise stated.

b 1 equiv of Cs_2_CO_3_ used.

c Reaction in toluene.

d Reaction in MeCN.

e Reaction in THF. Note: all reactions were carried out until all starting material was consumed and/or no further product formation was generated.

Excitingly, we found that the combination of CuBr·SMe_2_ (10 mol %) and Cs_2_CO_3_ (20 mol %) gave 81% conversion to disaccharide **3a** with complete α-selectivity in CH_2_Cl_2_ (entry 3). In the presence of only Cs_2_CO_3_ (20 mol % or 1 equiv), the reaction gave lower yields and the stereoselectivity was also compromised (51–70% and 16:1 α:β) (entries 8 and 9). The catalytic activity and stereoselectivity of transition metals can be tuned by the judicious choice of ligands.^19^ To that end, common phosphine ligands (BINAP, Tol BINAP and XPhos)^25^ were also screened in the reaction and we found that addition of 10 mol % XPhos in combination with CuBr·SMe_2_ (10 mol %) and Cs_2_CO_3_ (20 mol %) gave the best result, with 91% yield and α-stereocontrol within 4 h (entry 10, Table 1). Using other inorganic bases such as K_2_CO_3_, Na_2_CO_3_, KOH, and Li_2_CO_3_ produced lower yields, likely due to Cs_2_CO_3_ exhibiting greater basic strength and higher solubility in CH_2_Cl_2_. Finally, lower yields were obtained when using other solvents such as toluene, acetonitrile, or THF (entries 17–19).

Having optimized the reaction conditions, the substrate scope was investigated by reacting nitrogalactal **1a** with a series of primary and secondary OH nucleophiles **2b**–**2l** (Table 2). In the case of primary alcohols (**2b**–**2h**, entries 1–7) the reaction went smoothly in good to excellent yields (65–91%) and with high to complete α-selectivity. In the case of secondary alcohols (**2i** and **2j**, entries 8 and 9), an increased catalyst-ligand loading of 20 mol % and more base (50 mol %) are needed to achieve yields of 64 and 65%, respectively. Finally, the reaction also proved to be suitable under the standard optimized conditions for thiol nucleophiles as in the case of thiophenol **2k**, which led to thioglycosides **3k** (72%; 8:1 α:β) and *C*-nucleophiles, e.g., malonate **2l**, to generate **3l** in 68% yield and complete α-selectivity (entry 11).

**Table 2:**
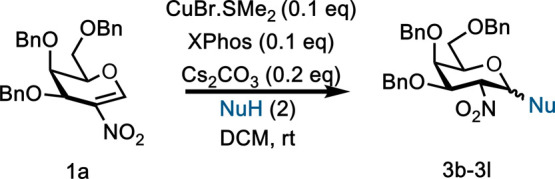

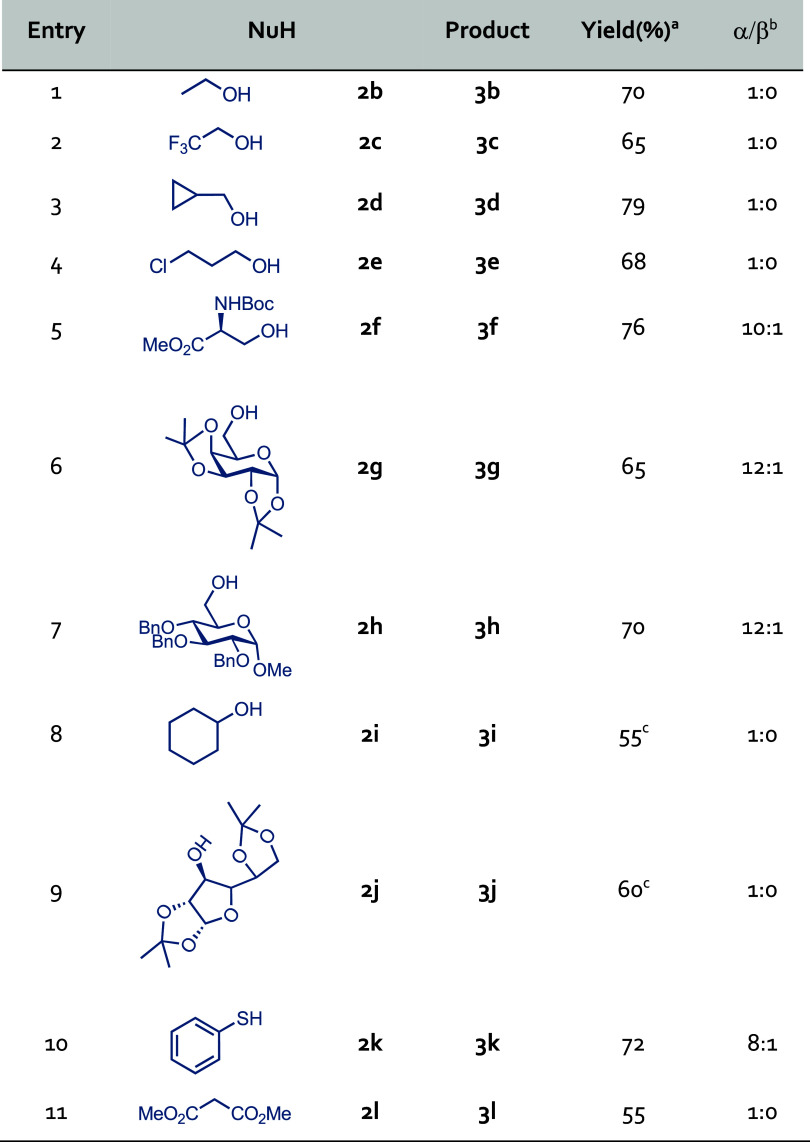
Reaction of Glycal **1a** with Glycoside Acceptors **2b**–**2m**

a Isolated yield.

b Determined from the NMR.

c 20 mol % CuBr·SMe_2_ and XPhos and 50 mol % Cs_2_CO_3_.

Next, we focused on evaluating the scope of the reaction with other glycal donors (Table 3). Reactions worked well for other orthogonally protected nitrogalactals **1b** and **1c** and **2a** as the OH nucleophile, with isolated product yields of 65–75% and α-stereocontrol (entries 1 and 2). On the other hand, peracetylated **1d** gave instead the 2,3-unsaturated Ferrier product **3o** in 60% yield and α-stereocontrol (entry 3).

**Table 3:**
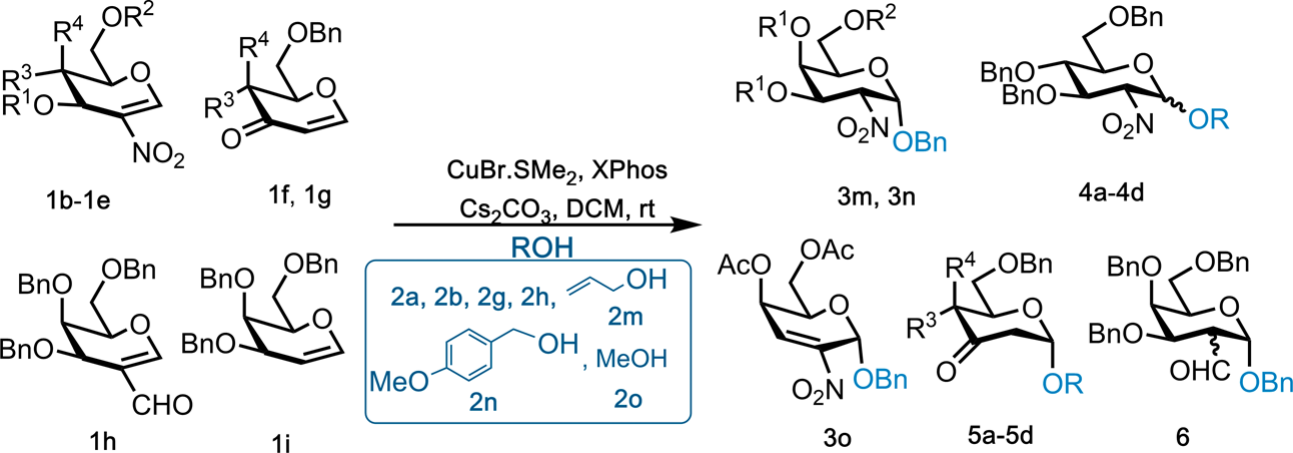
Reaction Scope between Glycals **1b**–**1h** with **ROH** Acceptors

entry	R1	R2	R3	R4	NuH	product	yield % (α:β)^a^
1 **1b**	Bn	TBS	H	OBn	2a	3m	75 (1:0)
2 **1c**	Bn	Ac	H	OBn	2a	3n	65 (1:0)
3 **1d**	Ac	Ac	H	OAc	2a	3o	60 (1:0)
4 **1e**	Bn	Bn	OBn	H	2h	4a	60^b^(1:4)
5 **1e**	Bn	Bn	OBn	H	2g	4b	51 (1:8)
6 **1e**	Bn	Bn	OBn	H	2m	4c	63 (0:1)
7 **1e**	Bn	Bn	OBn	H	2n	4d	66 (1:16)
8 **1f**		Bn	H	OBn	2o	5a	90 (1:0)
9 **1f**		Bn	H	OBn	2a	5b	78 (1:0)
10 **1g**		Bn	OBn	H	2o	5c	85 (>30:1)
11 **1g**		Bn	OBn	H	2a	5d	73^b^ (>30:1)
12 **1h**					2a	6	42^b^(2:1)^c^
13 **1i**					2a	NR	NR

a Determined from ^1^H NMR.

b 20 mol % CuBr·SMe_2_ and XPhos and 50 mol % Cs_2_CO_3_.

c Two *cis* and *trans* α-isomers. NR: no reaction.

The reaction scope with 2-nitroglucal donor **1e** with acceptors **2h**, **2g**, **2m**, and **2n** was also explored, and although we needed to increase the catalyst-ligand loading to 20 mol % and base to 50 mol %, conversions of 51–66% were achieved. Interestingly, in this case, we observed a reversal of stereoselectivity, with products being predominantly β-selective. In the case of allyl alcohol **2m** (entry 6) and for *p*-methoxy benzyl acceptor **2n** (entry 7), complete or high β-selectivity (1:16 α:β) was observed, respectively.

Encouraged by the results, we investigated other “electron-deficient” glycals such as glycal-derived enones (**1f**, **1g**) and 2-formyl glycal (**1h**) Table 3. We anticipated that the electron-withdrawing nature of the carbonyl group in conjugation with the glycal double bond should facilitate a Michael-type addition reaction at the anomeric position, as in the case of the nitroglycals. Reactions with perbenzylated galactose enone **1f** subjected to our standard conditions proceeded smoothly and produced selectively the α-isomer in 78–90% yield (entries 8 and 9). The glucoside’s enone counterpart **1g** could also be activated and led to the desired products, giving selectively the α-isomer in 90% yield with small nucleophiles. In the case of the bulkier benzyl alcohol, the reaction was slower and required 20 mol % catalyst-ligand and 50 mol % of Cs_2_CO_3_ to produce **5c** in 73% yield with complete α-stereoselectivity (entries 10 and 11). Similarly, activation of 2-formyl galactal donor **1h** was also possible and α-selective products **6** were isolated in 42% yield and with a 2:1 (*cis*:*trans*) isomer ratio, respectively, likely due to base-catalyzed enolization under the basic conditions. Finally, activation of perbenzylated galactal was not possible, suggesting chemoselectivity toward electron-deficient enol ethers.

In order to showcase the versatility of the methodology, the synthesis of mucin type core 5 structure **8** was attempted. First, nitrogalactal **1a** was reacted with *N*-Boc-methylester serine **2f** under the optimized Cu­(I) conditions to give glycopeptide **3p** in 65% yield and α-stereocontrol (13:1 α:β). Removal of the silyl ether protecting group with TBAF gave monohydroxylated acceptor **7**,^26^ followed by Cu­(I) glycosylation with **1a**, afforded the target aminoglycoside **8** in 71% yield. Additionally, the distinct chemoselectivity of Cu­(I) and Cu­(II) precatalysts toward glycal^23^ vs nitroglycals could be exploited for the one-pot synthesis of trisaccharide **9**. Activation of nitrogalactal **1a** with CuBr·SMe_2_ in the presence of galactal-acceptor **2p** generated **3q** *in situ*, as monitored by TLC, and subsequent glycosylation with galactose acceptor **2g** in the presence of 20 mol % of (CuOTf)_2_.C_6_H_6_ at 40°C, afforded **9** with an overall 42% yield from **1a** at 15:1 αα:βα.

To gain a better understanding of the reaction mechanism, ^1^H NMR spectroscopy studies were carried out at room temperature in CD_2_Cl_2_ using equimolar combinations of Cu­(I) catalyst, base (Cs_2_CO_3_), donor **1a** or acceptor **2i**. Mixtures of **1a** with either Cu­(I) or both Cu­(I) and base led to a slight broadening of both H-1 and H-3 proton signals of nitroglycal **1a** (Figure S2 in SI), which was not observed when **1a** and only Cs_2_CO_3_ were mixed together, suggesting the copper catalyst interacts with the enol ether. Interestingly, the broad OH singlet in **2i** shifted and split into a doublet of doublets (dd) upon exposure to either the catalyst or the base. In the presence of Cu­(I) and Cs_2_CO_3_, a slightly altered splitting pattern was observed (Figure S1 in the SI). This implies that although the OH nucleophile can engage separately with the catalyst and the base, when all constituents converge, an acid–base catalysis mechanism likely operates, expediting the Michael-type addition reaction (Scheme 2).

**Scheme 2:**
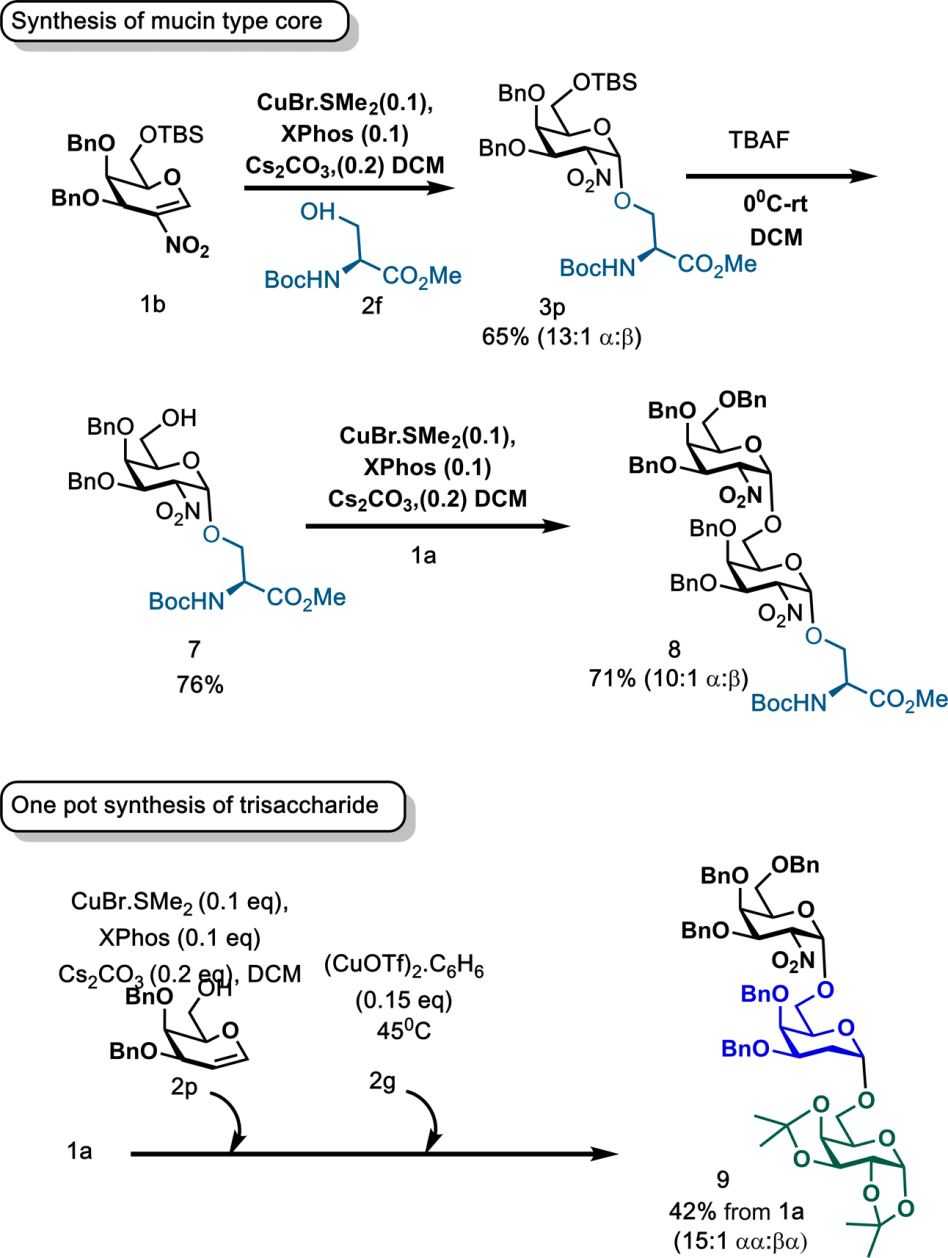
(A) Synthesis of Mucin-Type Core 5 **7** and (B) One-Pot Synthesis of Trisaccharide **8**

To further elucidate the mechanism, we first performed a primary KIE study with 0.1 mmol of donor **1a**, 0.2 mmol of MeOH, and 0.2 mmol of MeOD in DCM-d2 using our standard conditions. This produced nondeuterated product **3r** and deuterated product **3s** in a 94:6 ratio. On the other hand, the secondary KIE study with 0.1 mmol of **1a**, 0.2 mmol of CD_3_OH, and MeOH led to products **3r** and **3t** in a 147:153 ratio, respectively, so nearly a 1:1 ratio. These two studies suggest that breaking of the O–H bond is involved in the reaction mechanism (Scheme 3).

**Scheme 3:**
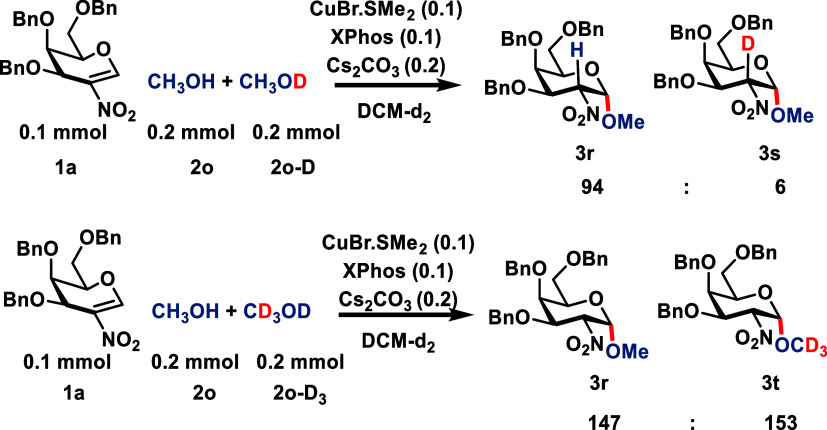
Primary and Secondary KIE Study of the Donor **1a** with Methanol and Deuterated Methanol (CH_3_OD and CD_3_OH)

Based on our initial investigations, we hypothesized that as part of the initial steps, the glycal acceptor is able to interact with the catalyst, which the presence of base facilitates. Furthermore, as donor **1** is added to this mixture, Cu likely coordinates to the nitro group, which brings the catalyst into the vicinity of the glycal double bond. The orientation of this complex facilitates the Michael addition of the nucleophile to the nitrogalactal from the sterically favored side, the anomeric effect, and transition state **C** to generate the α-anomer **D** with C2 carbanion (Scheme 4A). Then proton transfer takes place from the β-face to produce product **3**. Interestingly, the reaction of nitroglucal produced predominantly β-selectivity using our optimized conditions, presumably generating the β-anomer **D′**, followed by proton transfer from the α face. We investigated this mechanistic postulate for nucleophilic addition of [OBn]^−^ to **1a** and **1e** with DFT calculations, focusing primarily on the intermediates **C/C′** and **D/D′** in Scheme 4B (B3LYP-D3/6-31G­(d) for all atoms, except for SDD on Cu, CPCM solvation with DCM, see SI for details). Two different half chair structures (^4^H_5_ and ^5^H_4_) for glucose were investigated to help determine the observed selectivity for Michael addition.

**Scheme 4:**
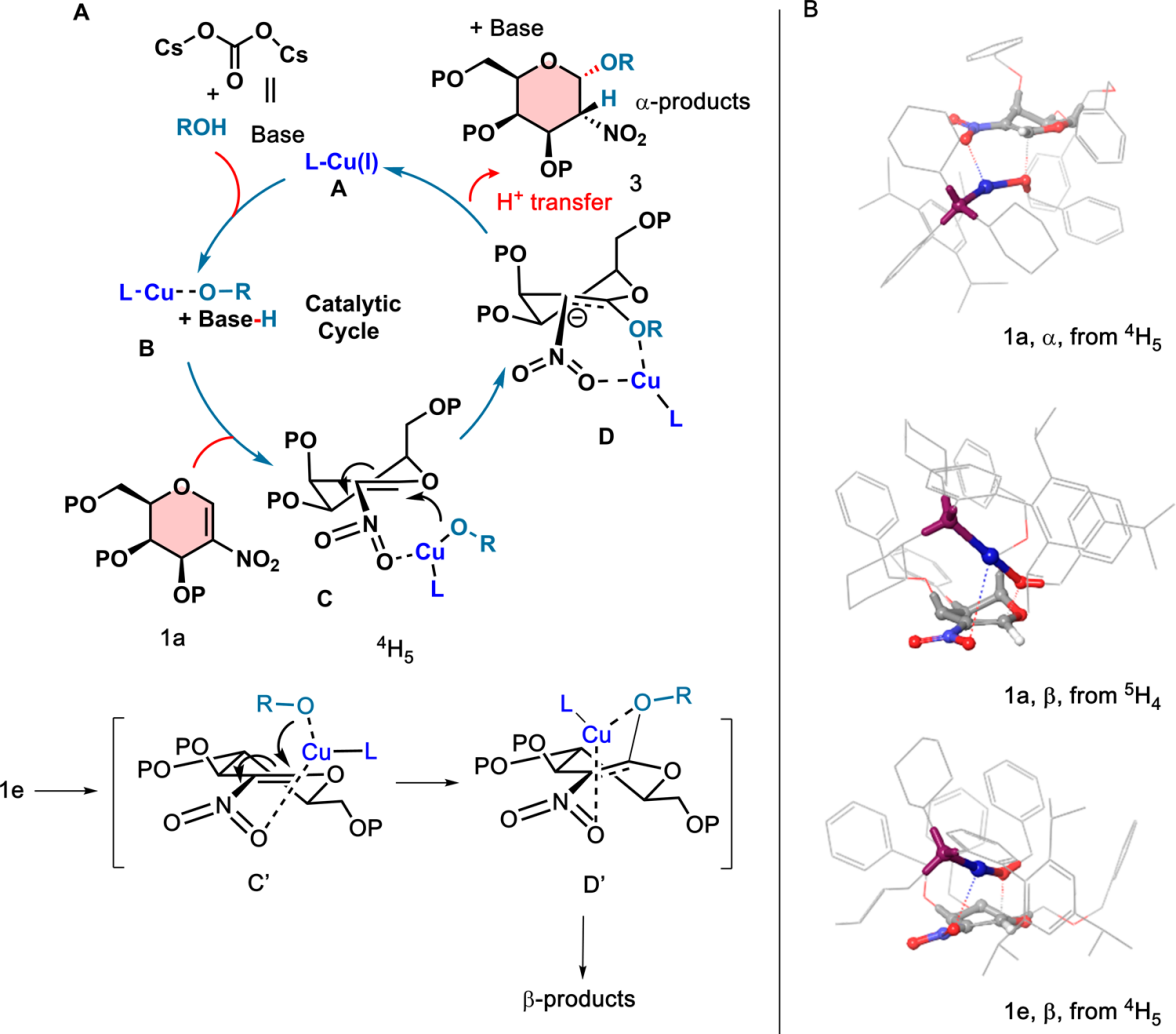
(A) Proposed Mechanism and (B) Lowest Energy DFT-Calculated Transition States for **1a** α- and β-Selective Routes, and **1e** β-Selective Pathway

For nitrogalactal **1a**, the energy difference between the two half chairs is small, favoring ^5^H_4_ by 1.7 kcal mol^–1^. We were able to locate transition states for nucleophilic attack for α- and β-selective routes starting from each half chair conformer. In all cases, the barrier to reaction from intermediate **C** is small (3–6 kcal mol^–1^). We found the β-selective routes slightly more favorable throughout, but as discussed in the Supporting Information, the dispersive contributions to the calculated energies may be excessive. Intermediate complexes **D** tend to be lower in energy than complexes of type **C**, with further low energy conformers located that did not connect directly to the best TS shown (see SI). Intermediates **D** generally show an interaction between copper and one of the nitro oxygens, with lower energy conformers generally moving away from interactions with OBn installed in the transition state. Scheme 4B shows the transition states for the lowest energy pathways found (distorted ^4^H_5_/α, ^5^H_4_/β), with Table 4 summarizing key structural features; results for the other routes can be found in the Supporting Information.

**Table 4:** DFT Relative Energies and Key Structural Metrics for Most Favorable α- and β-Selective Routes (See SI for Alternatives)^a^

**1a** ^b^	Δ*G*, α	key distances	Δ*G*, β	key distances
C	0.0	r1 1.841	–2.7^b^	r1 1.851
		r2 3.108		r2 3.385
		r3 2.710		r3 2.749
TS	5.9	r1 1.924	3.0	r1 1.885
		r2 2.285		r2 3.231
		r3 2.055		r3 1.885
D	–0.9^d^	r1 2.271	–7.6^d^	r1 3.076
		r2 1.958		r2 1.927
		r3 1.455		r3 1.426

**1e** ^c^	Δ*G*, α	key distances	Δ*G*, β	key distances
C′	–0.9^c^	r1 1.840	0.0	r1 1.839
		r2 4.181		r2 3.437
		r3 2.646		r3 2.120
TS	7.3	r1 1.893	6.6	r1 1.908
		r2 3.637		r2 2.971
		r3 1.824		r3 1.850
D′	0.7^d^	r1 2.317	1.1^d^	r1 2.221
		r2 2.004		r2 2.052
		r3 1.460		r3 1.454

a All energies given in kcal mol^–1^, all distances in Å, Cu-OBn = r1, Cu-ONO = r2, C-OBn = r3.

b Relative to lowest α (experimentally observed for **1a**).

c Relative to lowest β (experimentally observed for **1e**).

d Lower energy conformers have been located for D, which do not connect directly to the best TS shown here (see SI for details).

For nitroglucal **1e**, the energetic preference for the ^5^H_4_ conformer is more pronounced (7.4 kcal mol^–1^), and this conformer seems to be maximizing intramolecular dispersive interactions. For the β-selective nucleophilic attack, this preference disappears, giving quite similar energies for **C′** and the transition states for both half chairs, with a slight preference for the ^4^H_5_ conformer. However, the ^4^H_5_ conformer remains more unfavorable for the α-selective routes. From these different starting points, barriers to nucleophilic attack are again small (3–8 kcal mol^–1^). While the intermediate complexes **D**' connecting to the TS geometries found are slightly uphill compared to those of **C′**, conformers lying downhill are easy to locate and have been included in the SI (Table S2). Overall, the β-selective routes are favored kinetically, in line with experimental observations. The lowest energy pathway (^4^H_5_/β) has also been included in Scheme 4B and Table 4.

Attractive interactions between the benzyl-substituents are more obvious in the ^5^H_4_ conformers, suggesting a preorganization of the complex driven by the interactions with the Cu­(I) catalyst and further enhanced by the XPhos ligand.

These calculations show that a copper­(I)-mediated pathway is energetically accessible for all combinations of half chair conformations and direction of nucleophilic addition. For the transition states, the copper center interacts with the incoming [OBn]^−^ nucleophile and, in more favorable transition states, also with the nitro-substituent of the glycals; an interaction with the nitro group is maintained for complexes **D**/**D′**. Some of these complexes also have an interaction with the OBn group, but distances are varied and unlikely to contribute as much. All transition states show a short Cu-OBn distance (r1), while there is greater variability for Cu-ONO distances (r2), supporting the mechanistic hypothesis that copper coordination supports and facilitates the nucleophilic attack.

In summary, we have developed an unprecedented copper­(I)-catalyzed Michael-type addition reaction to produce *O-*, -*S*, and *C*-glycosides with high stereoselectivity. The optimized reaction conditions are robust, mild, and cost-effective. This method has further established that copper is a powerful metal and can contribute to stereoselectivity in glycosylation reactions. The reaction provides a mechanistically intriguing example of Cu-catalyzed nitro-alkene functionalization. Our experimental data and theoretical analysis suggest that the Cu catalyst, aided by the base, likely coordinates both the nitro group and the nucleophile, creating a preorganized complex that positions the nucleophile close to the double bond for stereoselective addition. This arrangement promotes Michael-type addition, echoing the precision often seen in enzyme-catalyzed processes. We also demonstrate that this method can be used for other Michael-type donors such as enone glycal and 2-formyl glycals and exemplify the versatility of this strategy in the efficient synthesis of biologically interesting natural and non-natural glycosides in a limited number of steps and with high stereocontrol. The mild and practical conditions, high yields, and stereoselectivity make this diastereoselective reaction a valuable tool for synthetic chemistry, with potential applications both within and beyond carbohydrate research.

## Supplementary Material





## Data Availability

The data underlying this study are available in the published article and its Supporting Information.

## Notes

Synthetic protocols and characterization data for all compounds including NMR spectra and the description of computational methods and results (PDF)
Compiled structures (XYZ)
