# Supplementary material for: Cu(I)-Catalyzed Stereoselective Glycosylation of “Electron-Deficient” Glycals
Source: J Org Chem. 2025 May 20;90(21):6937–45. doi: 10.1021/acs.joc.5c00172 (PMC12131213; doi:10.1021/acs.joc.5c00172)

Supporting Information

Cu(I)-catalyzed stereoselective glycosylation of 'electron-deficient' glycals

Mukul Mahanti, Carla M. Saunders, Nicholas Walker, Natalie Fey* and M. Carmen Galan*

School of Chemistry, University of Bristol, Cantock's Close, Bristol, BS8 1TS, United Kingdom

Table of Contents

General Experimental Part **2**

List of Donor **3**

List of Acceptors **3**

General procedure for Cu-catalysed glycosylation of nitro glycals **4**

Synthesis of 2-deoxy-2-nitro-glycosides **5**

Synthesis of mucin core 7 .... **22**

One pot synthesis of the trisaccharide: **24**

Mechanistic Study **25**

- **NMR Experiment** **25**
  - **With the acceptor** 26
  - **With Donor** 27
- **KIE study** **27**
  - **Primary Kinetic Isotope Effect** 27
  - **Secondary Kinetic Isotope Effect** 28

Computational Studies **30**

REFERENCES **38**

NMR spectra **41**

General Experimental Part

All reactions, unless otherwise stated, were carried out at Rt under an inert (N_2) atmosphere in standard glassware. Compositions of solvents are given as ratios of volumes unless otherwise stated. Anhydrous solvents were either purchased from Sigma Aldrich or obtained from the University of Bristol's anhydrous Grubbs' type solvent stills. Reactions were monitored by TLC analysis, on aluminium-backed TLC plates, Silica Gel 60 F254 (Merck). TLCs were visualized with either UV light (λ = 254 nm) or charring with 5% H_2SO_4 in EtOH solution. Solutions were concentrated under reduced pressure using both a Büchi rotary evaporator at a pressure of either 15 mmHg (diaphragm pump) or 0.1 mmHg (oil pump), as appropriate, and a high vacuum line at room temperature. Reactions that required heating were carried out on a Dry syn heating block.

^{1}H NMR and ^{13}C NMR spectra were measured in the solvent stated at 400, 500 or 600 MHz. Chemical shifts are quoted in parts per million from residual solvent peak ($CDCl_3$: ^{1}H - 7.26 ppm and ^{13}C - 77.16 ppm) and coupling constants (*J*) given in Hertz. ^{1}H shifts are given to 2.d.p and ^{13}C shifts are given to 1 d.p. Multiplicities are abbreviated as bs (broad), s (singlet), d (doublet), t (triplet), q (quartet), m (multiplet) or combinations thereof. Coupling constants (*J* values) are quoted to the nearest 0.1 Hz. Where a signal in the ^{1}H or ^{13}C NMR cannot be fully assigned as much information as possible is given for assignment. If signals overlap, both signal assignments are included in the same bracket (eg. C-3 and C-4) or (2C, CH_2Ph). Where ^{1}H NMR data for a mixture of anomers has been measured, separate 1H assignments are given. Where signals in the ^{13}C NMR spectrum are not assignable due to spectral overlap, the multiplicity of the carbon is given (e.g. CH_2). Where required, structural assignments were made with additional information from 2D COSY and HSQC experiments. Mass spectrometry was carried out by the University of Bristol Mass Spectrometry service on a micrOTOF II (ESI) spectrometer, with the HRMS mode incorporating a lock-in mass injected midway through the run (sodium formate). IR spectra were recorded on a Perkin Elmer Spectrum One FT-IR spectrometer fitted with a universal ATR accessory.

List of Donors

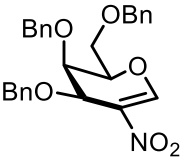

1a

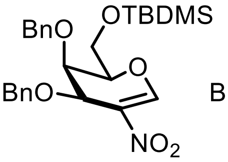

1b

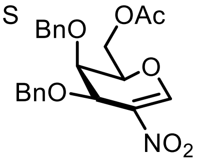

1c

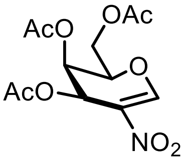

1d

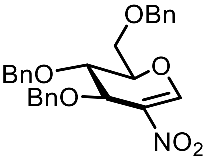

1e

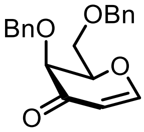

1f

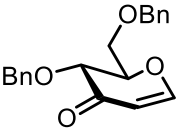

1g

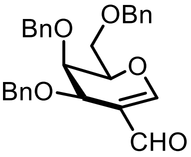

1h

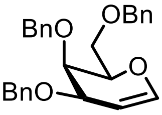

1i

The synthesis for glycosyl donors **1a**, **1c**, **1d**, **1e**, **1f**, **1g**, **1h** is reported in the literature.[1-4] **1i** is commercially available.

List of Acceptors

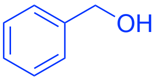

2a

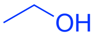

2b

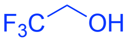

2c

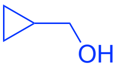

2d

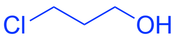

2e

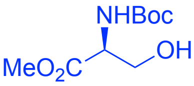

2f

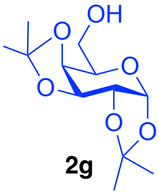

2g

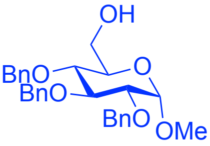

2h

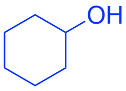

2i

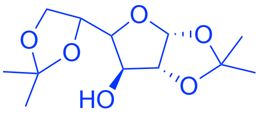

2j

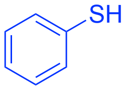

2k

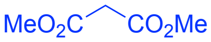

2l

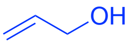

2m

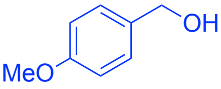

2n

MeOH

2o

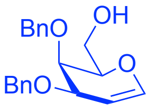

2p

General procedure for synthesising the nitro glycals A

In an oven dried flask, glycal (45 mM, 1eq) and TBAN (45 mM, 1eq) were taken in dry DCM. The solution was cooled to 0^0 C. To that was added TFA (45 mM, 1eq) dropwise for 5 min and it was warmed to room temperature. The reaction was continued at the room temperature until TLC showed complete consumption of the starting material. After that it was again cooled to 0^0 C and quenched with Et_3N (45mM, 1eq). It was then further stirred for 15 min. Then it was diluted with DCM and washed with water (2 times) and brine (2times). The organic layer was separated and concentrated before the it was purified by flash chromatography.

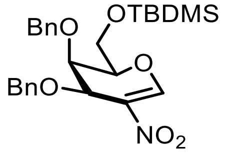

1b

Compound **1b** was prepared by the general procedure A from the corresponding glycal (1g, 2.27 mmol), TBAN (930 mg, 2.27 mmol) and TFA (460 μL, 2.27 mmol) in 50 ml dry DCM. Purification by column chromatography (8:1 Hexane: EtOAc) afforded 716 mg of compound **1b** as colourless oil in 65% yield.

^{1}H NMR (400 MHz, $CDCl_3$) δ 8.07 (s, 1H, H-1), 7.36-7.25 (m, 10H, Ph), 4.91 – 4.83 (m, 2H, H-3, -OCH*H*Ph), 4.79 (d, *J* = 10.9 Hz, 1H, OCH*H*Ph), 4.71 (d, *J* = 12.0 Hz, 1H, OCH*H*Ph), 4.62 (d, *J* = 12.0 Hz, 1H, OCH*H*Ph), 4.54 – 4.46 (m, 1H, H-6a), 4.09 – 4.01 (m, 2H, H-4, H-6b), 3.88 (dd, *J* = 5.3, 3.7 Hz, 1H, H-5), 0.85 (s, 9H), 0.02 (d, *J* = 2.4 Hz, 6H). **^{13}C{^{1}H} NMR (126 MHz, $CDCl_3$)** δ 154.7, 138.2, 137.1, 131.7, 128.9, 128.8, 128.6, 128.6, 128.4, 128.3, 127.9, 127.8, 80.6, 74.9, 73.3, 72.3, 67.6, 61.2, 26.0, 25.9, 18.5, -5.1, -5.2.

HRMS cald. for $C_{26}H_{35}NO_6Si$ (M+H)+ 486.2312 found 486.2319.

General procedure for Cu-catalysed glycosylation of nitro glycals B

In an oven dried flask, to the solution of the acceptor (43 mM, 2eq) in dry DCM was added $CuBr.SMe_2$ (2.2 to 4.4 mM, 10-20 mol%) or any Cu(II) catalysts screened, XPhos (2.2 to 4.4 mM, 10-20mol%) and Cs_2CO_3 (4.4 to 11 mM, 20 to 50 mol%) and the mixture was stirred at room temperature for 10 mins. To that was added the solution of the donor (22 mM, 1eq) in dry DCM. The overall concentration of the reaction mixture was 0.1 mM with respect to the donor. After that the reaction was left on stirring at room temperature and monitored by TLC.

Synthesis of 2-deoxy-nitroglycosides

Benzyl 3,4,6-tri-O-benzyl-2-deoxy-2-nitro-α-D-galactopyranoside (3a)

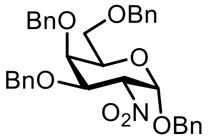

Following the general glycosylation procedure, to the solution of **2a** (9 μL, 0.086 mmol) in 2ml of dry DCM was added $CuBr.SMe_2$ (1 mg, 0.0043 mmol), XPhos (2 mg, 0.0043 mmol) and Cs_2CO_3 (3 mg, 0.0086 mmol) and this suspension was stirred for 15 min in room temperature. To this mixture was added the solution of **1a** (20 mg, 0.043 mmol) in 1 ml dry DCM. The reaction mixture was stirred for 5h in room temperature following purification by column chromatography (10:1 to 3:1 Heptane: EtOAc) to afford 22 mg of compound **3a** as colourless oil in 90% yield (α only).

^{1}H NMR (400 MHz, $CDCl_3$) δ 7.50 – 7.16 (m, 20H, Ar*H*), 5.39 (d, J = 4.2 Hz, 1H), 5.02 (dd, J = 10.6, 4.2 Hz, 1H, H-2), 4.85 (d, J = 11.2 Hz, 1H, OCH*H*Ph), 4.80 – 4.64 (m, 3H, OCH*H*Ph), 4.59 – 4.39 (m, 5H, OCH*H*Ph, H-3), 4.08 – 3.96 (m, 2H, H-4, H-5), 3.63 – 3.46 (m, 2H, H-6).**^{13}C{^{1}H} NMR (101 MHz, $CDCl_3$) δ** 138.1 (Ar*C*), 137.9 (Ar*C*), 137.5 (Ar*C*), 136.4 (Ar*C*), 128.6 (Ar*C*), 128.6 (Ar*C*), 128.5 (Ar*C*), 128.3 (Ar*C*), 128.3 (Ar*C*), 128.2 (Ar*C*), 128.2 (Ar*C*), 128.0 (Ar*C*), 128.0 (Ar*C*), 127.9 (Ar*C*), 95.6 (C-1), 84.4 (C-2), 75.4 (C-3), 75.2 (C-5), 73.7, 73.4, 73.2, 70.0 (C-4), 70.0, 68.4 (C-6).

Spectroscopic data was in agreement with previously reported literature.[5]

Ethyl 3,4,6-tri-*O*-benzyl-2-deoxy-2-nitro-α-D-galactopyranoside (3b)

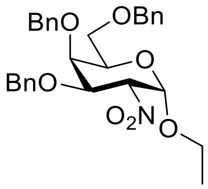

Following the general glycosylation procedure, to the solution of **2b** (5 μL, 0.086 mmol) in 2ml of dry DCM was added $CuBr.SMe_2$ (1 mg, 0.0043 mmol), XPhos (2 mg, 0.0043 mmol) and Cs_2CO_3 (3 mg, 0.0086 mmol) and this suspension was stirred for 15 min in room temperature. To this mixture was added the solution of **1a** (20 mg, 0.043 mmol) in 1 ml dry DCM. The reaction mixture was stirred for 2h in room temperature following purification by column chromatography (10:1 to 3:1 Heptane: EtOAc) to afford 15 mg of compound **3b** as colourless oil in 70% yield (α only).

^{1}H NMR (400 MHz, CDCl$_3$) δ 7.49 – 7.19 (m, 15H), 5.35 (d, J = 4.2 Hz, 1H, H-1), 5.03 (dd, J = 10.7, 4.2 Hz, 1H, H-2), 4.89 (d, J = 11.2 Hz, 1H, OCH*H*Ph), 4.85 – 4.72 (m, 2H, OC*H*HPh), 4.59 – 4.40 (m, 4H, OC*H*HPh, H-3), 4.13 – 4.00 (m, 2H, H-4, H-5), 3.76 (dd, J = 10.0, 7.1 Hz, 1H, -C*H*H-CH$_3$), 3.69 – 3.45 (m, 3H, -CH*H*-CH$_3$, H-6), 1.21 (t, J = 7.1 Hz, 3H, -CH$_2$-*CH$_3$*).**^{13}C{^{1}H} NMR (101 MHz, CDCl$_3$) δ** 138.2 (Ar*C*), 137.9 (Ar*C*), 137.5 (Ar*C*), 128.6 (Ar*C*), 128.6 (Ar*C*), 128.5 (Ar*C*), 128.3 (Ar*C*), 128.3 (Ar*C*),, 128.2 ((Ar*C*), 128.0 (Ar*C*), 128.0 (Ar*C*), 96.2 (C-1), 84.6 (C-2), 75.4 (C-3), 75.2, 73.7 (C-5), 73.42, 73.21, 69.7 (C-4), 68.5 , 64.4 (C-6), 14.76.

Spectroscopic data was in agreement with previously reported literature.[6]

2,2,2-Trifluoroethyl 3,4,6-tri-O-benzyl-2-deoxy-2-nitro-α-D-galactopyranoside (3c)

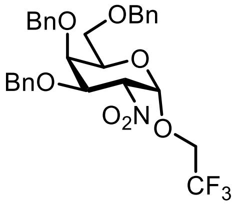

Following the general glycosylation procedure, to the solution of **2c** (9 μL, 0.086 mmol) in 2ml of dry DCM was added CuBr.SMe$_2$ (1 mg, 0.0043 mmol), XPhos (2 mg, 0.0043 mmol) and Cs$_2$CO$_3$ (3 mg, 0.0086 mmol) and this suspension was stirred for 15 min in room temperature. To this mixture was added the solution of **1a** (20 mg, 0.043 mmol) in 1 ml dry DCM. The reaction mixture was stirred for 5h in room temperature following purification by column chromatography (10:1 to 3:1 Heptane: EtOAc) to afford 15 mg of compound **3c** as colourless oil at 65% yield (α only).

^{1}H NMR (600 MHz, CDCl$_3$) δ 7.40 – 7.20 (m, 15H), 5.40 (d, J = 4.2 Hz, 1H, H-1), 5.02 (dd, J = 10.7, 4.2 Hz, 1H, H-2), 4.84 (d, J = 11.1 Hz, 1H), 4.80 – 4.70 (m, 2H), 4.51 – 4.40 (m, 4H, OCH*H*Ph, H-3), 4.03 (dd, J = 3.1, 1.1 Hz, 1H, -H4), 4.00 (ddd, J = 7.4, 6.1, 1.2 Hz, 1H, H-5), 3.95 (dd, J = 12.3, 8.5 Hz, 1H, -OCH*H*CF$_3$), 3.88 (dd, J = 12.3, 8.4 Hz, 1H, -OCH*H*CF$_3$), 3.58 – 3.52 (m, 2H, H-6). **^{13}C{^{1}H} NMR (151 MHz, CDCl$_3$) δ** 137.9 (Ar*C*), 137.7 (Ar*C*), 137.3 (Ar*C*), 128.7 (Ar*C*), 128.6 (Ar*C*), 128. 5 (Ar*C*), 128.5 (Ar*C*), 128.4 (Ar*C*), 128.3 (Ar*C*), 128.3 (Ar*C*), 128.1 (Ar*C*), 127.9 (Ar*C*), 124.2 (Ar*C*), 122.4 (Ar*C*), 120.8 (Ar*C*), 96.9 (C-1), 83.7 (C-2), 75.3, 74.9, 73.7, 73.35, 73.1, 70.6, 68.2, 65.5, 65.3, 65.1, 64.8, 34.4, 31.0, 29.8, 26.6, 26.3, 26.2, 25.9, 25.7, 24.2, 22.8.

Spectroscopic data was in agreement with previously reported literature.[1]

2-Cyclopropyl-ethyl 3,4,6-tri-*O*-benzyl-2-deoxy-2-nitro-α-D-galactopyranoside (3d)

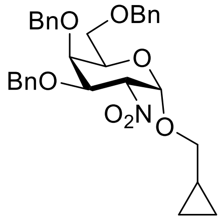

Following the general glycosylation procedure, to the solution of **2d** (6 μL, 0.086 mmol) in 2ml of dry DCM was added CuBr.SMe_2 (1 mg, 0.0043 mmol), XPhos (2 mg, 0.0043 mmol) and Cs_2CO_3 (3 mg, 0.0086 mmol) and this suspension was stirred for 15 min in room temperature. To this mixture was added the solution of **1a** (20 mg, 0.043 mmol) in 1 ml dry DCM. The reaction mixture was stirred for 3h in room temperature following purification by column chromatography (10:1 to 3:1 Heptane: EtOAc) to afford 18 mg of compound **3d** as colourless oil in 79% yield (α only).

^{1}H NMR (400 MHz, $CDCl_3$) δ 7.36 – 7.13 (m, 15H, Ar*H*), 5.35 (d, *J* = 4.2 Hz, 1H, -H1), 4.94 (dd, *J* = 10.6, 4.2 Hz, 1H, -H2), 4.79 (d, *J* = 11.2 Hz, 1H, -OCH*H*Ph), 4.75 – 4.65 (m, 1H, -OCH*H*Ph), 4.47 – 4.32 (m, 2H, -OCH*H*Ph, -H3), 4.04 – 3.93 (m, 2H, H-4, H-5), 3.57 – 3.37 (m, 3H, -OCH_2-cylcopropane. -H6a, H-6b), 3.25 (dd, *J* = 10.9, 7.1 Hz, 1H, OCH_2-C*H*-cylcopropane), 1.31 – 1.12 (m, 3H), 0.94 (ddt, *J* = 9.2, 7.6, 2.8 Hz, 1H), 0.45 (ddt, *J* = 9.6, 8.0, 4.0 Hz, 2H), 0.21 – 0.00 (m, 2H).**^{13}C{^{1}H} NMR (101 MHz, $CDCl_3$) δ** 138.2, 137.9, 137.6, 128.6, 128.6, 128.5, 128.3, 128.2, 128.0, 127.98, 127.9 (Ar*C*), 95.9 (C-1), 84.6 (C-2), 77.4, 75.4 (C-3), 75.2, 73.7 , 73.4 (C, 73.3, 73.2, 69.8 (C-5), 68.6 (C-6), 10.0, 3.4, 2.8.

HRMS cald. for $C_{31}H_{35}NO_7$ (M+Na)+ 556.2311 found 556.2320.

3-Chloropropyl- 3,4,6-tri-*O*-benzyl-2-deoxy-2-nitro-α-D-galactopyranoside (3e)

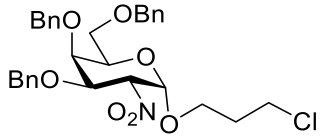

Following the general glycosylation procedure, to the solution of **2e** (8 μL, 0.086 mmol) in 2ml of dry DCM was added CuBr.SMe_2 (1 mg, 0.0043 mmol), XPhos (2 mg, 0.0043 mmol) and Cs_2CO_3 (3 mg, 0.0086 mmol) and this suspension was stirred for 15 min in room temperature. To this mixture was added the solution of **1a** (20 mg, 0.043 mmol) in 1 ml dry DCM. The reaction mixture was stirred for 3h in room temperature following

purification by column chromatography (10:1 to 3:1 Heptane: EtOAc) to afford 16 mg of compound **3e** as colourless oil in 68% yield (α only).

^{1}H NMR (600 MHz, CDCl$_3$) δ 7.38 – 7.19 (m, 15H), 5.30 (d, *J* = 4.3 Hz, 1H), 5.00 (dd, *J* = 10.6, 4.2 Hz, 1H), 4.84 (d, *J* = 11.2 Hz, 1H), 4.74 (d, *J* = 2.2 Hz, 1H), 4.52 – 4.46 (m, 1H), 4.46 – 4.39 (m, 2H), 4.07 – 3.97 (m, 2H), 3.86 (ddd, *J* = 10.0, 6.6, 5.2 Hz, 1H), 3.62 – 3.48 (m, 5H), 1.99 (qd, *J* = 5.8, 1.6 Hz, 2H), 1.56 (s, 2H). **^{13}C{^{1}H} NMR (151 MHz, CDCl$_3$) δ** 138.1, 137.8, 137.4, 128.7, 128.6, 128.5, 128.3, 128.2, 128.0, 128.0, 128.0, 96.5, 84.4, 75.3, 75.2, 73.7, 73.2, 69.9, 68.4, 65.0, 41.5, 32.0.

HRMS cald. for $C_{30}H_{34}NClO_7$ (M+Na)+ 578.1921 found 578.1926.

_N_-Boc-L-threonine methyl ester 3,4,6-_O_-tri-benzyl-2-deoxy-2-nitro-D galactopyranoside (3f)

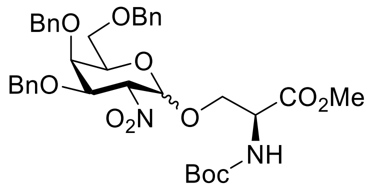

Following the general glycosylation procedure, to the solution of **2f** (18 mg, 0.086 mmol) in 2ml of dry DCM was added $CuBr.SMe_2$ (1 mg, 0.0043 mmol), XPhos (2 mg, 0.0043 mmol) and Cs_2CO_3 (3 mg, 0.0086 mmol) and this suspension was stirred for 15 min in room temperature. To this mixture was added the solution of **1a** (20 mg, 0.043 mmol) in 1 ml dry DCM. The reaction mixture was stirred for 12h in room temperature following purification by column chromatography (10:1 to 3:1 Heptane: EtOAc) to afford 22 mg of compound **3f** as colourless oil in 76% yield (α: β 10:1).

3fα

^{1}H NMR (400 MHz, CDCl$_3$) δ 7.52 – 7.12 (m, 15H), 5.30 – 5.25 (d, 1H, J 4.1 Hz, -H1), 5.21 (d, J = 8.8 Hz, 1H, -N*H*Boc), 4.99 (dd, J = 10.6, 4.2 Hz, 1H, -H2), 4.83 (d, J = 11.2 Hz, 1H, -OCH*H*Ph), 4.73 (d, J = 1.8 Hz, 2H), 4.52 – 4.40 (m, 4H, -OCH*H*HPh, -C*H*-Ser), 4.36 (dd, J = 10.7, 3.0 Hz, 1H), 4.17 – 4.06 (m, 1H, -H6a), 3.99 (dd, J = 3.0, 1.1 Hz, 1H, -H4), 3.91 – 3.82 (m, 1H, -H5), 3.72 (s, 3H, -CO$_2$C*H*$_3$), 3.64 (dd, J = 9.8, 3.7 Hz, 1H), 3.54 (dq, J = 5.4, 2.5 Hz, 2H, H-6b, -OC*H*$_2$CH-Ser), 1.46 (s, 9H).**^{13}C{^{1}H} NMR (101 MHz, CDCl$_3$) δ** 170.2, 137.8, 137.6, 136.6, 128.7, 128.7, 128.5, 128.4, 128.4, 128.2, 128.1, 128.0, 100.8, 87.3, 80.2, 79.4, 75.0, 74.2, 73.8, 72.5, 71.5, 71.3, 67.9, 53.8, 52.7, 28.5.

3fβ

^{1}H NMR (400 MHz, CDCl$_3$) δ 7.31 – 7.17 (m, 15H), 7.16 – 7.10 (m, 2H), 5.24 – 5.17 (m, 1H), 5.13 (d, *J* = 8.8 Hz, 1H), 4.91 (dd, *J* = 10.6, 4.2 Hz, 1H), 4.75 (d, *J* = 11.2 Hz, 1H), 4.65 (d, *J* = 1.8 Hz, 2H), 4.44 – 4.31 (m, 4H), 4.28 (dd, *J* = 10.7, 3.0 Hz, 1H), 4.10 – 3.98 (m, 1H), 3.91 (dd, *J* = 3.0, 1.1 Hz, 1H), 3.82 – 3.73 (m, 1H), 3.63 (s, 3H), 3.56 (dd, *J* = 9.8, 3.7 Hz, 1H), 3.45 (dq, *J* = 5.4, 2.5 Hz, 2H), 1.38 (s, 9H). **^{13}C{^{1}H} NMR (101 MHz, CDCl$_3$) δ** 170.6, 155.4, 138.0, 137.8, 137.4, 128.7, 128.7, 128.6, 128.5, 128.41, 128.3, 128.3, 128.2, 128.1, 128.0, 128.0, 96.4, 84.2, 80.4, 75.2, 75.1, 73.7, 73.2, 73.1, 70.2, 68.6, 68.2, 53.7, 52.7, 28.4.

Spectroscopic data was in agreement with previously reported literature.[7]

1,2;3,4-Di-O-isopropylidene-6-*O*-(3,4,6-tri-*O*-benzyl-2-deoxy-2-nitro-D-galactopyranosyl)-α-D-galactopyranose (3g)

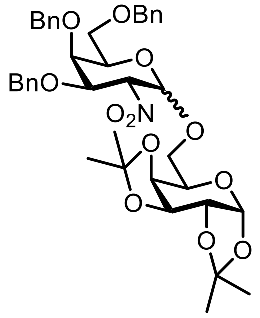

Following the general glycosylation procedure, to the solution of **2g** (22 mg, 0.086 mmol) in 2ml of dry DCM was added CuBr.SMe$_2$ (1 mg, 0.0043 mmol), XPhos (2 mg, 0.0043 mmol) and Cs$_2$CO$_3$ (3 mg, 0.0086 mmol) and this suspension was stirred for 15 min in room temperature. To this mixture was added the solution of **1a** (20 mg, 0.043 mmol) in 1 ml dry DCM. The reaction mixture was stirred for 12h in room temperature following purification by column chromatography (10:1 to 3:1 Heptane: EtOAc) to afford 20 mg of compound **3g** as colourless oil in 65% yield (α:β 12:1).

3gα

^{1}H NMR (400 MHz, CDCl$_3$) δ 7.28-7.12 (m, 15H, Ar*H*), 5.41 (d, *J* = 4.9 Hz, 1H, -H1), 5.28 (d, *J* = 4.1 Hz, 1H, H-1'), 4.92 (dd, *J* = 10.6, 4.2 Hz, 1H, H-2'), 4.76 (d, *J* = 11.2 Hz, 1H, -OCH*H*Ph), 4.65 (s, 2H, -OCH*H*Ph), 4.50 (dd, *J* = 8.0, 2.4 Hz, 1H), 4.45 – 4.38 (m, 3H, -OCHH*H*Ph, H-3'), 4.35 (d, *J* = 11.8 Hz, 1H, -OCH*H*Ph), 4.21 (dd, *J* = 5.0, 2.3 Hz, 1H, -H2), 4.02 (dt, *J* = 8.0, 1.9 Hz, 2H, -H4, -H5), 3.97 (dd, *J* = 3.1, 1.2 Hz, 1H, -H4'), 3.88 – 3.80 (m, 1H, -H5'), 3.73 (dd, *J* = 10.3, 6.6 Hz, 1H, -H6a'), 3.60 – 3.42 (m, 3H, -H6b', H6), 1.48 (s, 3H, -C(C*H*$_3$)$_2$), 1.33 (s, 3H, -C(C*H*$_3$)$_2$), 1.26 (2S, 6H, -C(C*H*$_3$)$_2$). **^{13}C{^{1}H} NMR (101 MHz, CDCl$_3$) δ** 138.2, 137.9, 137.5, 128.7, 128.6, 128.6, 128.5, 128.4, 128.3, 128.3, 128.2, 128.2, 128.1, 128.0, 127.9 (Ar*C*), 109.4 (-*C*(CH$_3$)$_2$), 108.8 (-*C*(CH$_3$)$_2$), 96.8 (C-1), 96.3,C-1'), 84.5

(C-2'), 77.4, 75.3 (C-4'), 75.2 (C-5'), 73.6 (C-2), 73.3, 73.1, 70.8, 70.7, 70.5 (C-4), 69.6 (C-5), 68.2, 67.3 (C-6'), 66.4 (C-6), 26.3, 26.0, 25., 24.3.

Spectroscopic data was in agreement with previously reported literature.[1]

3gβ

^{1}H NMR (400 MHz, CDCl$_3$) δ 7.39 – 7.26 (m, 15H, Ar*H*), 7.24 (dd, *J* = 7.8, 1.8 Hz, 2H, Ar*H*), 5.47 (d, *J* = 5.0 Hz, 1H, -H1), 4.94 (d, *J* = 8.0 Hz, 1H, -H2), 4.90 – 4.81 (m, 2H, -OCH*H*Ph), 4.60 (d, *J* = 11.5 Hz, 1H, -H), 4.58 – 4.51 (m, 2H), 4.51 – 4.41 (m, 3H), 4.27 (dd, *J* = 4.9, 2.4 Hz, 1H), 4.18 – 4.06 (m, 2H), 3.99 (d, *J* = 2.8 Hz, 1H), 3.94 (dd, *J* = 11.3, 4.6 Hz, 1H), 3.88 (td, *J* = 5.5, 1.8 Hz, 1H), 3.75 (dd, *J* = 11.3, 6.5 Hz, 1H), 3.68 – 3.57 (m, 3H), 1.51 (s, 3H), 1.40 (s, 3H), 1.32 (s, 3H), 1.30 (s, 3H). **^{13}C{^{1}H} NMR (101 MHz, CDCl$_3$) δ** 138.0, 137.8, 136.9, 128.7, 128.6, 128.4, 128.4, 128.3, 128.2, 128.1, 128.0, 109.4, 100.0, 100.3, 96.3, 87.6, 79.9, 77.4, 74.9, 73.9, 73.8, 72.7, 71.8, 71.2, 70.7, 70.6, 68.4, 68.0, 67.8, 26.2, 26.1, 25.1, 24.4.

Spectroscopic data was in agreement with previously reported literature.[8]

Methyl 2,3,4-tri-*O*-benzyl-6-*O*-(3,4,6-tri-O-benzyl-2-deoxy-2-nitro-D-galactopyranosyl)-D-glucopyranoside (3h)

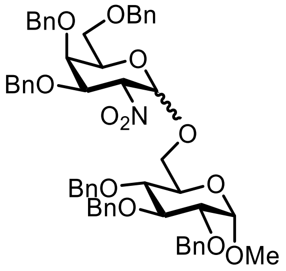

Following the general glycosylation procedure, to the solution of **2h** (40 mg, 0.086 mmol) in 2ml of dry DCM was added CuBr.SMe$_2$ (1 mg, 0.0043 mmol), XPhos (2 mg, 0.0043 mmol) and Cs$_2$CO$_3$ (3 mg, 0.0086 mmol) and this suspension was stirred for 15 min in room temperature. To this mixture was added the solution of **1a** (20 mg, 0.043 mmol) in 1 ml dry DCM. The reaction mixture was stirred for 12h in room temperature following purification by column chromatography (10:1 to 3:1 Heptane: EtOAc) to afford 28 mg of compound **3h** as a colourless oil in 70% yield (α:β 12:1).

3hα

^{1}H NMR (400 MHz, CDCl$_3$) δ 7.38 – 7.14 (m, 30H, Ar*H*), 5.38 (d, *J* = 4.1 Hz, 1H, -H1'), 4.91 (dd, *J* = 10.8, 4.0 Hz, 2H, OCH*H*Ph, H-2'), 4.86 – 4.70 (m, 4H, -OCH*H*Ph), 4.66 (s, 2H), 4.60 (d, *J* = 11.9 Hz, 1H, -OCH*H*Ph), 4.51 (d, *J* = 11.3 Hz, 1H, -OCH*H*Ph), 4.46 (d, *J* = 3.5 Hz, 1H, -H1), 4.44 – 4.38 (m, 2H, -OCH*H*Ph), 4.37 – 4.31 (m, 2H), 3.97 – 3.85 (m, 3H, H-3, H4', H5'), 3.70 (dd, *J* = 12.1, 4.3 Hz, 1H, -H6a), 3.64 – 3.54 (m, 2H, -H5, -H6b), 3.54 – 3.42 (m,

3H, -H2, H6a', H6b'), 3.34 – 3.29 (m, 1H, -H4), 3.27 (s, 3H, OC*H*$_3$). **^{13}C{^{1}H} NMR (101 MHz, CDCl$_3$) δ** 139.0, 138.5, 138.3, 138.1, 137.9, 137.4, 128.7, 128.6, 128.6, 128.5, 128.5, 128.4, 128.3, 128.3, 128.3, 128.2, 128.2, 128.1, 128.1, 128.0, 128.0, 127.9, 127.9, 127.9, 127.8, 127.6 (Ar*C*), 98.1 (C-1), 96.8 (C-1'), 84.4 (C-2'), 81.9, 80.3 (C-2), 75.7 (C-4), 75.2, 75.1, 73.7, 73.6, 73.3, 73.1, 70.4 (C-5), 69.7, 68.3 (C-6'), 66.2 (C-6), 55.2 (O*C*H$_3$).

Spectroscopic data was in agreement with previously reported literature.[7]

Spectroscopic data was in agreement with previously reported literature.[7]

Cyclohexyl 3,4,6-tri-*O*-benzyl-2-deoxy-2-nitro-α-D-galactopyranoside (3i)

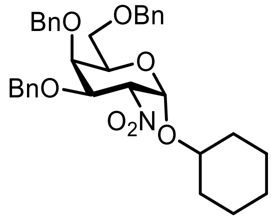

Following the general glycosylation procedure, to the solution of **2i** (9 μL, 0.086 mmol) in 2ml of dry DCM was added CuBr.SMe$_2$ (2 mg, 0.0086 mmol), XPhos (4 mg, 0.0086 mmol) and Cs$_2$CO$_3$ (6 mg, 0.0172 mmol) and this suspension was stirred for 15 min in room temperature. To this mixture was added the solution of **1a** (20 mg, 0.043 mmol) in 1 ml dry DCM. The reaction mixture was stirred for 24h in room temperature following purification by column chromatography (10:1 to 3:1 Heptane: EtOAc) to afford 13 mg of compound **3i** as a colourless oil in 55% yield (α only).

^{1}H NMR (400 MHz, CDCl$_3$) δ 7.40-7.23 (m, 15H, Ar*H*), 5.42 (d, *J* = 4.3 Hz, 1H, -H1), 4.97 (dd, *J* = 10.6, 4.3 Hz, 1H, H-2), 4.85 (d, *J* = 11.2 Hz, 1H, -OCH*H*Ph), 4.80 – 4.71 (m, 2H, -OCH*H*Ph), 4.54 – 4.42 (m, 4H, -OCH*H*Ph, H-3), 4.10 (t, *J* = 6.6 Hz, 1H, -H5), 4.03 (d, *J* = 3.1 Hz, 1H, -H4), 3.66 – 3.51 (m, 3H, H-6, -OC*H*-cylcohexane), 1.87 – 1.57 (m, 6H, cyclohex), 1.53 – 1.31 (m, 3H, cyclohex), 1.23 (d, *J* = 7.5 Hz, 4H, cyclohex). **^{13}C {^{1}H}NMR (101 MHz, CDCl$_3$) δ** 138.1(Ar*C*), 137.8 (Ar*C*), 137.5 (Ar*C*), 128.6 (Ar*C*), 128.5 (Ar*C*), 128.5 (Ar*C*), 128.4 (Ar*C*), 128.3 (Ar*C*), 128.2 (Ar*C*), 128.2 (Ar*C*), 128.0 (Ar*C*), 127.9 (Ar*C*), 127.9 (Ar*C*), 127.8 (Ar*C*), 127.8 (Ar*C*), 94.9 (C-1), 84.7 (C-2), 79.6, 77.2, 75.3, 75.1, 73.8, 73.6, 73.5, 73.3, 73.0, 69.6, 68.4, 33.0, 30.7, 29.7, 25.4, 23.7, 23.4.
HRMS cald. for $C_{33}H_{39}NO_7$ (M+Na)+ 584.2624 found 584.2621.

3-*O*-(2-deoxy-2-nitro-3,4,6-tri-O-benzyl-2-deoxy-α-D-galactopyranosyl)-1,2;5,6-di-*O*-isopropy-lidene-α-D-glucofuranose (3j)

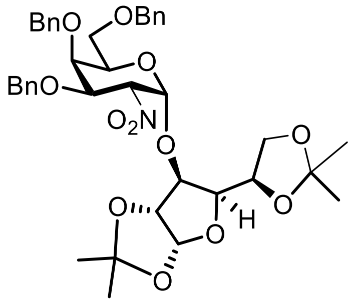

Following the general glycosylation procedure, to the solution of **2j** (22 mg, 0.086 mmol) in 2ml of dry DCM was added CuBr.SMe_2 (2 mg, 0.0086 mmol), XPhos (2 mg, 0.0086 mmol) and Cs_2CO_3 (7 mg, 0.0180 mmol) and this suspension was stirred for 15 min in room temperature. To this mixture was added the solution of **1a** (20 mg, 0.043 mmol) in 1 ml dry DCM. The reaction mixture was stirred for 12h in room temperature following purification by column chromatography (10:1 to 3:1 Heptane: EtOAc) to afford 18 mg of compound **3j** as a colourless oil in 60% yield (α only).

^{1}H NMR (400 MHz, $CDCl_3$) δ 7.41 – 7.21 (m, 15H, Ar*H*), 5.86 (d, *J* = 3.6 Hz, 1H, H-1), 5.62 (d, *J* = 4.2 Hz, 1H, H-1'), 5.01 (dd, *J* = 10.8, 4.2 Hz, 1H, H-2'), 4.85 (d, *J* = 11.3 Hz, 1H, OCH*H*Ph), 4.72 (dd, *J* = 7.4, 3.6 Hz, 3H, -OC*H*HPh, H-2), 4.50-4.35 (m, 4H, OCH*H*Ph, H-3'), 4.19 (d, *J* = 2.0 Hz, 1H, -H4'), 4.13 – 4.06 (m, 1H, -H6a'), 4.04 – 3.94 (m, 4H, H3', H-3, -H4, H5), 3.91 – 3.84 (m, 1H, -H6b'), 3.59 (dd, *J* = 9.6, 6.5 Hz, 1H, -H6a), 3.48 (dd, *J* = 9.7, 5.8 Hz, 1H, -H6b), 1.46 (s, 3H, -CH_3), 1.38 (d, *J* = 8.3 Hz, 6H, -CH_3), 1.18 (s, 3H, -CH_3). **^{13}C{^{1}H} NMR (100 MHz; $CDCl_3$) δ**: 137.8 , 137.7, 137.3, 128.7, 128.6, 128.5, 128.29, 128.27, 128.1, 128.0, 127.9 (ArC), 112.0 (*C*(CH_3)$_2$), 109.5 (*C*(CH_3)$_2$) , 105.3 (C-1), 97.2 (C-1'), 84.0 (C-2'), 83.2 (C-2), 81.9, 81.8 (C-4'), 75.2, 75.1 (C-3'), 73.8, 73.34, 73.30, 71.8, 70.8, 69.1 (C-6), 67.7 (C-6'), 26.83, 26.80, 26.2, 24.9.

Spectroscopic data was in agreement with previously reported literature.[1]

Phenyl 3,4,6-tri-*O*-benzyl-2-deoxy-2-nitro-α-D-thiogalactopyranoside (3k)

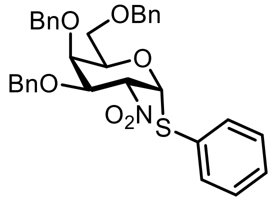

Following the general glycosylation procedure, to the solution of **2k** (10 μL, 0.086 mmol) in 2ml of dry DCM was added CuBr.SMe_2 (1 mg, 0.0043 mmol), XPhos (2 mg, 0.0043 mmol) and Cs_2CO_3 (3 mg, 0.0086 mmol) and this suspension was stirred for 15 min in room temperature. To this mixture was added the solution of **1a** (20 mg, 0.043 mmol) in 1

ml dry DCM. The reaction mixture was stirred for 24h in room temperature following purification by column chromatography (10:1 to 3:1 Heptane: EtOAc) to afford 17 mg of compound **3k** as a colourless oil in 72% yield (α:β 8:1).

^{1}H NMR (400 MHz, $CDCl_3$) δ 7.35 (dq, *J* = 6.8, 1.4 Hz, 2H, Ar*H*), 7.29 – 7.16 (m, 17H, Ar*H*),, 5.76 (d, *J* = 5.9 Hz, 1H, -H1), 5.23 (ddd, *J* = 11.0, 6.0, 1.1 Hz, 1H, -H2), 4.76 (d, *J* = 11.2 Hz, 1H, -OCH*H*Ph), 4.72 – 4.61 (m, 2H, -OCH*H*Ph), 4.45 (t, *J* = 6.5 Hz, 1H, -H5), 4.42 – 4.29 (m, 3H, -OCH*H*Ph), 4.24 (dd, *J* = 11.0, 2.9 Hz, 1H, -H3), 3.96 (d, *J* = 3.0 Hz, 1H, -H4), 3.49 (m, 2H, H-6). **^{13}C{^{1}H} NMR (101 MHz, $CDCl_3$) δ** 138.0, 137.8, 137.2, 133.1, 132.3, 129.3, 128.7, 128.6, 128.5, 128.5, 128.4, 128.3, 128.2, 128.0, 127.9 (Ar*C*), 85.8 (C-1), 84.6 (C-2), 77.4, 76.0, 75.4, 73.7, 73.6 (C-4), 73.3 (C-3), 70.8 (C-5), 68.5 (C-6).

Spectroscopic data was in agreement with previously reported literature.[9]

Dimethyl (3,4,6-tri-*O*-benzyl-2-deoxy-2-nitro-α-D-galactopyranosyl) malonate (3l)

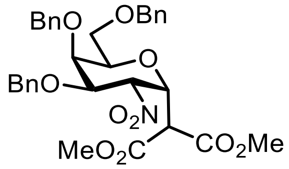

Following the general glycosylation procedure, to the solution of **2l** (9 μL, 0.086 mmol) in 2ml of dry DCM was added $CuBr.SMe_2$ (1 mg, 0.0043 mmol), XPhos (2 mg, 0.0043 mmol) and Cs_2CO_3 (3 mg, 0.0086 mmol) and this suspension was stirred for 15 min in room temperature. To this mixture was added the solution of **1a** (20 mg, 0.043 mmol) in 1 ml dry DCM. The reaction mixture was stirred for 24h in room temperature following purification by column chromatography (10:1 to 3:1 Heptane: EtOAc) to afford 13 mg of compound **3l** as a colourless oil in 55% yield (α only).

^{1}H NMR (400 MHz, $CDCl_3$) δ 7.40 – 7.24 (m, 15H), 5.22 (t, *J* = 10.1 Hz, 1H), 4.84 (d, *J* = 11.2 Hz, 1H), 4.65 (d, *J* = 11.3 Hz, 1H), 4.55 – 4.37 (m, 5H), 4.17 (dd, *J* = 10.3, 2.7 Hz, 1H), 4.05 (d, *J* = 2.7 Hz, 1H), 3.71 (s, 4H), 3.64 (d, *J* = 5.4 Hz, 4H), 3.59 – 3.50 (m, 2H). **^{13}C{^{1}H} NMR (101 MHz, $CDCl_3$) δ** 166.6, 165.9, 138.2, 137.8, 136.8, 128.7, 128.6, 128.4, 128.3, 128.1, 128.0, 128.0, 127.9, 85.7, 80.2, 74.9, 74.8, 73.7, 72.6, 72.0, 67.9, 53.9, 53.0, 52.9.

HRMS cald. for $C_{32}H_{35}NO_{10}$ (M+Na)+ 616.2159 found 616.2143.

Benzyl 6-*O*-tertbutyl dimethyl silyl-3,4-di-*O*-benzyl-2-deoxy-2-nitro-α-D-galactopyranoside (3m)

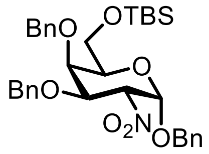

Following the general glycosylation procedure, to the solution of **2a** (9 μL, 0.086 mmol) in 2ml of dry DCM was added CuBr.SMe_2 (1 mg, 0.0043 mmol), XPhos (3 mg, 0.0043 mmol) and Cs_2CO_3 (5 mg, 0.0063 mmol) and this suspension was stirred for 15 min in room temperature. To this mixture was added the solution of **1b** (25 mg, 0.052 mmol) in 1 ml dry DCM. The reaction mixture was stirred for 5h in room temperature following purification by column chromatography (10:1 to 2:1 Heptane: EtOAc) to afford 22 mg of compound **3m** in 75% yield (α only).

^{1}H NMR (400 MHz, $CDCl_3$) δ 7.53 – 7.15 (m, 15H, Ar*H*), 5.39 (d, *J* = 4.2 Hz, 1H, H-1), 5.03 (dd, *J* = 10.6, 4.2 Hz, 1H, H-2), 4.87 (d, *J* = 11.2 Hz, 1H, -OCH*H*Ph), 4.83 – 4.67 (m, 3H, -OC*H*$_2$Ph), 4.59 – 4.47 (m, 3H, H-3, OC*H*$_2$Ph), 3.96 (dd, *J* = 3.1, 1.1 Hz, 1H, H-4), 3.89 – 3.83 (m, 1H, H-5), 3.63 (d, *J* = 6.7 Hz, 2H, H-6), 0.92 (s, 9H), 0.07 (d, *J* = 4.2 Hz, 6H). **^{13}C{^{1}H} NMR (101 MHz, $CDCl_3$) δ** 138.2, 137.6, 136.4, 128.7, 128.6, 128.4, 128.3, 128.2, 128.2, 128.0, 127.9 (Ar*C*), 95.6 (C-1), 84.5 (C-2), 75.4 (C-3), 75.2, 73.4 (C-4), 73.3, 71.7 (C-5), 69.9, 61.5 (C-6), 26.0,18.3, -5.2, -5.3.

HRMS cald. for $C_{33}H_{43}NO_7Si$ (M+H)+ 594.2887 found 594.2901.

Benzyl 6-*O*-acetyl-3,4-di-*O*-benzyl-2-deoxy-2-nitro-α-D-galactopyranoside (3n)

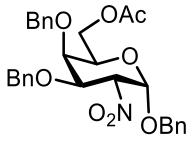

Following the general glycosylation procedure, to the solution of **2a** (9 μL, 0.086 mmol) in 2ml of dry DCM was added CuBr.SMe_2 (1 mg, 0.0043 mmol), XPhos (3 mg, 0.0043 mmol) and Cs_2CO_3 (5 mg, 0.0063 mmol) and this suspension was stirred for 15 min in room temperature. To this mixture was added the solution of **1c** (25 mg, 0.060 mmol) in 1 ml dry DCM. The reaction mixture was stirred for 5h in room temperature following purification by column chromatography (10:1 to 2:1 Heptane: EtOAc) to afford 20 mg of compound **3n** as a colourless oil in 65% yield (α only).

^{1}H NMR (400 MHz, $CDCl_3$) δ 7.38 – 7.21 (m, 15H, Ar*H*), 5.39 (d, *J* = 4.2 Hz, 1H, H-1), 5.02 (dd, *J* = 10.6, 4.2 Hz, 1H, H-2), 4.88 (d, *J* = 11.3 Hz, 1H, -OCH*H*Ph), 4.82 – 4.73 (m, 2H, -OC*H*$_2$Ph), 4.69 (d, *J* = 11.9 Hz, 1H, -OCH*H*Ph), 4.56 – 4.48 (m, 3H, -OC*H*$_2$Ph, H-3), 4.16 (dd, *J* = 10.8, 6.5 Hz, 1H, H-6a), 4.09 – 3.97 (m, 2H, H-5, H-6b), 3.93 – 3.86 (m, 1H, H-4), 2.01 (s, 3H, -COC*H*$_3$). **^{13}C {^{1}H}NMR (101 MHz, $CDCl_3$) δ** 170.5, 137.6, 137.3, 136.1, 128.8, 128.7, 128.6, 128.5, 128.4, 128.37, 128.2, 128.0 (Ar*C*), 95.5 (C-1), 84.2 (C-2), 77.4, 75.5, 75.1, 73.5, 72.9, 70.0, 69.1, 63.0 , 20.9.

HRMS cald. for $C_{29}H_{31}NO_8$ (M+Na)+ 544.1947 found 544.1937.

Benzyl 4,6-Di-O-acetyl-2,3-dideoxy-2-nitro-α- D -threo-hex-2-eno-pyranoside (**3o**)

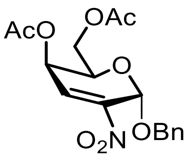

Following the general glycosylation procedure, to the solution of **2a** (9 μL, 0.086 mmol) in 2ml of dry DCM was added CuBr.SMe_2 (1 mg, 0.0043 mmol), XPhos (3 mg, 0.0043 mmol) and Cs_2CO_3 (5 mg, 0.0063 mmol) and this suspension was stirred for 15 min in room temperature. To this mixture was added the solution of **1d** (20 mg, 0.063 mmol) in 1 ml dry DCM. The reaction mixture was stirred for 5h in room temperature following purification by column chromatography (10:1 to 2:1 Heptane: EtOAc) to afford 22 mg of compound **3o** as a colourless oil in 60% yield (α only).

^{1}H NMR (500 MHz, $CDCl_3$) δ 7.38−7.31(m, 5H, Ar*H*), 7.28 (d, J = 5.7 Hz, 1H, H-3), 5.77 (s, 1H,H-1), 5.38 (dd, J = 2.8, 5.7 Hz, 1H, H-4), 4.83 (d, J = 11.2 Hz, 1H,OCHPh), 4.72 (d, J = 11.2 Hz, 1H, OCH*H*Ph), 4.48−4.45 (m, 1H, H-5), 4.29−4.20 (m, 2H, H-6, H-6'), 2.12 (s, 3H, $COCH_3$), 2.09 (s, 3H,COC*H*$_3$). **^{13}C{^{1}H} NMR (125 MHz, $CDCl_3$) δ** 170.4, 169.7, 150.3, 136.1,128.5−127.9 (Ar-C), 91.2, 71.3, 66.3, 62.4, 61.7, 20.7, 20.5.

Spectroscopic data was in agreement with previously reported literature.[10]

Methyl 2,3,4-tri-*O*-benzyl-6-*O*-(3,4,6-tri-*O*-benzyl-2-deoxy-2-nitro-D-glucopyranosyl)-D-glucopyranoside (4a)

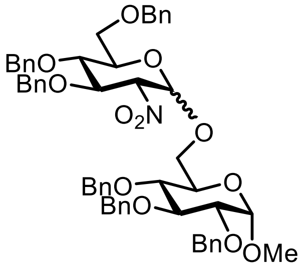

Following the general glycosylation procedure, to the solution of **2h** (40 mg, 0.086 mmol) in 2ml of dry DCM was added CuBr.SMe_2 (2 mg, 0.0086 mmol), XPhos (4 mg, 0.0086 mmol) and Cs_2CO_3 (3 mg, 0.0086 mmol) and this suspension was stirred for 15 min in room temperature. To this mixture was added the solution of **1e** (20 mg, 0.043 mmol) in 1 ml dry DCM. The reaction mixture was stirred for 12h in room temperature following purification by column chromatography (10:1 to 3:1 Heptane: EtOAc) to afford 24 mg of compound **4a** as a colourless oil in 60% yield (α:β 1:8).

4aβ

^{1}H NMR (400 MHz, $CDCl_3$) δ 7.24-7.07 (m, 15H, Ar*H*), 4.89 (d, *J* = 10.8 Hz, 1H), 4.81 – 4.64 (m, 6H), 4.57 (d, *J* = 12.1 Hz, 1H), 4.54 – 4.38 (m, 7H), 4.34 (d, *J* = 11.1 Hz, 1H), 4.17 (dd, *J* = 10.3, 8.9 Hz, 1H), 4.01 (dd, *J* = 10.6, 1.9 Hz, 1H), 3.89 (t, *J* = 9.2 Hz, 1H), 3.70 – 3.65 (m, 1H), 3.63 (m, 2H), 3.60 – 3.52 (m, 2H), 3.49 – 3.41 (m, 2H), 3.37 – 3.20 (m, 4H). **^{13}C{^{1}H} NMR (101 MHz, $CDCl_3$) δ** 138.8, 138.5, 138.3, 137.9, 137.6, 137.0, 128.7, 128.7, 128.6, 128.6, 128.6, 128.5, 128.3, 128.3, 128.2, 128.2, 128.1, 128.1, 128.0, 127.9, 127.9, 127.8, 1278, 127.7, 100.2, 98.3, 89.6, 82.2, 81.6, 79.9, 77.6, 77.6, 75.9, 75.7, 75.6, 75.3, 75.0, 73.6, 73.6, 69.6, 68.9, 68.3, 55.4.

HRMS cald. for $C_{55}H_{59}NO_{12}$ (M+Na)+ 948.3935 found 948.3925.

1,2;3,4-Di-*O*-isopropylidene-6-*O*-(3,4,6-tri-O-benzyl-2-deoxy-2-nitro-D-glucopyranosyl)-α-D-galactopyranose (4b)

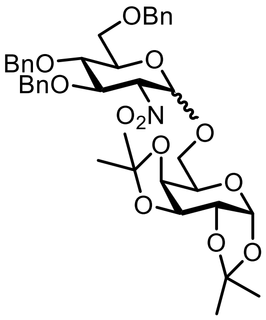

Following the general glycosylation procedure, to the solution of **2g** (22 mg, 0.086 mmol) in 2ml of dry DCM was added CuBr.SMe_2 (2 mg, 0.0086 mmol), XPhos (4 mg, 0.0086 mmol) and Cs_2CO_3 (8 mg, 0.022 mmol) and this suspension was stirred for 15 min in room temperature. To this mixture was added the solution of **1e** (20 mg, 0.043 mmol) in 1 ml

dry DCM. The reaction mixture was stirred for 12h in room temperature following purification by column chromatography (10:1 to 3:1 Heptane: EtOAc) to afford 15 mg of compound **4b** as a colourless oil in 51% yield (α:β 1:4).

4bβ

^{1}H NMR (400 MHz, $CDCl_3$) δ 7.37 – 7.19 (m, 15H, Ar*H*), 5.49 (d, *J* = 4.9 Hz, 1H, H-1), 4.99 (d, *J* = 8.0 Hz, 1H, H-1'), 4.76 (dd, *J* = 16.7, 10.8 Hz, 2H, -OCH_2Ph), 4.66 – 4.47 (m, 7H,H-2', H-3, -OCH_2Ph), 4.34 – 4.24 (m, 2H, H-2, H-3'), 4.17 (dd, *J* = 8.0, 1.9 Hz, 1H, H-4), 3.99 (dd, *J* = 11.3, 4.7 Hz, 1H, H-6a), 3.90 (ddd, *J* = 6.6, 4.6, 1.9 Hz, 1H, H-5), 3.84 – 3.70 (m, 4H, H-4', H-6',H-6b), 3.57 (dt, *J* = 9.8, 2.9 Hz, 1H, H-5'), 1.54 (s, 3H, -C(CH_3)$_2$), 1.43 (s, 3H, -C(CH_3)$_2$), 1.32 (2s, 6H, -C(CH_3)$_2$). **^{13}C{^{1}H} NMR (101 MHz, $CDCl_3$) δ** 138,0, 137.7, 137.2, 128.7, 128.6, 128.6, 128.6, 128.2, 128.2, 128.1, 128.0, 127.9, 127.9 (Ar*C*), 1095, 109.0, 99.9 (C-1'), 96.3 (C-1), 89.8 (C-2'), 81.7 (C-2), 77.7, 77.4, 75.6, 75.4, 75.2, 73.7, 71.2, 70.8, 70.6, 68.5 (C-6), 68.1 (C-5'), 67.6 (C-6), 26.1(-*C*(CH_3)$_2$), 26.1(-*C*(CH_3)$_2$), 25.1(-*C*(CH_3)$_2$), 24.5 (-*C*(CH_3)$_2$).

Spectroscopic data was in agreement with previously reported literature.[1]

4bα

^{1}H NMR (400 MHz, $CDCl_3$) δ 7.35 – 7.15 (m, 15*H*),, 5.48 (d, *J* = 4.9 Hz, 1H, H-1), 5.37 (d, *J* = 3.7 Hz, 1H, H-1'), 4.88 (d, *J* = 2.7 Hz, 2H, OCH*H*Ph), 4.80 (d, *J* = 10.9 Hz, 1H, OCH*H*Ph), 4.67 – 4.54 (m, 5H, H-3, H-2', H-3', OCH_2Ph), 4.50 (d, *J* = 12.0 Hz, 1H, -OCH*H*Ph), 4.29 (dd, *J* = 5.0, 2.3 Hz, 1H, H-2), 4.09 (dd, *J* = 8.0, 1.9 Hz, 1H, H-4'), 3.98 – 3.87 (m, 2H, H-5, H-6a), 3.85 – 3.76 (m, 3H, H-4', H-5', H-6a'), 3.68 (dd, *J* = 11.0, 2.0 Hz, 1H, H-6b), 3.62 (dd, *J* = 10.1, 6.9 Hz, 1H, H-6b'), 1.54 (s, 3H, -C(CH_3)$_2$), 1.41 (s, 3H, -C(CH_3)$_2$), 1.33 (2s, 6H, -C(CH_3)$_2$). **^{13}C{^{1}H} NMR (101 MHz, $CDCl_3$) δ** 138.0, 137.9, 137.8, 128.6, 128.60, 128.6, 128.1, 128.1, 128.0, 128.1, 127.9, 127.8 (Ar*C*), 109.4 , 108.8, 96.9 (C-1), 96.3 (C-1'), 87.0 (C-2), 78.38, 77.4, 76.0, 75.2, 73.7, 70.9, 70.8, 70.6, 70.6, 67.9, 67.4, 66.3, 26.3 (-*C*(CH_3)$_2$), 26.0 (-*C*(CH_3)$_2$), 25.1 (-*C*(CH_3)$_2$), 24.3 (-*C*(CH_3)$_2$).

Spectroscopic data was in agreement with previously reported literature.[1]

Allyl 3,4,6-tri-*O*-benzyl-2-deoxy-2-nitro-β-D-glucopyranoside (4c)

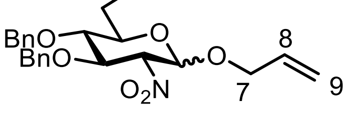

Following the general glycosylation procedure, to the solution of **2m** (6 μL, 0.086 mmol) in 2ml of dry DCM was added $CuBr.SMe_2$ (1 mg, 0.0043 mmol), XPhos (2 mg, 0.0043 mmol) and Cs_2CO_3 (3 mg, 0.0086 mmol) and this suspension was stirred for 15 min in room temperature. To this mixture was added the solution of **1e** (20 mg, 0.043 mmol) in 1

ml dry DCM. The reaction mixture was stirred for 5h in room temperature following purification by column chromatography (10:1 to 2:1 Heptane: EtOAc) to afford 23 mg of compound **4c** as a colourless oil in 63% yield (β only).

^{1}H NMR (400 MHz, $CDCl_3$) δ 7.40 – 7.25(m, 15H, Ar*H*), 5.82 (m, 1H, -H-8), 5.31 – 5.16 (m, 2H, H-9), 4.93 – 4.72 (m, 3H, H-1, -OC*H*$_2$Ph), 4.66 – 4.48 (m, 5H, H-2, OC*H*$_2$Ph), 4.34 (ddt, *J* = 12.8, 5.1, 1.5 Hz, 1H, H-7a), 4.25 (dd, *J* = 10.4, 9.0 Hz, 1H, H-3), 4.09 (ddt, *J* = 12.7, 6.4, 1.4 Hz, 1H, H-7b), 3.80 – 3.67 (m, 3H, H-4, H-6), 3.55 (dt, *J* = 9.8, 3.1 Hz, 1H, H-5). **^{13}C{^{1}H} NMR (101 MHz, $CDCl_3$) δ** 137.9, 137.7, 137.10, 132.9, 132.6, 128.6, 128.6, 128.6, 128.6, 128.3, 128.2, 128.16, 128.1, 128.0, 128.0, 127.9, 127.9 (Ar*C*), 118.5, 98.8 (C-1), 89.8 (C-2), 81.6 (C-3), 78.5, 77.7 (C-4), 77.4, 76.0, 75.6 (C-5), 75.5, 75.3, 73.8, 73.7, 70.4 (CH, 68.2 (C-6)Spectroscopic data was in agreement with previously reported literature.[11]

_p_-Methoxybenzyl 3,4,6-tri-_O_-benzyl-2-deoxy-2-nitro-β-D-glucopyranoside (4d)

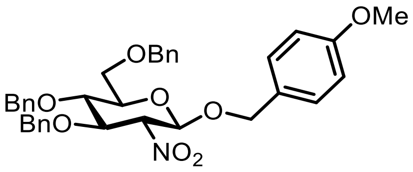

Following the general glycosylation procedure, to the solution of **2n** (12 mg, 0.086 mmol) in 2ml of dry DCM was added CuBr.SMe$_2$ (2 mg, 0.0086 mmol), XPhos (4 mg, 0.0086mmol) and Cs_2CO_3 (8 mg, 0.017 mmol) and this suspension was stirred for 15 min in room temperature. To this mixture was added the solution of **1e** (20 mg, 0.043 mmol) in 1 ml dry DCM. The reaction mixture was stirred for 5h in room temperature following purification by column chromatography (10:1 to 3:1 Heptane: EtOAc) to afford 17 mg of compound **4d** as a colourless oil in 66% yield (α:β 1:16).

^{1}H NMR (400 MHz, $CDCl_3$) δ 7.36 – 7.2 (m, 17H, Ar*H*), 6.89 – 6.83 (m, 2H, Ar*H*), 4.85 – 4.75 (m, 3H, H-1, -OC*H*$_2$PhOMe), 4.72 (d, *J* = 10.7 Hz, 1H, -OCH*H*Ph), 4.66 – 4.56 (m, 4H, H-2, -OC*H*$_2$Ph), 4.56 – 4.54 (m, 2H, -OCH$_2$Ph), 4.52 (d, *J* = 3.1 Hz, 1H, H-3), 4.21 (dd, *J* = 10.3, 8.9 Hz, 1H), 3.80 (s, 3H), 3.73 (dd, *J* = 3.9, 2.2 Hz, 2H), 3.52 (dt, *J* = 9.8, 3.2 Hz, 1H). **^{13}C{^{1}H} NMR (101 MHz, $CDCl_3$) δ** 159.7, 138.0, 137.7, 137.1, 129.9, 129.7, 128.6, 128.6, 128.6, 128.3, 128.2, 128.1, 128.1, 127.9, 127.7, 114.0, 98.2, 89.8, 81.6, 77.8, 77.4, 75.6, 75.4, 75.2, 73.7, 70.9, 68.3, 55.4.

Spectroscopic data was in agreement with previously reported literature.[11]

Methyl 2-deoxy-4,6-di-*O*-benzyl-α-D-galacto--hex-3-ulopyranoside (5a)

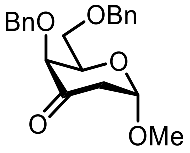

Following the general glycosylation procedure, to the solution of **2o** (5 μL, 0.12 mmol) in 2ml of dry DCM was added $CuBr.SMe_2$ (1.5 mg, 0.0061 mmol), XPhos (3 mg, 0.0061 mmol) and Cs_2CO_3 (4 mg, 0.012 mmol) and this suspension was stirred for 15 min in room temperature. To this mixture was added the solution of **1f** (20 mg, 0.061 mmol) in 1 ml dry DCM. The reaction mixture was stirred for 1h in room temperature following purification by column chromatography (10:1 to 3:1 Heptane: EtOAc) to afford 19 mg of compound as a colourless oil in 90% yield.

^{1}H NMR (400 MHz, $CDCl_3$) δ 7.47 – 7.09 (m, 10H, Ar*H*), 5.11 (dd, *J* = 4.5, 1.5 Hz, 1H, H-1), 4.56 (dd, *J* = 11.9, 3.0 Hz, 2H, OCH_2Ph), 4.46 (d, *J* = 11.9 Hz, 1H, -OCH*H*Ph), 4.34 (d, *J* = 11.8 Hz, 1H, -OCH*H*Ph), 4.18 (dd, *J* = 2.1, 0.6 Hz, 1H, H-5), 3.75 – 3.67 (m, 3H, H-4, H-6), 3.33 (s, 3H, -OCH_3), 3.13 (dd, *J* = 13.9, 4.4 Hz, 1H, H-2_{ax}), 2.40 (dt, *J* = 13.9, 1.4 Hz, 1H, H-2_{eq}). **^{13}C{^{1}H} NMR (101 MHz, $CDCl_3$) δ** 204.0 (C-3), 138.1, 137.0, 128.6, 128.5, 128.3, 128.2, 127.8, 127.7 (Ar*C*), 100.0 (C-1), 79.8 (C-4), 73.6, 72.2, 71.4 (C-5), 68.8 (C-6), 55.2 (OCH_3), 44.1 (C-2).

HRMS cald. for $C_{21}H_{24}O_5$ (M+Na)+ 379.1521 found 379.1530.

1-Benzyl-2-deoxy-4,6-di-*O*-benzyl-α-D-galacto-hex-3-ulopyranoside (5b)

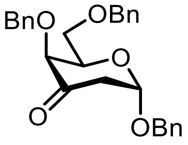

Following the general glycosylation procedure, to the solution of **2a** (12 μL, 0.12 mmol) in 2ml of dry DCM was added $CuBr.SMe_2$ (1.5 mg, 0.0061 mmol), XPhos (3 mg, 0.0061 mmol) and Cs_2CO_3 (4 mg, 0.012 mmol) and this suspension was stirred for 15 min in room temperature. To this mixture was added the solution of **1f** (20 mg, 0.061 mmol) in 1 ml dry DCM. The reaction mixture was stirred for 1h in room temperature following purification by column chromatography (10:1 to 3:1 Heptane: EtOAc) to afford 20 mg of compound as a colourless oil in 78% yield.

^{1}H NMR (400 MHz, CDCl$_3$) δ 7.39 – 7.21 (m, 15H, Ar*H*), 5.31 (dd, *J* = 4.6, 1.5 Hz, 1H, H-1), 4.70 (d, *J* = 12.0 Hz, 1H, OCH*H*Ph), 4.63 – 4.42 (m, 4H, OC*H*$_2$Ph), 4.37 (d, *J* = 11.9 Hz, 1H, -OCH*H*Ph), 4.28 (td, *J* = 6.0, 2.1 Hz, 1H, H-5), 3.79 – 3.65 (m, 3H, H-4, H-6), 3.15 (dd, *J* = 14.1, 4.6 Hz, 1H, H-2$_{ax}$), 2.46 (dt, *J* = 14.1, 1.4 Hz, 1H, H-2$_{eq}$). **^{13}C{^{1}H} NMR (101 MHz, CDCl$_3$) δ** 203.9 (C-3), 137.8, 137.4, 136.8, 128.4, 128.3, 128.2, 127.9, 127.8, 127.8, 127.7 (Ar*C*), 97.7 (C-1), 79.0 (C-4), 77.1, 73.6, 73.5, 72.5 (C-5), 69.0, 68.4 (C-6), 46.5 (C-2).

HRMS cald. for $C_{27}H_{28}O_5$ (M+Na)+ 455.1834 found 455.1840

Methyl 2-deoxy-4,6-di-*O*-benzyl-α-D-gluco-hex-3-ulopyranoside (5c)

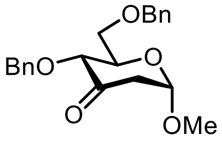

Following the general glycosylation procedure, to the solution of **2o** (5 μL, 0.12 mmol) in 2ml of dry DCM was added CuBr.SMe$_2$ (1.5 mg, 0.0061 mmol), XPhos (3 mg, 0.0061 mmol) and Cs_2CO_3 (4 mg, 0.012 mmol) and this suspension was stirred for 15 min in room temperature. To this mixture was added the solution of **1g** (20 mg, 0.061 mmol) in 1 ml dry DCM. The reaction mixture was stirred for 1h in room temperature following purification by column chromatography (10:1 to 3:1 Heptane: EtOAc) to afford 17 mg of compound as a colourless oil in 85% yield (α:β >30:1).

^{1}H NMR (400 MHz, CDCl$_3$) δ 7.27 – 7.18 (m, 10H, Ar*H*)), 5.06 (dd, *J* = 4.6, 1.3 Hz, 1H, H-1)), 4.83 (d, *J* = 11.0 Hz, 1H, OCH*H*Ph), 4.55 (d, *J* = 12.1 Hz, 1H, -OCH*H*Ph), 4.45 (d, *J* = 12.1 Hz, 1H, -OCH*H*Ph), 4.35 (d, *J* = 11.0 Hz, 1H, -OCH*H*Ph), 4.11 (dd, *J* = 9.8, 1.2 Hz, 1H, H-4), 3.97 – 3.87 (m, 1H, H-5), 3.72 (dd, *J* = 10.6, 3.4 Hz, 1H, H-6a), 3.69 – 3.61 (m, 1H, H-6b), 3.27 – 3.23 (s, 3H, OC*H*$_3$), 2.72 (ddd, *J* = 14.1, 4.5, 1.1 Hz, 1H, H-2$_{ax}$), 2.53 (dd, *J* = 14.1, 1.2 Hz, 1H, H-2$_{eq}$). **^{13}C{^{1}H} NMR (101 MHz, CDCl$_3$) δ** 204.2 (C-3), 138.0, 137.6, 128.7, 128.6, 128.6, 128.5, 128.4, 128.1, 128.1, 128.0, 127.9, 127.9 (Ar*C*), 99.9 (C-1), 79.1 (C-4), 73.8, 73.6, 72.5 (C-5), 68.6 (C-6), 55.0 (O*C*H$_3$), 46.8 (C-2).

Spectroscopic data was in agreement with previously reported literature.[12]

Benzyl 2-deoxy-4,6-di-*O*-benzyl-α-D-gluco-hex-3-ulopyranoside (5d)

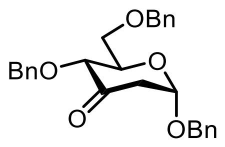

Following the general glycosylation procedure, to the solution of **2a** (12 μL, 0.12 mmol) in 2ml of dry DCM was added CuBr.SMe$_2$ (1.5 mg, 0.0061 mmol), XPhos (3 mg, 0.0061 mmol) and Cs$_2$CO$_3$ (4 mg, 0.012 mmol) and this suspension was stirred for 15 min in the room temperature. To this mixture was added the solution of **1g** (20 mg, 0.061 mmol) in 1 ml dry DCM. The reaction mixture was stirred for 1h at room temperature following purification by column chromatography (10:1 to 3:1 Heptane: EtOAc) to afford 19 mg of compound as a colourless oil in 73% yield (α:β >30:1).

^{1}H NMR (400 MHz, CDCl$_3$) δ 7.38 – 7.27 (m, 15H), 5.34 – 5.27 (m, 1H, H-1), 4.90 (d, *J* = 11.0 Hz, 1H, -OCH*H*Ph), 4.64 (dd, *J* = 18.9, 12.1 Hz, 2H, -OC*H*$_2$Ph), 4.51 (dd, *J* = 12.2, 7.1 Hz, 2H, -OC*H*$_2$Ph), 4.42 (d, *J* = 11.0 Hz, 1H, -OCH*H*Ph), 4.21 (dd, *J* = 9.9, 1.1 Hz, 1H, H-4), 4.08 (ddd, *J* = 9.8, 3.3, 1.9 Hz, 1H, H-5), 3.83 – 3.75 (m, 1H, H-6a), 3.69 (dd, *J* = 10.6, 2.0 Hz, 1H, H-6b), 2.81 (ddd, *J* = 14.2, 4.6, 1.1 Hz, 1H, H-2$_{ax}$), 2.65 (dd, *J* = 14.1, 1.3 Hz, 1H, H-2$_{eq}$). **^{13}C{^{1}H} NMR (101 MHz, CDCl$_3$) δ** 204.1 (C-3), 138.0, 137.6, 137.0, 128.6, 128.5, 128.4, 128.1, 128.0, 128.0, 127.9 (Ar*C*), 97.9 (C-1), 79.2 (C-4), 77.4, 73.8, 73.7, 72.8 (C-5), 69.2, 68.6 (C-6), 46.7 (C-2).

Spectroscopic data was in agreement with previously reported literature.[13]

Benzyl 3,4,6-tri-O-benzyl-2-deoxy-2-formyl-α-D-galactopyranoside (6)

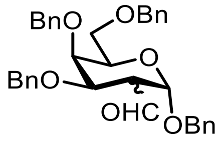

Following the general glycosylation procedure, to the solution of **2a** (10 μL, 0.09 mmol) in 2ml of dry DCM was added CuBr.SMe$_2$ (2 mg, 0.009 mmol), XPhos (4 mg, 0.009 mmol) and Cs$_2$CO$_3$ (8 mg, 0.023 mmol) and this suspension was stirred for 15 min at room temperature. To this mixture was added the solution of **1h** (20 mg, 0.045 mmol) in 1 ml dry DCM. The reaction mixture was stirred for 1h at room temperature following purification by column chromatography (10:1 to 3:1 Heptane: EtOAc) to afford 10 mg of compound as a colourless oil in 42% yield (cis:trans 2:1).

^{1}H NMR (600 MHz, CDCl$_3$) δ 9.91 (s, 0.5H), 9.60 (dd, *J* = 2.8, 0.9 Hz, 1H), 7.42 – 7.20 (m, 30H), 5.47 (s, 0.5H), 5.20 (d, *J* = 3.9 Hz, 1H), 4.92 (d, *J* = 11.6 Hz, 0.5H), 4.88 (d, *J* = 11.4 Hz,

1H), 4.75 (d, J = 11.6 Hz, 1H), 4.71 (d, J = 15.6 Hz, 1H), 4.67 (d, J = 10.0 Hz, 1H), 4.64 (s, 0.5H), 4.60 – 4.55 (m, 2.7H), 4.54 – 4.49 (m, 1.5 H), 4.49 – 4.44 (m, 2.6H), 4.39 (d, J = 11.6 Hz, 0.5H), 4.36 (dd, J = 11.4, 2.5 Hz, 1H), 4.27 (dd, J = 5.9, 2.2 Hz, 0.5H) 4.08 (d, J = 2.5 Hz, 1H), 4.02 (t, J = 6.6 Hz, 1H), 4.00 – 3.89 (m, 1H), 3.64 (dd, J = 9.1, 7.4 Hz, 1H), 3.62 – 3.58 (m, 0.5H), 3.56 (dd, J = 9.2, 5.8 Hz, 1H), 3.52 (dd, J = 9.1, 5.6 Hz, 0.5 H), 3.18 (dt, J = 11.4, 3.4 Hz, 1H), 2.86 (d, J = 5.8 Hz, 0.5H). **$^{13}C\{^1H\}$ NMR (151 MHz, $CDCl_3$) δ** 201.1, 199.7 , 138.48, 138.3 , 138.1 , 138.0 , 137.9, 137.7 , 137.5, 137.2 , 128.7, 128.6, 128.6, 128.6, 128.5,128.5, 128.4, 128.2, 128.2, 128.2, 128.2, 128.1, 128.1, 128.0, 128.0, 128.0, 127.9, 127.9, 127.8, 127.7, 127.5, 97.1, 96.1, 76.7, 74.8, 74.7, 74.6, 73.7, 73.6, 73.4, 71.6, 71.6, 71.2, 70.2, 70.1, 69.6, 69.5, 69.1, 69.0, 53.1, 52.3.

HRMS cald. for $C_{35}H_{36}O_6$ (M+Na)+ 575.2410 found 575.2404.

Synthesis of mucin core 8

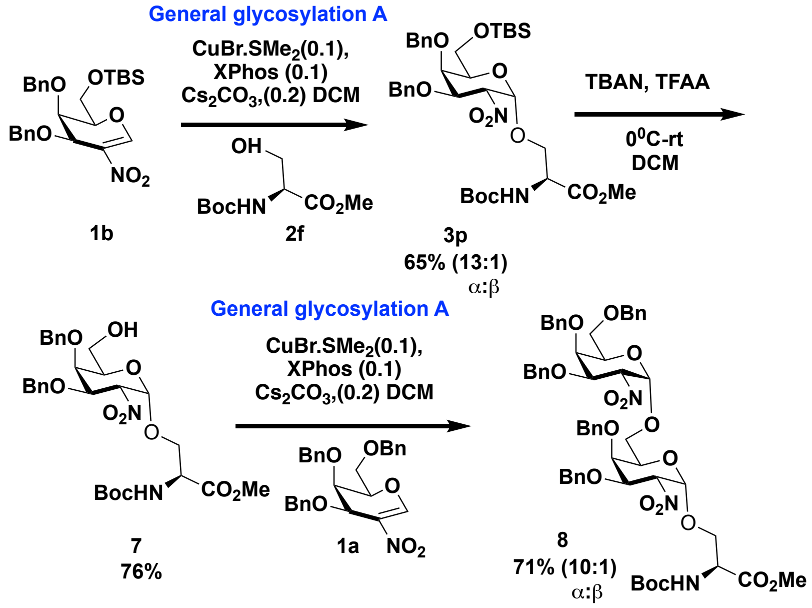

Scheme S1. Synthesis of mucin type core **8**.

N-Boc-L-serine methyl ester 2-deoxy-2-nitro-3,4-di-*O*-benzyl-6-*O*-tertbutyl dimethyl silyl-α-D-galactopyranoside (3p)

Following the general glycosylation procedure A, to the solution of n-Boc-Serine (451 mg, 2.06 mmol) in 10 ml of dry DCM was added $CuBr.SMe_2$ (22 mg, 0.1 mmol), XPhos (50 mg, 0.1 mmol) and Cs_2CO_3 (70 mg, 0.2 mmol) and this suspension was stirred for 15 min at room temperature. To this mixture was added the solution of **1b** (500 mg, 1.03 mmol) in 3 ml dry DCM. The reaction mixture was stirred for 12 h at room temperature following

purification by column chromatography (10:1 to 3:1 Heptane: EtOAc) to afford 471 mg of 3**p** as a colourless oil at 65% yield.

^{1}H NMR (400 MHz, CDCl$_3$) δ 7.42 – 7.21 (m, 10H, Ar*H*), 5.26 (d, *J* = 4.1 Hz, 1H, H-1), 5.19 (d, *J* = 8.8 Hz, 1H), 4.98 (dd, *J* = 10.6, 4.1 Hz, 1H, H-2), 4.84 (d, *J* = 11.0 Hz, 1H), 4.80 – 4.68 (m, 2H, OCH$_2$Ph), 4.48 (d, *J* = 11.0 Hz, 2H, OCH$_2$Ph), 4.35 (dd, *J* = 10.6, 3.0 Hz, 1H, H-3), 4.10 (dd, *J* = 9.9, 2.9 Hz, 1H, H-6a), 3.88 (d, *J* = 2.9 Hz, 1H, H-4), 3.76 (s, 3H, OC*H*$_3$), 3.70 – 3.56 (m, 4H, H-5, H-6b, OC*H*$_2$-Ser), 1.46 (s, 9H), 0.88 (s, 9H), 0.05 (d, *J* = 4.2 Hz, 6H). **^{13}C{^{1}H} NMR (101 MHz, CDCl$_3$)** δ 170.6, 138.2, 137.5, 128.7, 128.5, 128.3, 128.1, 128.0 (Ar*C*), 96.4 (C-1), 84.3 (C-2), 80.5, 77.4, 75.3 (C-3), 75.1, 73.3, 73.2 (C-4), 71.8 (C-5), 68.4 (C-6), 61.2, 53.7, 52.7, 28.4, 26.0, 18.3, -5.3, -5.4.

HRMS cald. for $C_{35}H_{52}N_2O_{11}Si$ (M+Na)+ 727.3238 found 727.3222.

Compound **3p** (400 mg, 0.57 mmol) was then further dissolved in 20 ml of THF and treated with 0.6 ml of TBAF and let it stir for 1h until TLC showed full consumption of the starting material. The reaction mixture was diluted with EtOAc and the combined organic extracts were washed with NH4Cl solution (2 × 20mL, water (1 × 20mL) and brine (1 × 20 mL) and then dried over Na_2SO_4. The organic layer was concentrated in vacuo that afforded 318 mg of compound **7** as a colourless oil at 97% yield and was used for the next step with our further purification.

Following the general glycosylation procedure, to the solution of **7** (50 mg, 0.084 mmol) in 4ml of dry DCM was added CuBr.SMe$_2$ (2 mg, 0.008 mmol), XPhos (4 mg, 0.008 mmol) and Cs_2CO_3 (7 mg, 0.016 mmol) and this suspension was stirred for 15 min at room temperature. To this mixture was added the solution of **1a** (20 mg, 0.04 mmol) in 1 ml dry DCM. The reaction mixture was stirred for 5h at room temperature following purification by column chromatography (10:1 to 3:1 Heptane: EtOAc) to afford 32 mg of compound **8** as a colourless oil at 71% yield.

***N*-Boc-L-serine methyl ester 2-deoxy-2-nitro-3,4-di-*O*-benzyl-6-*O*-(2-deoxy-2- nitro-3,4,6-tri-*O*-benzyl-D-galactopyranosyl)-α-D-galactopyranoside** (**8**)

^{1}H NMR (600 MHz, CDCl$_3$) δ 7.39 – 7.22 (m, 25H, Ar*H*), 7.22 (ddt, *J* = 8.3, 3.7, 2.1 Hz, 4H), 5.23 (d, *J* = 4.2 Hz, 1H), 5.18 (d, *J* = 4.4 Hz, 1H, H-1'), 5.02 – 4.98 (m, 1H, H-2'), 4.97 – 4.91 (m, 1H, H-2), 4.84 (dt, *J* = 11.2, 8.0 Hz, 2H, -OC*H*$_2$Ph), 4.73 (d, *J* = 12.0 Hz, 4H, OC*H*$_2$Ph), 4.51 – 4.45 (m, 3H), 4.41 (dd, *J* = 11.8, 2.2 Hz, 2H, H-3, H-3'), 4.35 (dd, *J* = 11.8, 3.2 Hz, 2H, OC*H*$_2$Ph), 4.06 – 3.95 (m, 3H), 3.81 – 3.75 (m, 2H), 3.72 (m, 4H), 3.62 (dd, *J* = 10.1, 3.6 Hz, 1H), 3.54 (ddd, *J* = 14.6, 9.4, 5.3 Hz, 3H), 3.35 (dd, *J* = 9.6, 6.5 Hz, 1H), 1.46 (s, 9H). **^{13}C{^{1}H} NMR (151 MHz, CDCl$_3$) δ** 170.6, 170.5, 155.4, 138.0, 137.8, 137.8, 137.7, 137.3, 137.3, 137.2, 128.7, 128.7, 128.7, 128.6, 128.6, 128.6, 128.5, 128.5, 128.5, 128.4, 128.4, 128.3, 128.3, 128.3, 128.1, 128.1, 128.1, 128.1, 128.0, 128.0, 96.6, 96.6, 84.4, 84.2, 84.1, 80.5,

75.3, 75.2, 75.2, 75.1, 74.9, 73.7, 73.7, 73.2, 73.2, 73.1, 73.1, 73.0, 73.0, 70.2, 70.1, 69.7, 68.8, 68.6, 68.2, 66.7, 53.8, 52.8, 52.7, 28.4.

One pot synthesis of the trisaccharide:

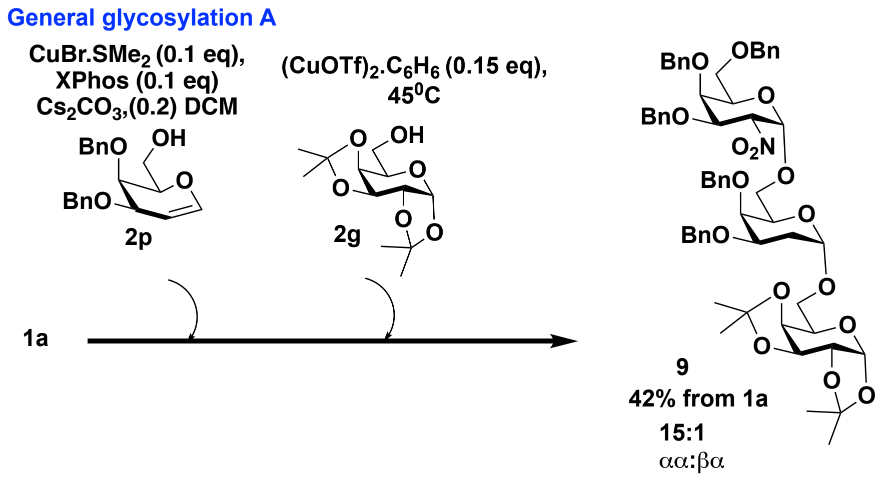

Scheme S2. One pot synthesis of trisaccharide **9**.

Following the general glycosylation procedure, to the solution of **2p** (28 mg, 0.086 mmol) in 2ml of dry DCM was added $CuBr.SMe_2$ (1 mg, 0.0043 mmol), XPhos (2 mg, 0.0043 mmol) and Cs_2CO_3 (3 mg, 0.0086 mmol) and this suspension was stirred for 15 min at room temperature. To this mixture was added the solution of **1a** (20 mg, 0.043 mmol) in 1 ml dry DCM. The reaction mixture was stirred for 12h at room temperature. As TLC showed satisfactory consumption of the starting material, $(CuOTf)_2.C_6H_6$ (4mg, 0.0086 mmol) and **2g** (22mg, 0.086 mmol) was added to the reaction mixture and it was warmed up to 45 ^{0}C. It was stirred at that temperature for another 2h until the intermediate **3q** got consumed. The solvent was evaporated, and the crude was purified by column chromatography that afforded 19 mg of the trisaccharide **9** as a colourless oil in 42% overall yield.

^{1}H NMR (600 MHz, $CDCl_3$) δ 7.37 – 7.27 (m, 23H), 7.22 (tt, *J* = 4.9, 1.9 Hz, 4H), 5.52 (d, *J* = 5.0 Hz, 1H), 5.21 (d, *J* = 4.1 Hz, 1H), 5.02 – 4.96 (m, 2H), 4.94 (d, *J* = 11.3 Hz, 1H), 4.83 (d, *J* = 11.1 Hz, 1H), 4.75 (d, *J* = 5.9 Hz, 2H), 4.63 – 4.57 (m, 3H), 4.51 – 4.47 (m, 2H), 4.47 – 4.43 (m, 2H), 4.40 (d, *J* = 11.7 Hz, 2H), 4.28 (dd, *J* = 5.0, 2.4 Hz, 1H), 4.23 (dd, *J* = 7.9, 1.9 Hz, 1H), 4.09 – 4.02 (m, 2H), 3.98 – 3.94 (m, 1H), 3.92 (d, *J* = 4.0 Hz, 1H), 3.87 (t, *J* = 6.5 Hz, 1H), 3.78 – 3.70 (m, 3H), 3.67 – 3.62 (m, 1H), 3.60 (d, *J* = 8.3 Hz, 1H), 3.55 (dd, *J* = 8.9, 5.4 Hz, 1H), 3.45 (dd, *J* = 9.3, 7.3 Hz, 1H), 2.21 (td, *J* = 12.4, 3.7 Hz, 1H), 2.01 (dd, *J* = 12.7, 4.6 Hz, 1H), 1.54 (s, 3H), 1.43 (s, 4H), 1.32 (s, 3H), 1.30 (s, 3H). **^{13}C{^{1}H} NMR (151 MHz, $CDCl_3$) δ** 138.9, 138.6, 138.2, 137.9, 137.6, 128.7, 128.7, 128.6, 128.6, 128.6, 128.5, 128.5, 128.5, 128.5, 128.4, 128.4, 128.4, 128.3, 128.3, 128.3, 128.25, 128.2, 128.2, 128.1, 128.1, 128.0, 128.0, 128.0, 127.9, 127.6, 127.6, 127.5, 109.4, 108.71, 97.8, 96.6, 96.5, 96.5,

84.5, 75.4, 75.3, 74.7, 74.4, 73.2, 73.3, 73.2, 72.8, 71.8, 71.2, 70.9, 70.8, 70.8, 70.7, 70.6, 69.6, 69.5, 68.2, 68.0, 67.5, 66.0, 65.8, 62.6, 26.3, 26.2, 26.1, 25.1, 24.7.

HRMS cald. for $C_{59}H_{69}NO_{16}$ (M+Na)+ 1070.4514 found 1070.4506.

Mechanistic Study

NMR Experiment: Nucleophile acceptor **2h** has been treated sequentially with the catalyst, ligand and the base at 1:1 stoichiometric ratio and the ^{1}H NMR has been recorded at 25 ^{0}C in CD_2Cl_2.

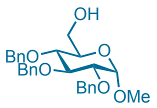

(a)

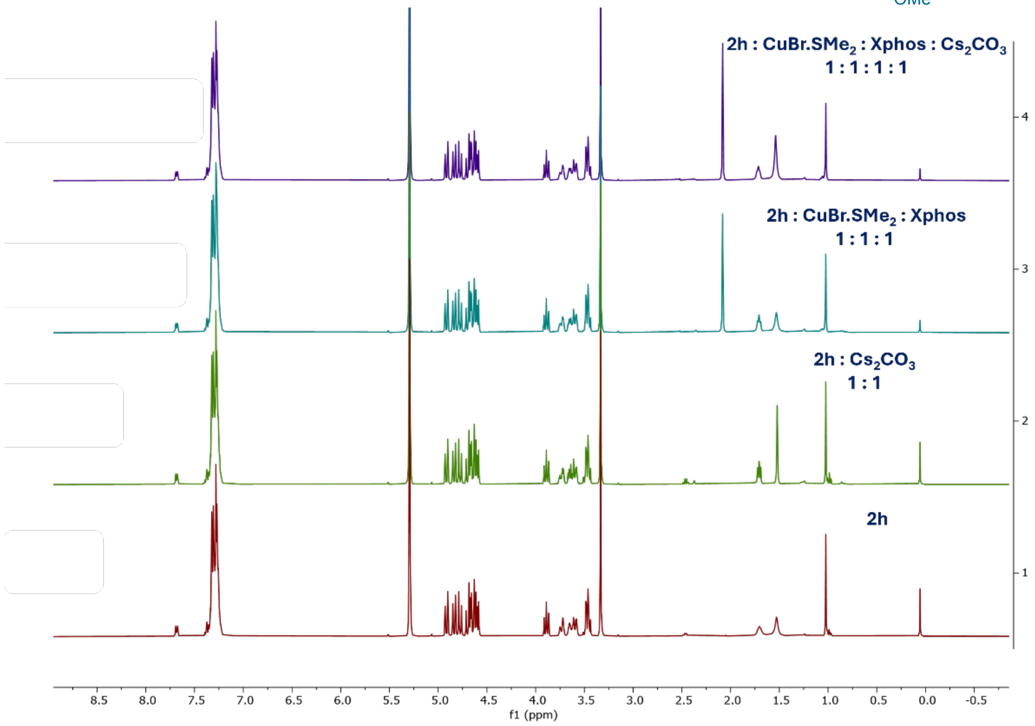

(b)

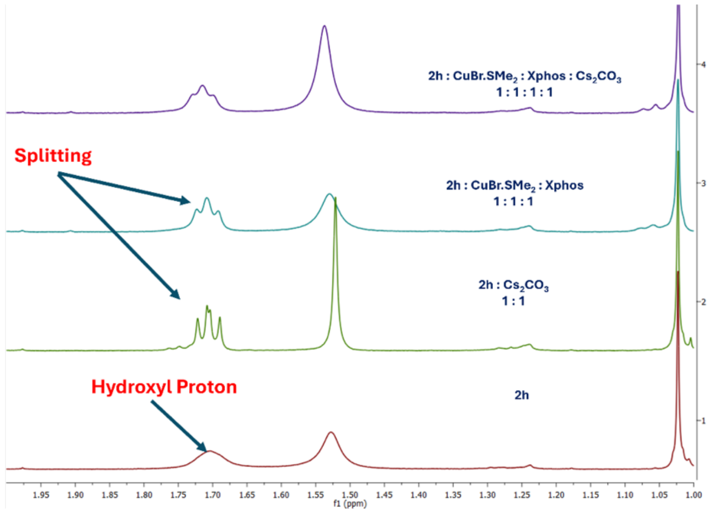

Figure S1. (a) ^{1}H NMR study with acceptor **2h** and catalyst, ligand and the base. (b) Hydroxyl region of the acceptor.

NMR Experiment: Nitrogalactal **1a** was mixed with either the base (caesium carbonate), the catalyst and ligand or catalyst, ligand and base at 1:1 stoichiometric ratio. The ^{1}H NMR was recorded at 25 ^{0}C in CD_2Cl_2.

(a)

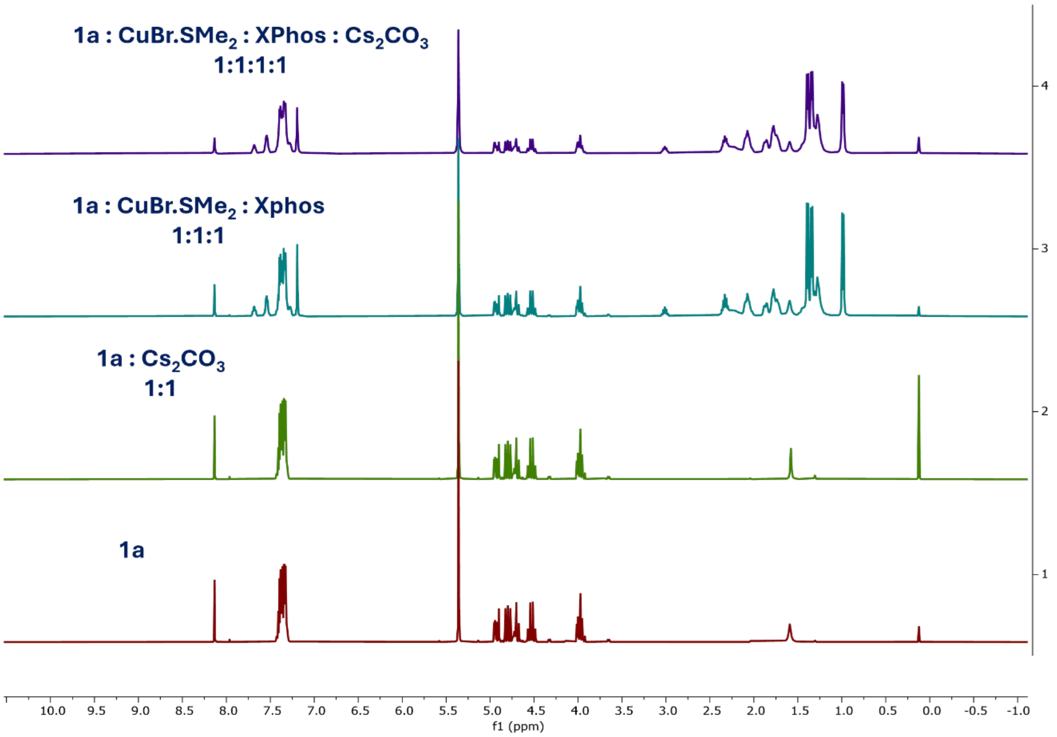

(b)

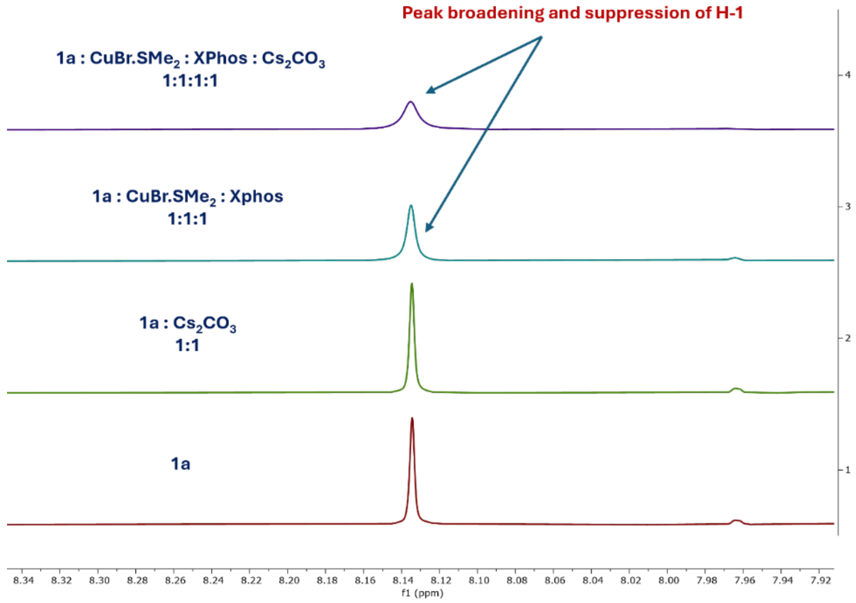

(c)

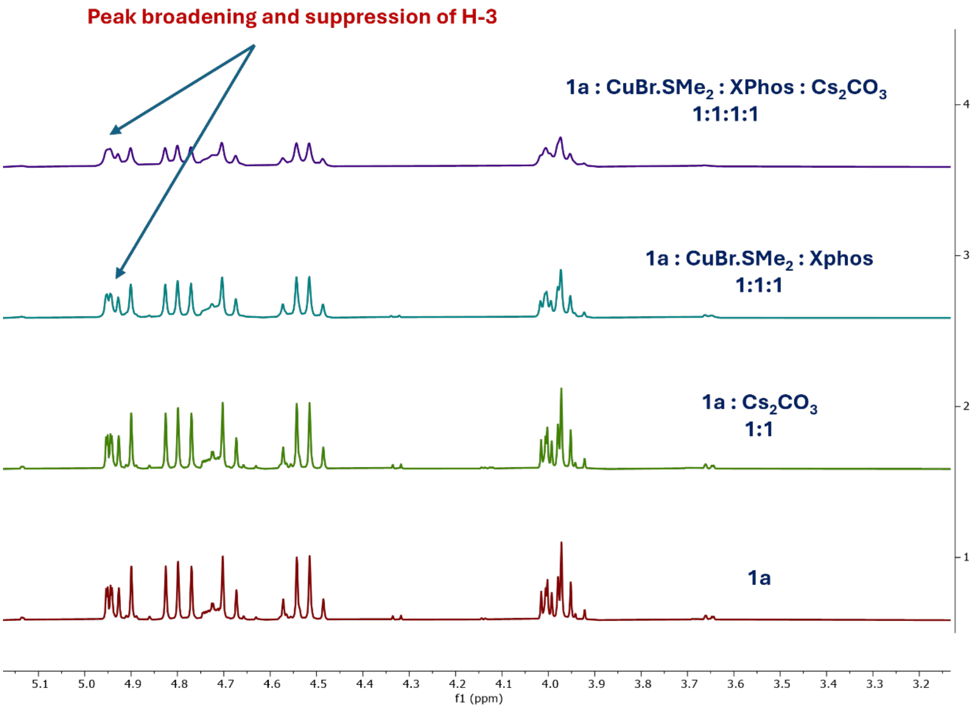

Figure S2. (a) ^{1}H NMR study with nitro glycal donor **1a** and catalyst, ligand and the base. (b) H-1 proton region of the donor. (c) H-3 proton region of the donor.

KIE NMR study

Primary Kinetic Isotope Measurement:

Competition reaction between nitrogalactal **1a** (46 mg, 0.1 mmol) and MeOH (6 μL, 0.2 mmol) and MeOD (6 μL, 0.2 mmol) in the presence of $CuBr.SMe_2$ (2 mg, 0.01 mmol), XPhos (5 mg, 0.01 mmol) and Cs_2CO_3 (8 mg, 0.04 mmol) in DCM-d2 under the general glycosylation procedure was carried out. The ratio of compound **3r** and **3s** was determined by ^{1}H NMR.

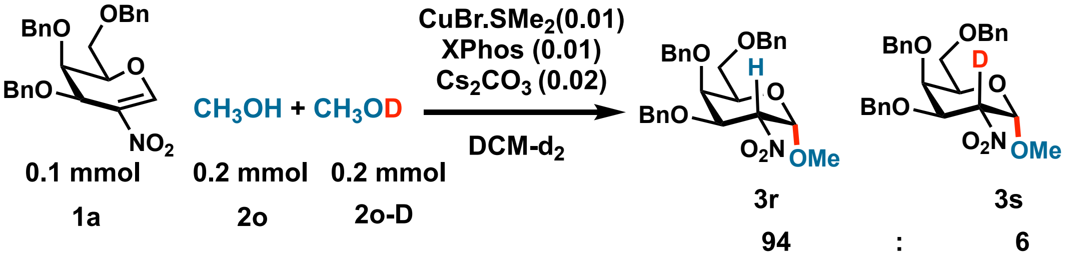

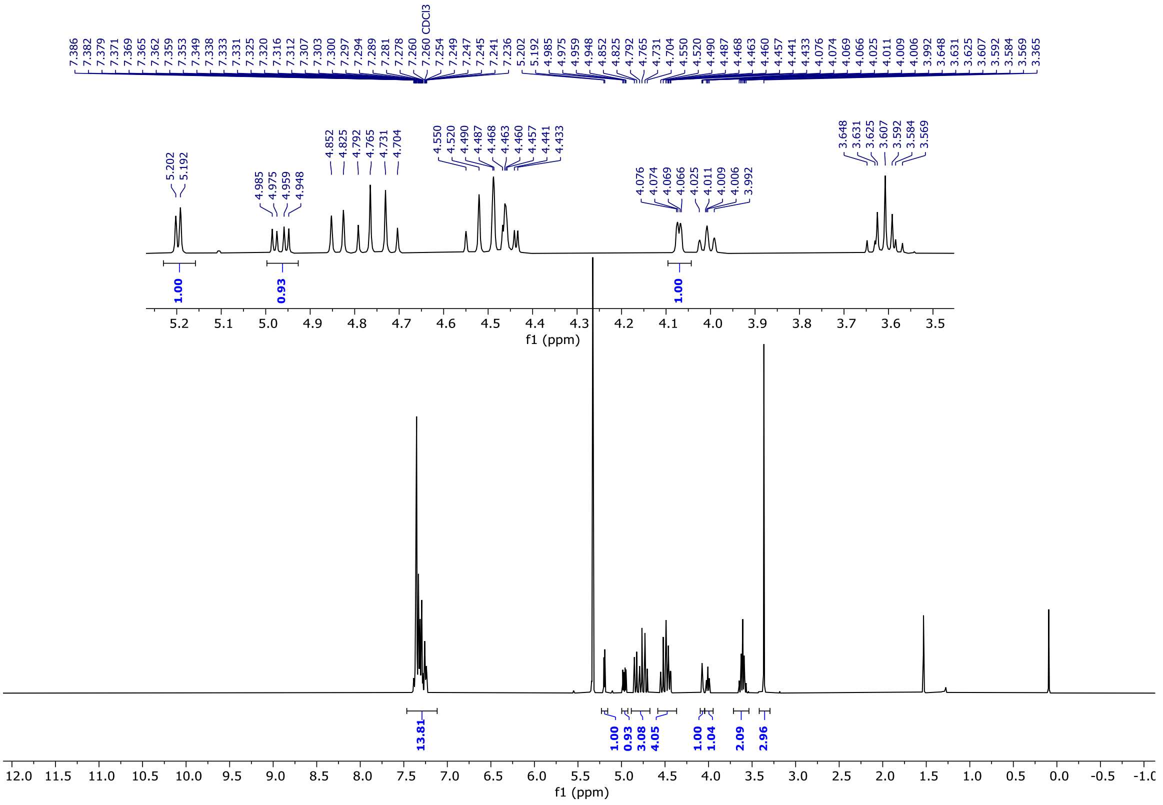

Figure S3. ^{1}H NMR spectra of competition experiments between CH_3OD and CH_3OH.

Secondary Kinetic Isotope Effect

Competitive reaction with nitrogalactal **1a** (46 mg, 0.1 mmol) and MeOH (6 μL, 0.2 mmol) and CD_3OH (6 μL, 0.2 mmol) with CuBr.SMe_2 (2 mg, 0.01 mmol), XPhos (5 mg, 0.01 mmol), Cs_2CO_3 (8 mg, 0.04 mmol) in DCM-d2 following our general glycosylation procedure. The ratio of compound **3r** and **3t** was determined by ^{1}H NMR.

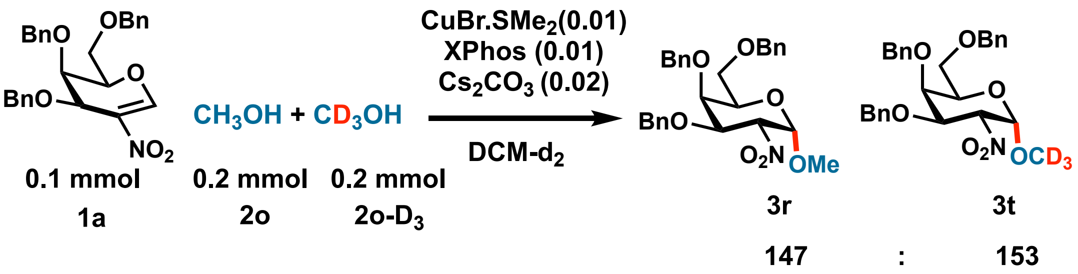

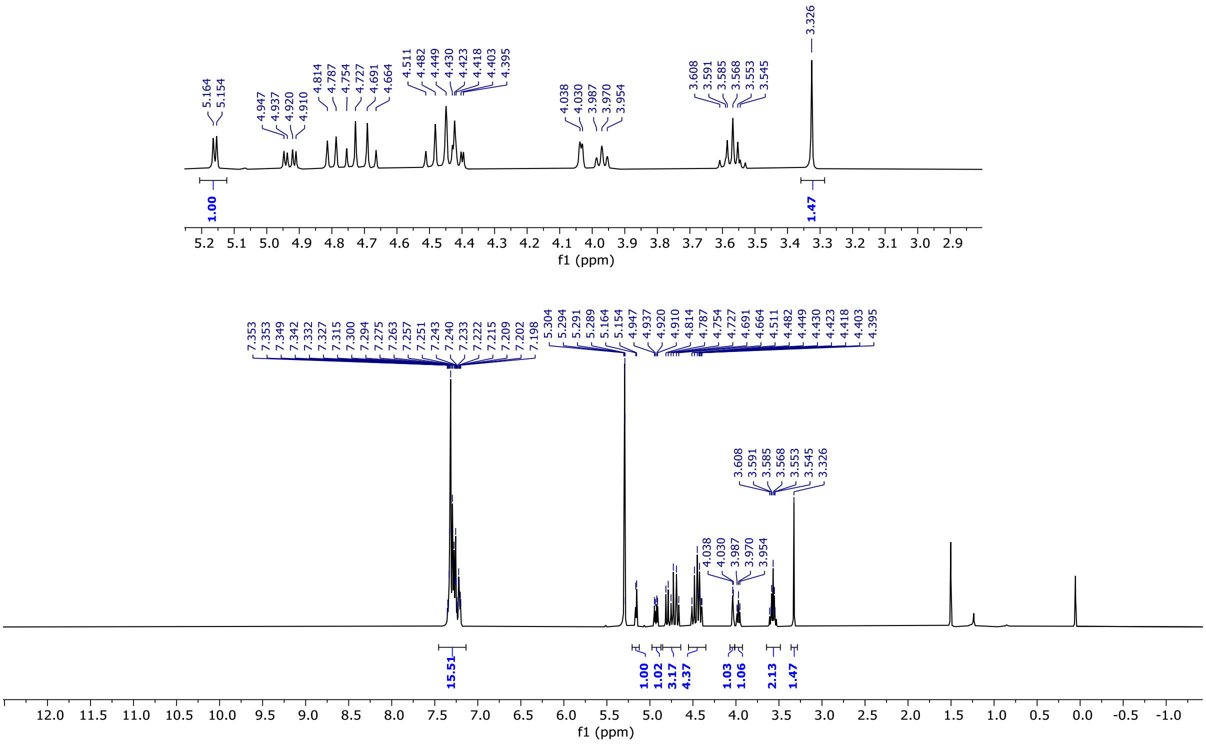

Figure S4. ^{1}H NMR spectra of competition experimnets between CD_3OH and CH_3OH.

Computational Details

Calculations used the standard B3LYP density functional[14,15-18] as implemented in Gaussian 16,[19] along with the 6-31G(d) basis set[20, 21] on all atoms, with only the five spherical harmonic components of the polarization functions, apart from copper, where the MDF10 version of the Stuttgart-Dresden relativistic ECP basis set[22, 23] was used. Grimme's D3 dispersion correction[24] and the polarizable conductor calculation model (CPCM)[25, 26] of solvation, with dichloromethane as the solvent, were also included. Geometries were fully optimised, unless where distance constraints were used to generate starting geometries for transition state searches, as described below. All stationary points were confirmed by calculation of vibrational frequencies at the same level of theory, allowing the determination of Gibbs energies.

Prior computational study of related systems, albeit with a P_4-*t*-Bu superbase rather than the copper catalyst considered here have been reported in the study by Pal et al.[1] There, the computational model was simplified by replacing some of the benzyl protecting groups with methyl, to reduce computational costs. Our initial mechanistic postulate was focussed on steric clashes, such that we decided to retain the full set of benzyl

groups in the present work. Compared to their computational model, we also introduced dispersion corrections. Re-optimisation without dispersion led to bidentate coordination of the copper by the NO_2 groups instead of showing Cu-OBn interactions as suggested by the experimental mechanistic studies, suggesting that the well-known problems arising from the neglect of dispersion in the standard B3LYP functional render this approach unsuitable in the present case.

The presence of OBn substituents complicated conformational space substantially and we performed extensive conformational searches, mainly on complexes of type D at the molecular mechanics level, exploring both MMFF[27] in Spartan[28] and PCModel 10,[29] in the latter case using the default MMX forcefield and the GMMX conformational search engine (default settings, running 500 steps, Emin found 10 times, Duplicates found 50, using distance restraints to achieve a sensible geometry around the copper centre). In these conformational searches, neither the nitroglycal rings nor the cyclohexyl groups on XPhos would change conformation and further conformers were generated by exchanging nitroglycals in optimised structures for different conformers, and by bond rotation in the XPhos ligand, often followed by further conformational searches. However, we do not wish to suggest that conformational space has been explored in full. Representative conformers were re-evaluated at the DFT level and our discussion is based on the DFT energies alone. Despite these substantial and costly explorations of conformational space, it is likely that further low energy conformers could be located, such that the results reported will be subject to conformational noise, which we would estimate as around 2-5 kcal mol^{-1}.

Most transition states were located by elongating the C-O distance in intermediates of type D, freezing these at either 1.75 or 1.9 Å, optimising the resulting geometry subject to this distance constraint and then removing the constraint, using the geometry as a starting point for a full transition state optimisation. For intermediates C, we elongated the C-O distances further, usually to more than 2.3 A and optimised the resulting encounter complexes from there.

For both C and D, multiple intermediates were located. We have largely sought to focus on the ones structurally related to the lowest energy transition state geometry found, but in some cases, lower energy conformers of D could be located; where significant, we've added these in Table S2 below. In light of the high computational costs, we did not attempt IRC calculations to verify the reaction pathways.

Computational Results and Discussion

We initially wondered whether the site selectivity may be determined before interactions with the copper center, similar to oxacarbenium ions.[30, 31] To explore this, new geometries of **1a** and **1e** were drawn in half (twist) boat conformations and used as starting points for

conformational searches (Figure S5); these have been denoted as $^{4}H_{5}$ and $^{5}H_{4}$, as shown in Fig. S5 below. The lowest energy conformers found at the MMFF level in Spartan were then fully optimised at our standard DFT level. For **1a**, the energy difference between the possible conformations **1a_4H5** and **1a_5H4** is small and does not provide conclusive evidence that there is a preferred conformer which locks the direction of nucleophilic attack. In contrast, **1e_5H4** is 9.6 kcal mol^{-1} lower in energy than **1e_4H5**. This is a more significant difference, and suggests that **1e** is much more likely to be found in the **1e_5H4** conformation. We speculated that this may predispose the Michael addition towards β-selectivity, as **1e_5H4** is conformationally more similar to the structure of the sugar ring found in intermediate D after β-addition. This ring conformation may also allow anomeric stabilisation of the benzyl group after β-addition. However, for intermediates C, these energy differences largely disappear, and the $^{4}H_{5}$ conformation becomes energetically competitive, with the corresponding transition states close in energy.

We note that changing to a bigger basis set or including dispersion here changes the magnitude, but not the sign of the energy preference.

Table S1. Summary of results for **1a** and **1e** (potential and Gibbs energies relative to minimum, kcal mol^{-1})

Conformer	Δ*E* (kcal mol^{-1})	Δ*G* (kcal mol^{-1})
**1a_4H5**	0.9	1.7
**1a_5H4**	0.0	0.0
**1e_4H5**	9.6	7.4
**1e_5H4**	0.0	0.0

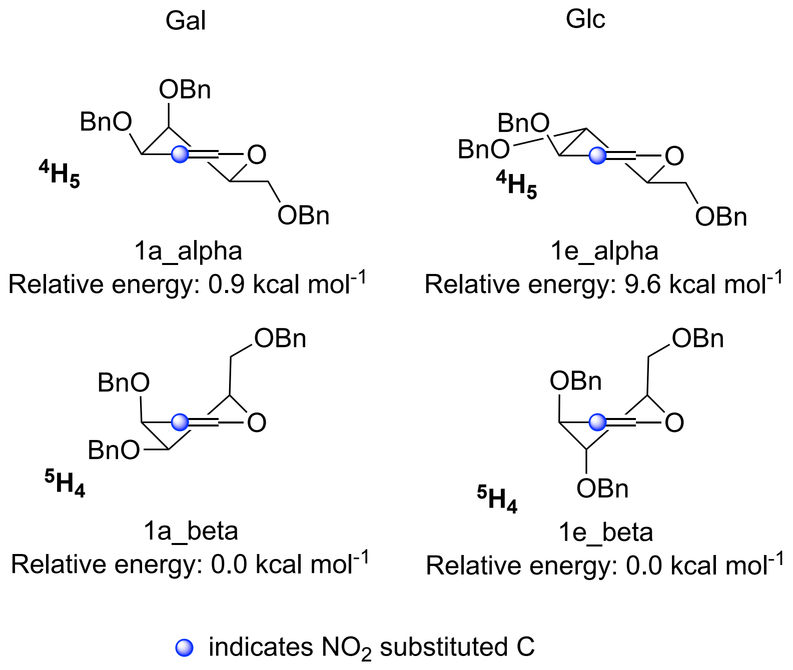

Figure S5: The four structures investigated to determine if the observed selectivities for Michael addition to **1a** and **1e** are influenced by the preferred conformation of the starting material. Note: 1a/1e_alpha refers to the conformation that favours alpha nucleophile approach, whilst 1a/1e_beta annotation is use for the beta or equatorial approach. For clarity, the nitro group on the C2 position has not been drawn, but this C is indicated by a blue sphere.

While we found it reasonably straightforward to locate optimised geometries for intermediate D, we initially struggled with transition state optimisations and locating isomers C. In case this is of interest to readers, we were eventually successful using constraints to freeze elongated C-OBn distances to mimic the approach of the nucleophile, followed by transition state searches from the resulting geometries. The energies calculated suggest that these transition states are quite low in energy relative to both C and D, helping to explain why they were difficult to find. Suitable encounter complexes for C were located by elongating those distances further while ensuring that there were reasonably close Cu-OBn distances. These geometries were quite varied and are likely stable minima on the potential energy surface because of the extensive dispersive interactions between OBn substituents and the XPhos-Cu-OBn complex, highlighting that conformational noise will need to be considered with care here.

In the manuscript (Table 4), we have included Gibbs energies relative to the experimentally-observed route for C; with the slight caveat that this relies on the geometries located for these encounter complexes to be comparable, this was done to relate clearly to experiment. Here, we present a more extensive set of results, including relative energies for separate glycal and XPhos-Cu-OBn species, with the lowest energy

isomers set as zero (Table S2). As noted throughout, these results are noisy, but show that these reaction pathways are energetically accessible. In addition, where significantly lower examples of intermediate D were connected, we've also included these here. Pal *et al.*[1] used QM/MM calculations to estimate barriers to reaction and found that the β-glycoside was kinetically favoured, but not thermodynamically. Our calculations suggest that in the present case the preference may be both kinetic and thermodynamic.

Table S2. Summary of results relative to lowest energy conformer for separate species. Where a lower energy conformer for D was found, this has also been included. Lowest energies routes are shown in bold. (Potential and Gibbs relative energies, kcal mol^{-1}. Cu-OBn = r1, Cu-ONO = r2, C-OBn = r3)

**Label (starting geometry, selectivity)**	**Δ*E* (kcal mol^{-1})**	**Δ*G* (kcal mol^{-1})**	**Key Distances, Å**
XPhosCuOBn			r1 1.843
**Separate, 1a**			
1a, $^4H_5$ + XPhosCuOBn	0.92	1.71	
1a, $^5H_4$ + XPhosCuOBn	0.00	**0.00**	
**1a, C**			
**$^4H_5$, α**	-23.69	**-5.21**	r1 1.841<br>r2 3.108<br>r3 2.710
$^4H_5$, β	-23.33	-4.83	r1 1.843<br>r2 3.360<br>r3 2.415
$^5H_4$, α	-18.51	-1.07	r1 1.847<br>r2 3.772<br>r3 2.775
**$^5H_4$, β**	-27.19	**-7.96**	r1 1.851<br>r2 3.385<br>r3 2.749
**1a, TS**			
**$^4H_5$, α**	-21.11	**0.72**	r1 1.924<br>r2 2.285<br>r3 2.055
$^4H_5$, β	-22.37	-2.03	r1 1.927<br>r2 2.488<br>r3 1.973
$^5H_4$, α	-14.60	4.19	r1 1.901<br>r2 2.500<br>r3 1.900
**$^5H_4$, β**	-24.47	**-2.22**	r1 1.885<br>r2 3.231<br>r3 1.885
**1a, D**			
**$^4H_5$, α**	-25.52	**-6.07**	r1 2.271<br>r2 1.958<br>r3 1.455
$^4H_5$, β	-32.36	-10.72	r1 3.683<br>r2 1.899<br>r3 1.414
$^5H_4$, α	-20.37	0.86	r1 3.000<br>r2 1.916

			r3 1.408
**$^5H_4$, β**	-32.75	**-12.77**	r1 3.076<br>r2 1.927<br>r3 1.426
Lower **$^5H_4$, β**	-33.84	**-13.23**	r1 3.612<br>r2 1.920<br>r3 1.425<br>(XPhos conf.)
**Separate, 1e**			
1e, $^4H_5$ + XPhos CuOBn	9.62	7.35	
1e, $^5H_4$ + XPhos CuOBn	0.00	**0.00**	
**1e, C**			
$^4H_5$, α	-16.83	1.86	r1 1.836<br>r2 4.152<br>r3 2.450
**$^4H_5$, β**	-25.99	**-6.48**	r1 1.839<br>r2 3.437<br>r3 2.120
**$^5H_4$, α**	-25.14	**-7.39**	r1 1.840<br>r2 4.181<br>r3 2.646
$^5H_4$, β	-25.47	-5.37	r1 1.839<br>r2 3.486<br>r3 2.576
**1e, TS**			
$^4H_5$, α	-15.61	4.63	r1 1.876<br>r2 3.723<br>r3 1.958
**$^4H_5$, β**	-21.69	**0.14**	r1 1.908<br>r2 2.971<br>r3 1.850
**$^5H_4$, α**	-19.28	**0.82**	r1 1.893<br>r2 3.637<br>r3 1.824
$^5H_4$, β	-21.11	0.62	r1 1.908<br>r2 2.971<br>r3 1.850
**1e, D**			
$^4H_5$, α	-24.81	-4.28	r1 2.261<br>r2 2.015<br>r3 1.443
**$^4H_5$, β**	-27.94	**-5.39**	r1 2.317<br>r2 2.004<br>r3 1.460
**$^5H_4$, α**	-25.45	**-5.76**	r1 2.221<br>r2 2.052<br>r3 1.454
$^5H_4$, β	-26.35	-4.43	r1 2.145<br>r2 2.128<br>r3 1.487
Lower **$^4H_5$, β**	-29.60	**-7.61**	r1 3.617<br>r2 1.901<br>r3 1.425
Lower **$^5H_4$, α**	-30.00	**-9.83**	r1 2.448<br>r2 1.991

			r3 1.440<br>(XPhos conf. change)

Tables S3. Calculated potential and Gibbs energies and imaginary frequencies

**Label<br>(starting geometry, selectivity)**	***E* (Hartrees)**	***G* (Hartrees)**	**TS imaginary frequency (cm^{-1})**
XPhosCuOBn	-2172.1478953	-2171.349932	
1a, $^{4}H_{5}$	-1551.130395	-1550.69326	
1a, $^{5}H_{4}$	-1551.1318682	-1550.695987	
**1a, C**			
**$^{4}H_{5}$, α**	-3723.3175138	-3722.054226	
$^{4}H_{5}$, β	-3723.3169426	-3722.053619	
$^{5}H_{4}$, α	-3723.3092672	-3722.047623	
**$^{5}H_{4}$, β**	-3723.3231	-3722.0586	
**1a, TS**			
**$^{4}H_{5}$, α**	-3723.3134107	-3722.044767	-113.0
$^{4}H_{5}$, β	-3723.3154099	-3722.049158	-135.5
$^{5}H_{4}$, α	-3723.3030365	-3722.039235	-216.9
**$^{5}H_{4}$, β**	-3723.3187603	-3722.049463	-234.1
**1a, D**			
**$^{4}H_{5}$, α**	-3723.320439	-3722.055589	
$^{4}H_{5}$, β	-3723.3313318	-3722.062998	
$^{5}H_{4}$, α	-3723.3122320	-3722.044544	
**$^{5}H_{4}$, β**	-3723.3319579	-3722.066266	
Lower **$^{5}H_{4}$, β**	-3723.3336843	-3722.067005	
1e, $^{4}H_{5}$	-1551.122478	-1550.691031	
1e, $^{5}H_{4}$	-1551.137802	-1550.702748	
**1e, C**			
$^{4}H_{5}$, α	-3723.312513	-3722.04972	
**$^{4}H_{5}$, β**	-3723.327115	-3722.063013	
**$^{5}H_{4}$, α**	-3723.325755	-3722.064454	
$^{5}H_{4}$, β	-3723.326285	-3722.061237	
**1e, TS**			
$^{4}H_{5}$, α	-3723.310573	-3722.045304	-147.4
**$^{4}H_{5}$, β**	-3723.32026	-3722.052459	-198.1
**$^{5}H_{4}$, α**	-3723.316424	-3722.051372	-266.7
$^{5}H_{4}$, β	-3723.319339	-3722.051691	-254.3
**1e, D**			
$^{4}H_{5}$, α	-3723.32523	-3722.059500	
**$^{4}H_{5}$, β**	-3723.330221	-3722.061266	
**$^{5}H_{4}$, α**	-3723.326253	-3722.061855	
$^{5}H_{4}$, β	-3723.327687	-3722.059741	
Lower **$^{4}H_{5}$, β**	-3723.332864	-3722.064811	
Lower **$^{5}H_{4}$, α**	-3723.333509	-3722.068348	

The Cartesian coordinates of all optimised geometries have been included in a separate xyz file, suitable for visualisation in Mercury and other readers.

Notes on Experimental vs. Calculated Selectivity (Dispersion)

While close in energy, we did not manage to locate an α-selective transition state for nitrogalactal 1a lower in energy than the corresponding β-selective pathway, which would be in line with the experimentally-observed selectivity. However, these transition states are close (within 3 kcal mol^{-1} in Gibbs energy) and a number of different factors affect the computational model used. The calculations are in better agreement for 1e, but by the same token, we need to concede that there could be a viable α-selective pathway in terms of the calculated Gibbs energies.

One of the potential issues is the exploration of conformational space, which remains incomplete and we note that these calculations are noisy, as well as computationally costly. While we have explored this quite extensively, this cannot be avoided in a system of this size and complexity.

Another possible issue arises from the deficiencies of the implicit modelling of solvation, which will affect the accuracy of entropic predictions to some extent, although the effect is likely to be quite similar for the competing routes, and this may well be another contribution to the computational noise.

As noted above, we also wondered whether the selectivity may be determined before direct interactions with the copper complex. As discussed, this does not seem to be the case, but there might be other parts of the proposed mechanism where such a differentiation could arise and by the time the copper becomes involved, the selectivity may have been pre-set.

Our final consideration was the impact of the dispersion correction. Our sense here is that the complexes investigated are sterically demanding and that the aromatic groups of the benzyl-substituents, coupled with comparatively easy rotations around the C-O bonds, present multiple possible conformations where intra-molecular dispersive interactions (particularly end-on C-H...π, π...π) could contribute to the conformational preferences observed. In solution, some of this would be countered by solute-solvent interactions, but these effects will not be captured fully by the present computational model.

Geometry optimisation of intermediates D at the B3LYP level gave structures where the only interactions between the glycal and CuXPhos were via the nitro group, showing no interactions at all with the reacting site. In contrast, B3LYP-D3 showed closer contacts as captured in Table S2 above (i.e. relatively short r1 and r2 distances). To get a better sense of the impact of dispersion on the energies, we performed single point calculations on the B3LYP-D3-optimised geometries without these corrections. While the barriers

relative to C increased, the picture remains mixed – in some examples, the α-selective pathway seems to become relatively more favourable, but this is not consistently the case. From inspecting various geometries, it is possible that the β-selective pathways benefit more, energetically, from this contribution.

References

(1) Pal, K. B.; Guo, A.; Das, M.; Báti, G.; Liu, X.-W. Superbase-Catalyzed Stereo- and Regioselective Glycosylation with 2-Nitroglycals: Facile Access to 2-Amino-2-deoxy-O-glycosides. *ACS Catal.* **2020**, *10* (12), 6707-6715. DOI: 10.1021/acscatal.0c00753.

(2) Dharuman, S.; Gupta, P.; Kancharla, P. K.; Vankar, Y. D. Synthesis of 2-nitroglycals from glycals using the tetrabutylammonium nitrate-trifluoroacetic anhydride-triethylamine reagent system and base-catalyzed Ferrier rearrangement of acetylated 2-nitroglycals. *J. Org. Chem.* **2013**, *78* (17), 8442-8450. DOI: 10.1021/jo401165y.

(3) Kirschning, A. Oxidation of Fully Protected Glycals by Hypervalent Iodine Reagents. *J. Org. Chem.* **2002**, *60* (5), 1228-1232. DOI: 10.1021/jo00110a028.

(4) Sagar, R.; Park, J.; Koh, M.; Park, S. B. Diastereoselective synthesis of polycyclic acetal-fused pyrano[3,2-c]pyran-5(2H)-one derivatives. *J. Org. Chem.* **2009**, *74* (5), 2171-2174. DOI: 10.1021/jo8023889.

(5) Medina, S.; Harper, M. J.; Balmond, E. I.; Miranda, S.; Crisenza, G. E.; Coe, D. M.; McGarrigle, E. M.; Galan, M. C. Stereoselective Glycosylation of 2-Nitrogalactals Catalyzed by a Bifunctional Organocatalyst. *Org. Lett.* **2016**, *18* (17), 4222-4225. DOI: 10.1021/acs.orglett.6b01962.

(6) Holzapfel, C. W.; Marais, C. F.; Van Dyk, M. S. 2-Nitroglycals Preparation and Nucleophilic Addition Reactions. *Synthetic Commun.* **1988**, *18* (1), 97-114. DOI: 10.1080/00397918808057825.

(7) Medina, S.; Harper, M. J.; Balmond, E. I.; Miranda, S.; Crisenza, G. E. M.; Coe, D. M.; McGarrigle, E. M.; Galan, M. C. Stereoselective Glycosylation of 2-Nitrogalactals Catalyzed.by a Bifunctional Organocatalyst. *Org. Lett.* **2016**, *18* (17), 4222-4225.

(8) Barroca, N.; Schmidt, R. R. 2-Nitro Thioglycoside Donors: Versatile Precursors of β-d-Glycosides of Aminosugars. *Org. Lett.* **2004**, *6* (10), 1551-1554. DOI: 10.1021/ol049729t.

(9) Hu, Y.-L.; Gou, B.-B.; Wang, J.; Zhao, M.; Liu, J.-L.; Chen, J.; Zhou, L. N-Heterocyclic Carbene Catalyzed Stereoselective Synthesis of 2-Nitro-thiogalactosides. *Synthesis* **2019**, *51* (18), 3451-3461. DOI: 10.1055/s-0039-1690099.

(10) Dharuman, S.; Gupta, P.; Kancharla, P. K.; Vankar, Y. D. Synthesis of 2-Nitroglycals from Glycals Using the Tetrabutylammonium Nitrate–Trifluoroacetic Anhydride–Triethylamine Reagent System and Base-Catalyzed Ferrier Rearrangement of Acetylated 2-Nitroglycals. *J. Org. Chem.* **2013**, *78* (17), 8442-8450. DOI: 10.1021/jo401165y.

(11) Liu, J.-L.; Zhang, Y.-T.; Liu, H.-F.; Zhou, L.; Chen, J. N-Heterocyclic Carbene Catalyzed Stereoselective Glycosylation of 2-Nitrogalactals. *Org. Lett.* **2017**, *19* (19), 5272-5275. DOI: 10.1021/acs.orglett.7b02543.

(12) Lin, Z.-P.; Wong, F. F.; Chen, Y.-B.; Lin, Y.-C.; Kimura, M.; Kaneko, K.; Takayama, H.; Wu, J. B.; Lin, C.-H.; Lin, H.-C. Synthesis of α-2-deoxy-ulosides by Michael addition of hex-1-en-3-ones. *Tetrahedron* **2013**, *69* (11), 2494-2500. DOI: 10.1016/j.tet.2012.12.085.

(13) Lin, P.-C.; Lin, Z.-P.; Chen, P.-Y.; Hsieh, M.-T.; Lin, H.-C. New metal-free one-pot synthesis of α-2-deoxy-Ulosides by microwave-assisted double Michael Addition of β-enamino ketones. *Carbohydr. Res.* **2023**, *523*, 108712. DOI: 10.1016/j.carres.2022.108712.
(14) J. C. Slater, Quantum Theory of Molecules and Solids, Vol. 4: The Self-Consistent Field for Molecules and Solids, McGraw-Hill, New York, 1974
(15) Becke, A. D. Density-functional exchange-energy approximation with correct asymptotic behavior. *Phys. Rev. A* **1988**, *38* (6), 3098-3100. DOI: 10.1103/PhysRevA.38.3098.
(16) Becke, A. D. Density-functional thermochemistry. III. The role of exact exchange. *J. Chem. Phys.* **1993**, *98* (7), 5648-5652. DOI: 10.1063/1.464913
(17) Vosko, S. H.; Wilk, L.; Nusair, M. Accurate spin-dependent electron liquid correlation energies for local spin density calculations: a critical analysis. *Can. J. Phys.* **1980**, *58* (8), 1200-1211. DOI: 10.1139/p80-159.
(18) Lee, C.; Yang, W.; Parr, R. G. Development of the Colle-Salvetti correlation-energy formula into a functional of the electron density. *Phys. Rev. B* **1988**, *37* (2), 785-789. DOI: 10.1103/PhysRevB.37.785.
(19) Gaussian 16, Revision C.01,M. J. Frisch, G. W. Trucks, H. B. Schlegel, G. E. Scuseria, M. A. Robb, J. R. Cheeseman, G. Scalmani, V. Barone, G. A. Petersson, H. Nakatsuji, X. Li, M. Caricato, A. V. Marenich, J. Bloino, B. G. Janesko, R. Gomperts, B. Mennucci, H. P. Hratchian, J. V. Ortiz, A. F. Izmaylov, J. L. Sonnenberg, D. Williams-Young, F. Ding, F. Lipparini, F. Egidi, J. Goings, B. Peng, A. Petrone, T. Henderson, D. Ranasinghe, V. G. Zakrzewski, J. Gao, N. Rega, G. Zheng, W. Liang, M. Hada, M. Ehara, K. Toyota, R. Fukuda, J. Hasegawa, M. Ishida, T. Nakajima, Y. Honda, O. Kitao, H. Nakai, T. Vreven, K. Throssell, J. A. Montgomery, Jr., J. E. Peralta, F. Ogliaro, M. J. Bearpark, J. J. Heyd, E. N. Brothers, K. N. Kudin, V. N. Staroverov, T. A. Keith, R. Kobayashi, J. Normand, K. Raghavachari, A. P. Rendell, J. C. Burant, S. S. Iyengar, J. Tomasi, M. Cossi, J. M. Millam, M. Klene, C. Adamo, R. Cammi, J. W. Ochterski, R. L. Martin, K. Morokuma, O. Farkas, J. B. Foresman, and D. J. Fox, Gaussian, Inc., Wallingford CT, 2019.

(20) Hariharan, P. C.; Pople, J. A. The influence of polarization functions on molecular orbital hydrogenation energies. *Theor. chim. acta* **1973**, *28* (3), 213-222. DOI: 10.1007/BF00533485.
(21) Hehre, W. J.; Ditchfield, R.; Pople, J. A. Self—Consistent Molecular Orbital Methods. XII. Further Extensions of Gaussian—Type Basis Sets for Use in Molecular Orbital Studies of Organic Molecules. *J. Chem. Phys.* **1972**, *56* (5), 2257-2261. DOI: 10.1063/1.1677527.
(22) Stoll, H.; Fuentealba, P.; Schwerdtfeger, P.; Flad, J.; Szentpály, L. v.; Preuss, H. Cu and Ag as one-valence-electron atoms: CI results and quadrupole corrections for Cu2, Ag2, CuH, and AgH. *J. Chem. Phys.* **1984**, *81* (6), 2732-2736. DOI: 10.1063/1.447992 (acccessed 11/15/2024).
(23) Dolg, M.; Wedig, U.; Stoll, H.; Preuss, H. Energy-adjusted ab initio pseudopotentials for the first row transition elements. *J. Chem. Phys.* **1987**, *86* (2), 866-872. DOI: 10.1063/1.452288 (acccessed 11/15/2024).
(24) Grimme, S.; Antony, J.; Ehrlich, S.; Krieg, H. A consistent and accurate ab initio parametrization of density functional dispersion correction (DFT-D) for the 94 elements H-Pu. *J. Chem. Phys.* **2010**, *132* (15). DOI: 10.1063/1.3382344 (acccessed 11/15/2024).

(25) Barone, V.; Cossi, M. Quantum Calculation of Molecular Energies and Energy Gradients in Solution by a Conductor Solvent Model. *J. Chem. Phys. A* **1998**, *102* (11), 1995-2001. DOI: 10.1021/jp9716997.
(26) Cossi, M.; Rega, N.; Scalmani, G.; Barone, V. Energies, structures, and electronic properties of molecules in solution with the C-PCM solvation model. *J. Comput. Chem.* **2003**, *24* (6), 669-681. DOI: 10.1002/jcc.10189.
(27) Halgren, T. A. Merck molecular force field. I. Basis, form, scope, parameterization, and performance of MMFF94. *Journal of Computational Chemistry* **1996**, *17* (5-6), 490-519. DOI: 10.1002/(SICI)1096-987X(199604)17:5/6
(28) Spartan'24, Version 1.1.0, Mar 11 2024
(29) Gilbert, K. PCModel, Serena Software; Bloomington, IN
(30) Balmond, E. I.; Benito-Alifonso, D.; Coe, D. M.; Alder, R. W.; McGarrigle, E. M.; Galan, M. C. A 3,4-trans-Fused Cyclic Protecting Group Facilitates alpha-Selective Catalytic Synthesis of 2-Deoxyglycosides. *Angew. Chem. Int. Ed.* **2014**, *53* (31), 8190-8194. DOI: 10.1002/anie.201403543.
(31) Ayala, L.; Lucero, C. G.; Romero, J. A. C.; Tabacco, S. A.; Woerpel, K. A. Stereochemistry of nucleophilic substitution reactions depending upon substituent: Evidence for electrostatic stabilization of pseudoaxial conformers of oxocarbenium ions by heteroatom substituents. *J. Am. Chem. Soc.* **2003**, *125* (50), 15521-15528.

NMR Spectra

^1H NMR (400 MHz, $CDCl_3$)

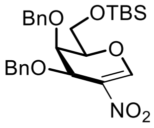

1b

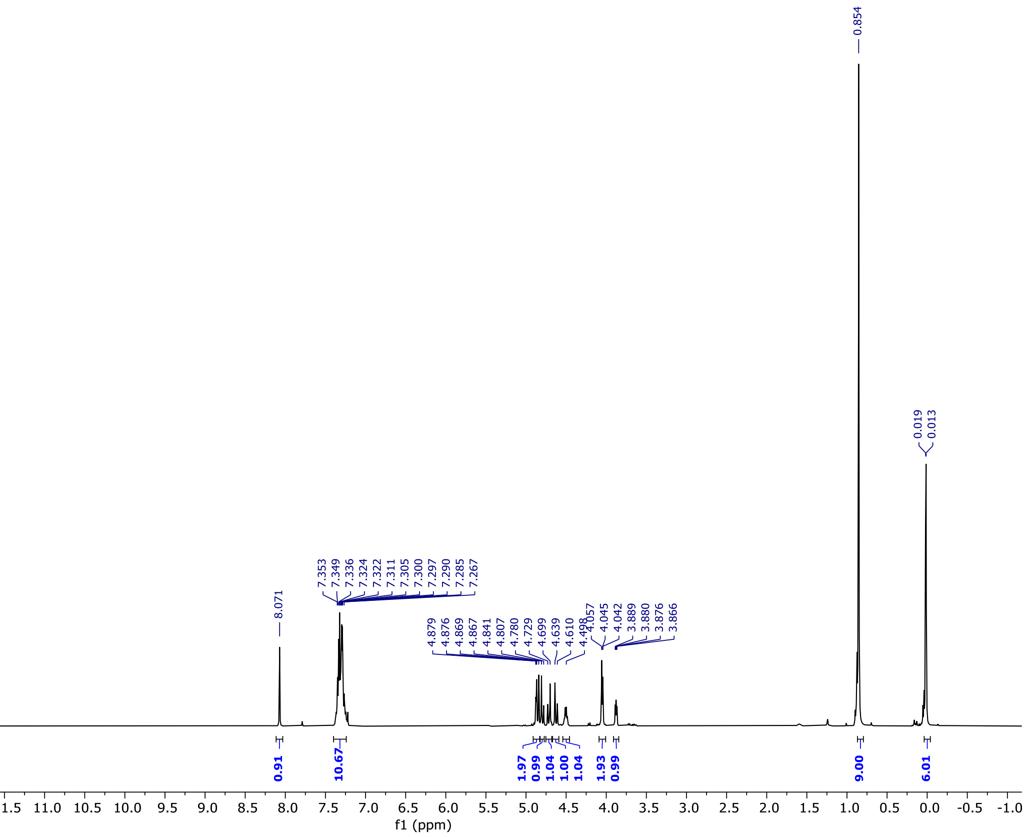

$^{13}C\{^1H\}$ NMR ($CDCl_3$, 101 MHz)

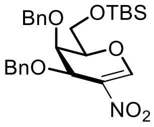

1b

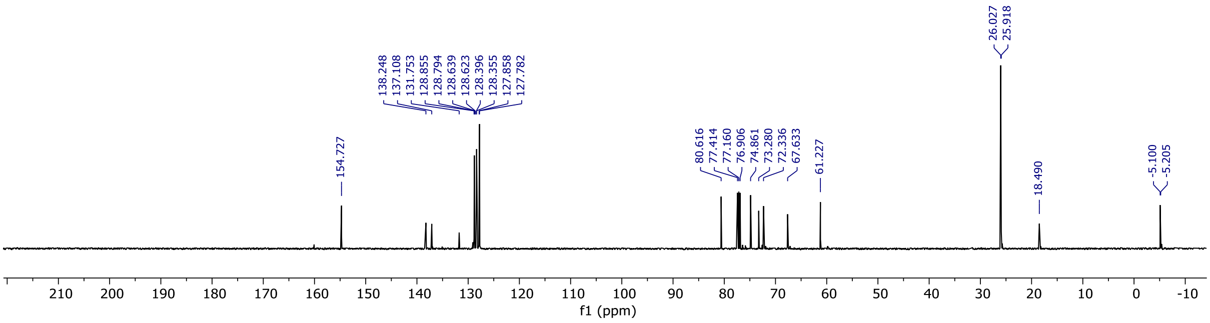

^{1}H NMR ($CDCl_3$, 400 MHz)

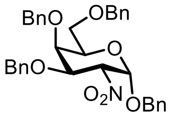

3a

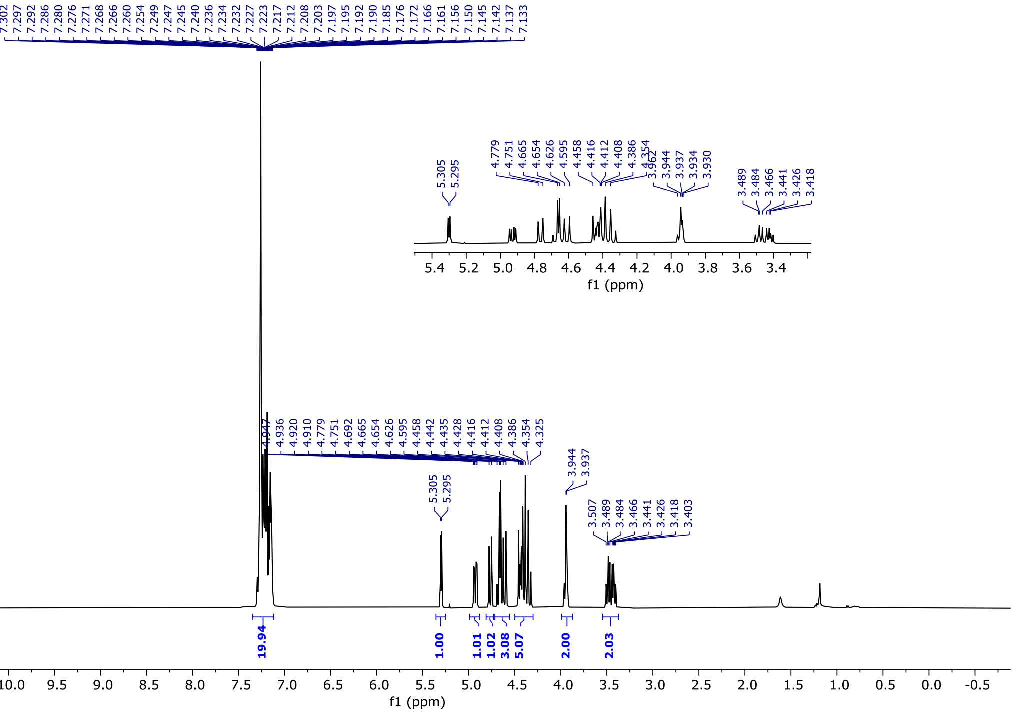

$^{13}C\{^{1}H\}$ NMR ($CDCl_3$, 101 MHz)

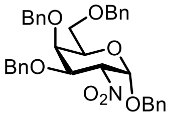

3a

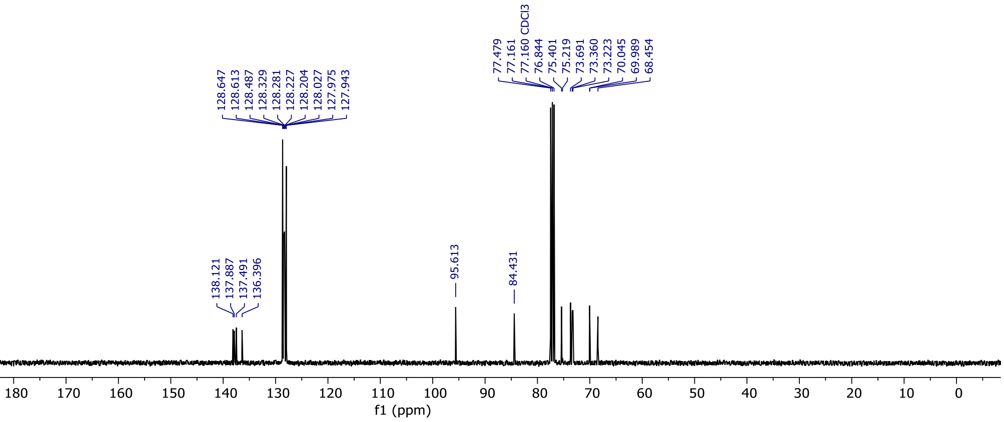

COSY NMR ($CDCl_3$, 400 MHz)

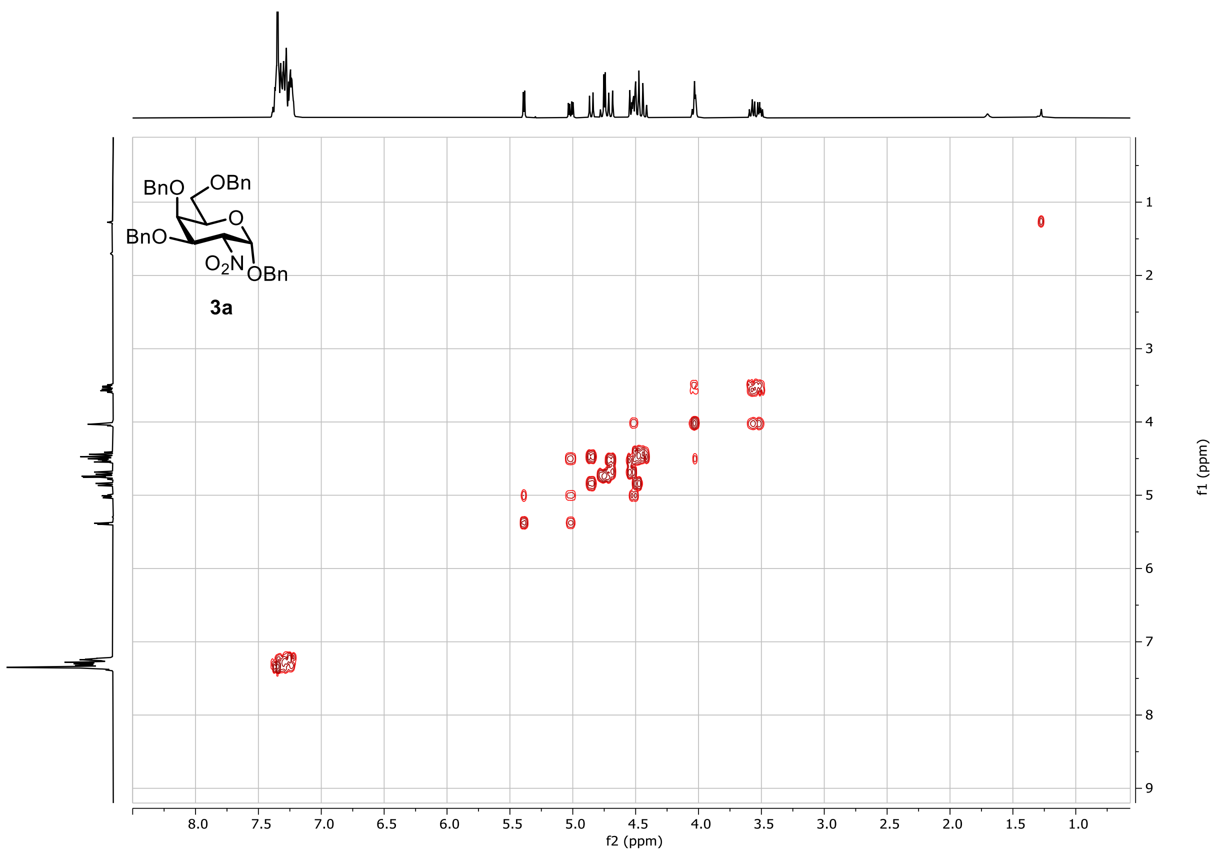

HSQC NMR ($CDCl_3$, 400 MHz)

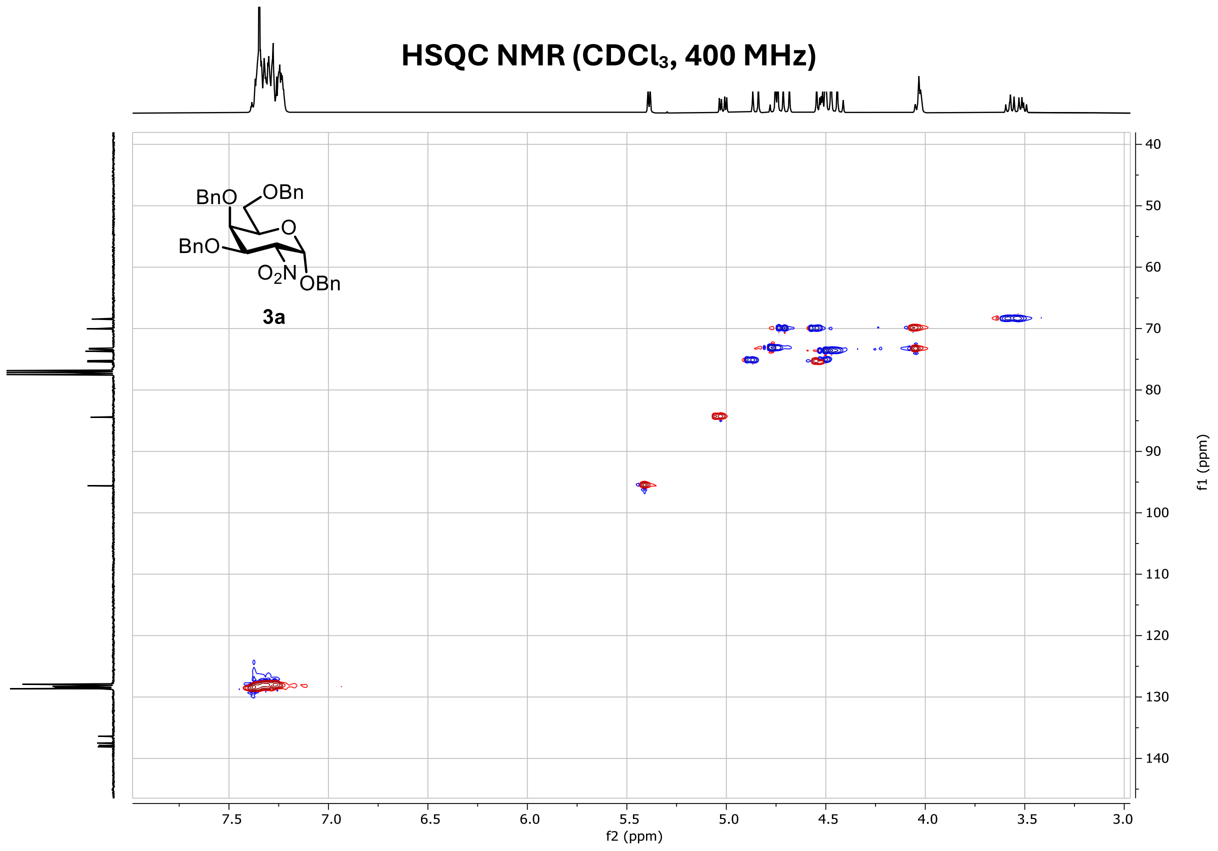

^{1}H NMR (CDCl$_3$, 400 MHz)

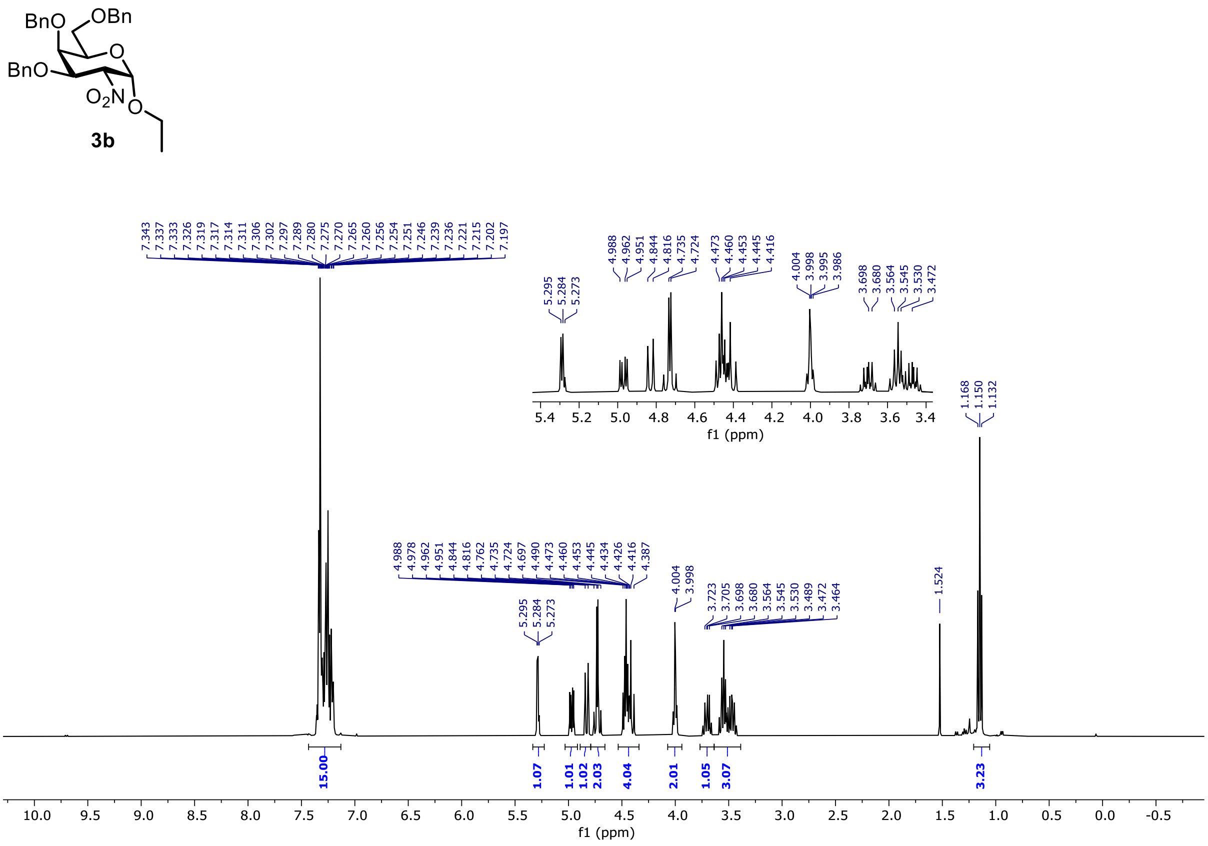

^{13}C{^{1}H} NMR (CDCl$_3$, 101 MHz)

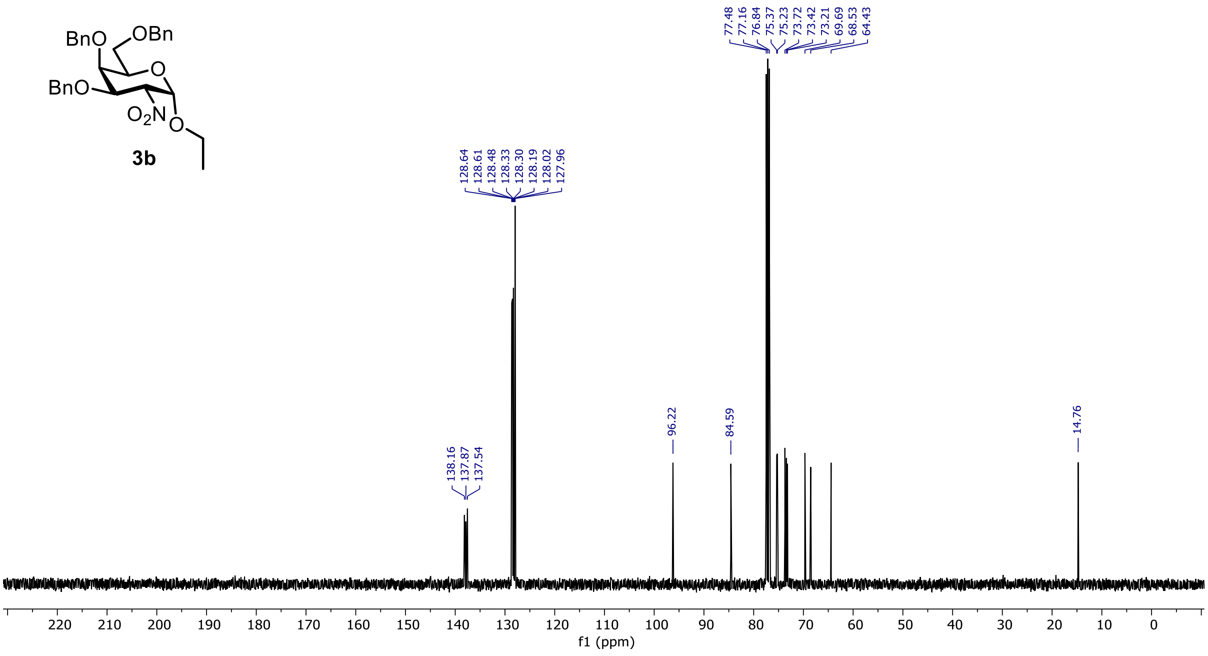

COSY NMR ($CDCl_3$, 400 MHz)

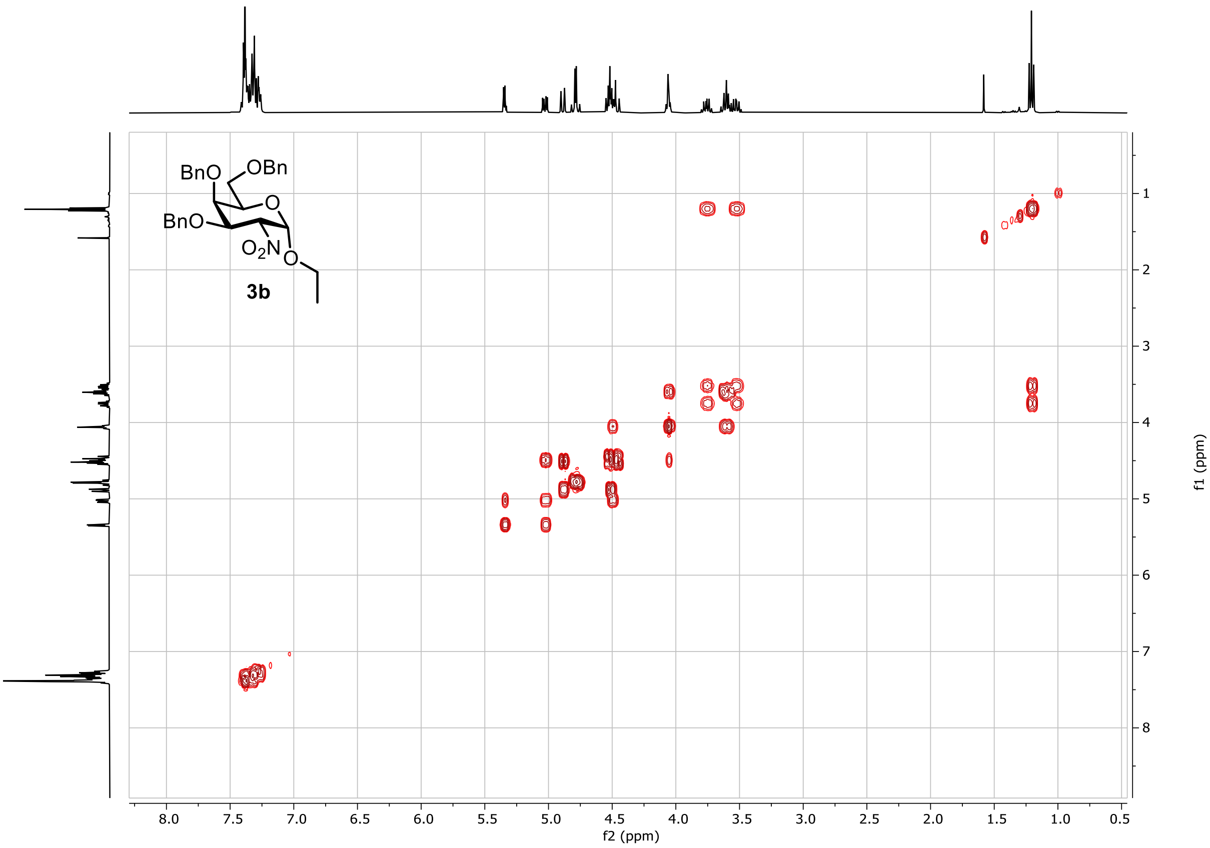

HSQC NMR ($CDCl_3$, 400 MHz)

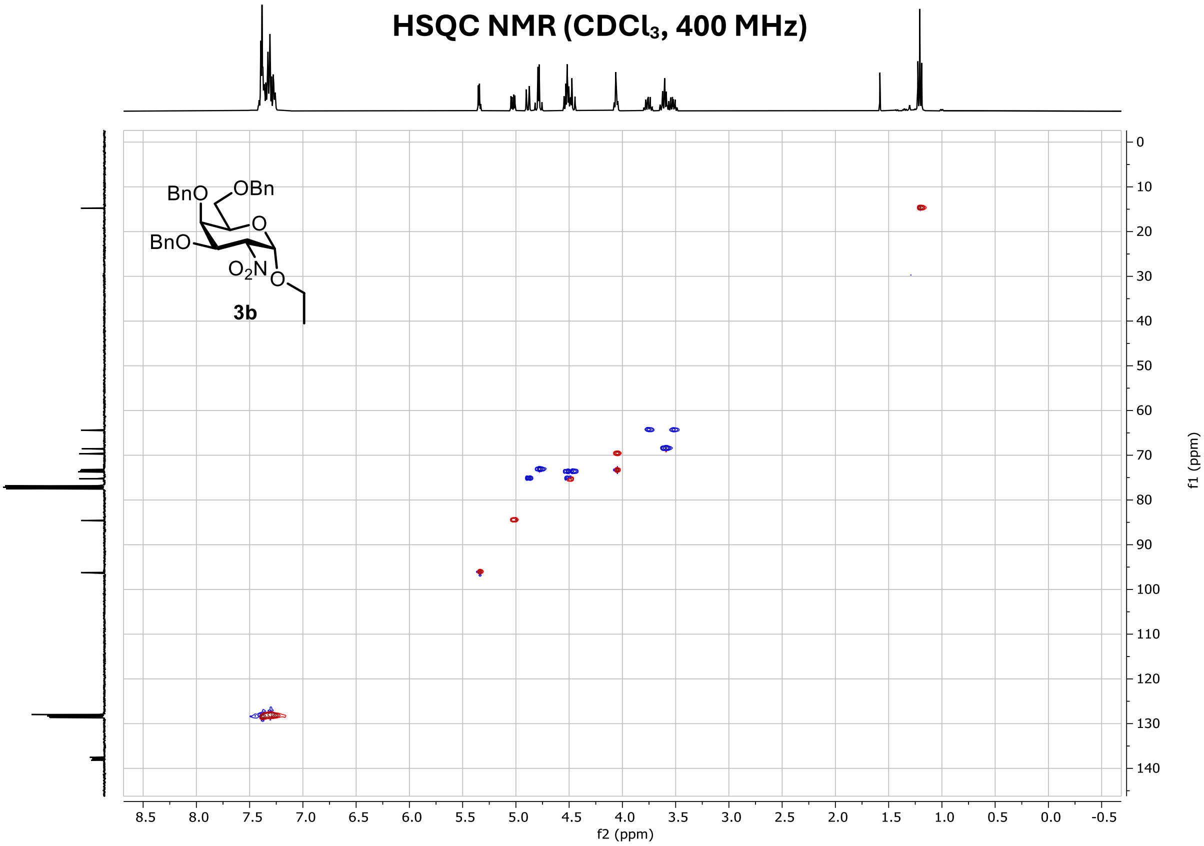

^{1}H NMR ($CDCl_3$, 600 MHz)

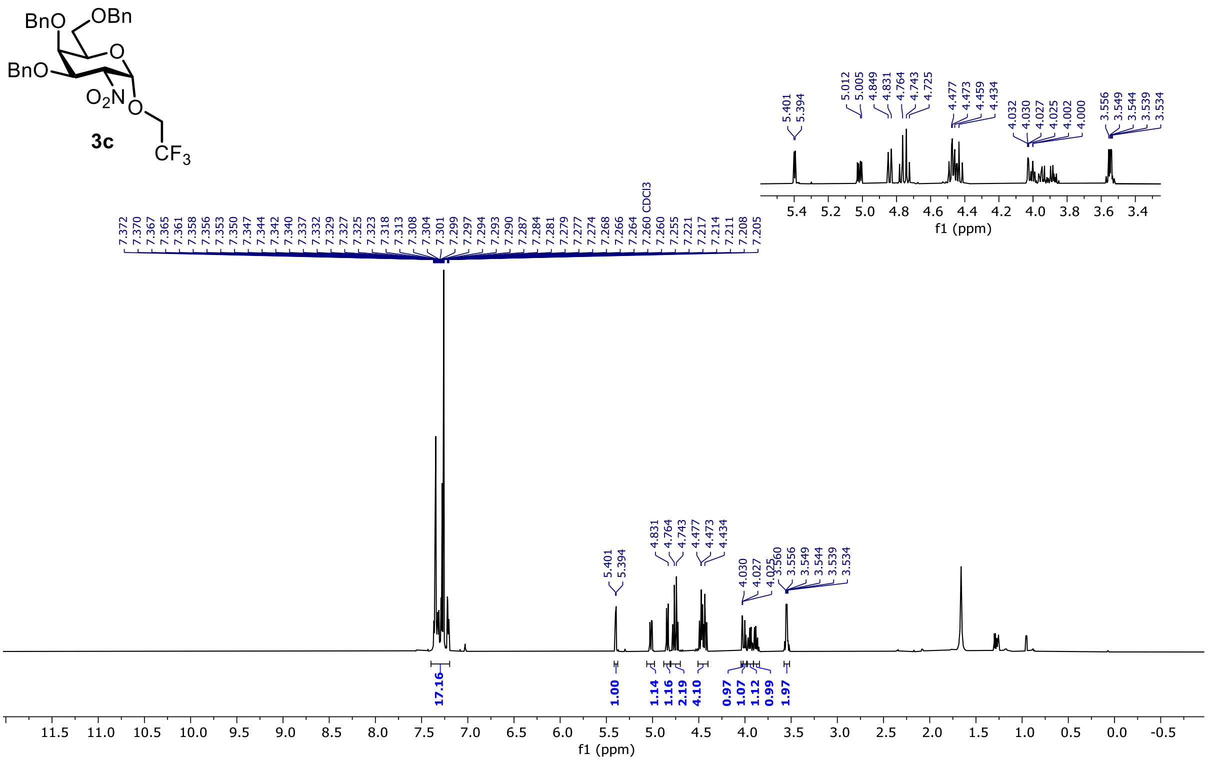

$^{13}C\{^{1}H\}$ NMR ($CDCl_3$, 151 MHz)

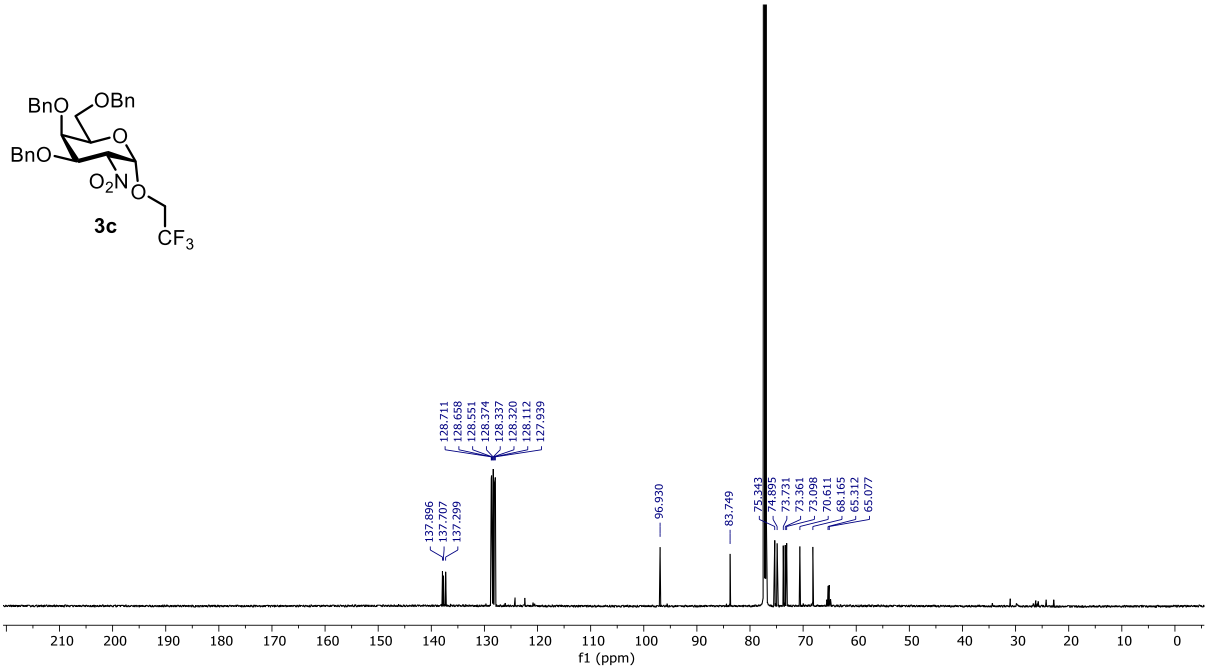

COSY NMR (CDCl$_3$, 600 MHz)

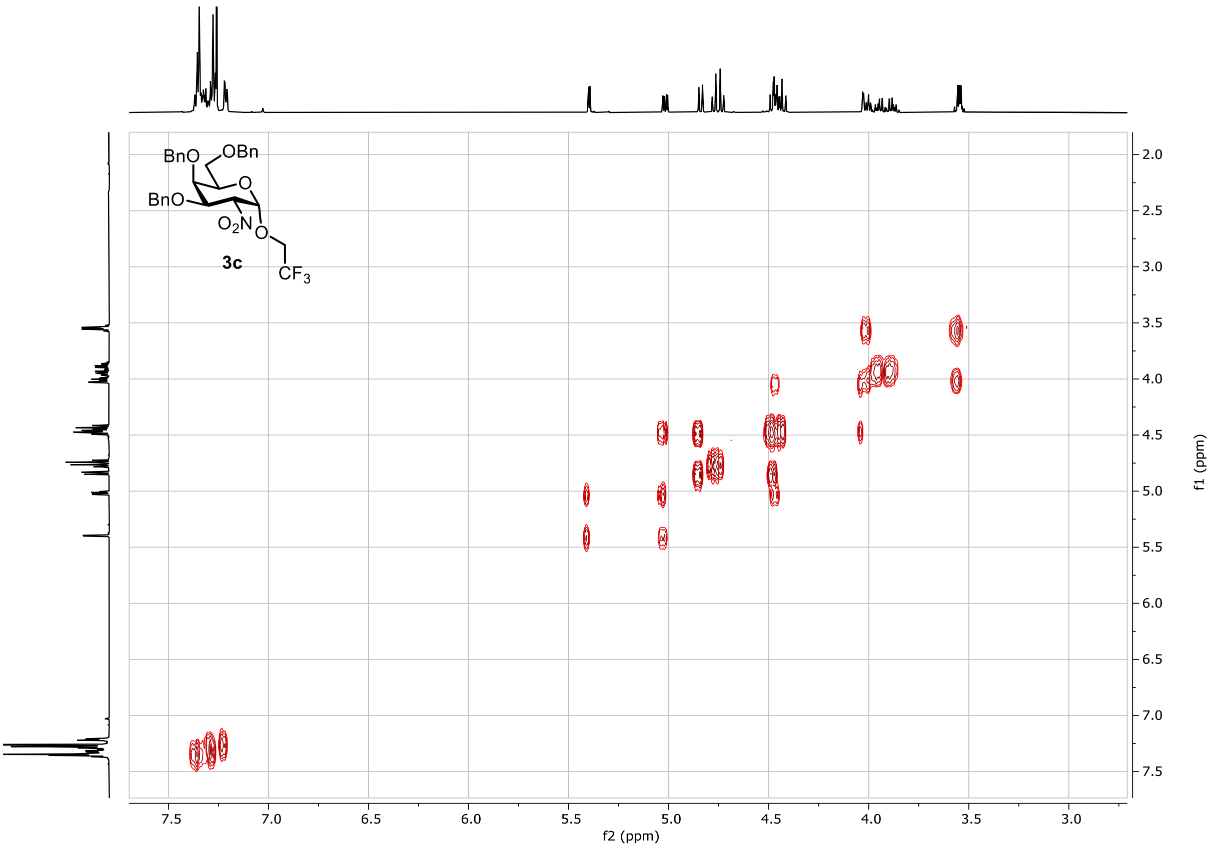

HSQC NMR (CDCl$_3$, 600 MHz)

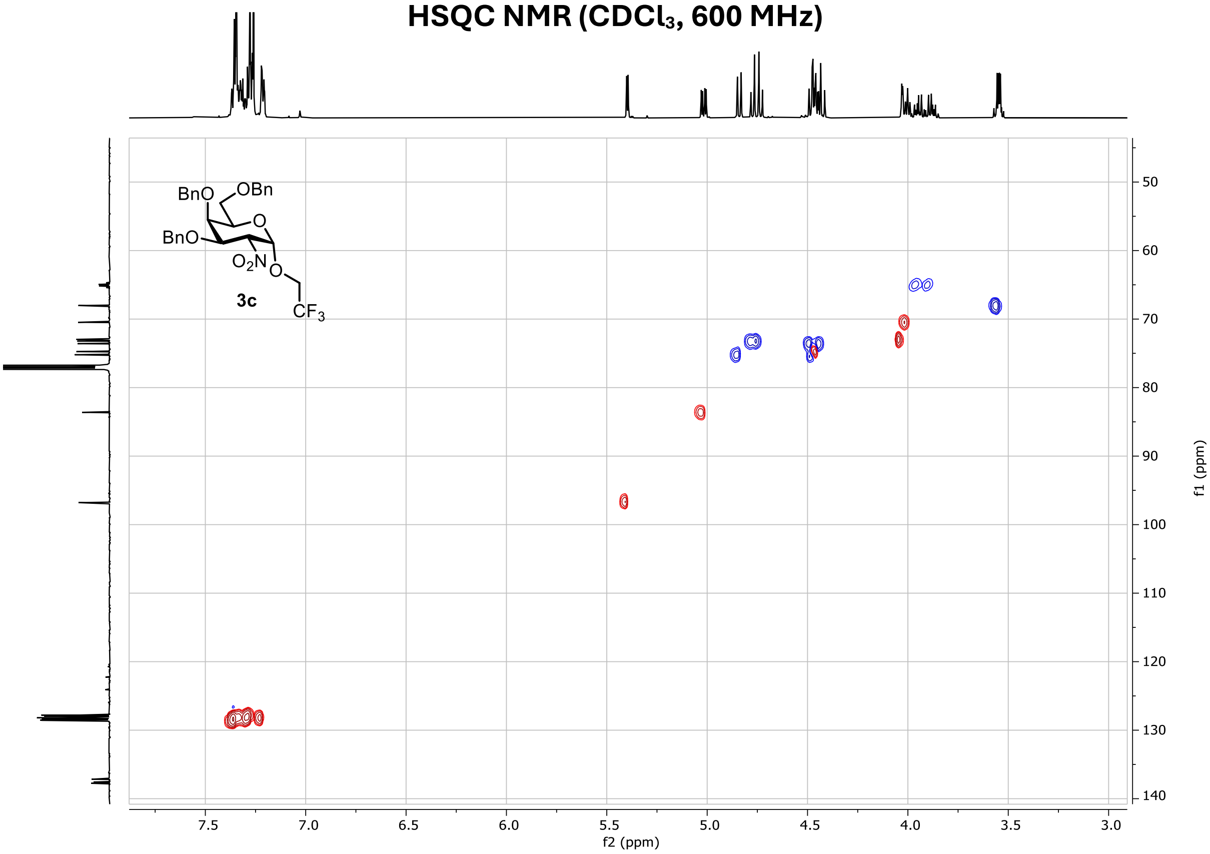

^{1}H NMR ($CDCl_3$, 400 MHz)

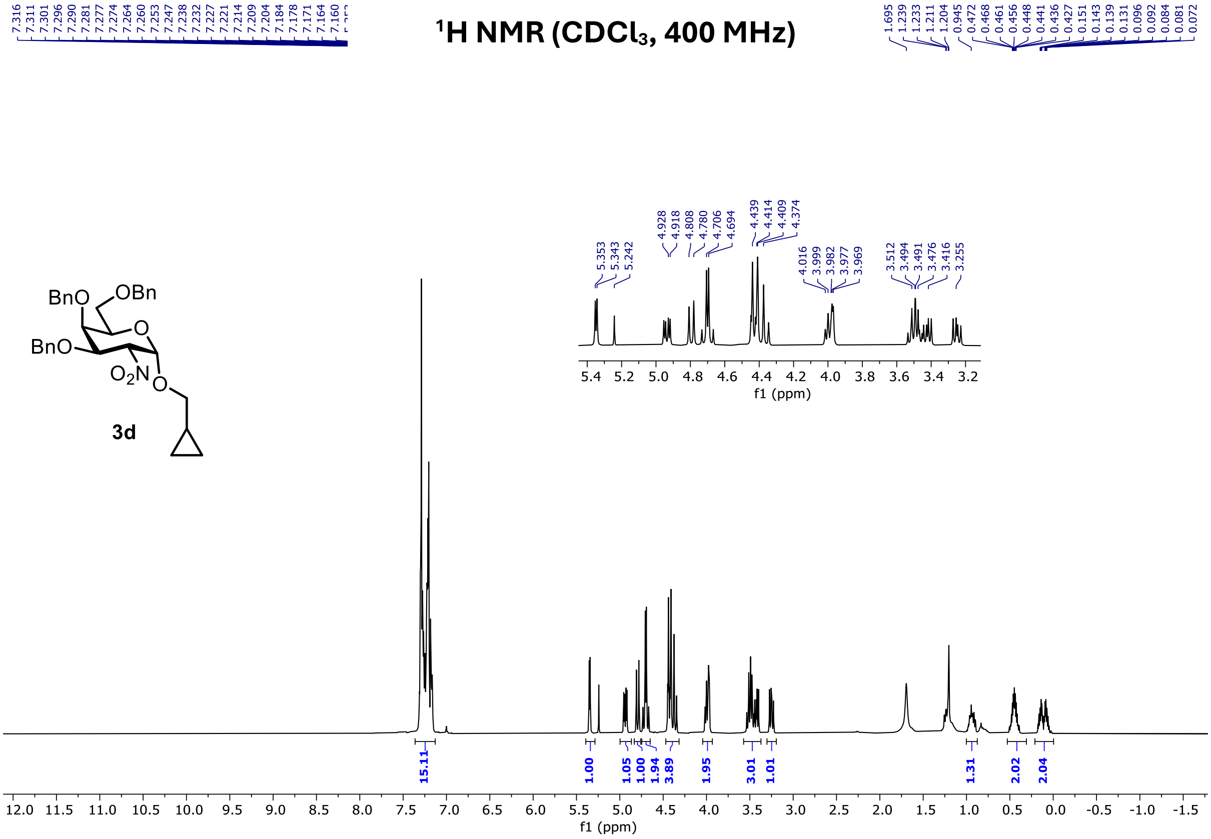

$^{13}C\{^{1}H\}$ NMR ($CDCl_3$, 101 MHz)

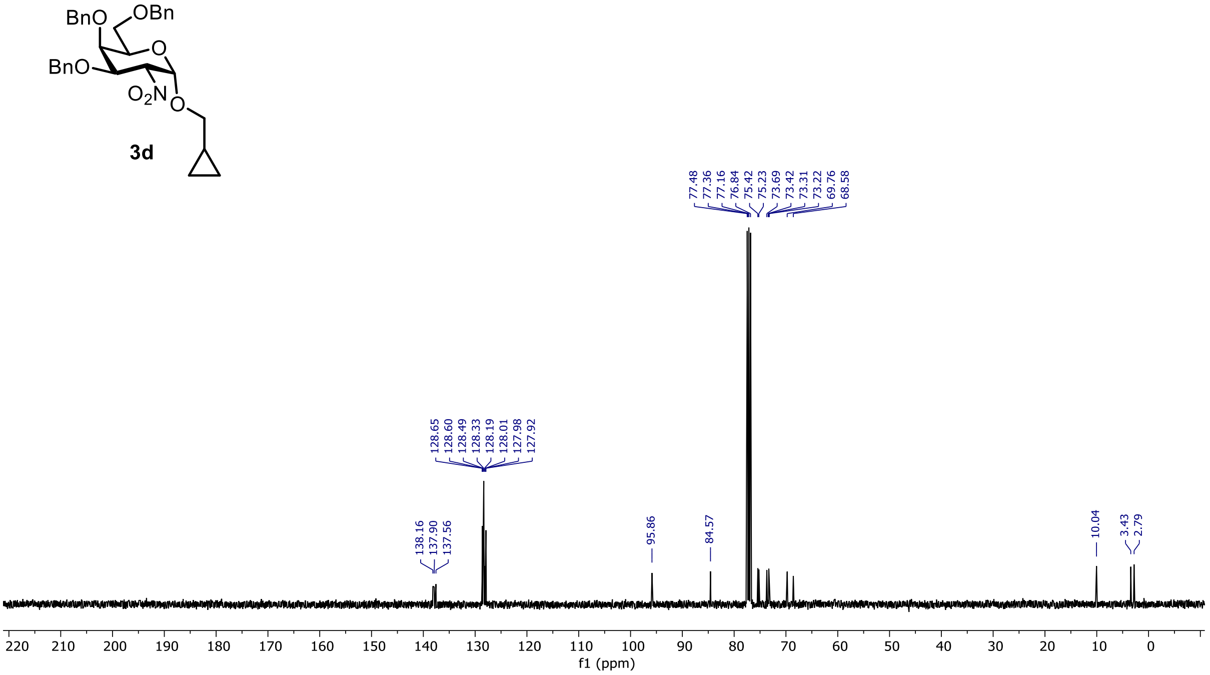

COSY NMR ($CDCl_3$, 400 MHz)

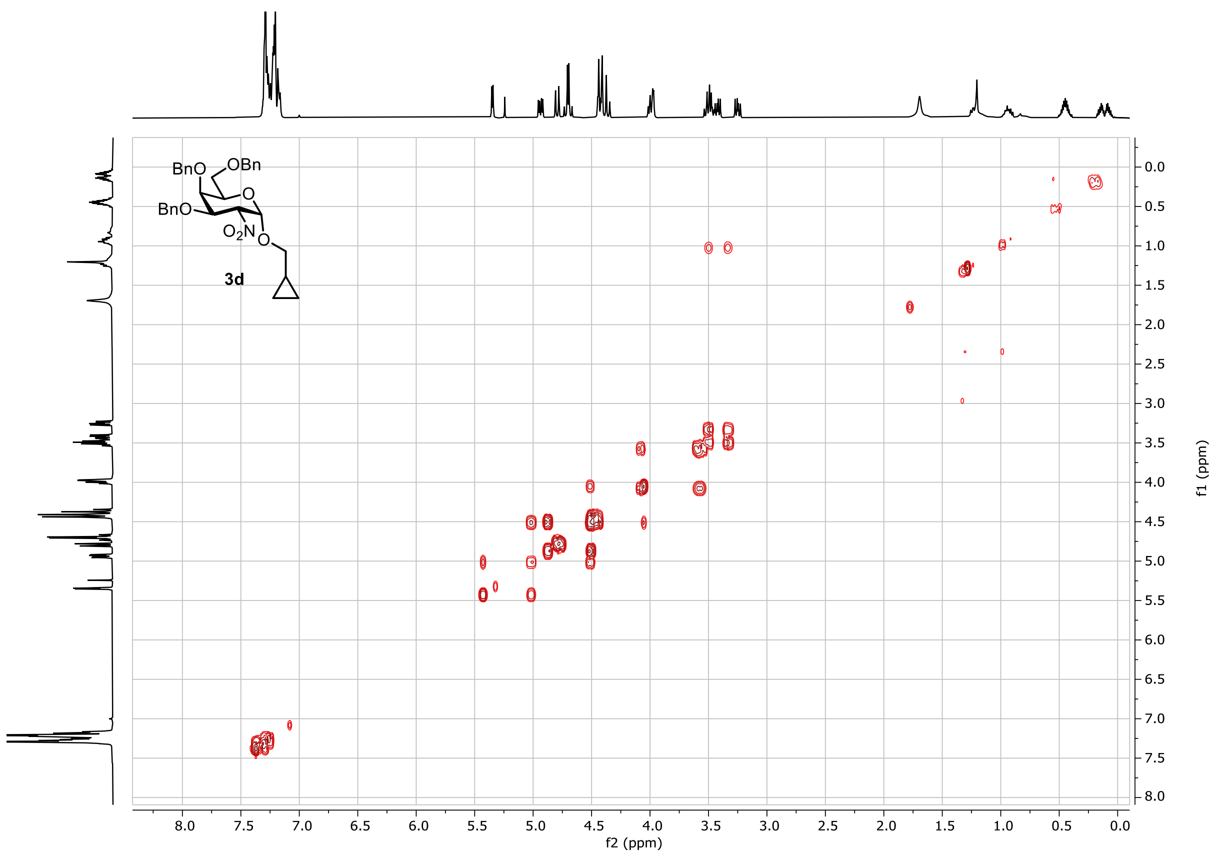

HSQC NMR ($CDCl_3$, 400 MHz)

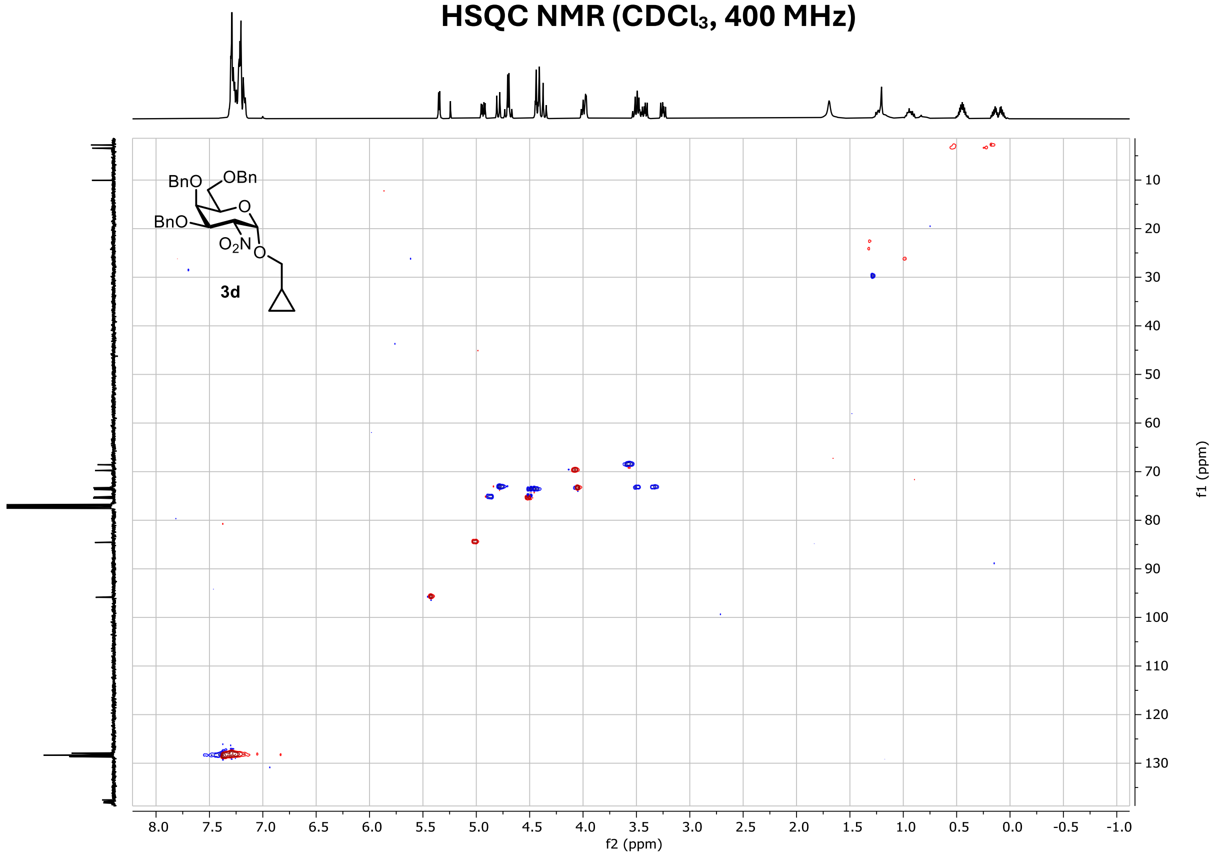

^{1}H NMR ($CDCl_3$, 600 MHz)

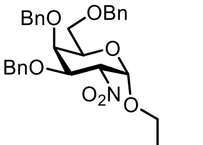

3e

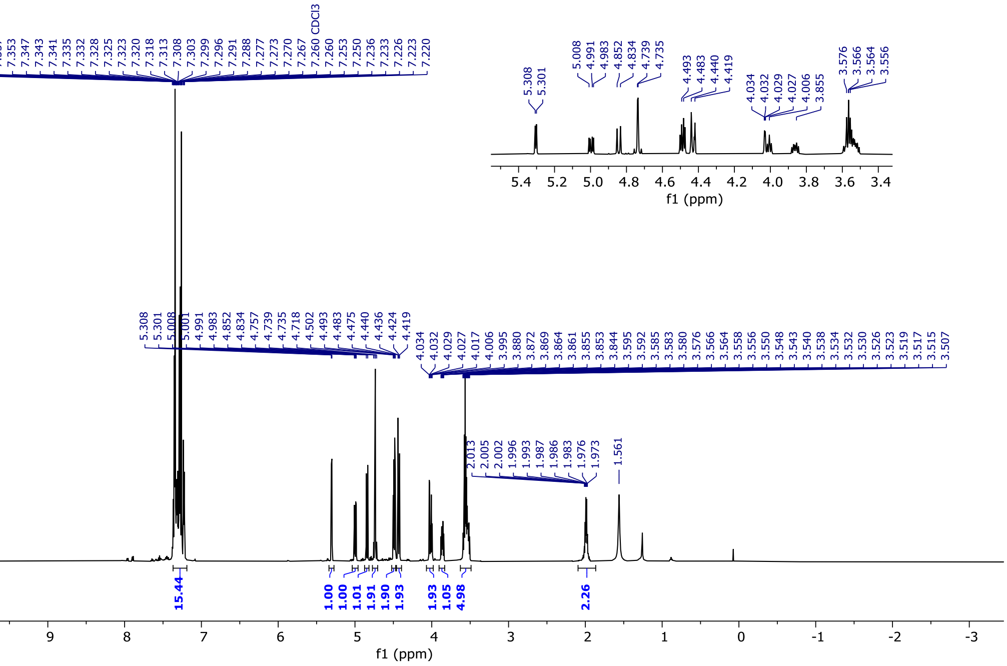

^{13}C{^{1}H} NMR ($CDCl_3$, 151 MHz)

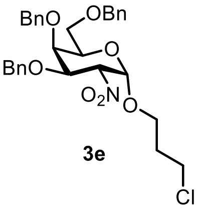

3e

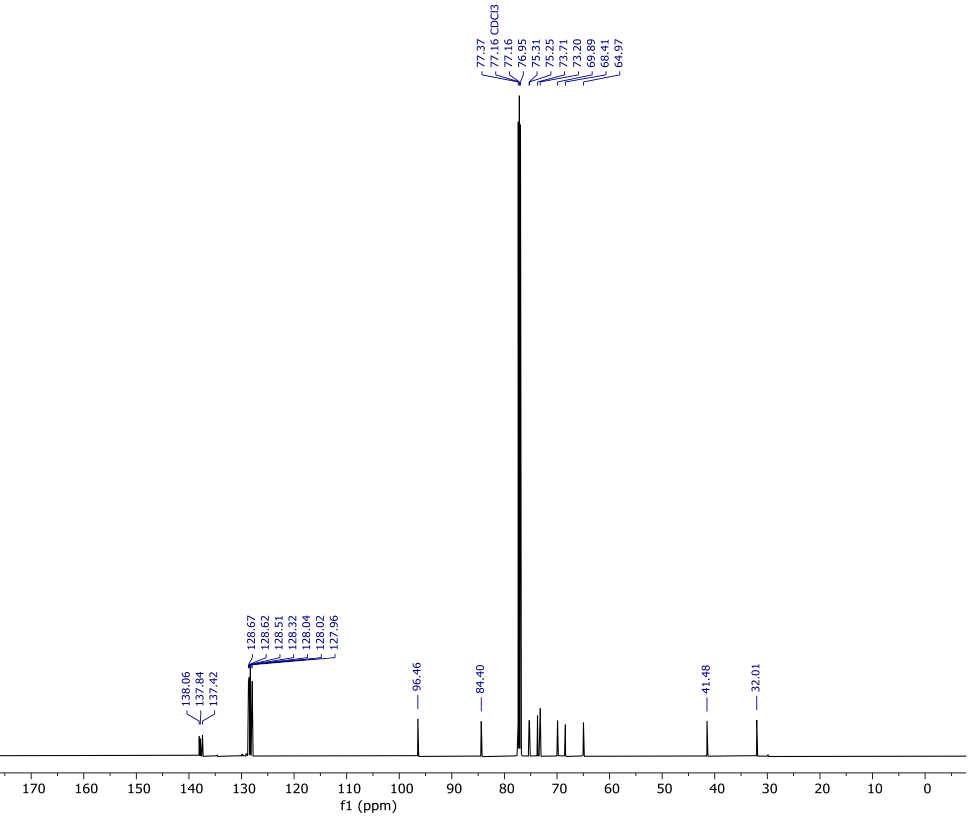

COSY NMR ($CDCl_3$, 600 MHz)

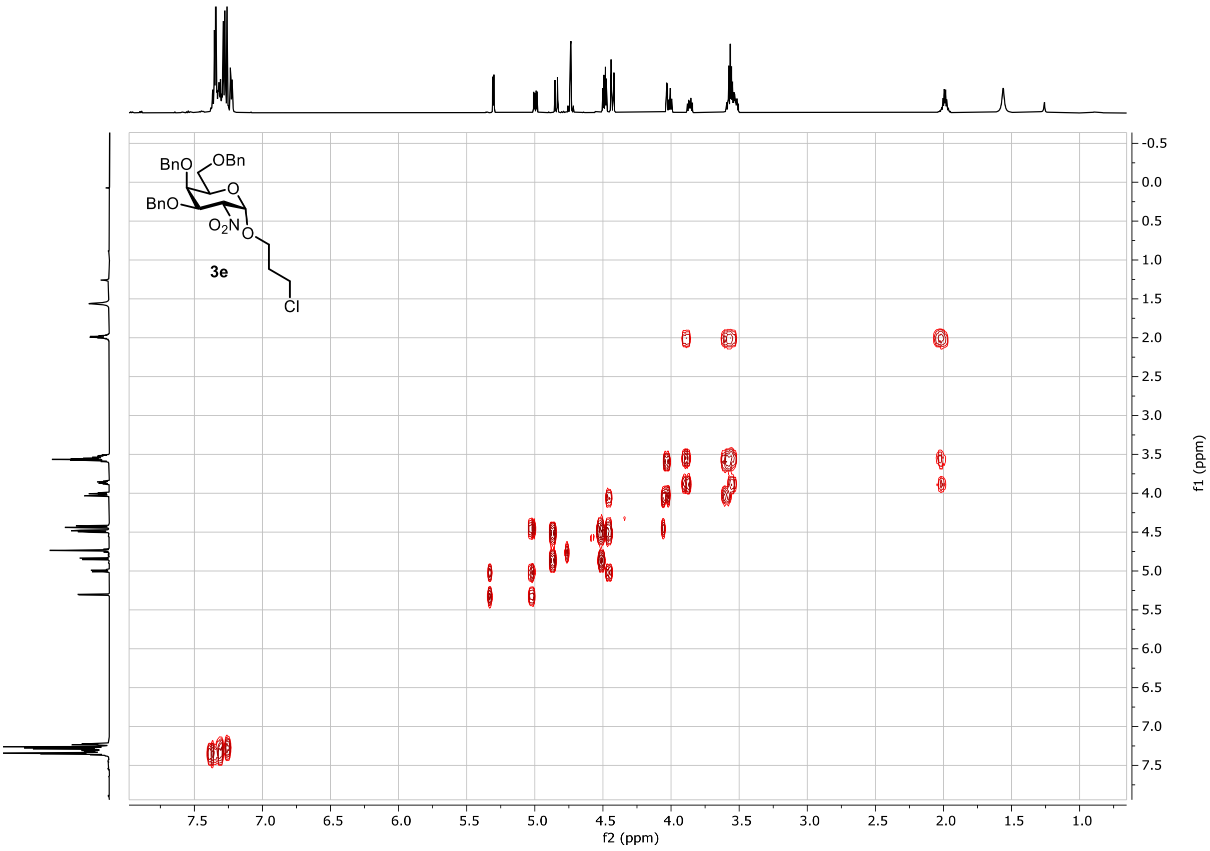

HSQC NMR ($CDCl_3$, 600 MHz)

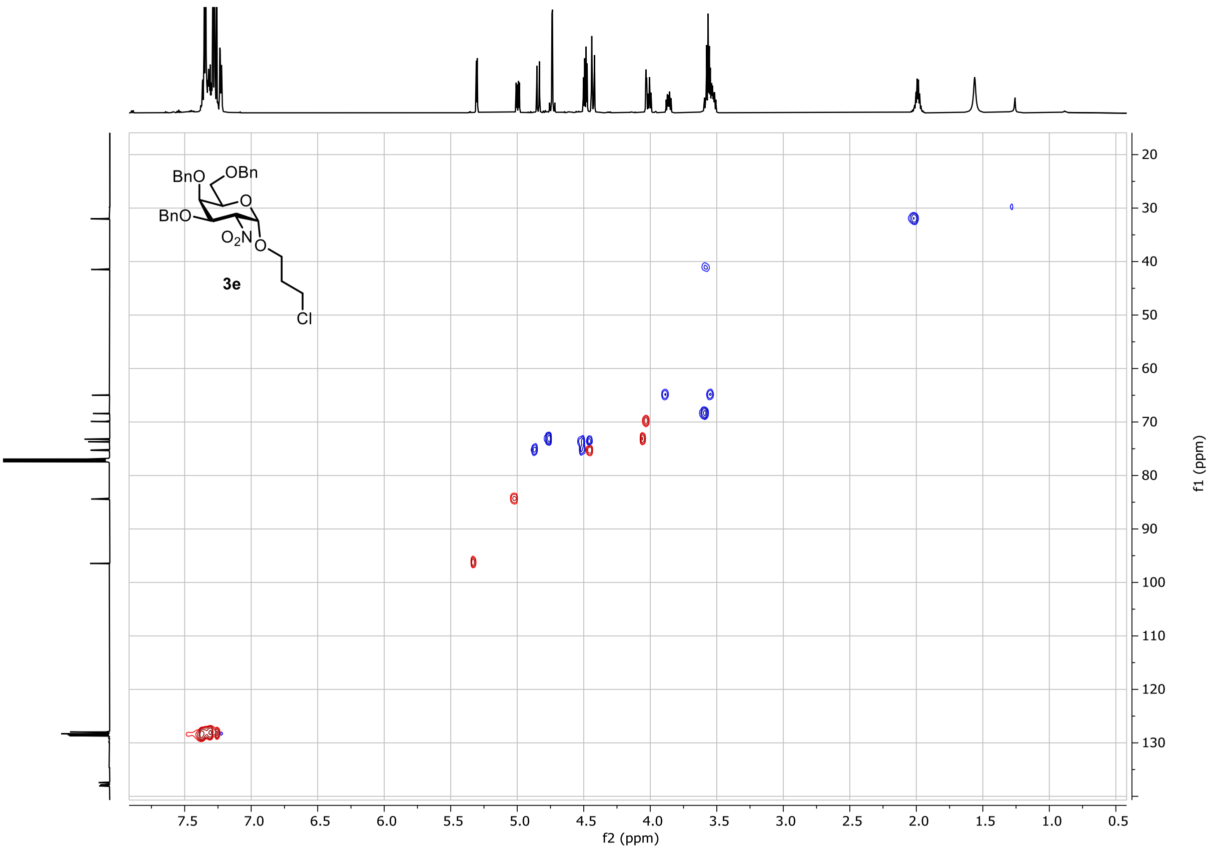

^{1}H NMR ($CDCl_3$, 400 MHz)

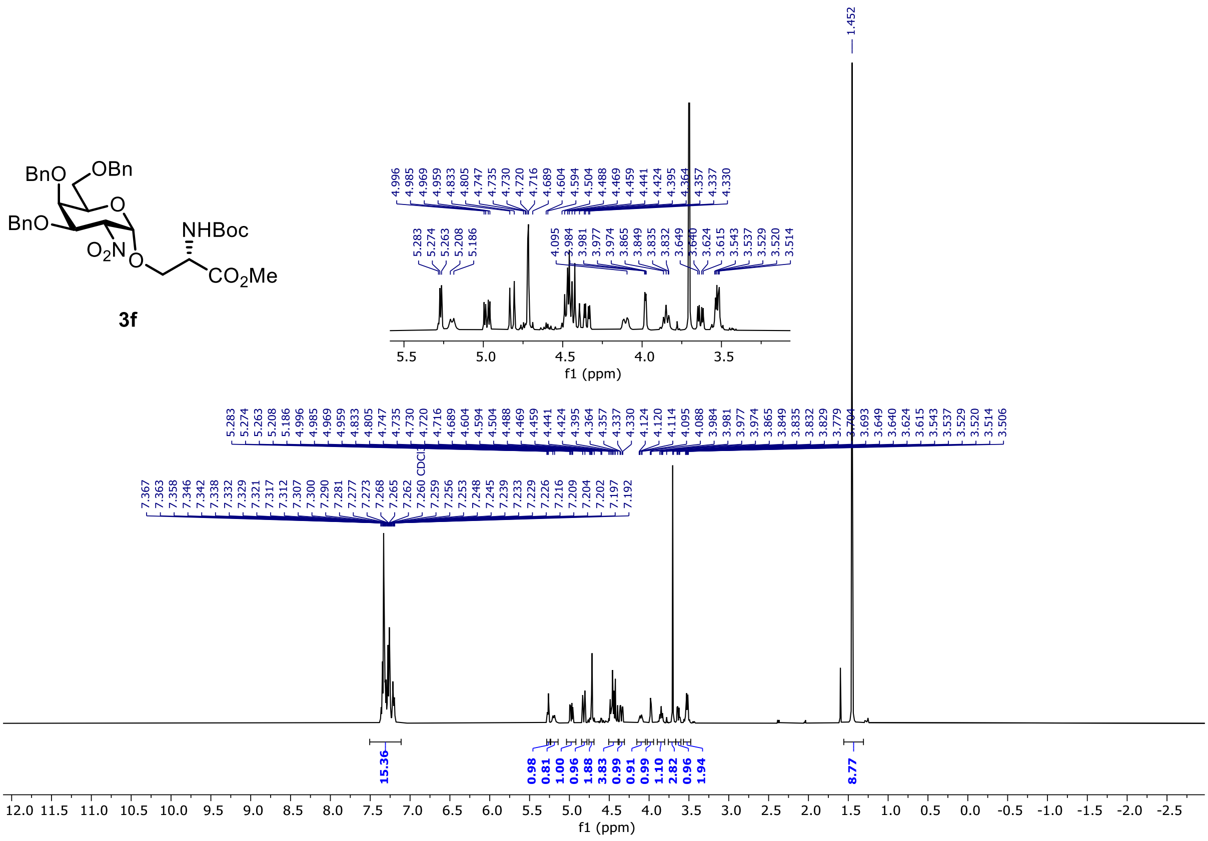

3f

$^{13}C\{^{1}H\}$ NMR ($CDCl_3$, 101 MHz)

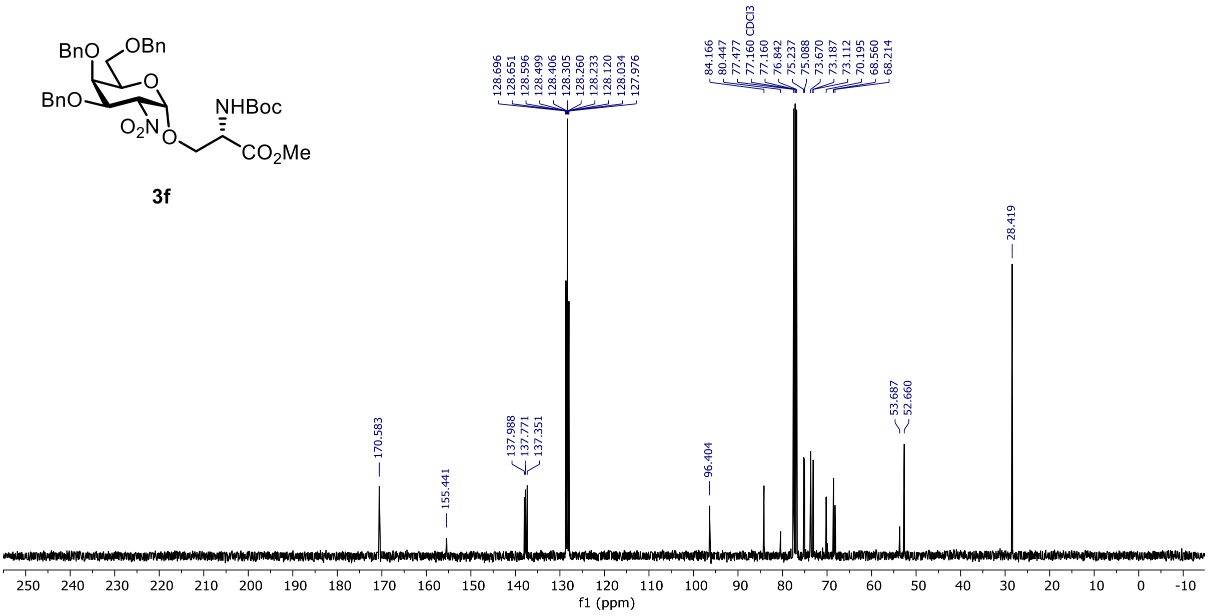

3f

COSY NMR ($CDCl_3$, 400 MHz)

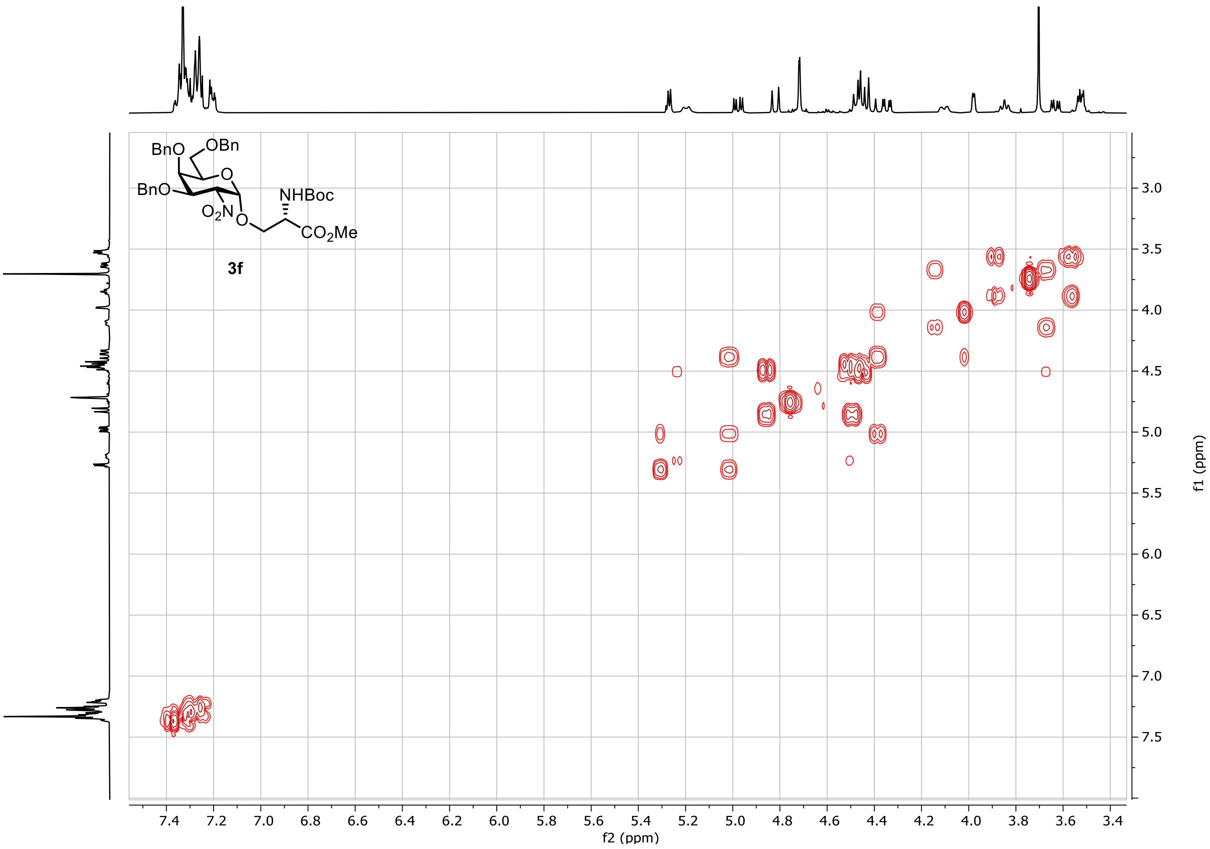

HSQC NMR ($CDCl_3$, 400 MHz)

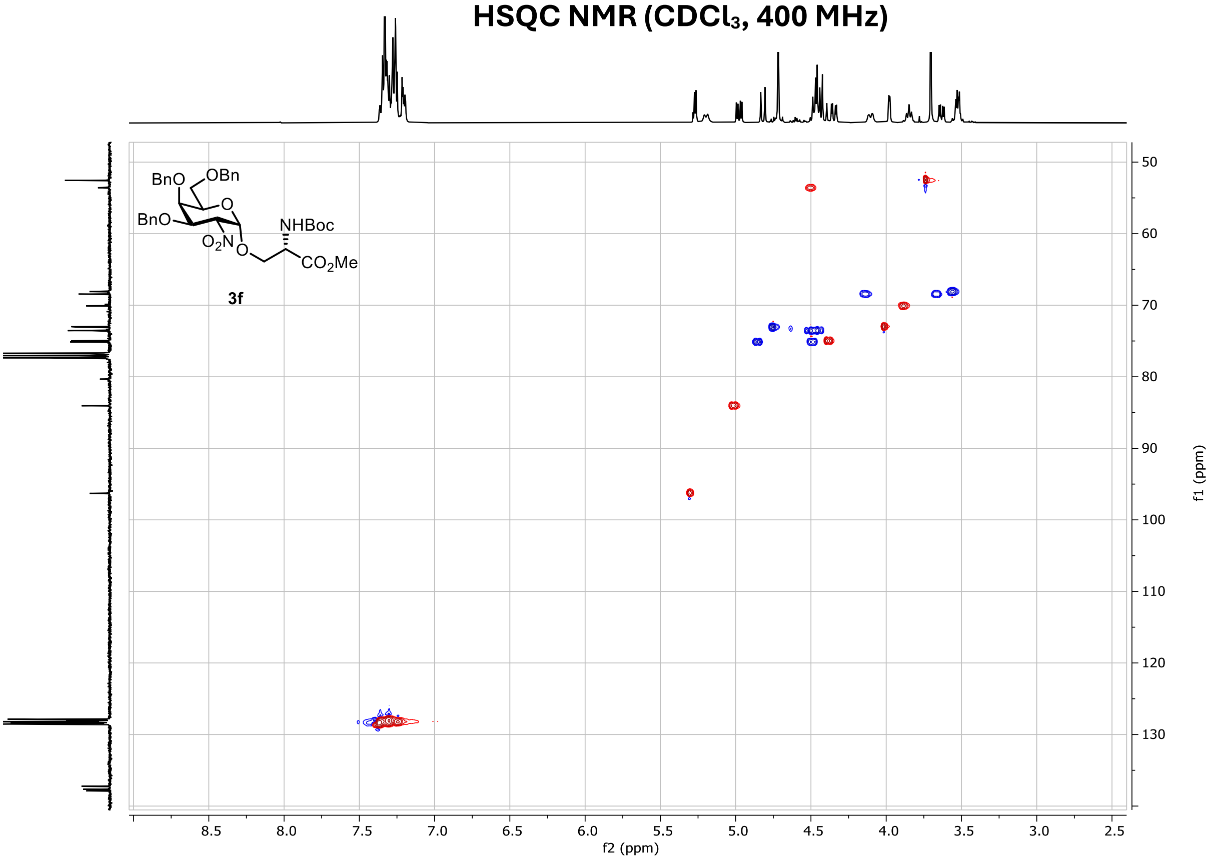

^{1}H NMR ($CDCl_3$, 400 MHz)

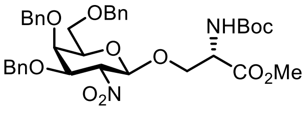

3f

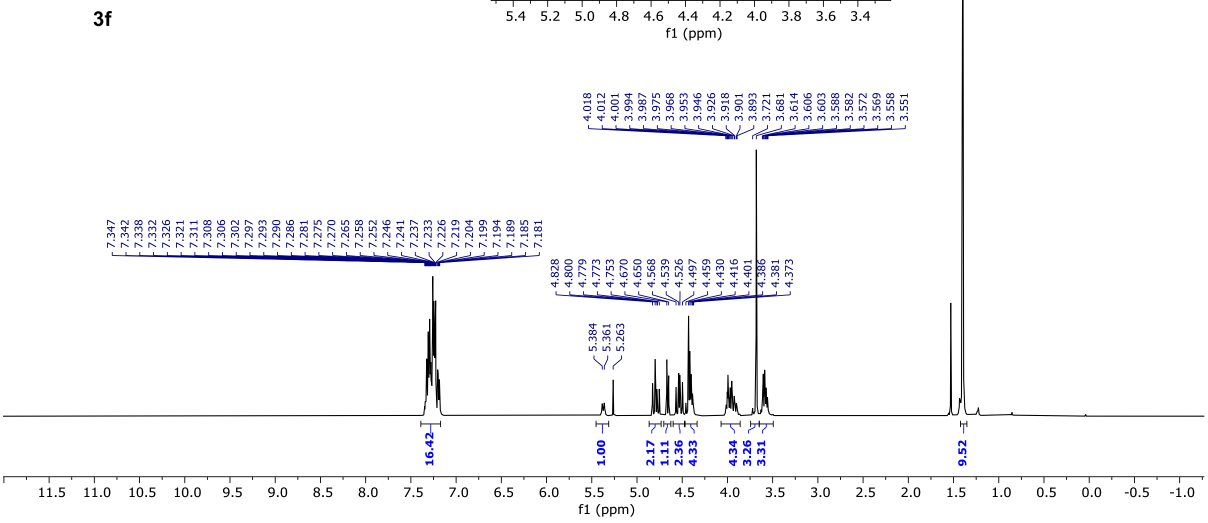

$^{13}C\{^1H\}$ NMR ($CDCl_3$, 101 MHz)

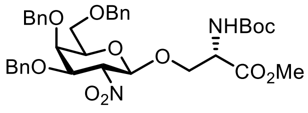

3f

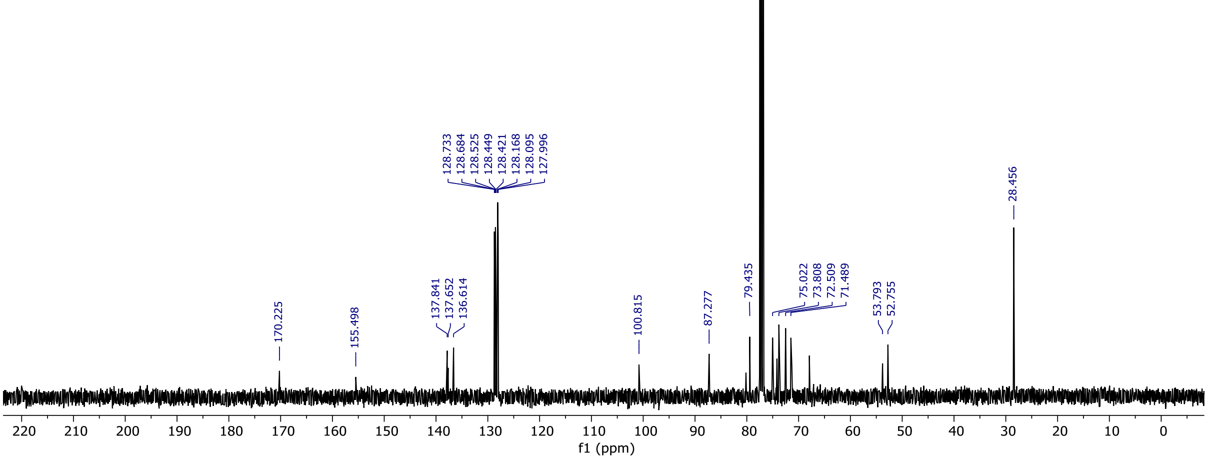

COSY NMR ($CDCl_3$, 400 MHz)

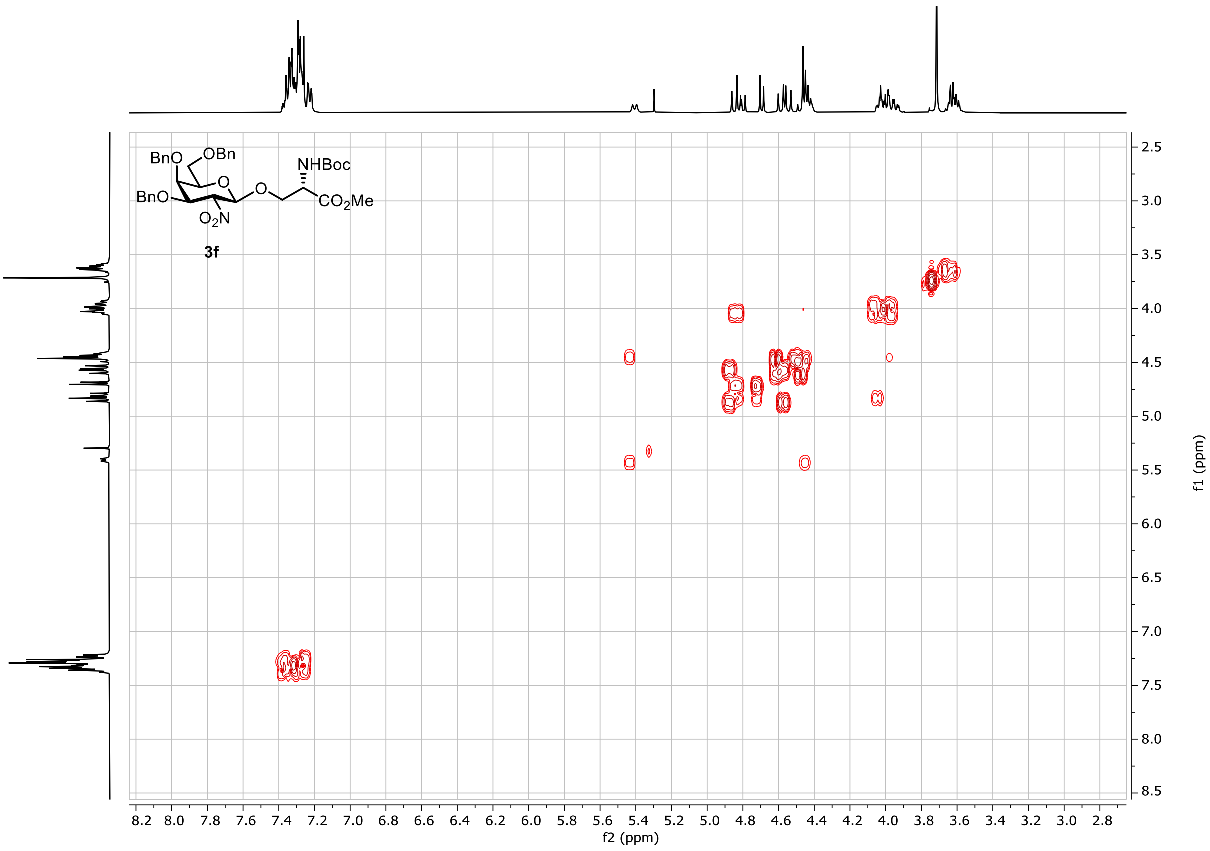

HSQC NMR ($CDCl_3$, 400 MHz)

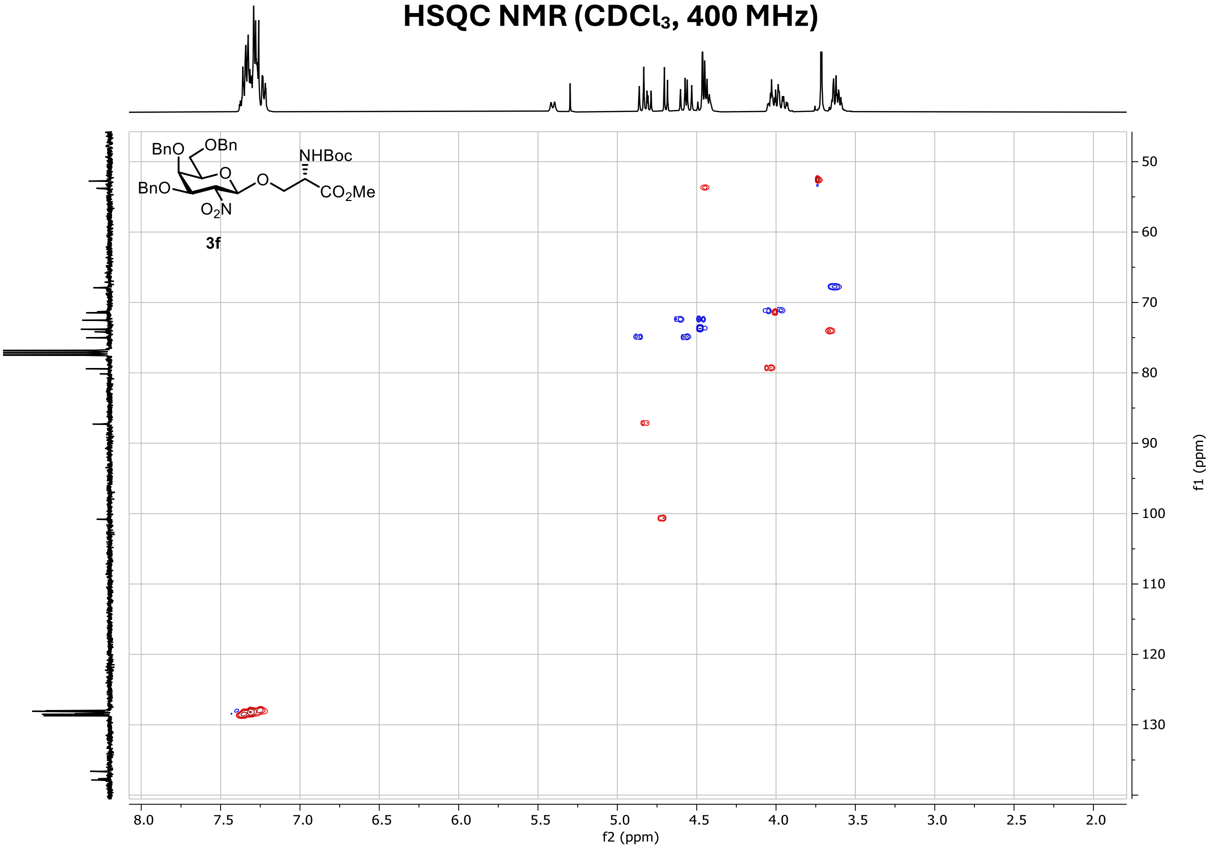

^{1}H NMR ($CDCl_3$, 400 MHz)

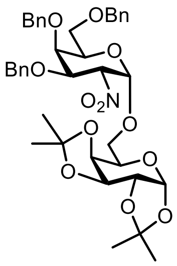

3g

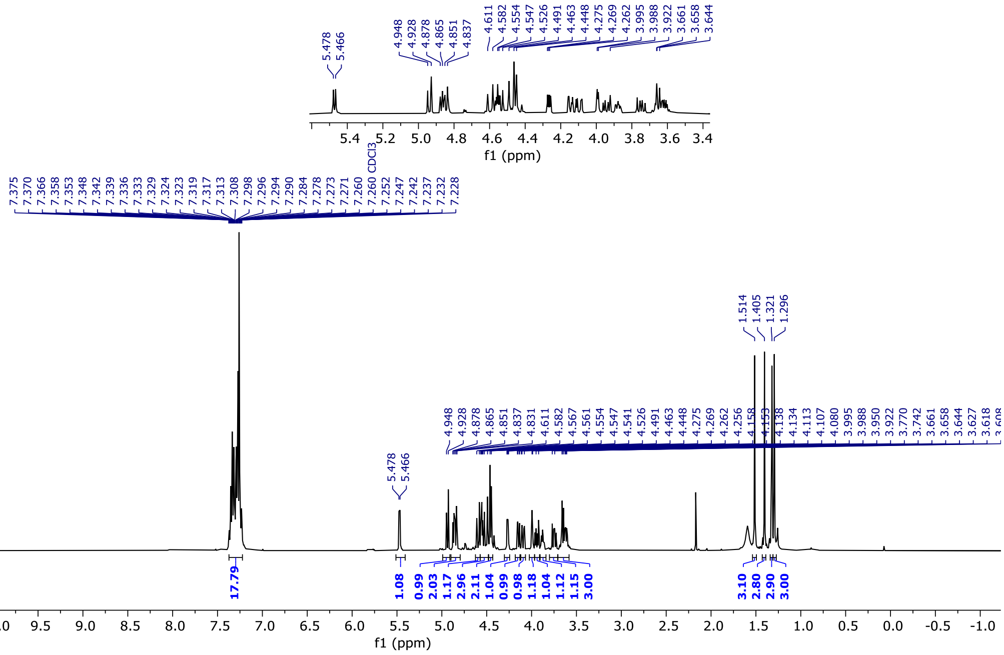

^{13}C{^{1}H} NMR ($CDCl_3$, 101 MHz)

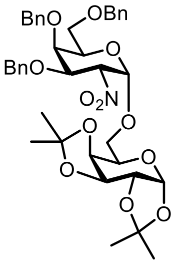

3g

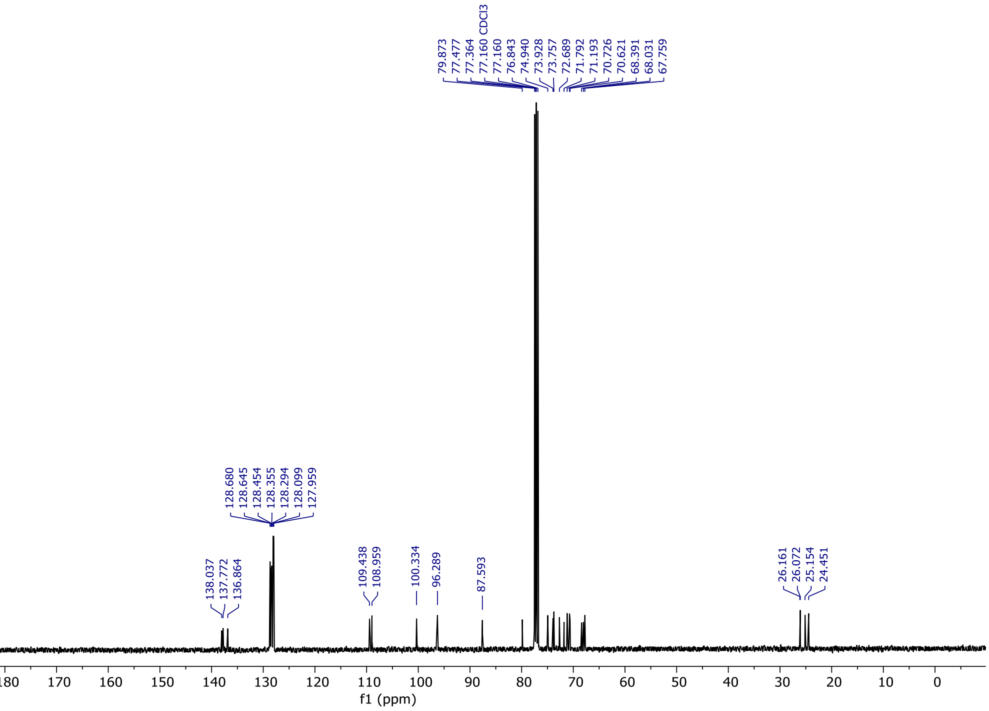

COSY NMR ($CDCl_3$, 400 MHz)

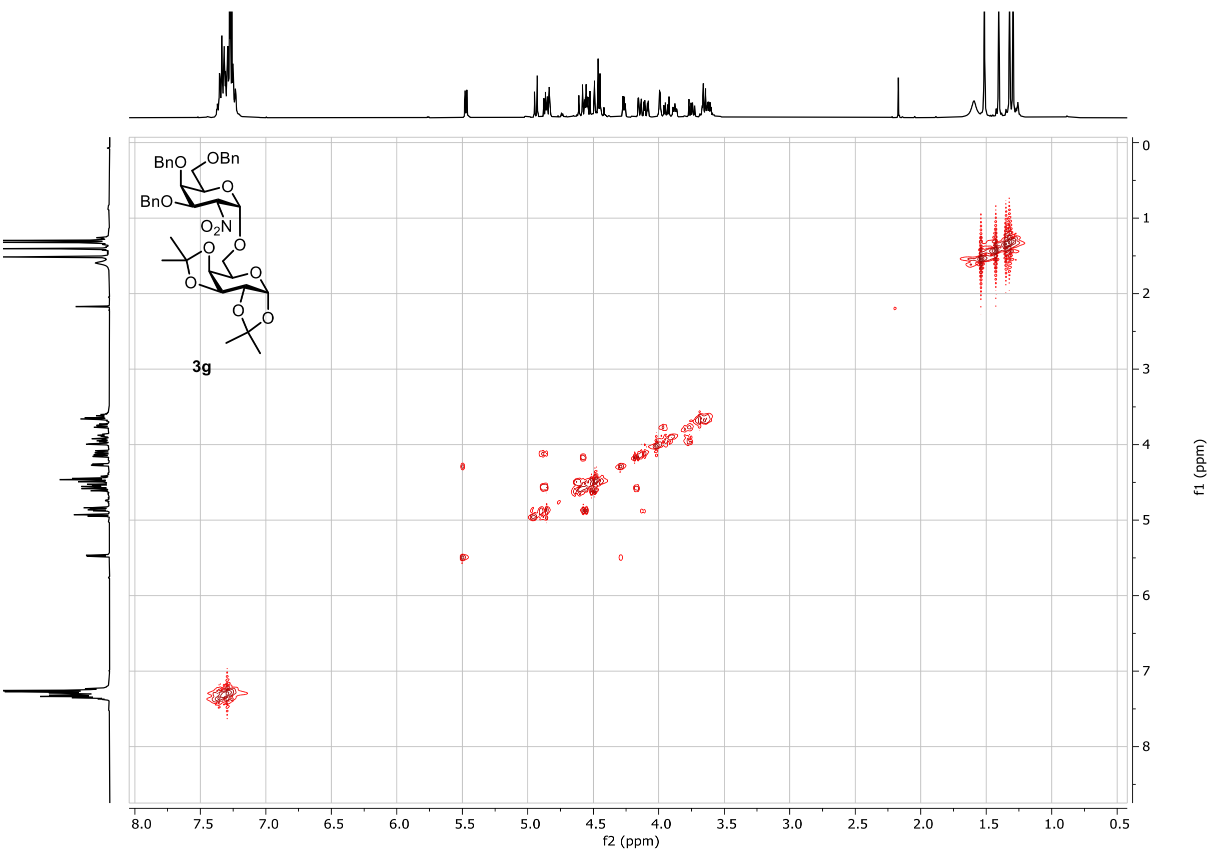

HSQC ($CDCl_3$, 400 MHz)

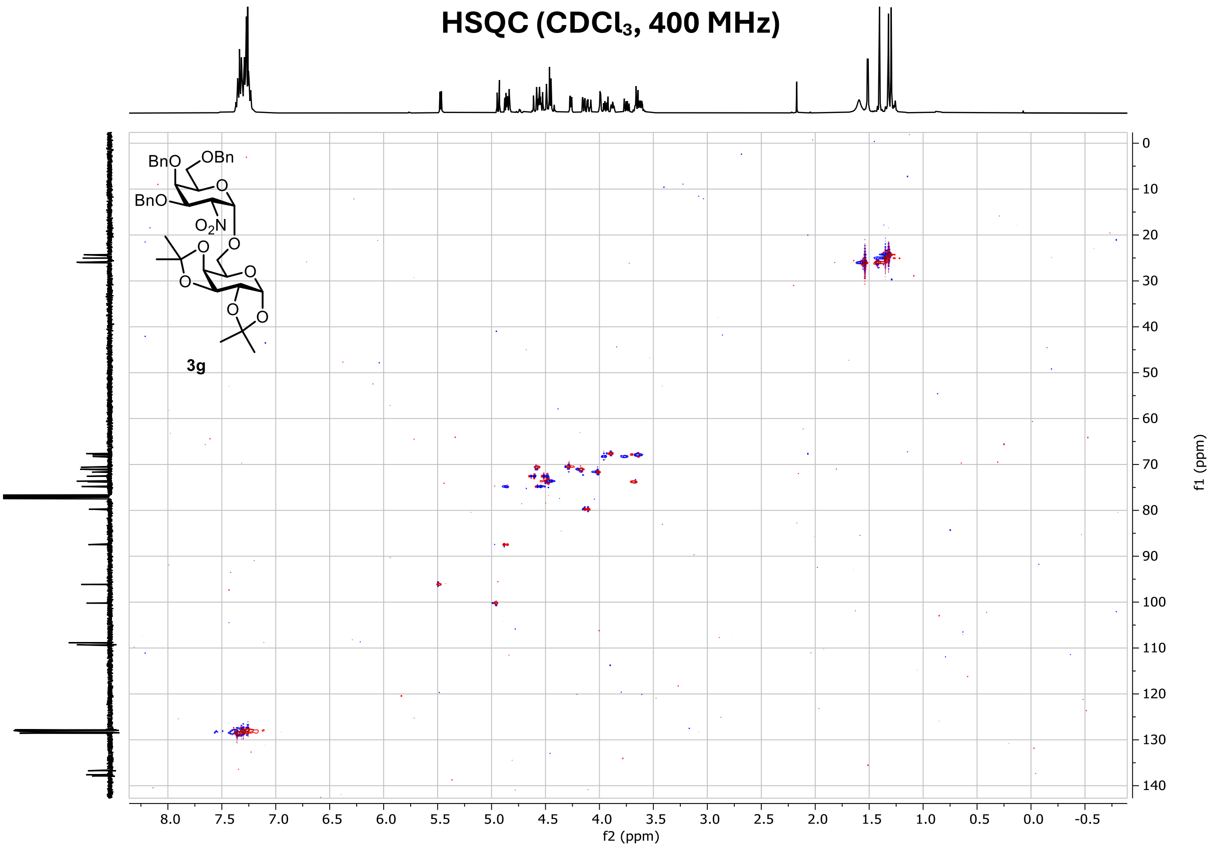

^{1}H NMR ($CDCl_3$, 400 MHz)

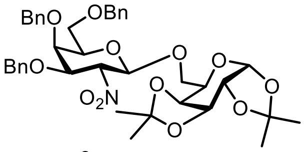

3g

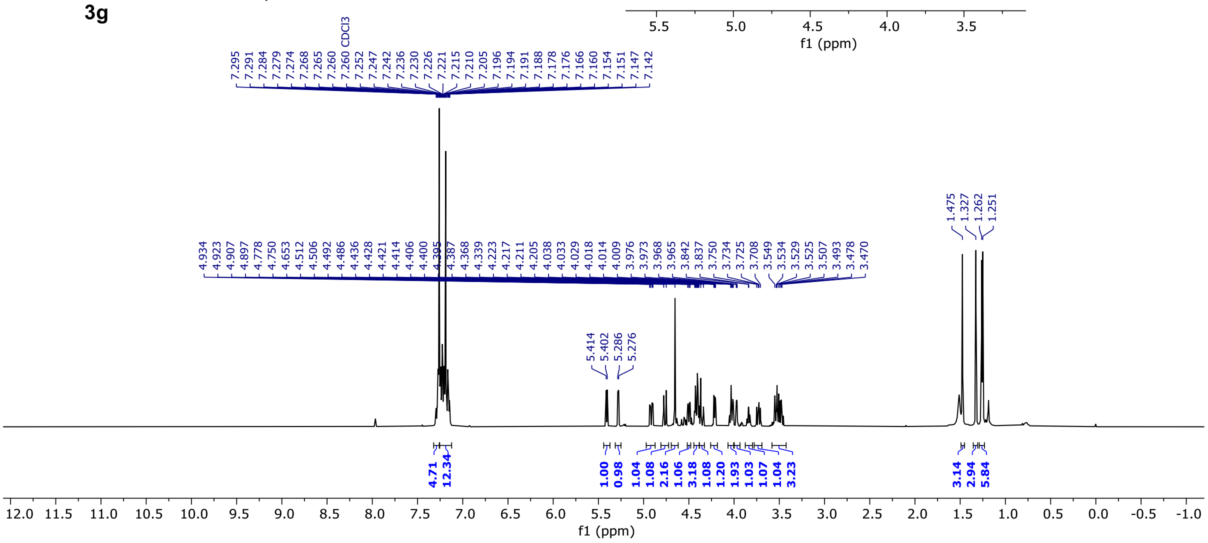

$^{13}C\{^{1}H\}$ NMR ($CDCl_3$, 101 MHz)

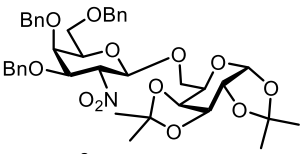

3g

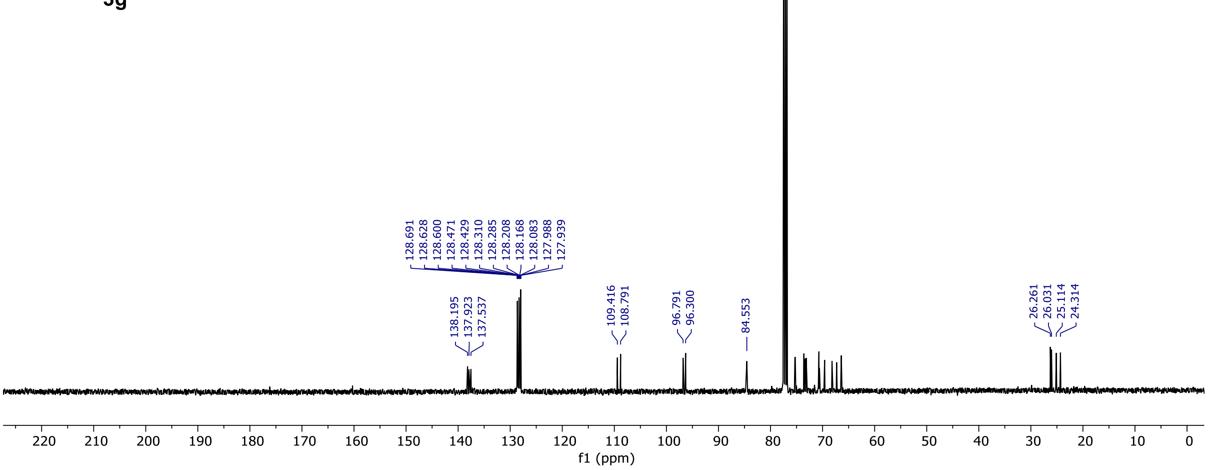

COSY NMR ($CDCl_3$, 400 MHz)

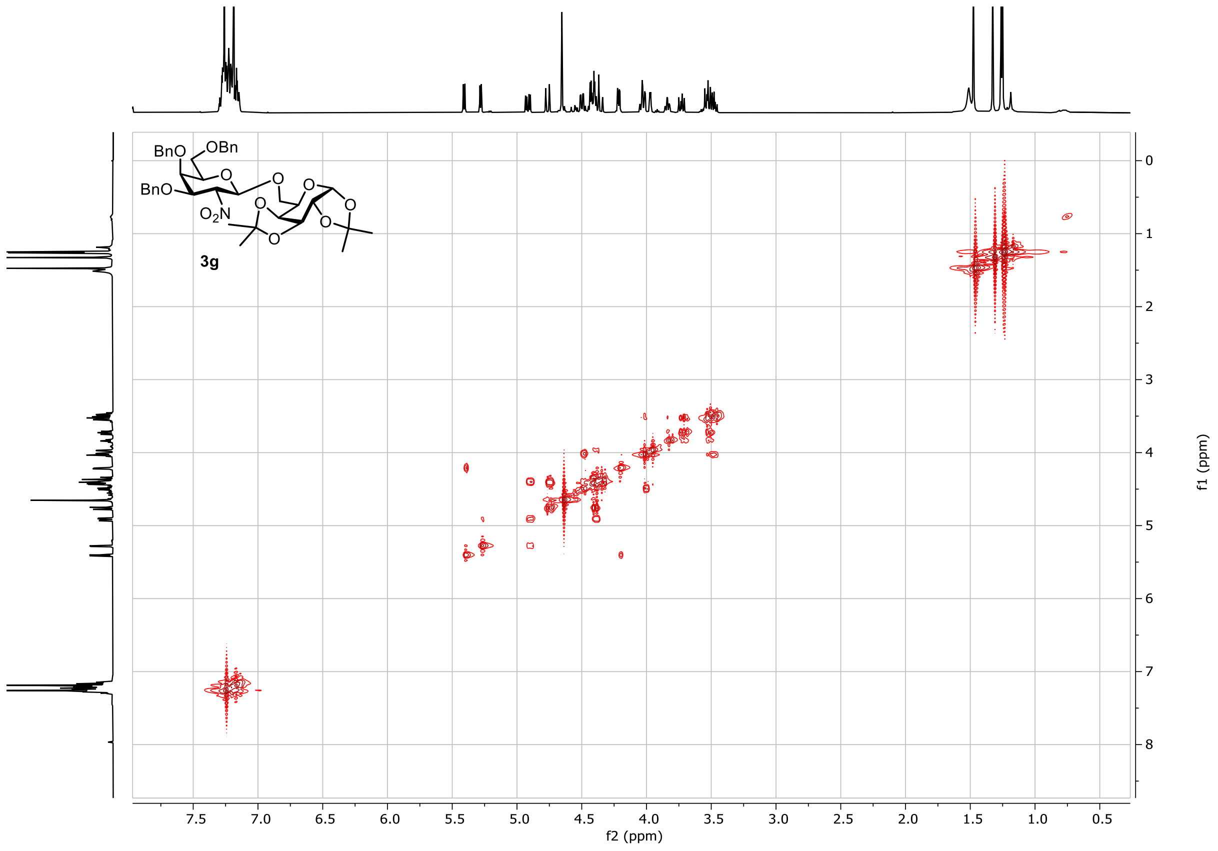

HSQC ($CDCl_3$, 400 MHz)

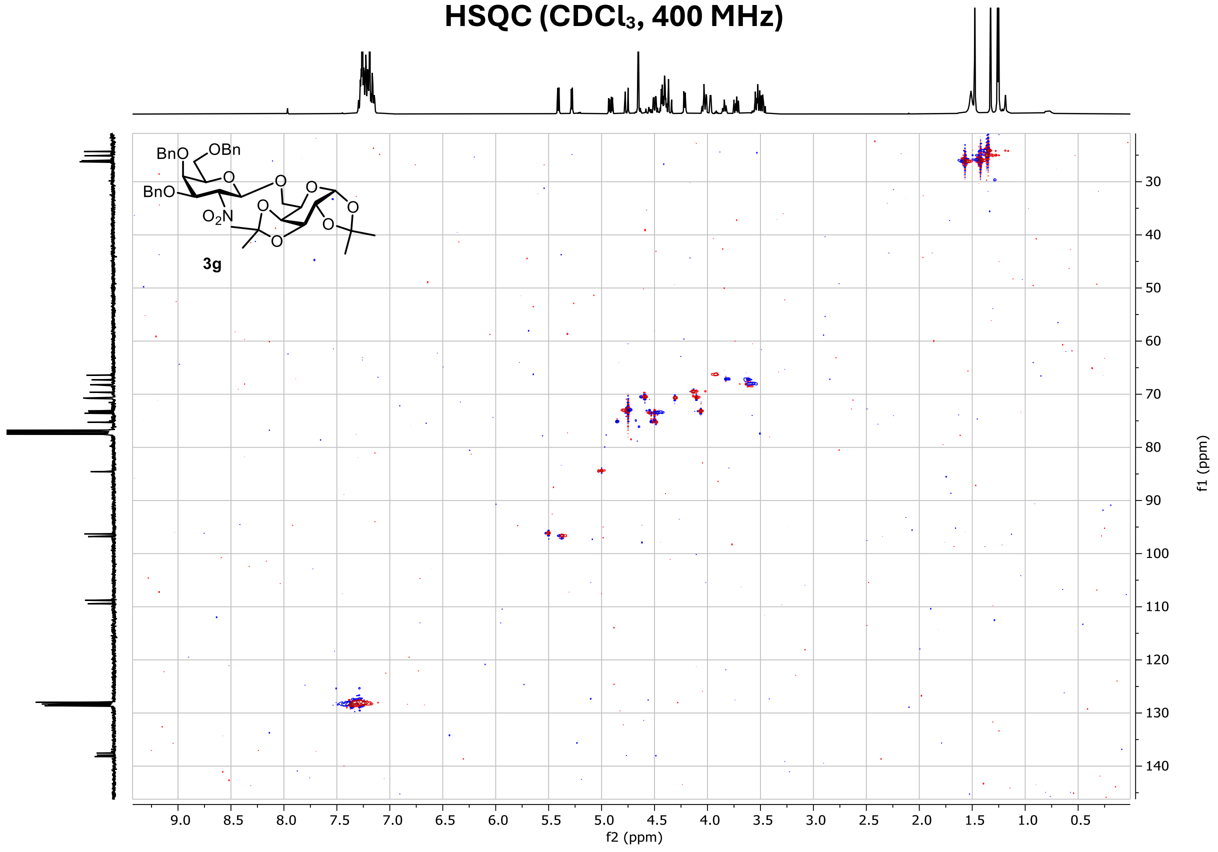

^{1}H NMR ($CDCl_3$, 400 MHz)

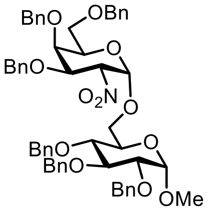

3h

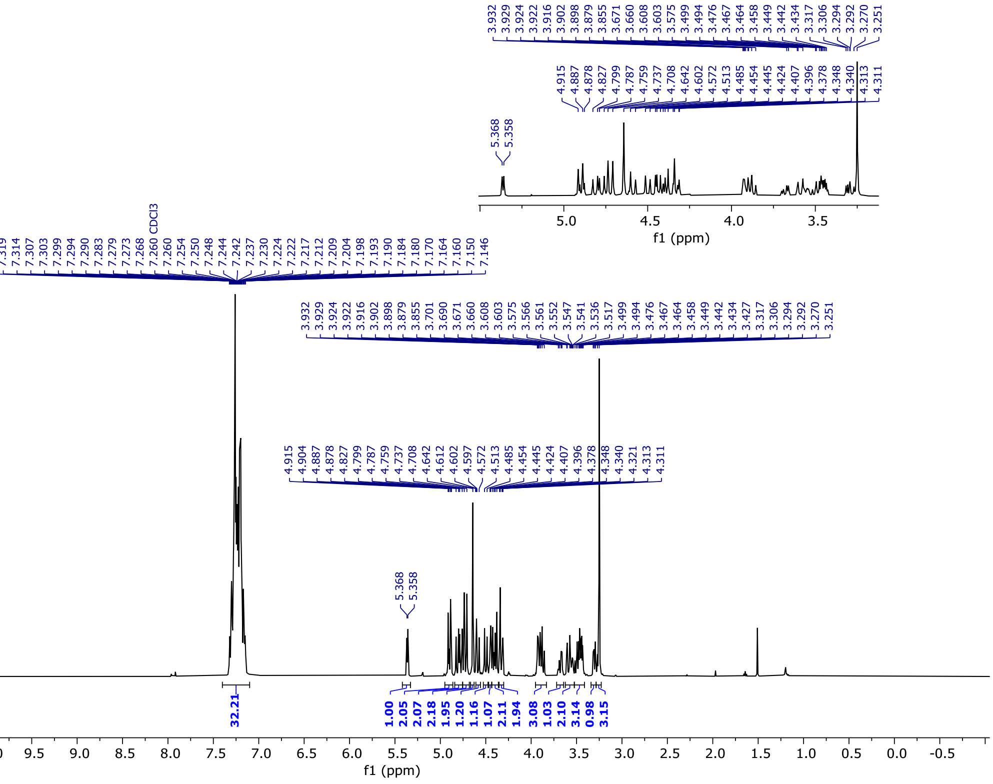

^{13}C{^{1}H} NMR ($CDCl_3$, 101 MHz)

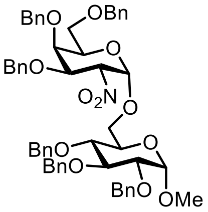

3h

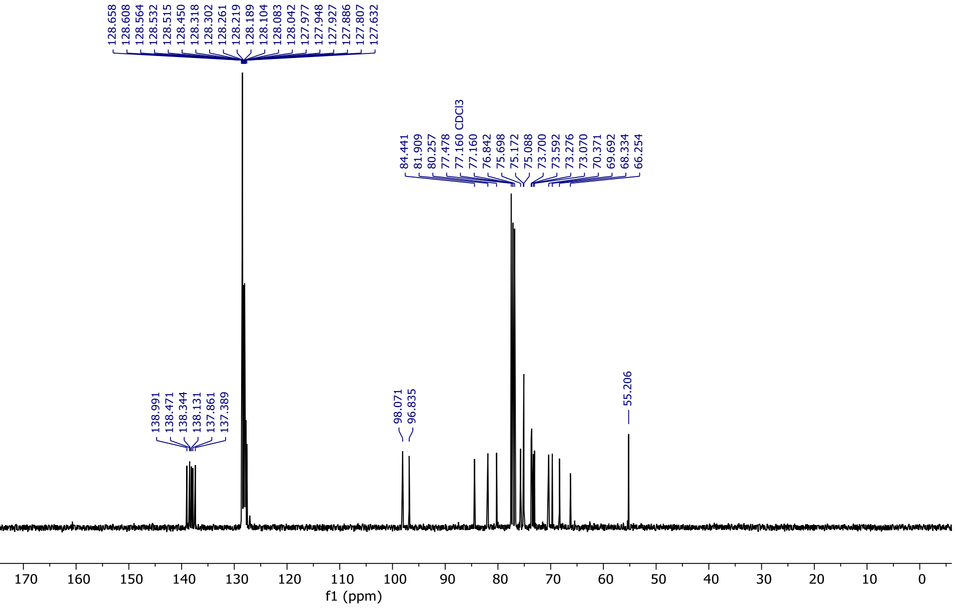

COSY NMR ($CDCl_3$, 400 MHz)

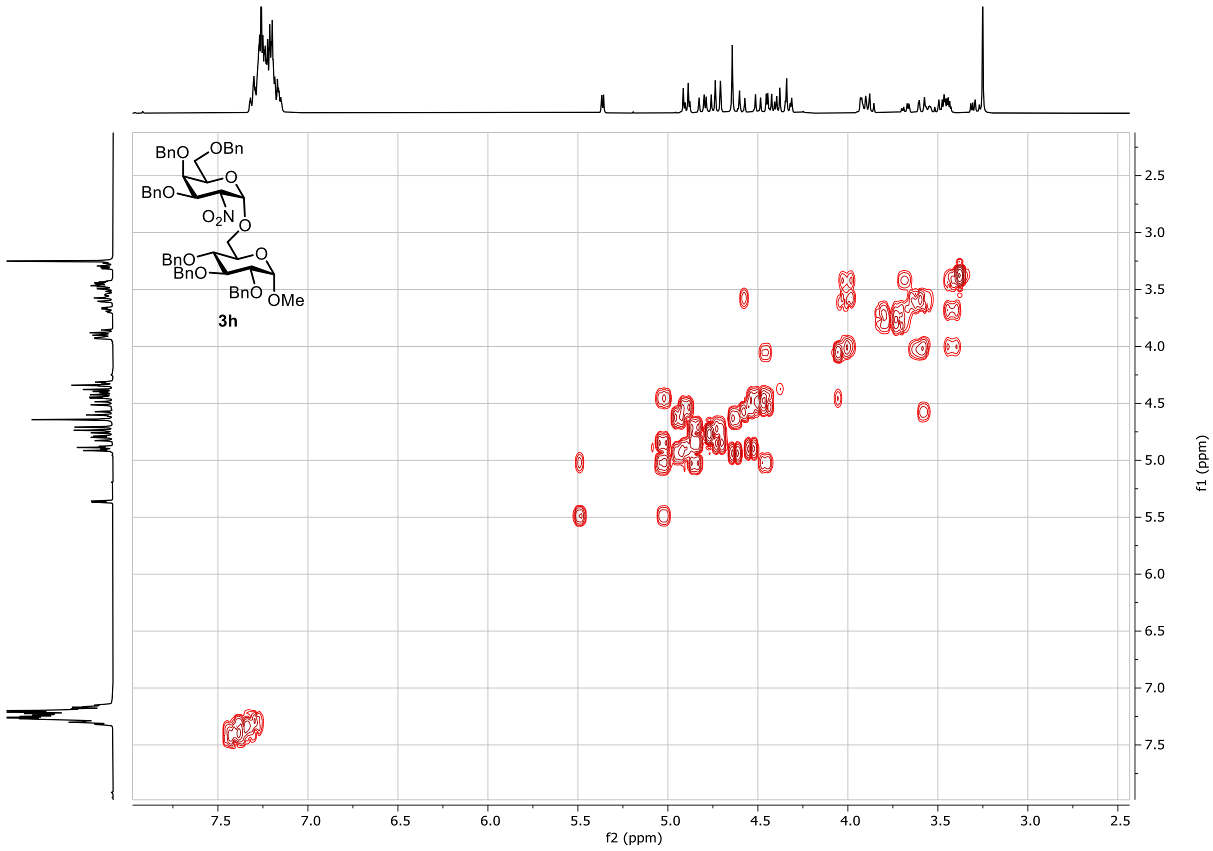

HSQC NMR ($CDCl_3$, 400 MHz)

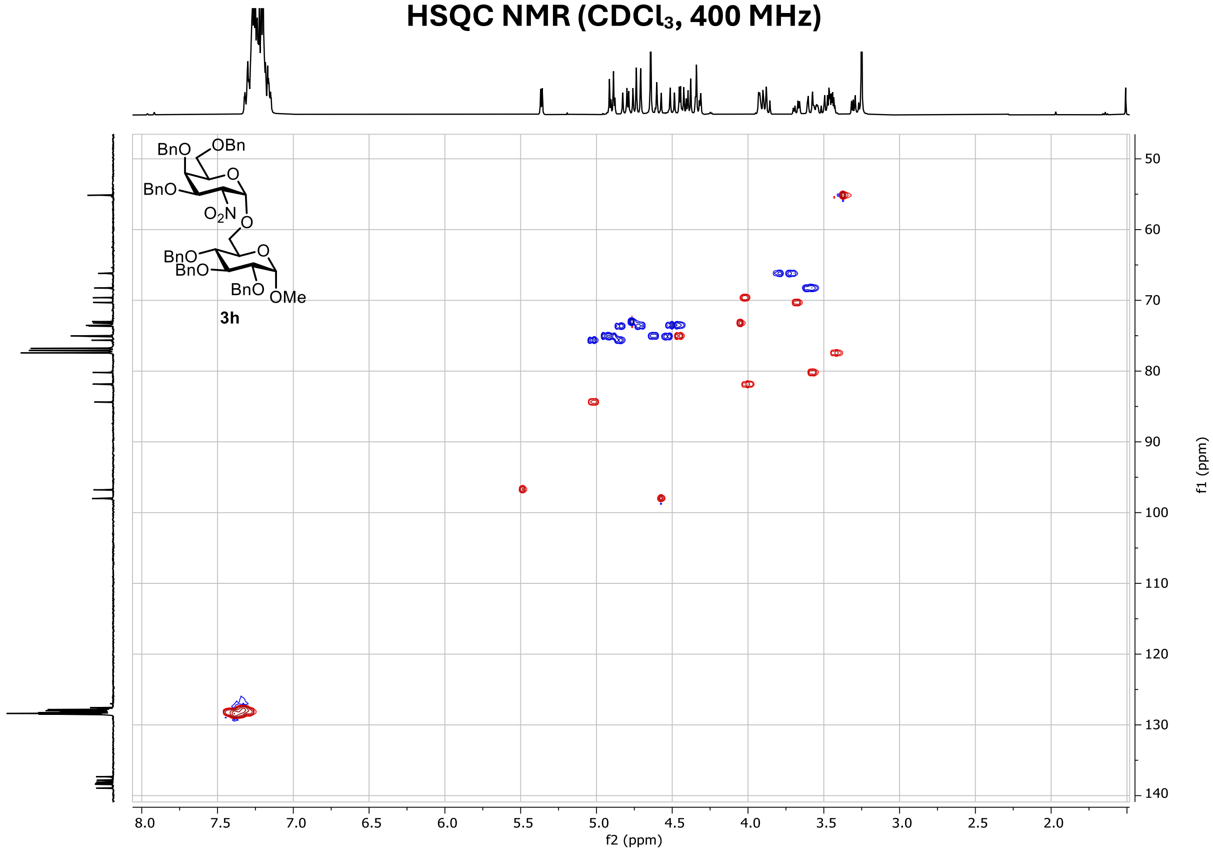

^{1}H NMR (CDCl$_3$, 400 MHz)

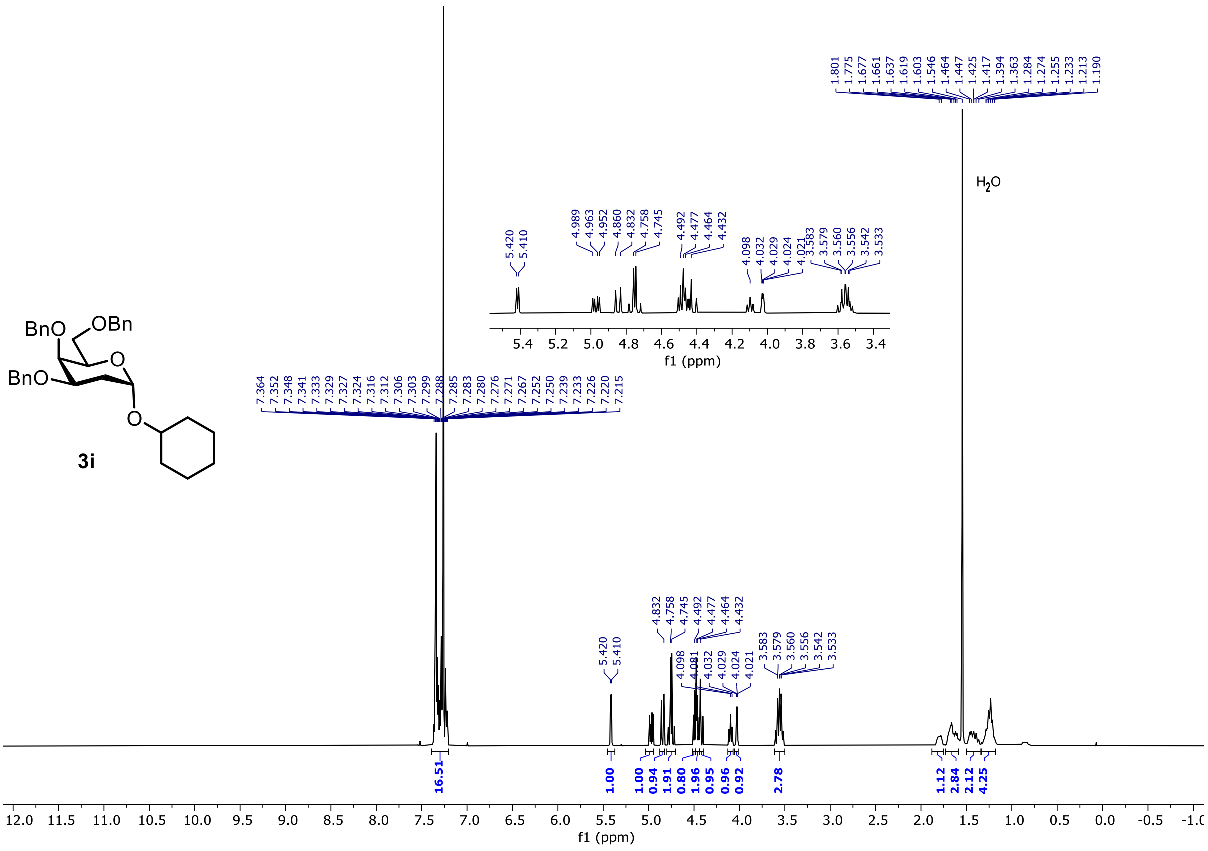

^{13}C{^{1}H} NMR (CDCl$_3$, 101 MHz)

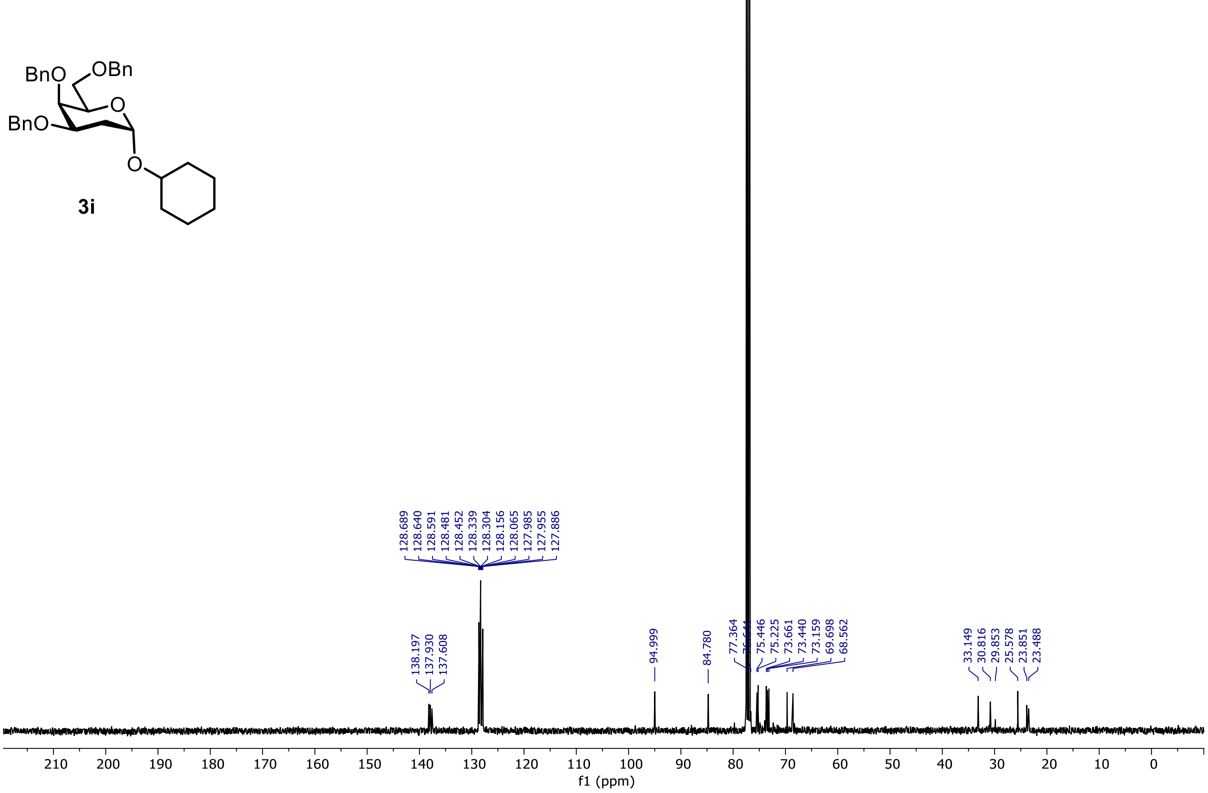

COSY NMR ($CDCl_3$, 400 MHz)

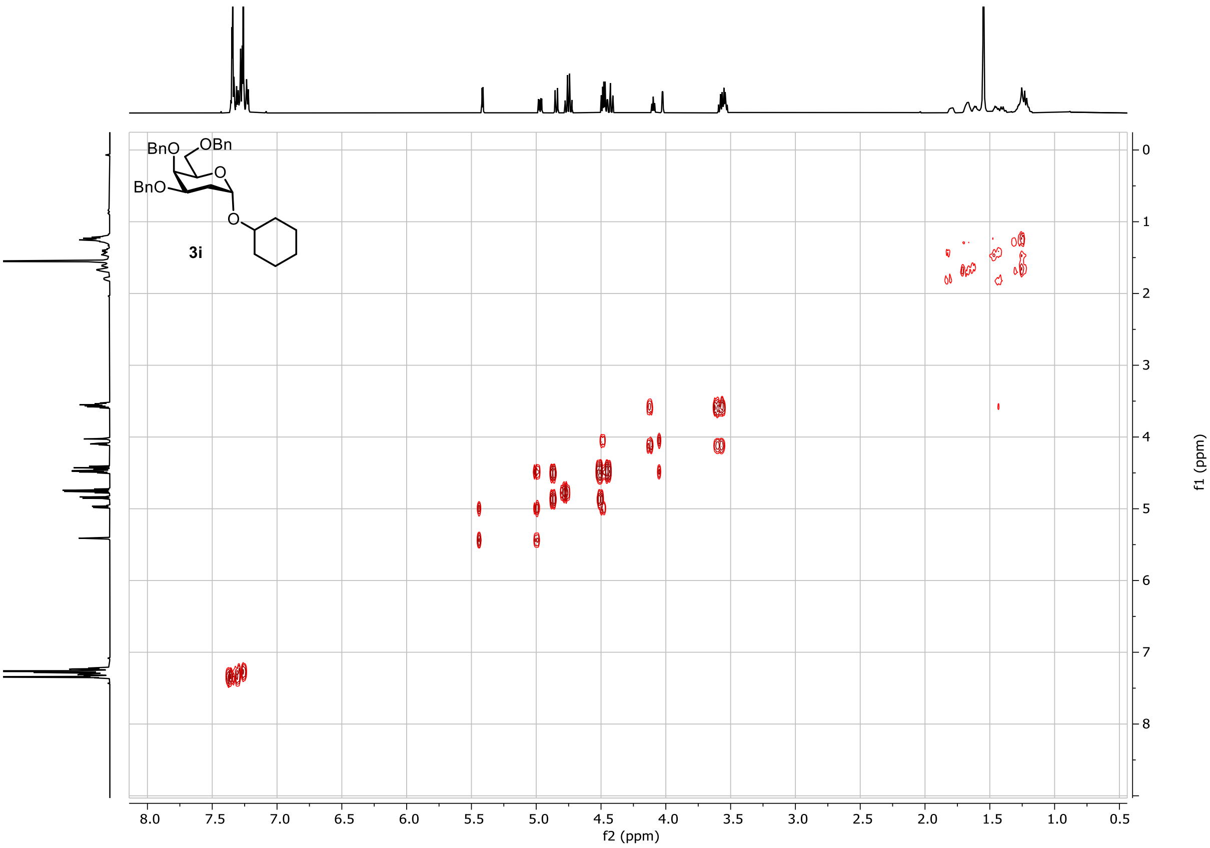

HSQC NMR ($CDCl_3$, 400 MHz)

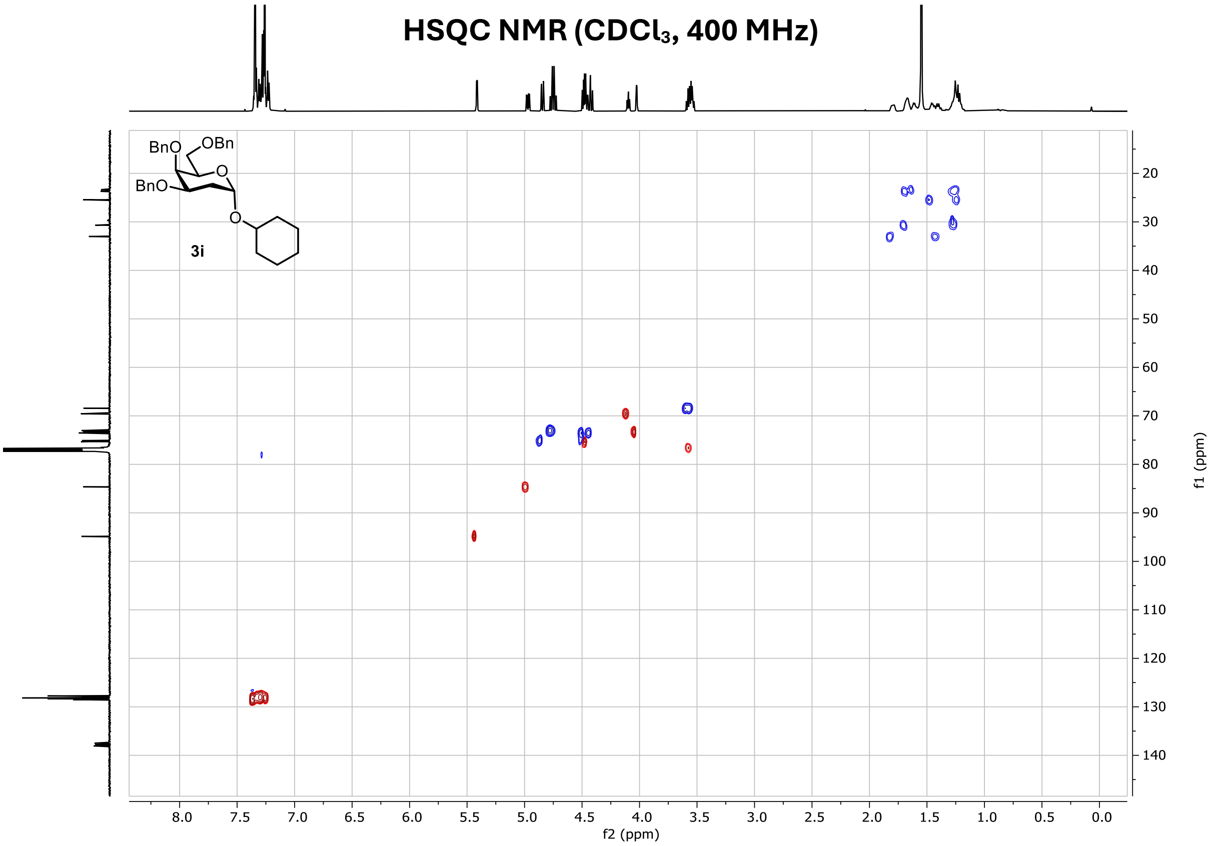

^{1}H NMR ($CDCl_3$, 400 MHz)

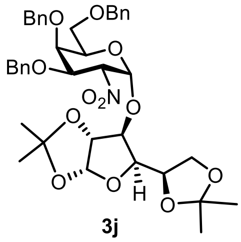

3j

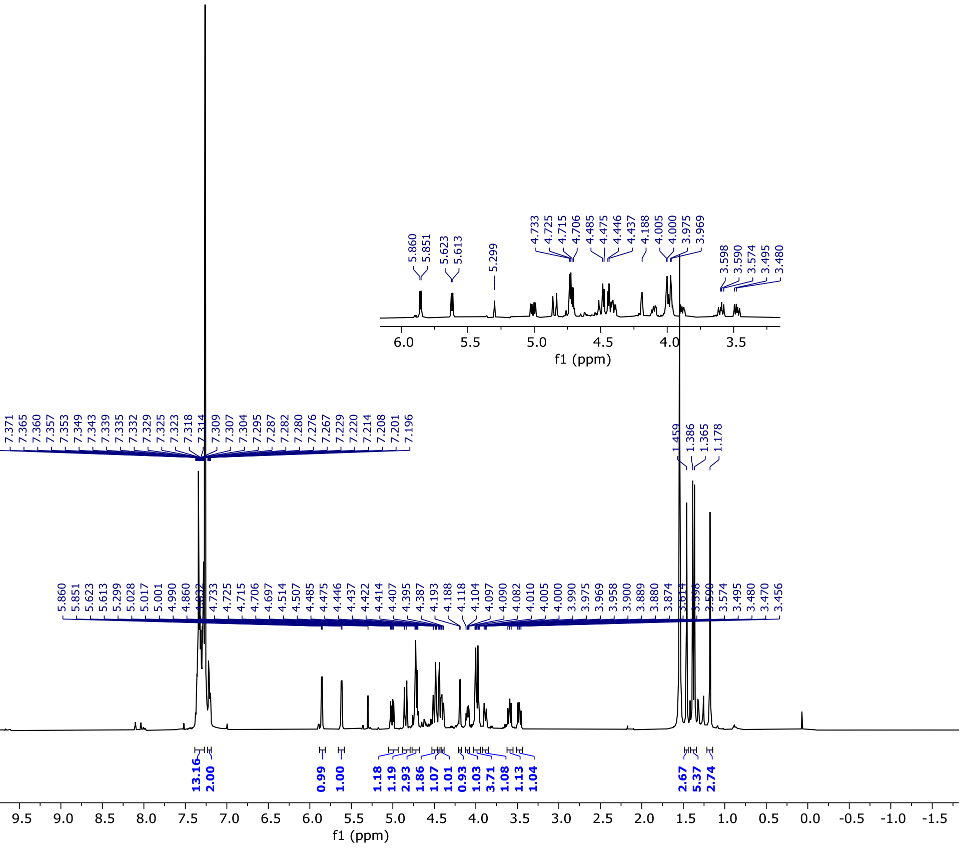

$^{13}C\{^1H\}$ NMR ($CDCl_3$, 101 MHz)

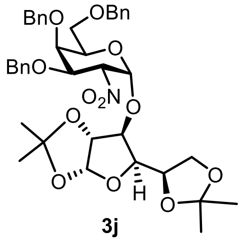

3j

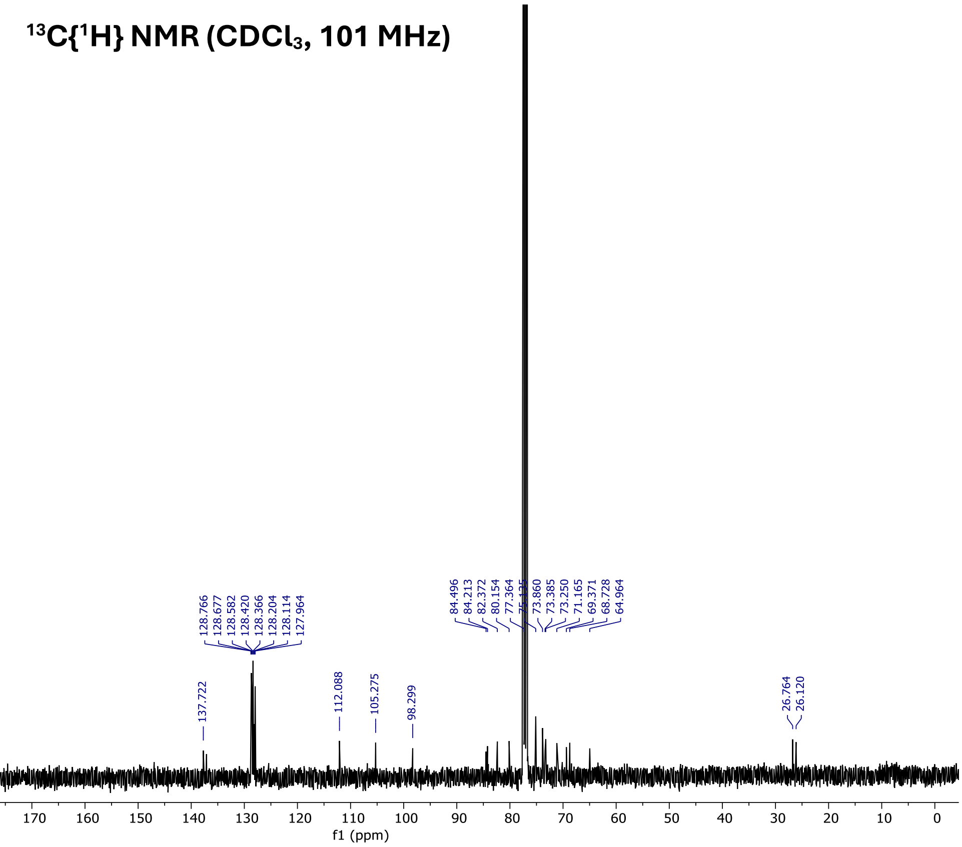

COSY NMR ($CDCl_3$, 400 MHz)

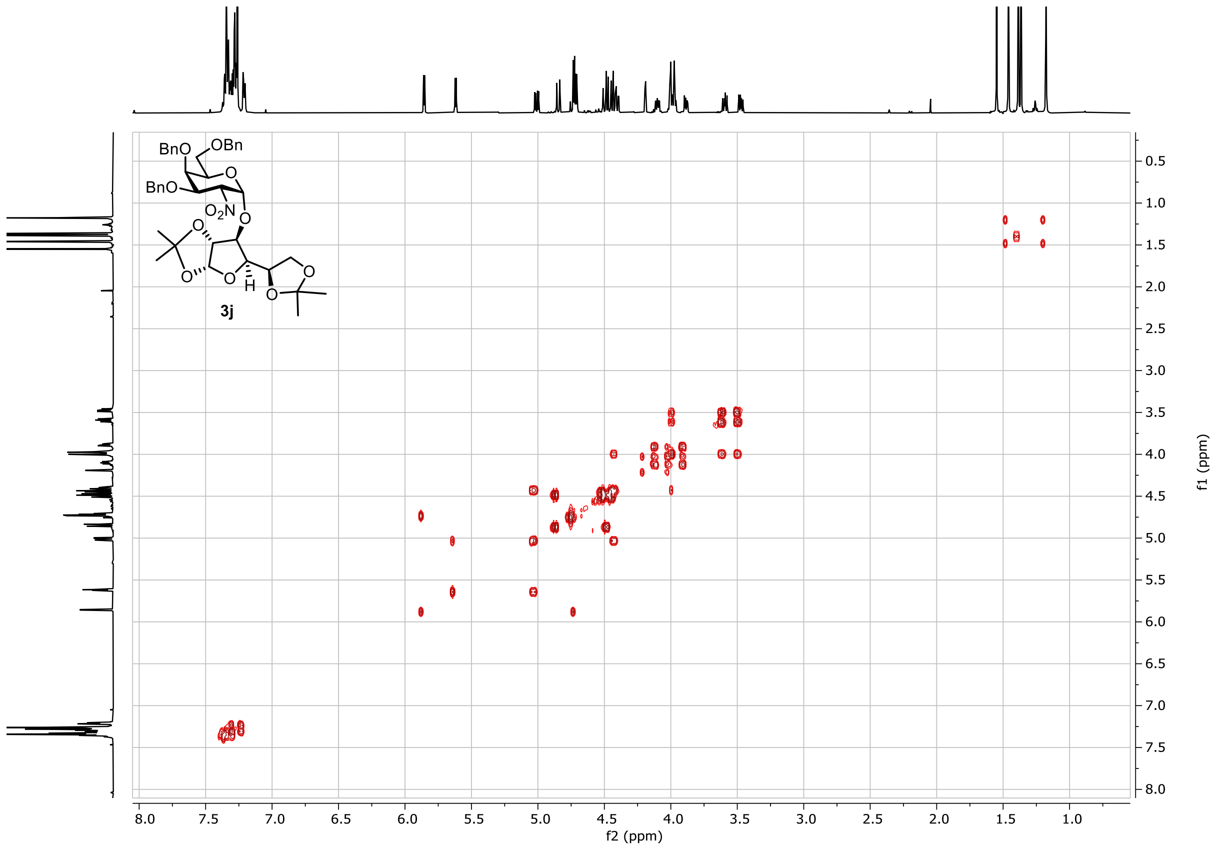

HSQC NMR ($CDCl_3$, 400 MHz)

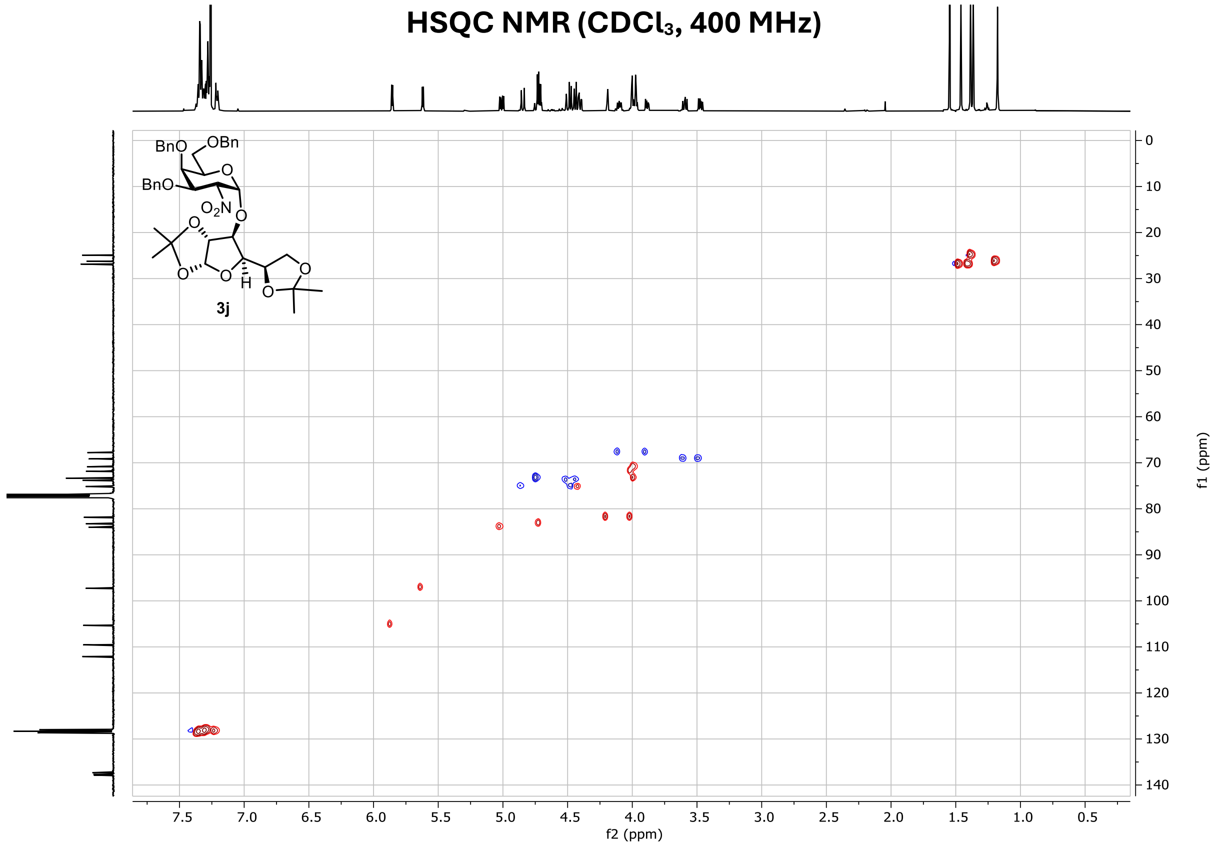

^{1}H NMR (CDCl$_3$, 400 MHz)

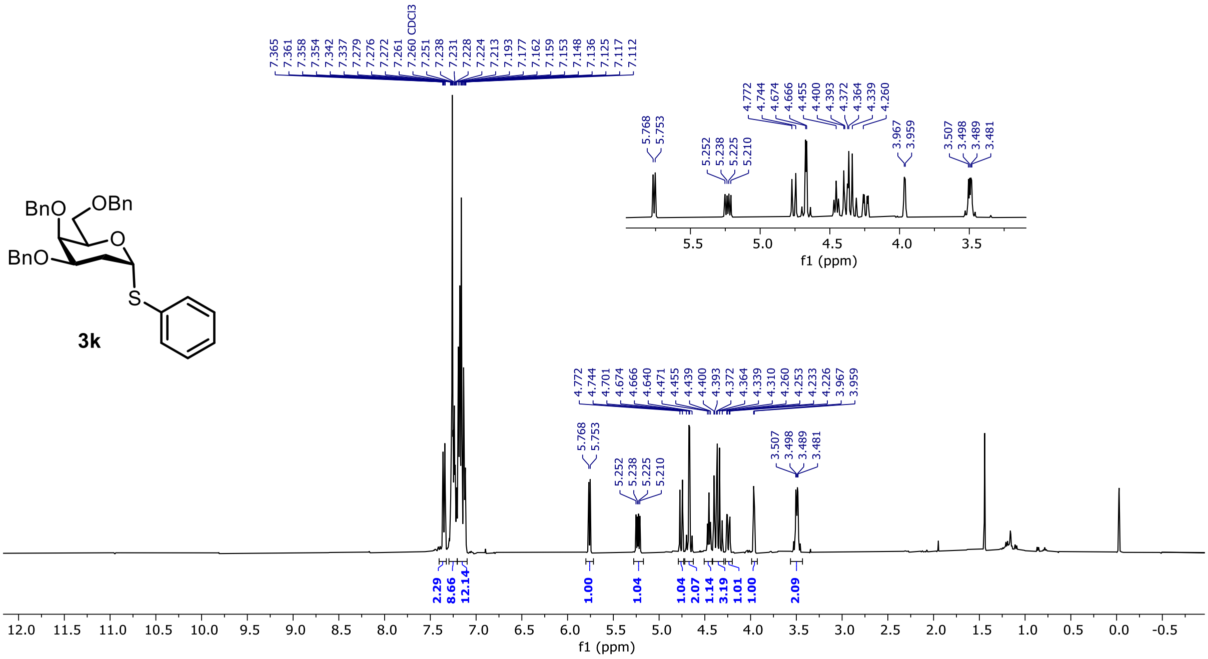

^{13}C{^{1}H} NMR (CDCl$_3$, 101 MHz)

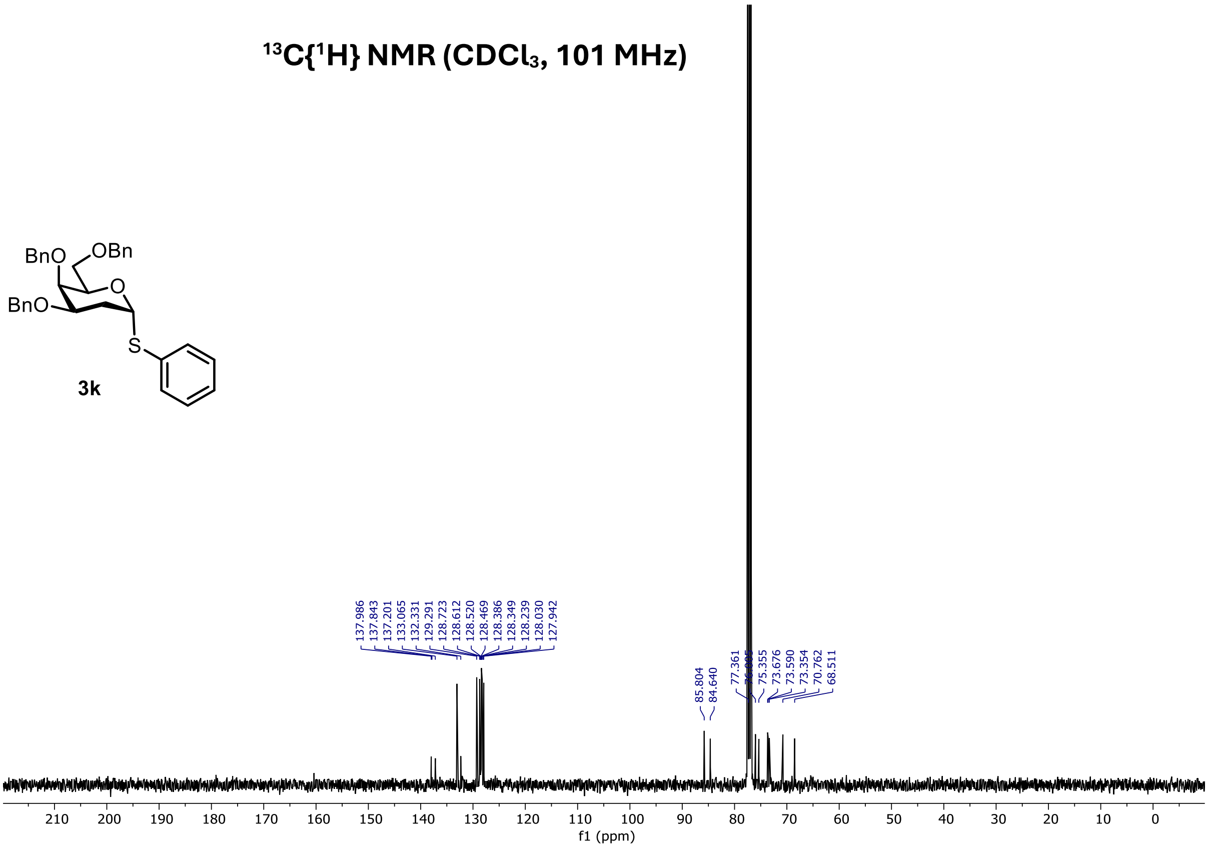

^{1}H NMR ($CDCl_3$, 400 MHz)

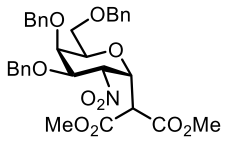

3l

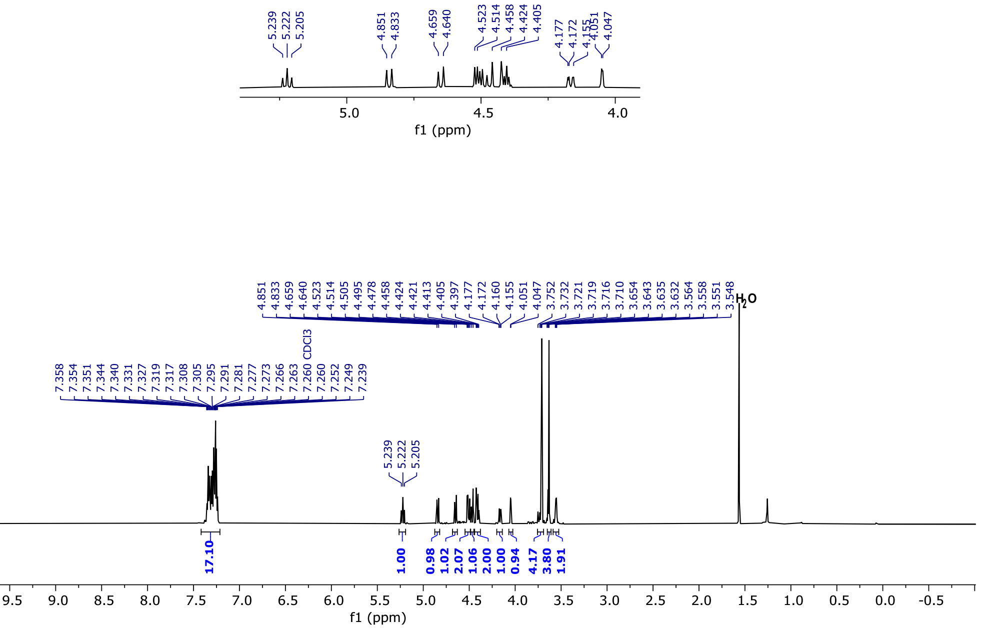

^{13}C{^{1}H} NMR ($CDCl_3$, 101 MHz)

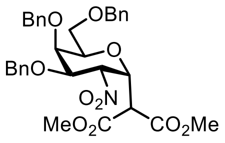

3l

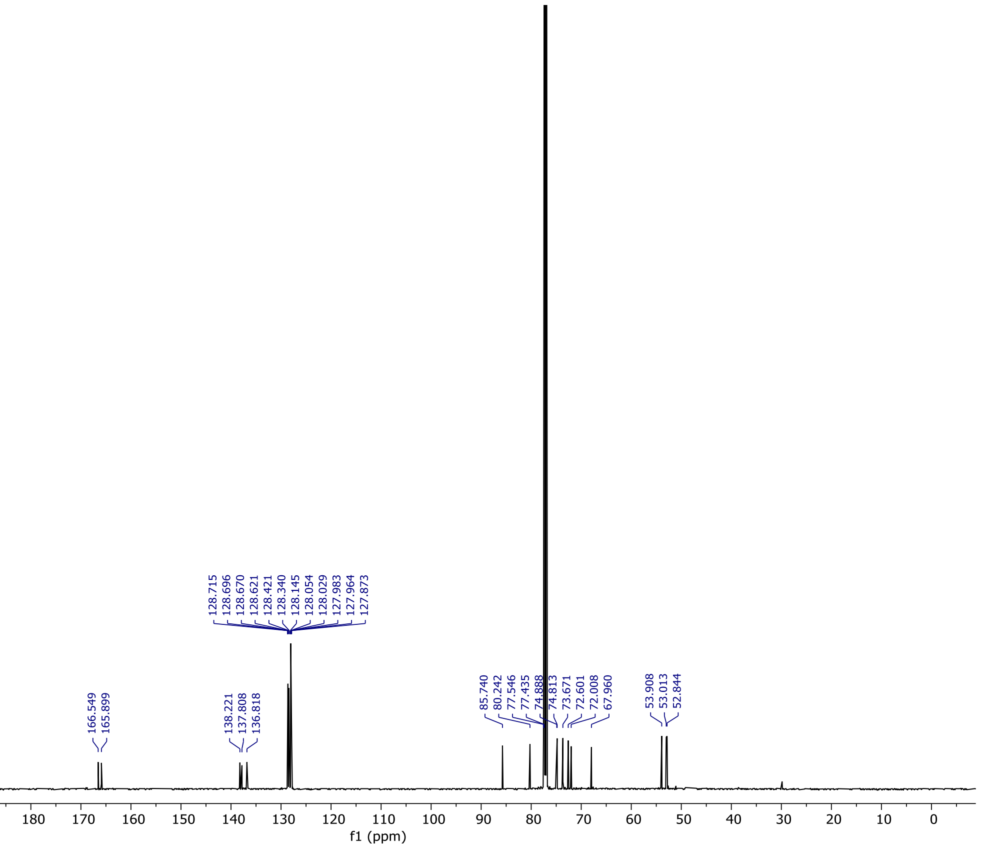

COSY NMR ($CDCl_3$, 400 MHz)

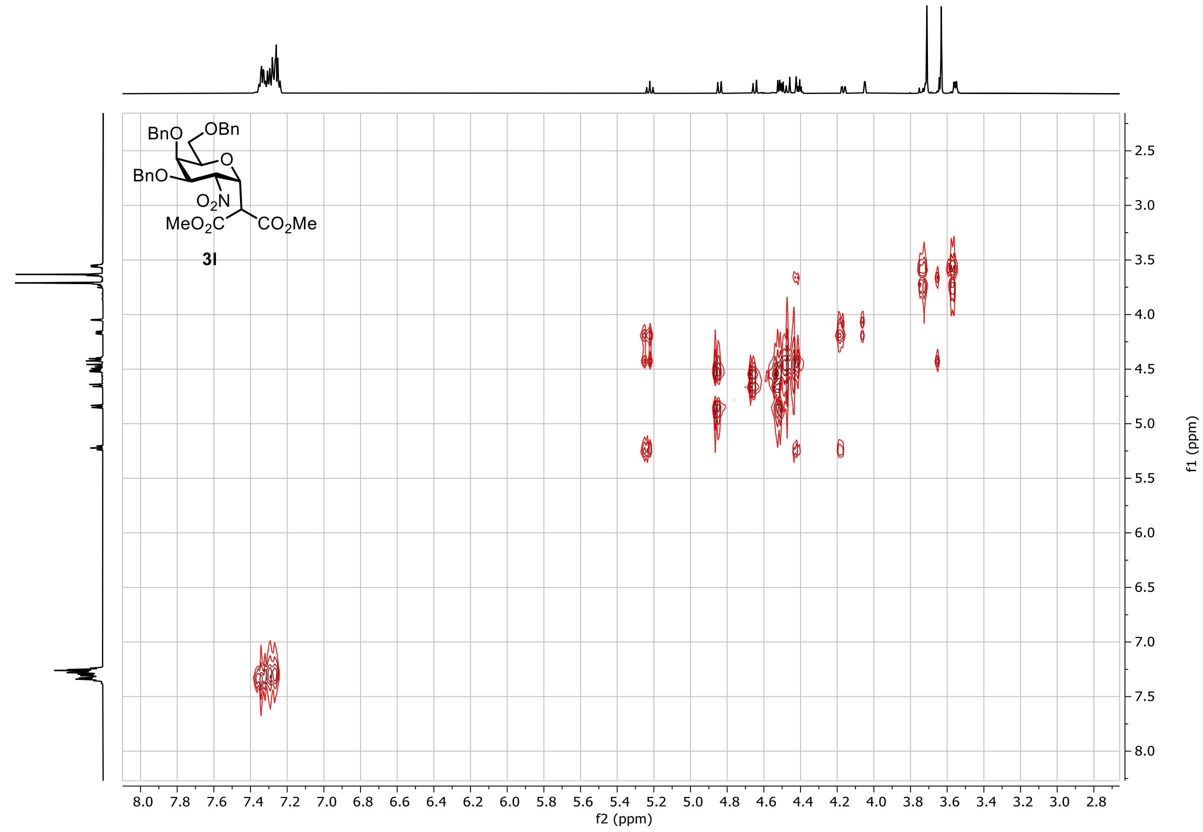

HSQC NMR ($CDCl_3$, 400 MHz)

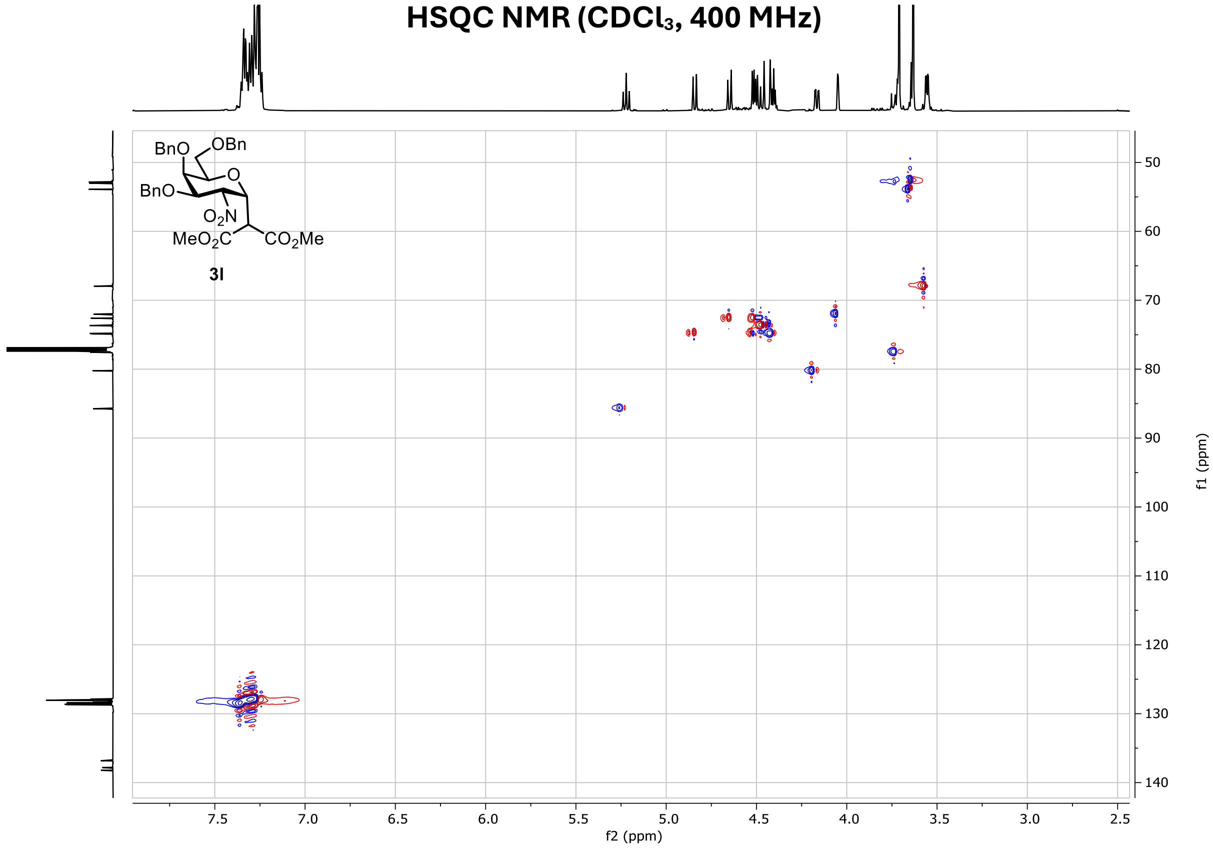

^{1}H NMR ($CDCl_3$, 400 MHz)

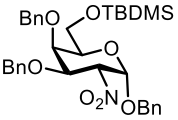

3m

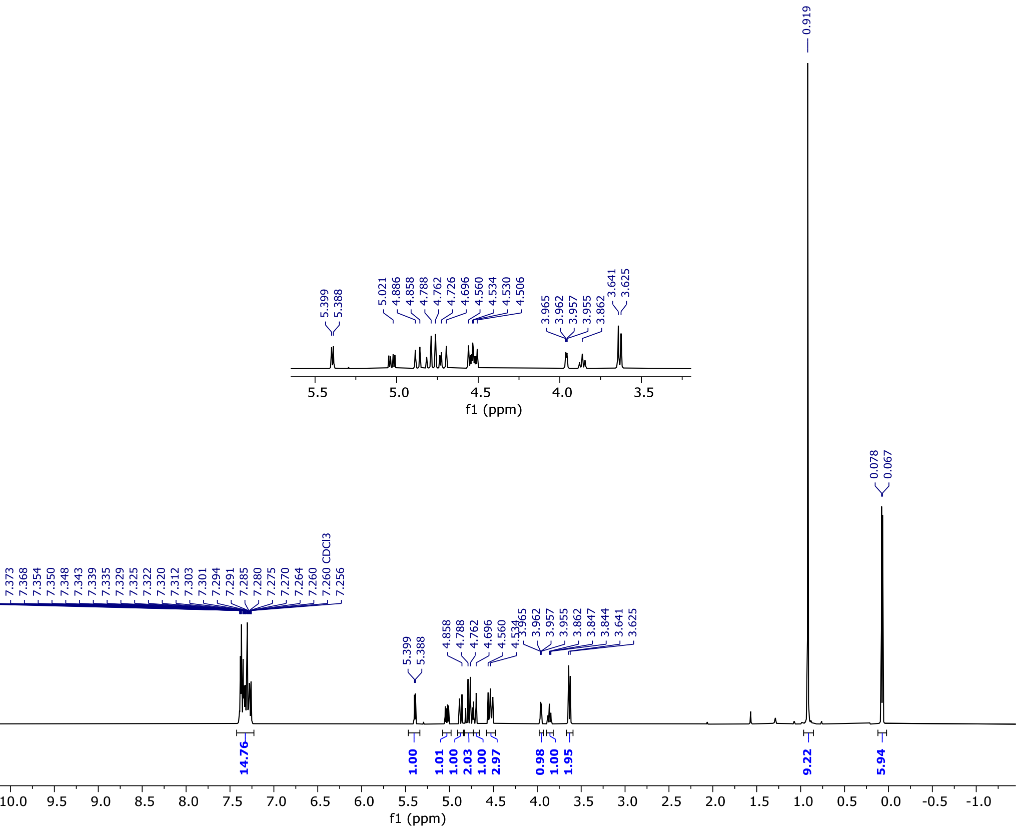

$^{13}C\{^1H\}$ NMR ($CDCl_3$, 101 MHz)

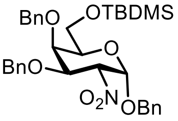

3m

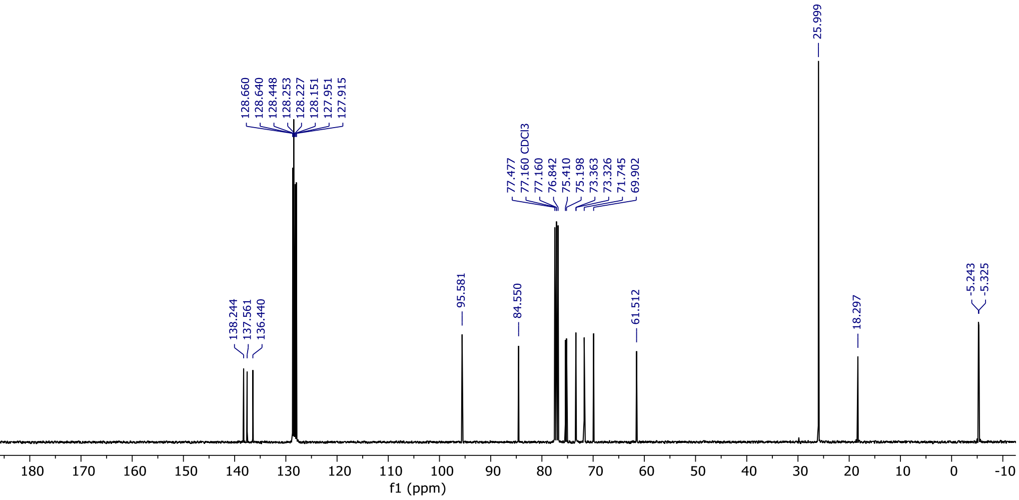

COSY NMR ($CDCl_3$, 400 MHz)

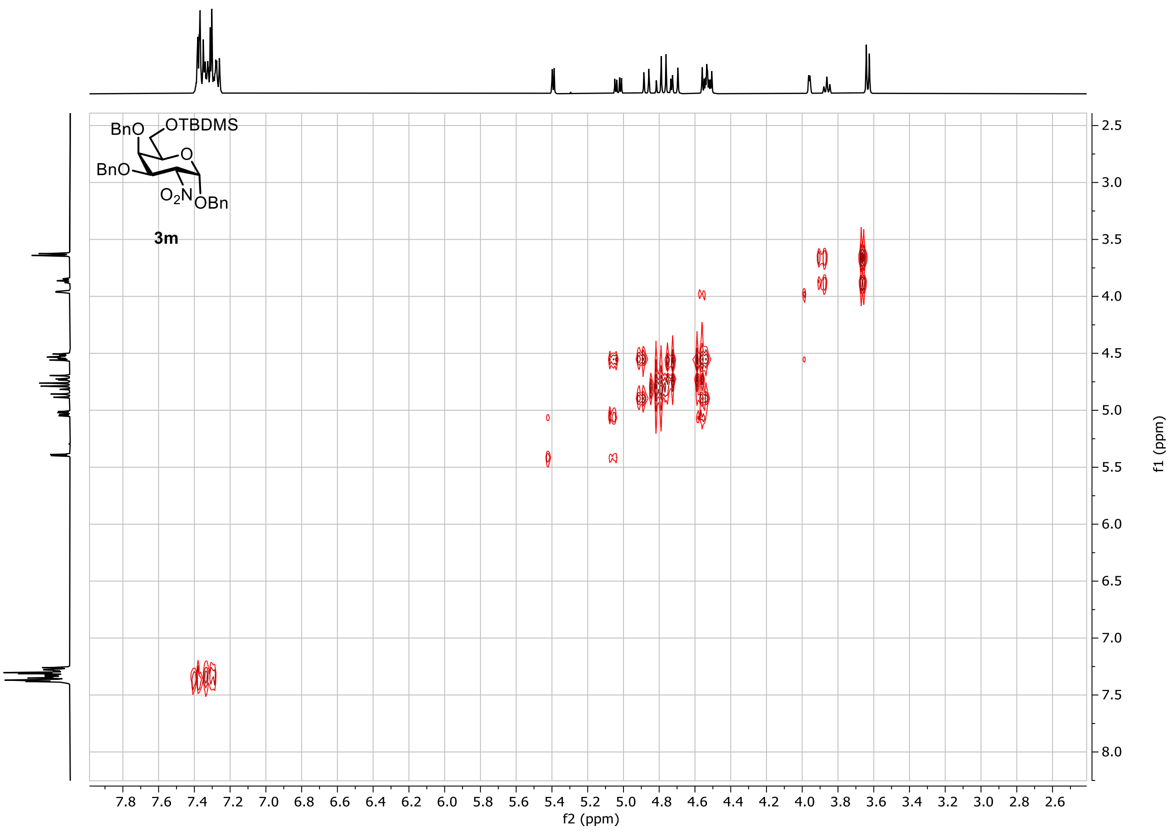

HSQC NMR ($CDCl_3$, 400 MHz)

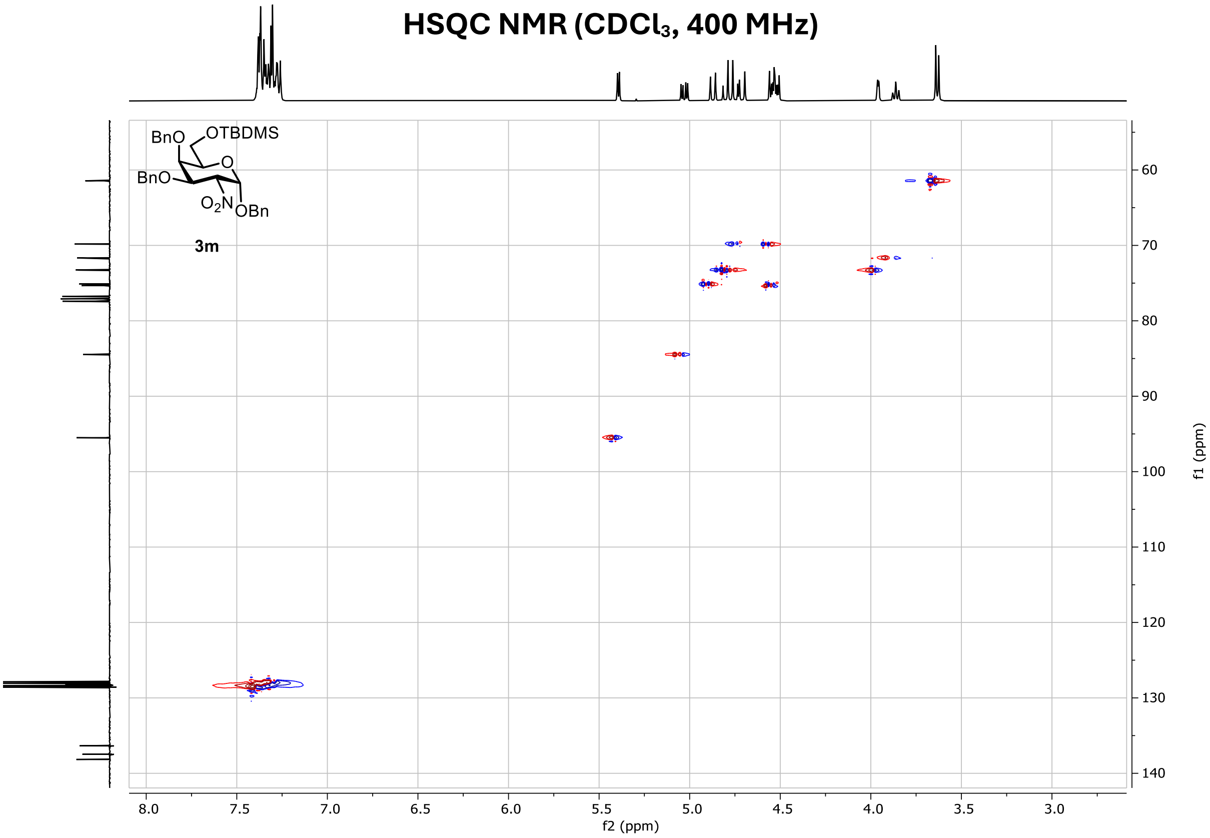

^{1}H NMR ($CDCl_3$, 400 MHz)

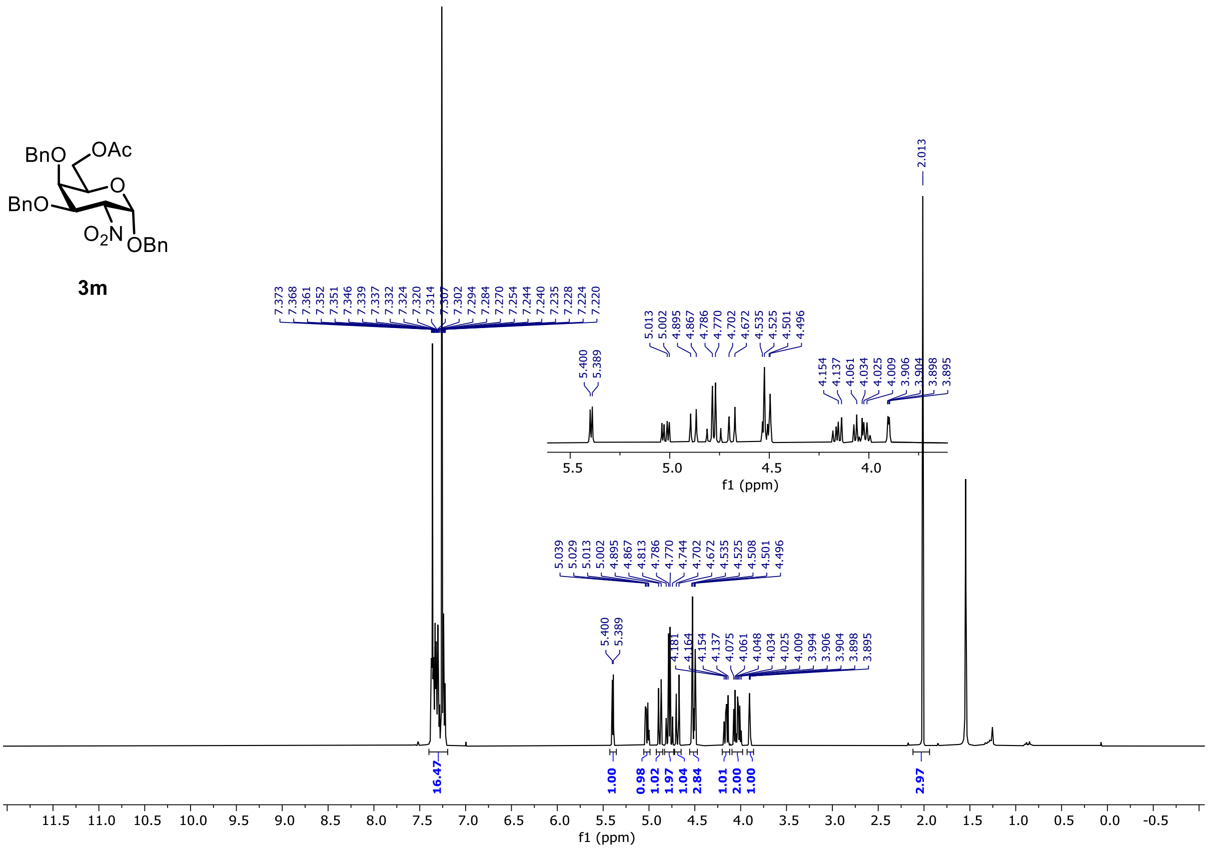

3m

^{13}C{^{1}H} NMR ($CDCl_3$, 101 MHz)

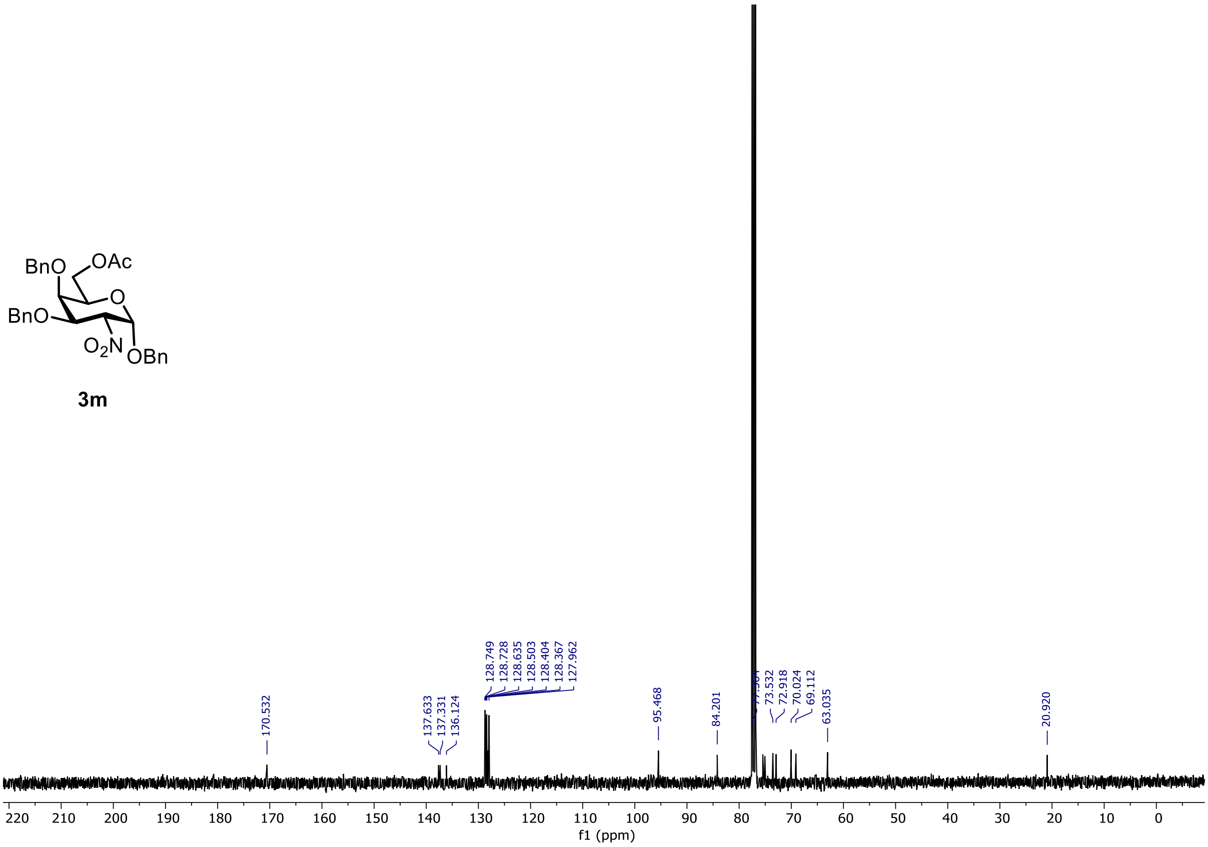

3m

^{1}H NMR ($CDCl_3$, 500 MHz)

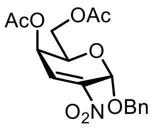

3o

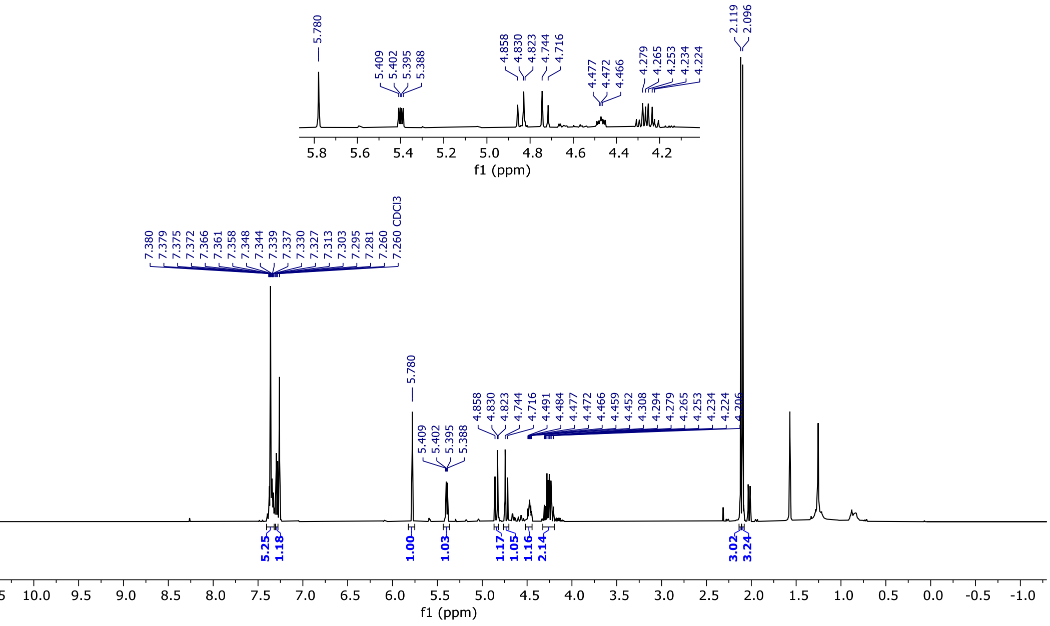

$^{13}C\{^{1}H\}$ NMR ($CDCl_3$, 125 MHz)

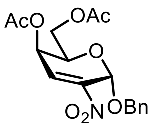

3o

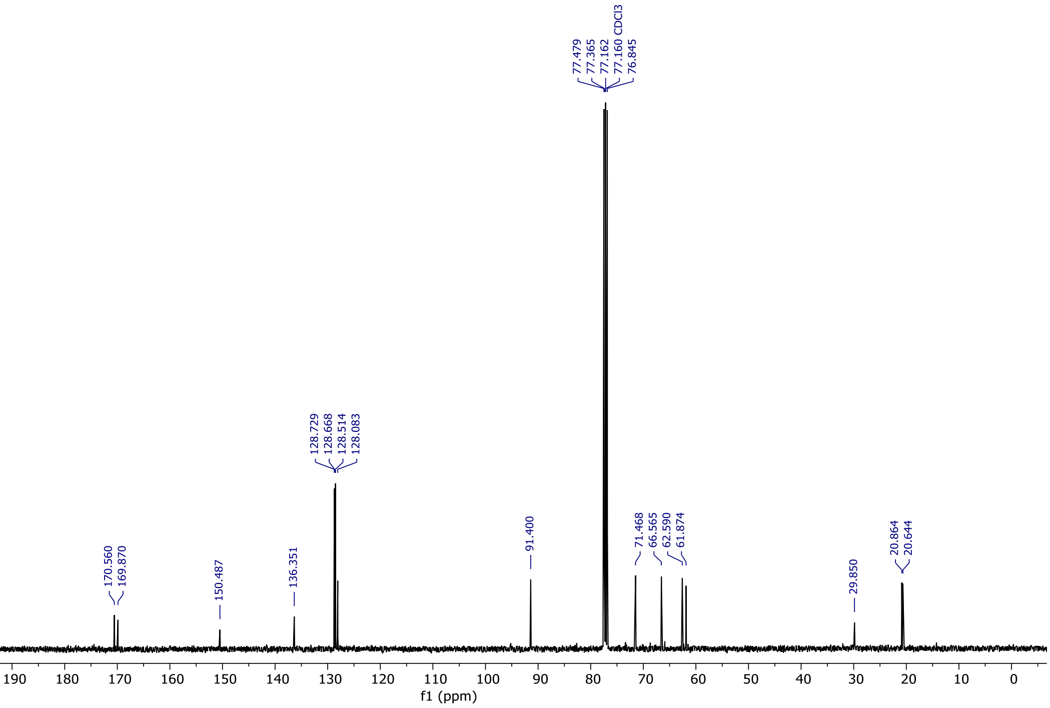

^{1}H NMR ($CDCl_3$, 400 MHz)

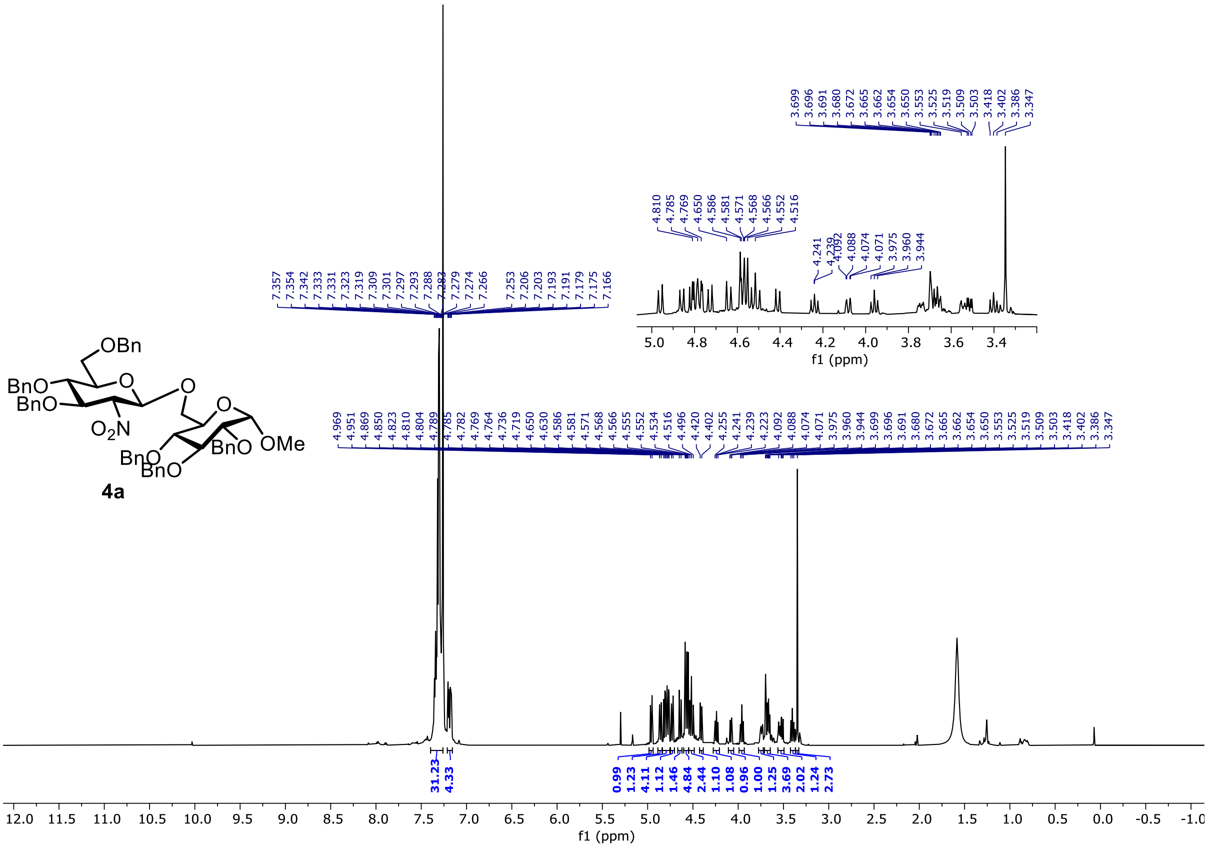

$^{13}C\{^{1}H\}$ NMR ($CDCl_3$, 101 MHz)

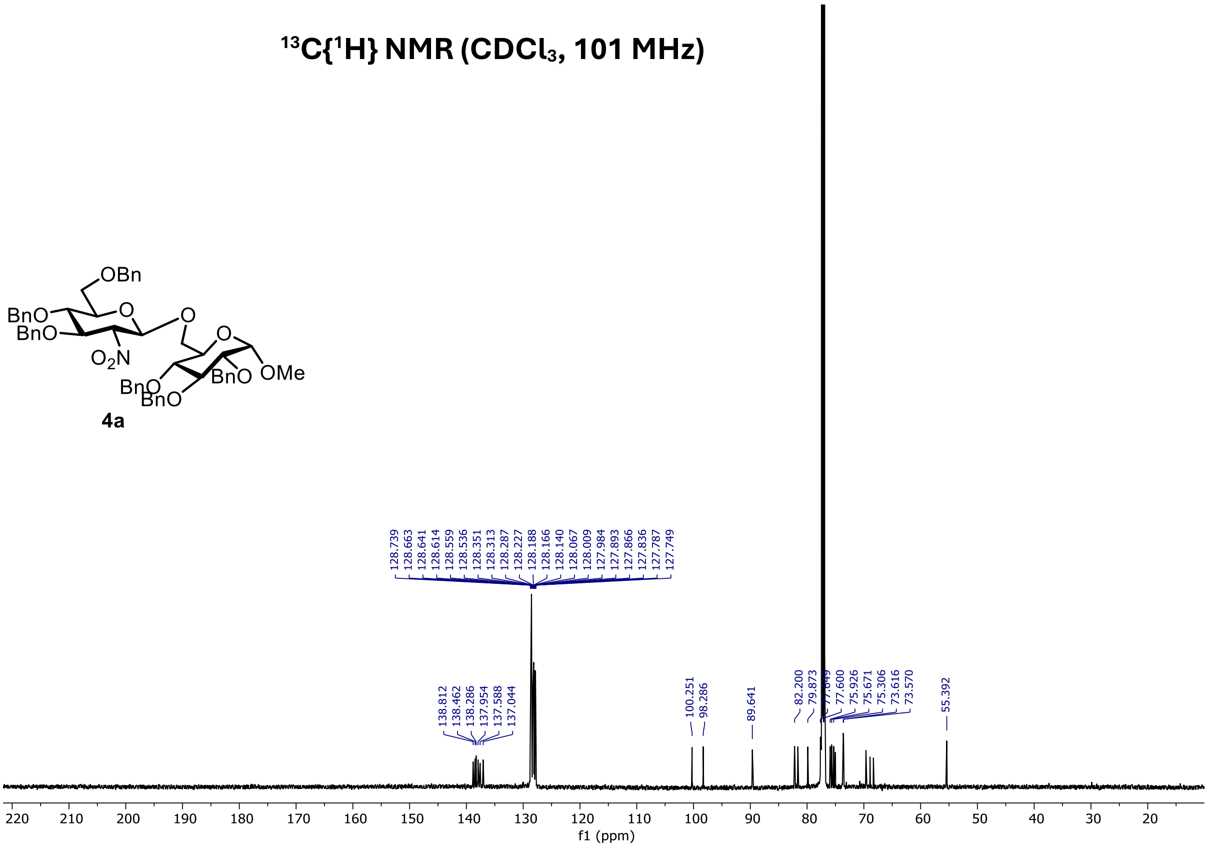

COSY NMR ($CDCl_3$, 400 MHz)

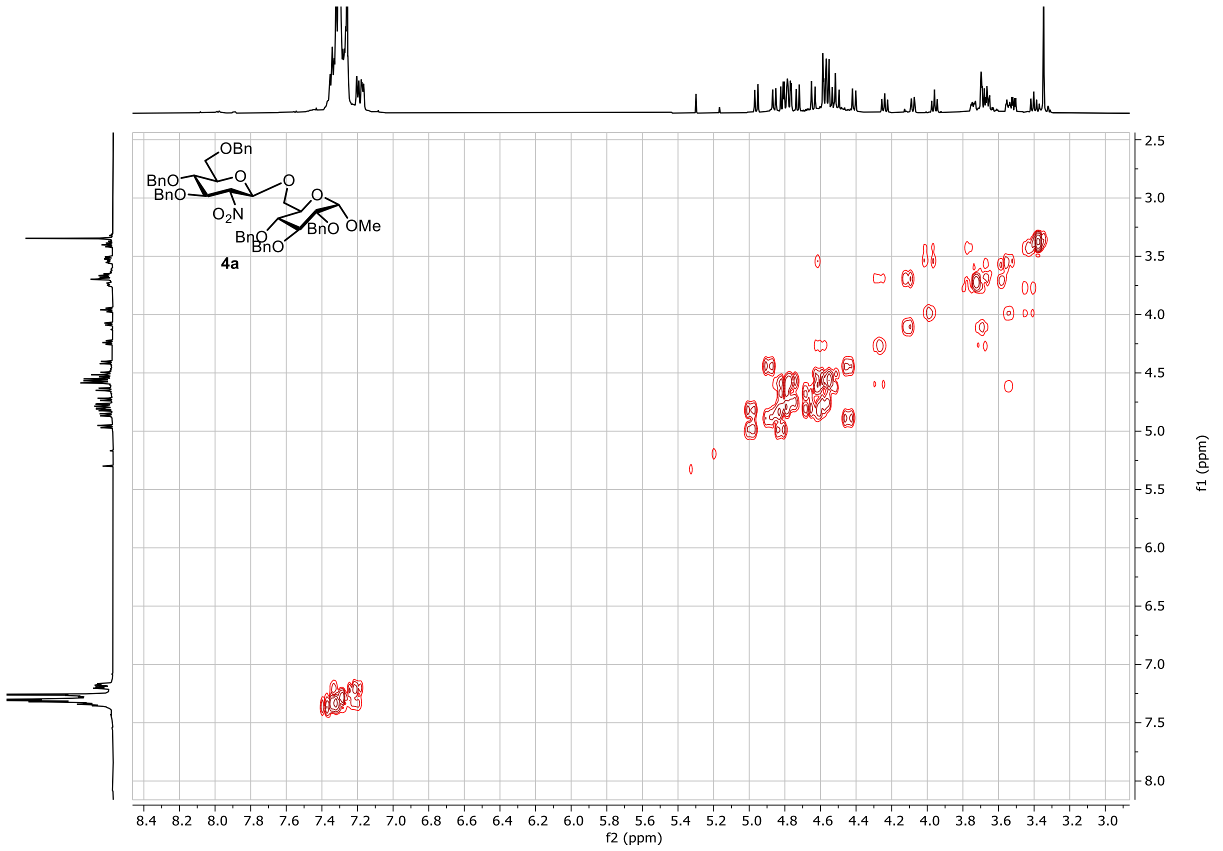

HSQC NMR ($CDCl_3$, 400 MHz)

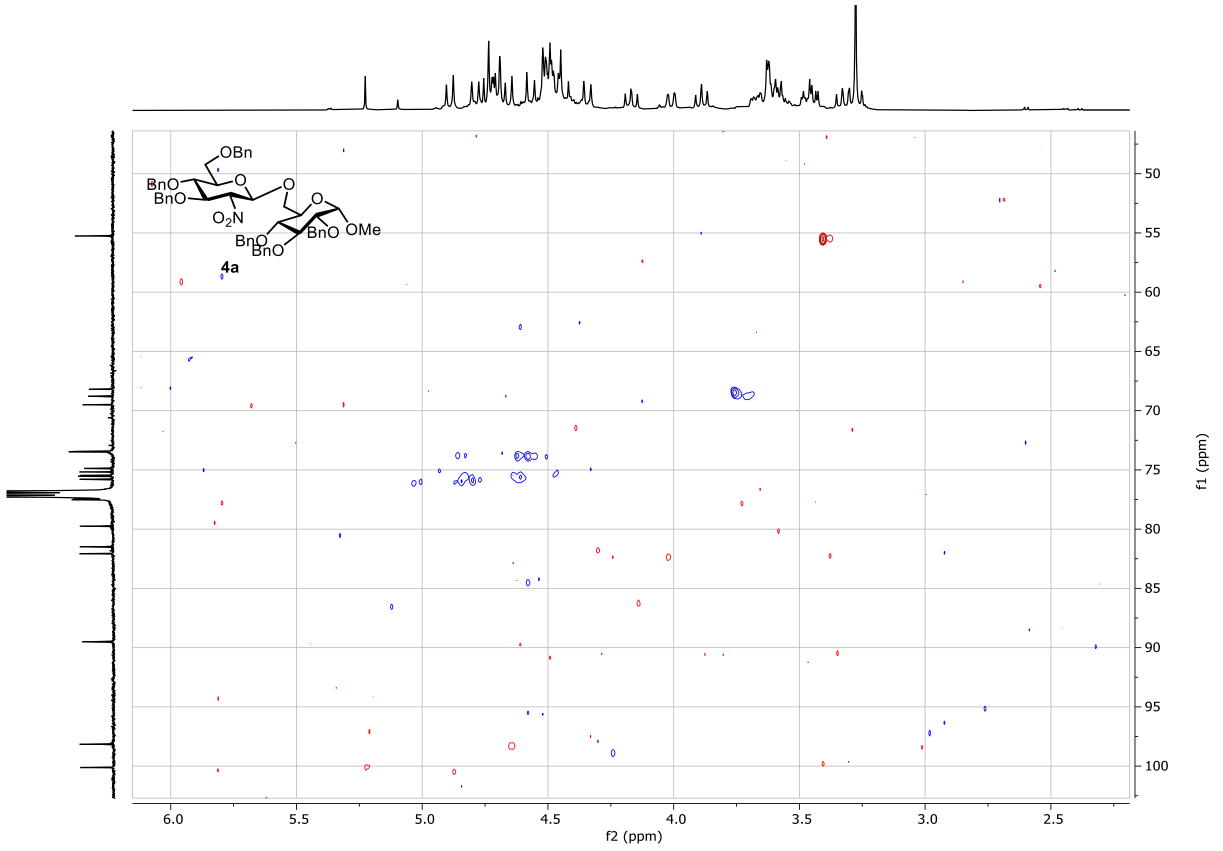

^{1}H NMR ($CDCl_3$, 400 MHz)

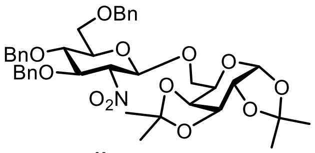

4b

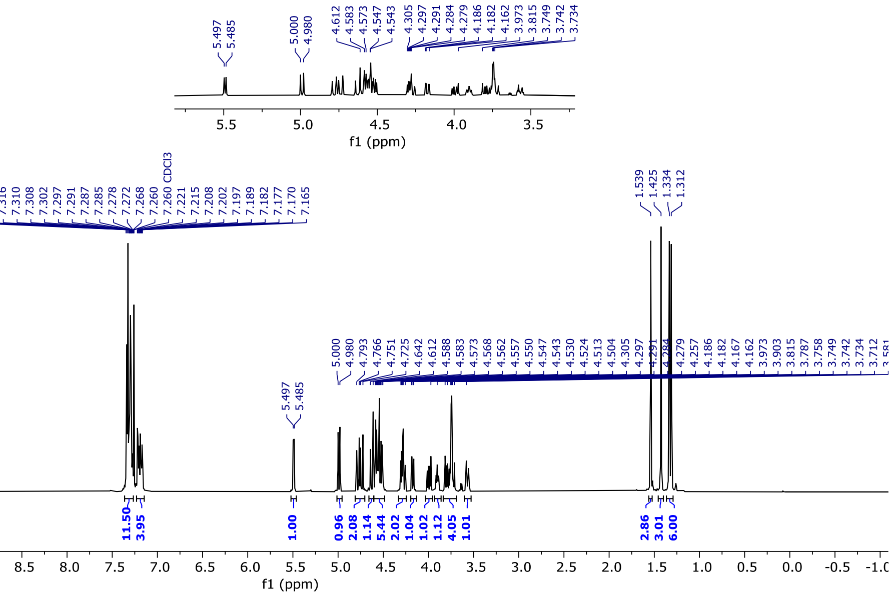

$^{13}C\{^{1}H\}$ NMR ($CDCl_3$, 101 MHz)

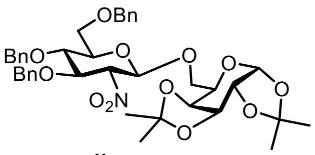

4b

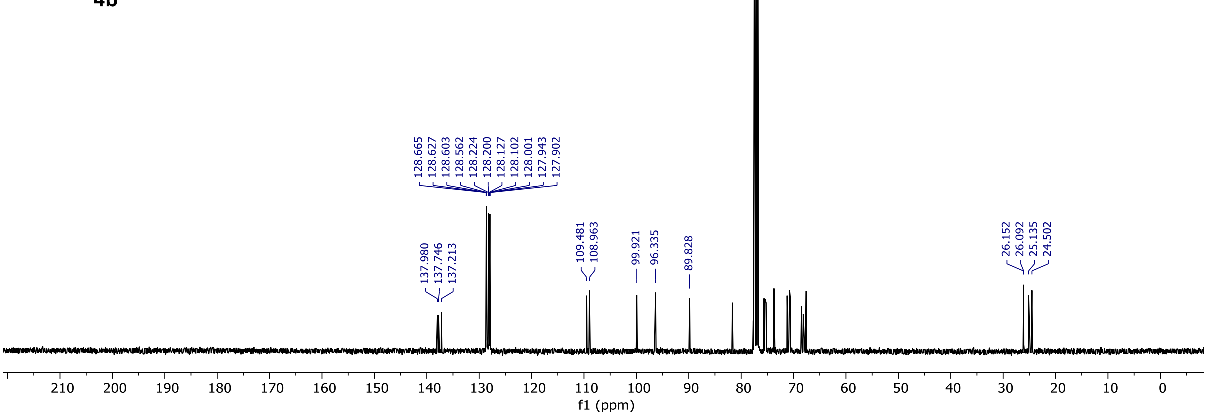

COSY NMR ($CDCl_3$, 400 MHz)

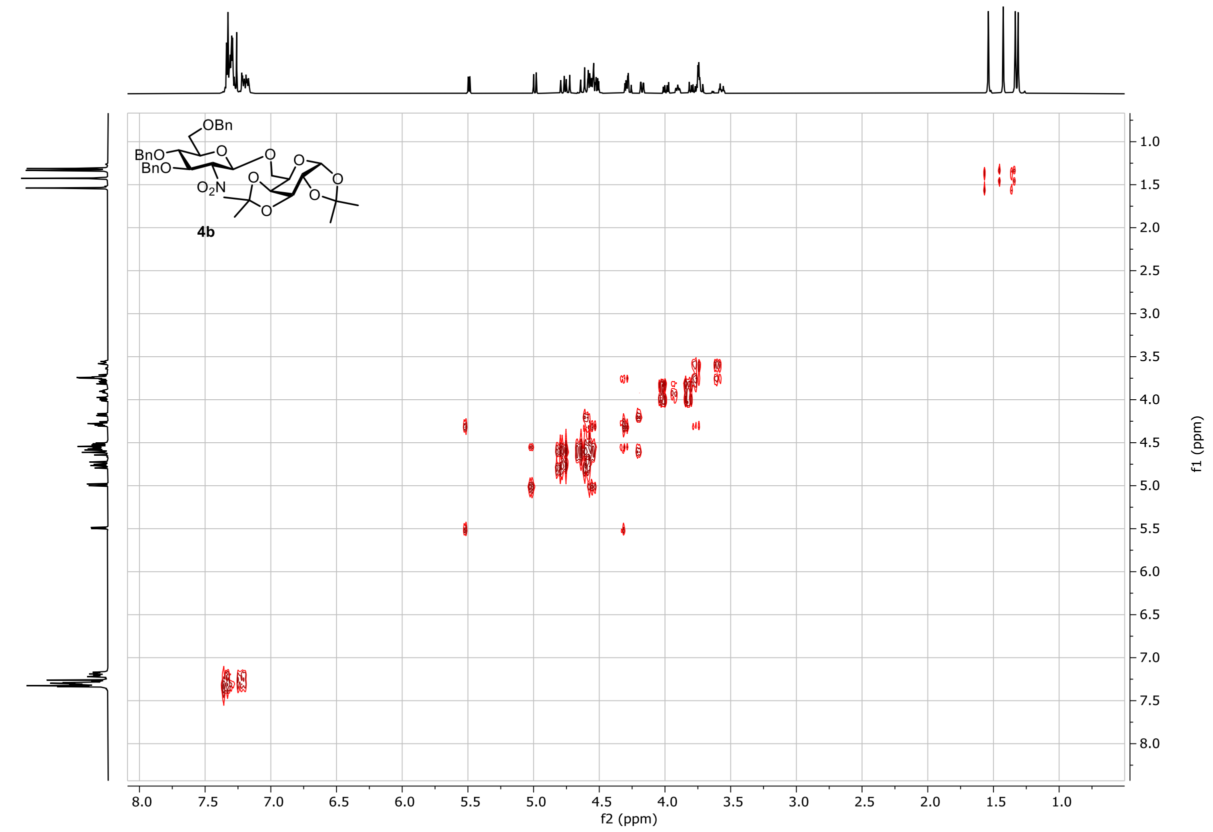

HSQC NMR ($CDCl_3$, 400 MHz)

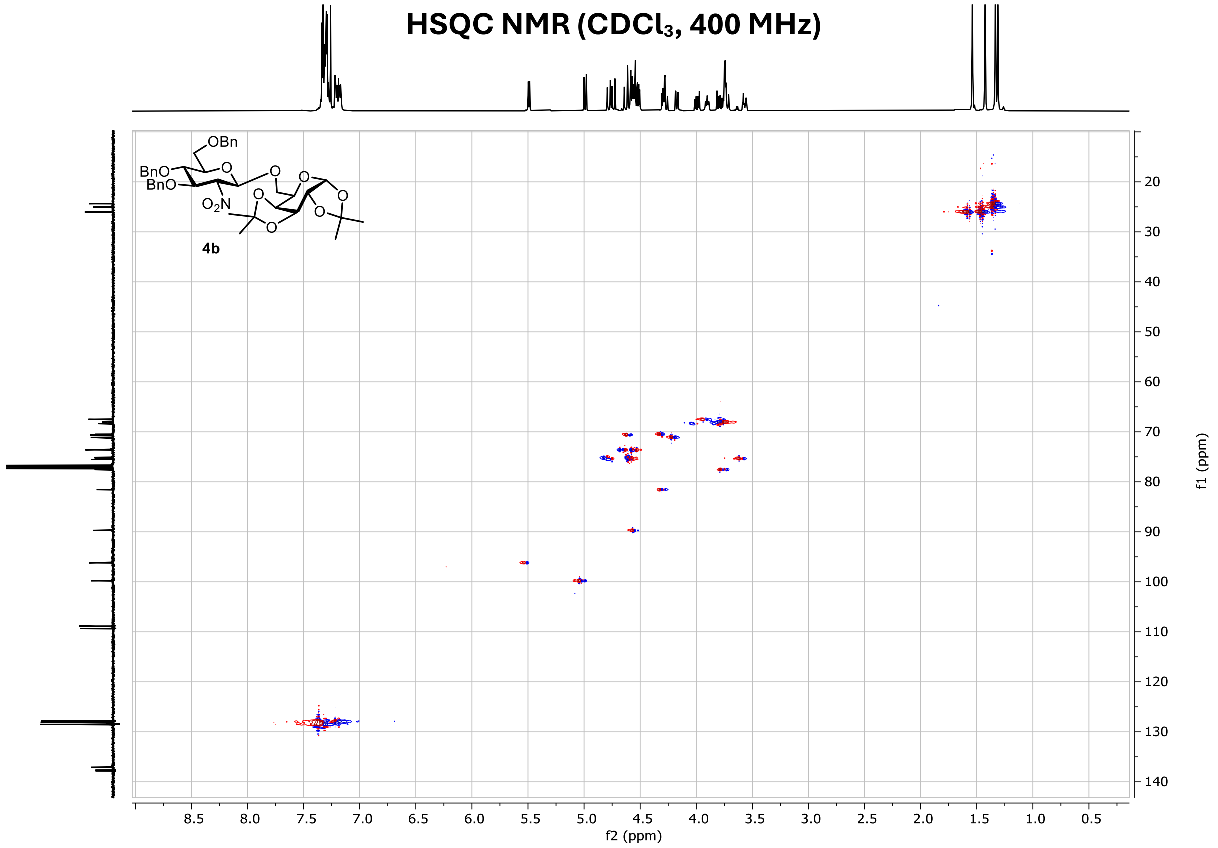

^{1}H NMR ($CDCl_3$, 400 MHz)

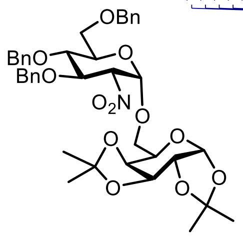

4b

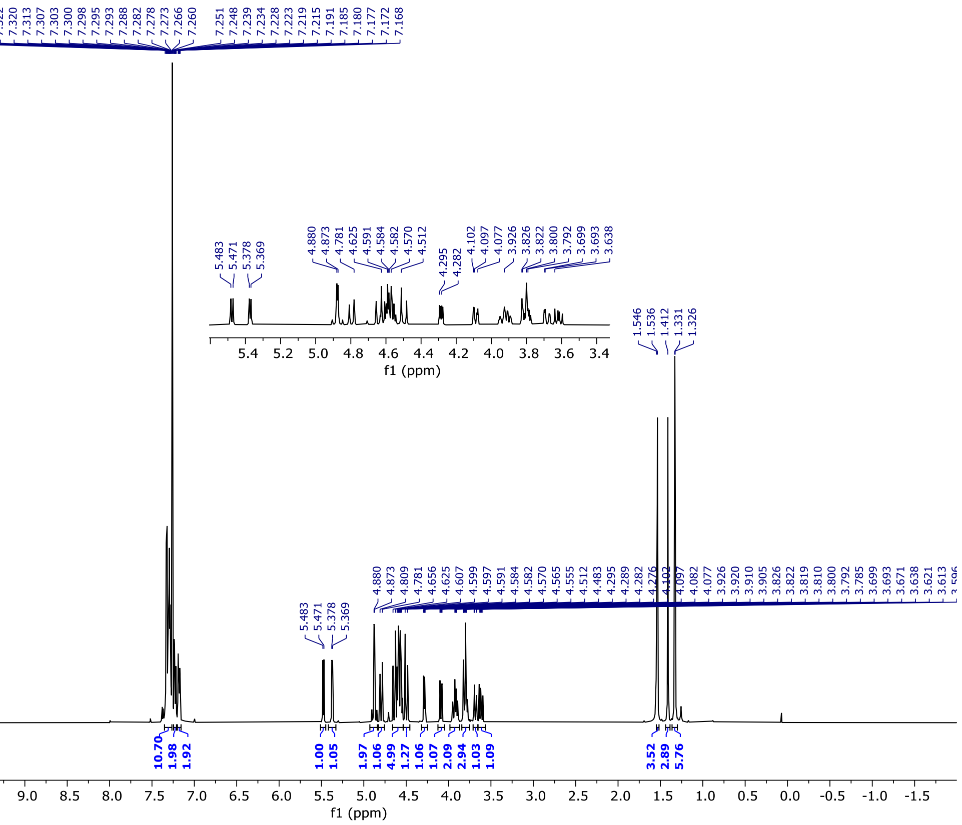

$^{13}C\{^{1}H\}$ NMR ($CDCl_3$, 101 MHz)

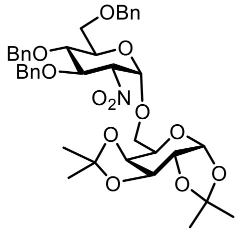

4b

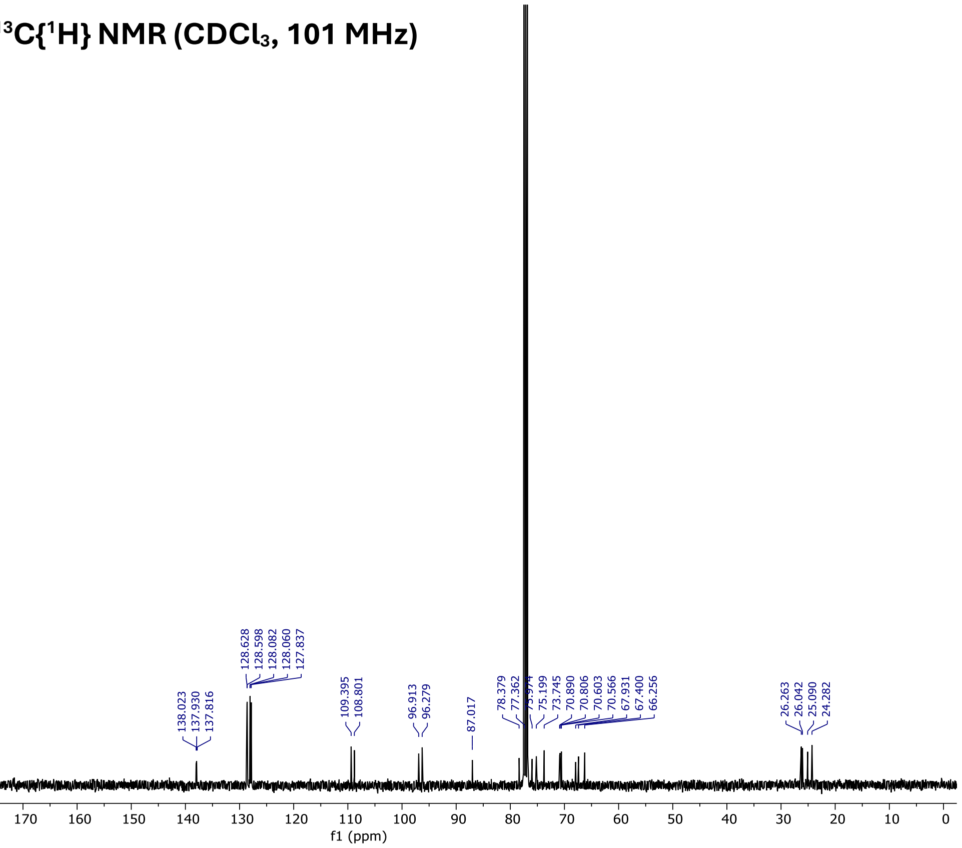

COSY NMR ($CDCl_3$, 400 MHz)

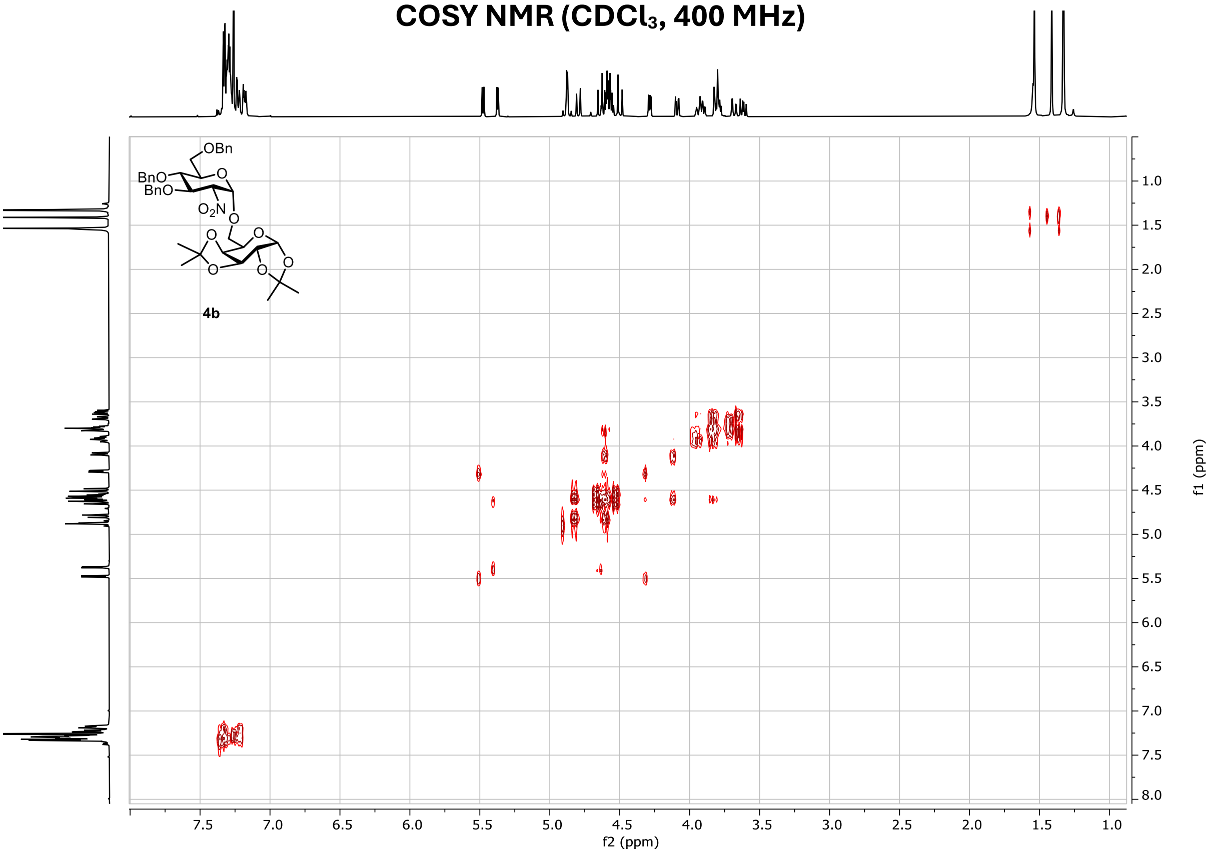

HSQC ($CDCl_3$, 400 MHz)

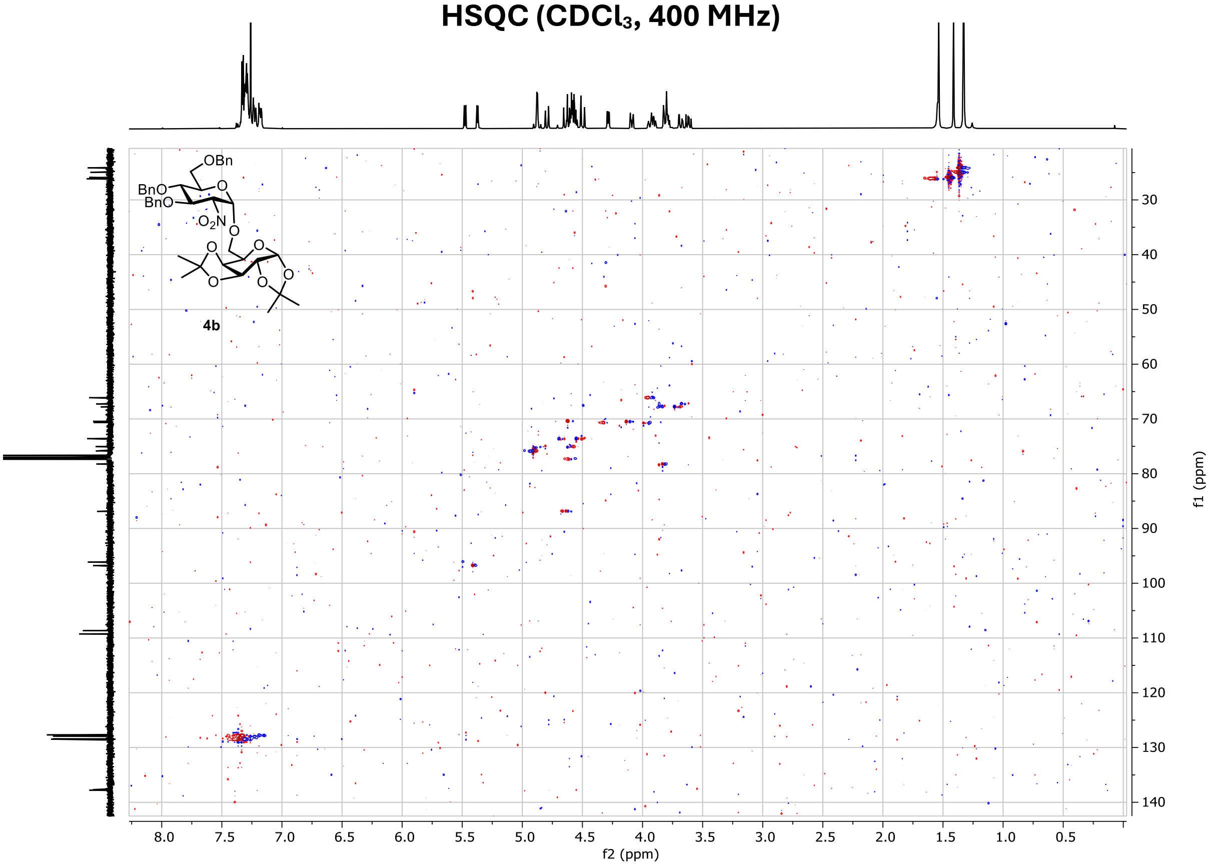

^{1}H NMR (CDCl$_3$, 400 MHz)

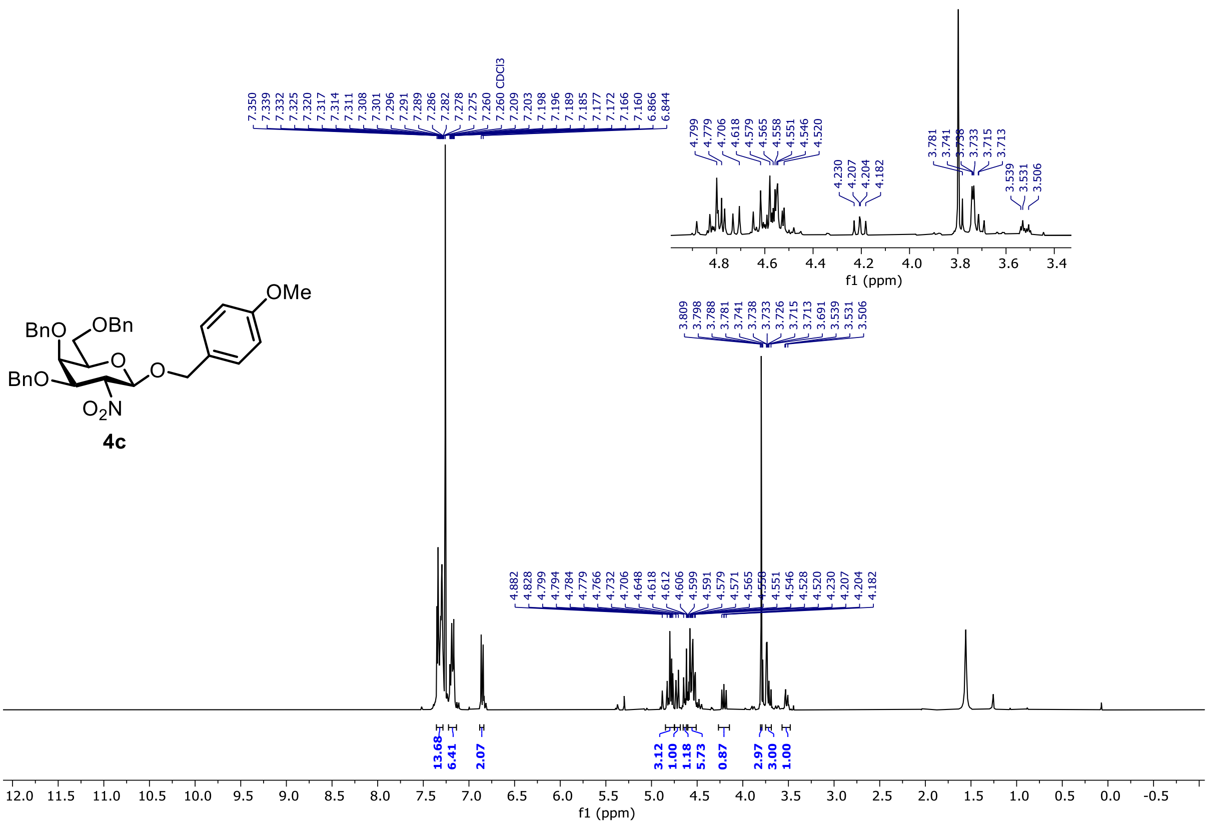

^{13}C{^{1}H} NMR (CDCl$_3$, 101 MHz)

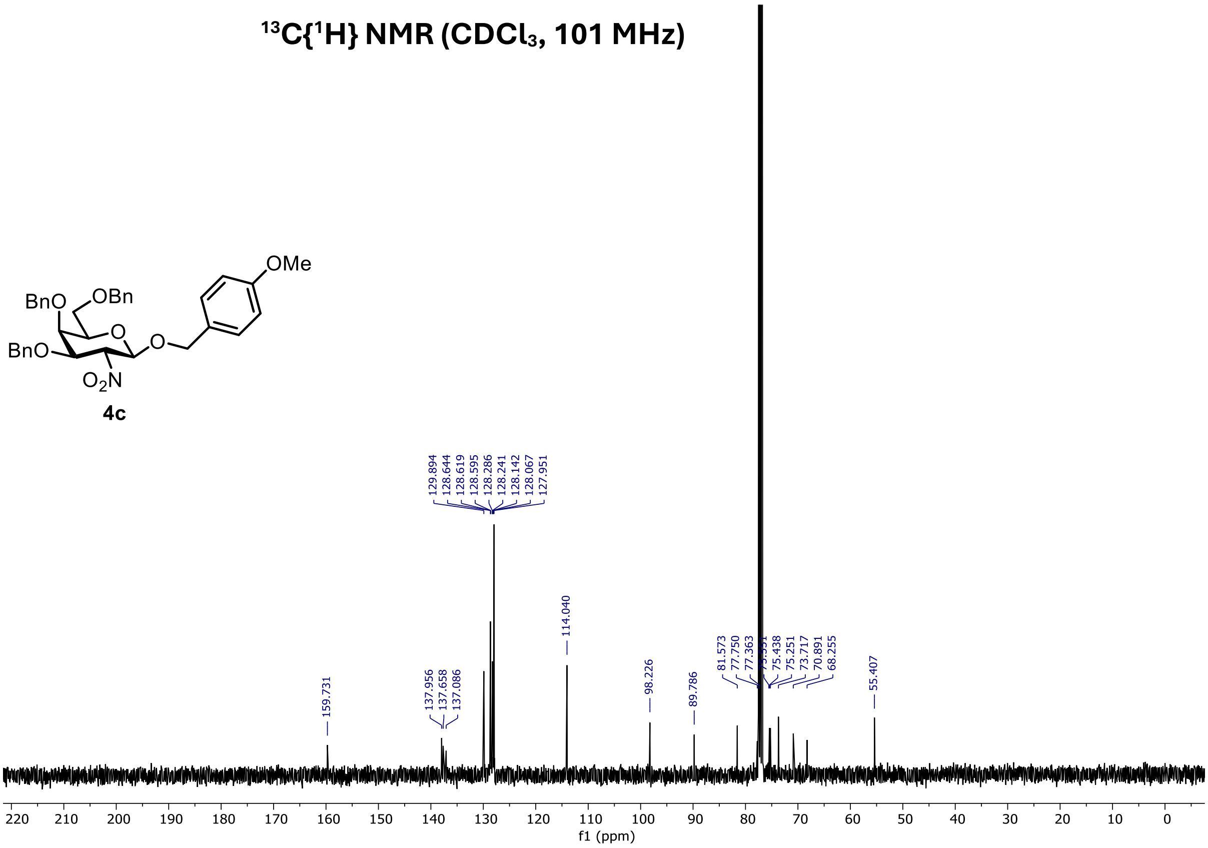

COSY NMR ($CDCl_3$, 400 MHz)

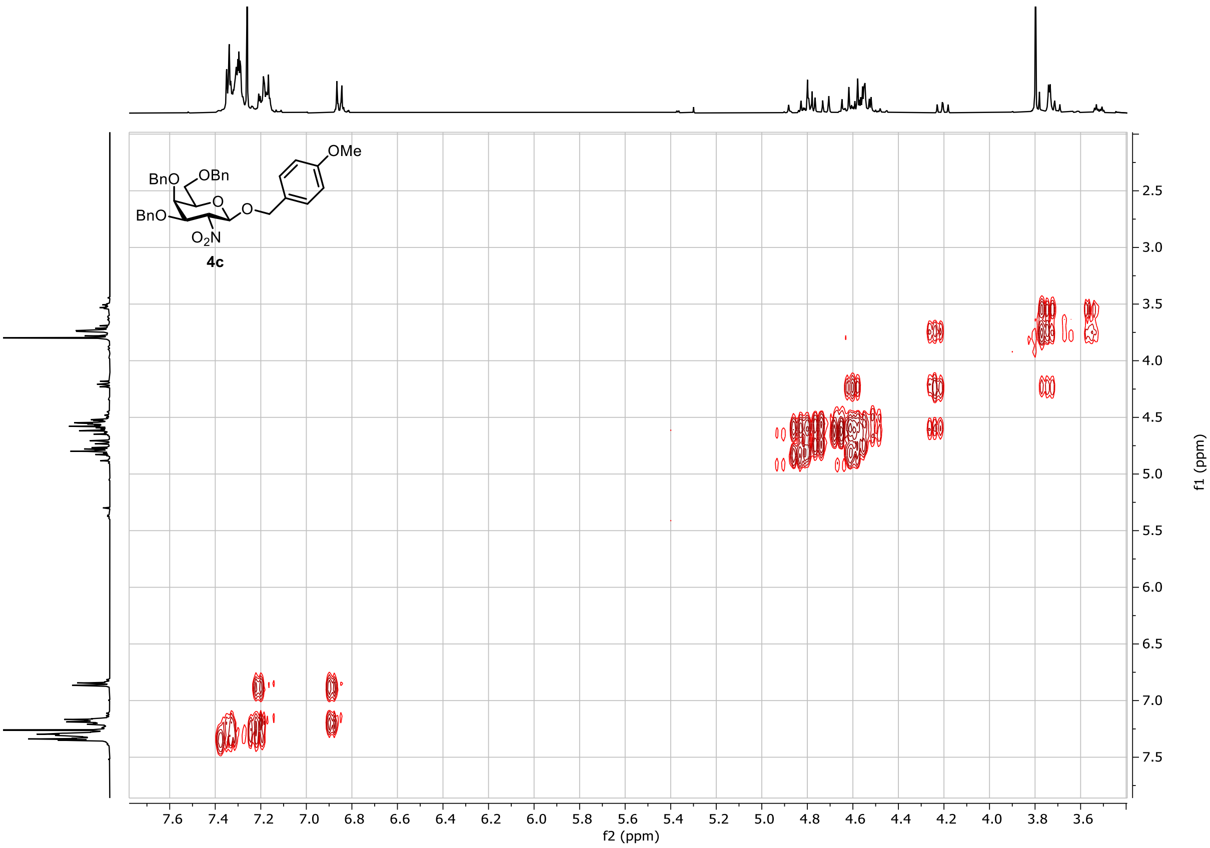

HSQC NMR ($CDCl_3$, 400 MHz)

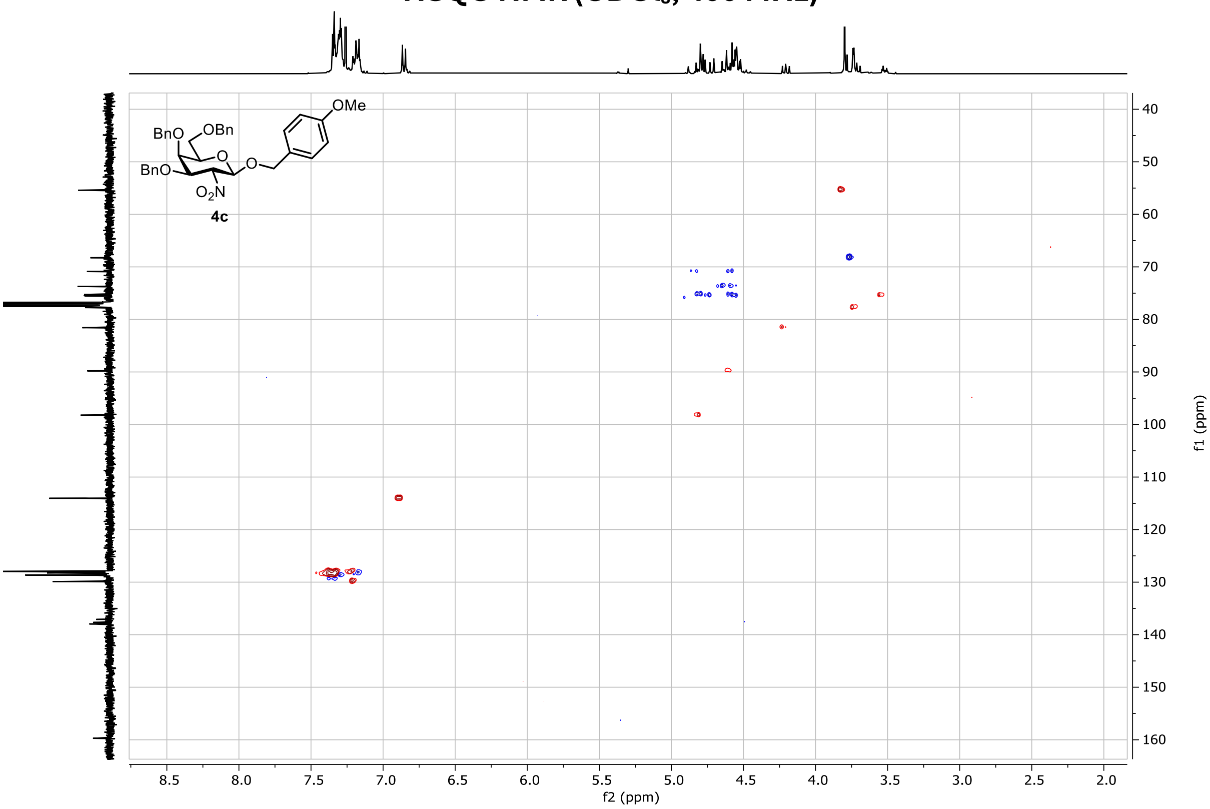

^{1}H NMR ($CDCl_3$, 400 MHz)

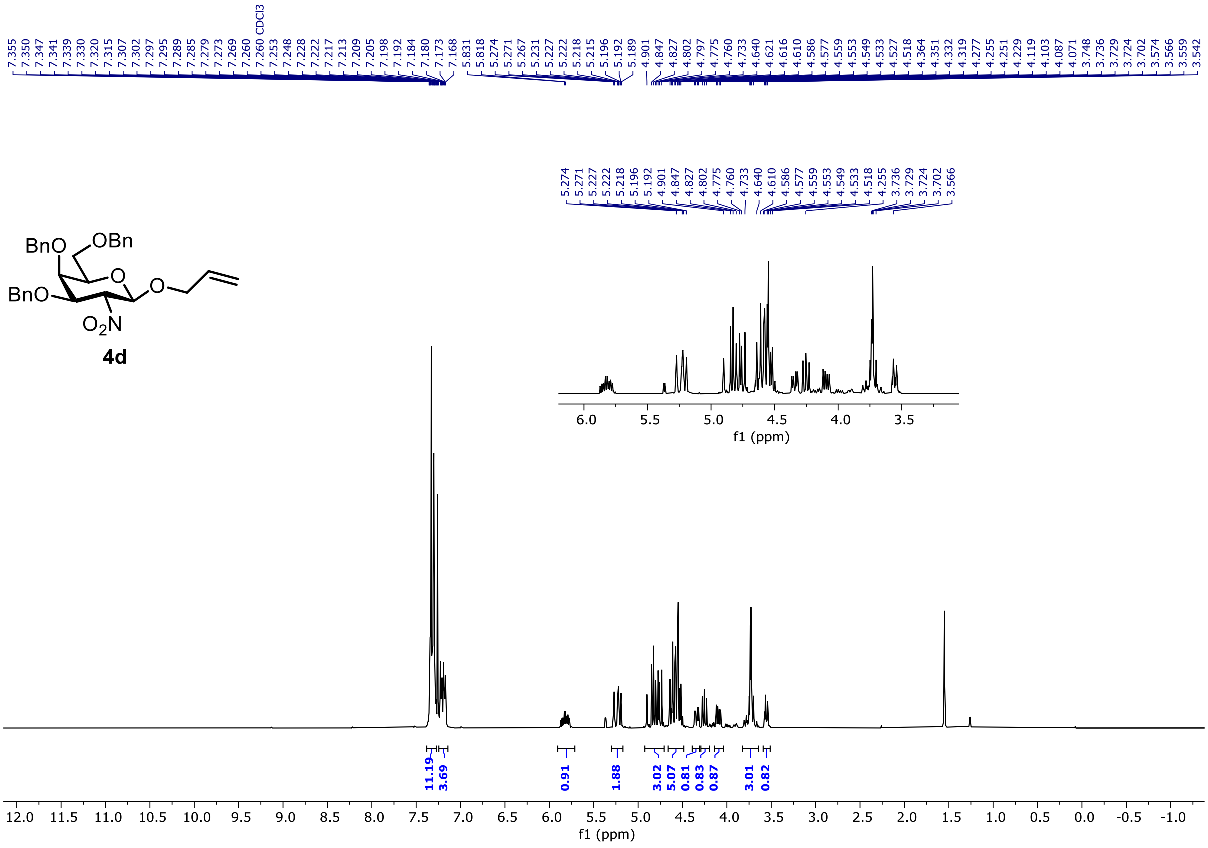

$^{13}C\{^{1}H\}$ NMR ($CDCl_3$, 101 MHz)

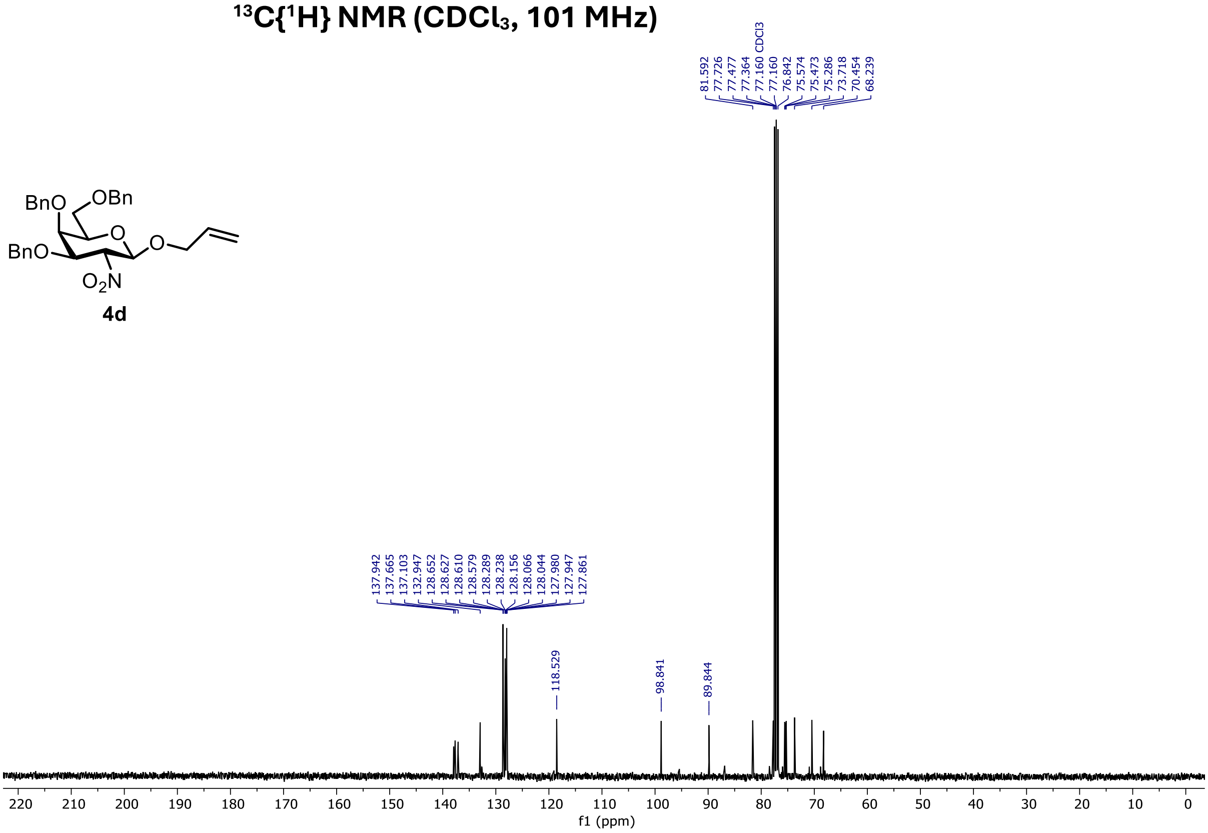

COSY ($CDCl_3$, 400 MHz)

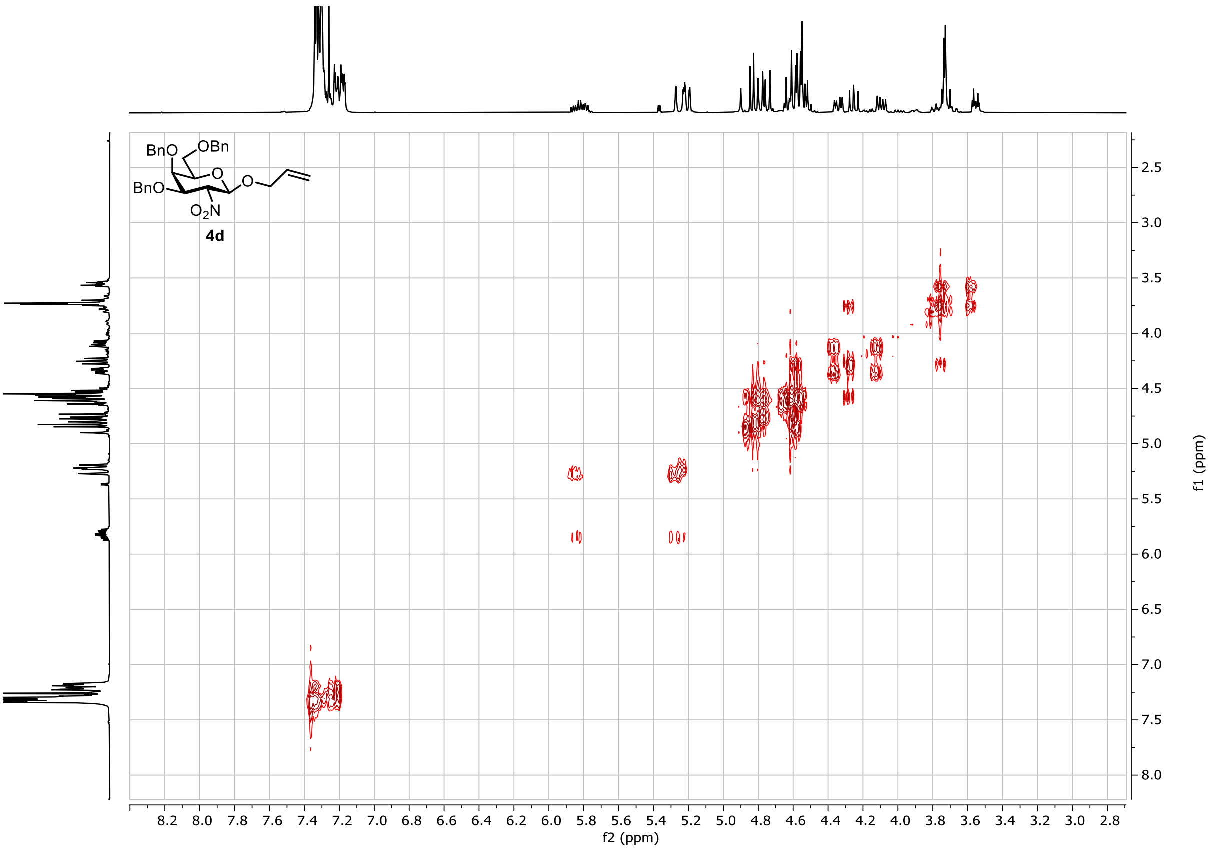

HSQC ($CDCl_3$, 400 MHz)

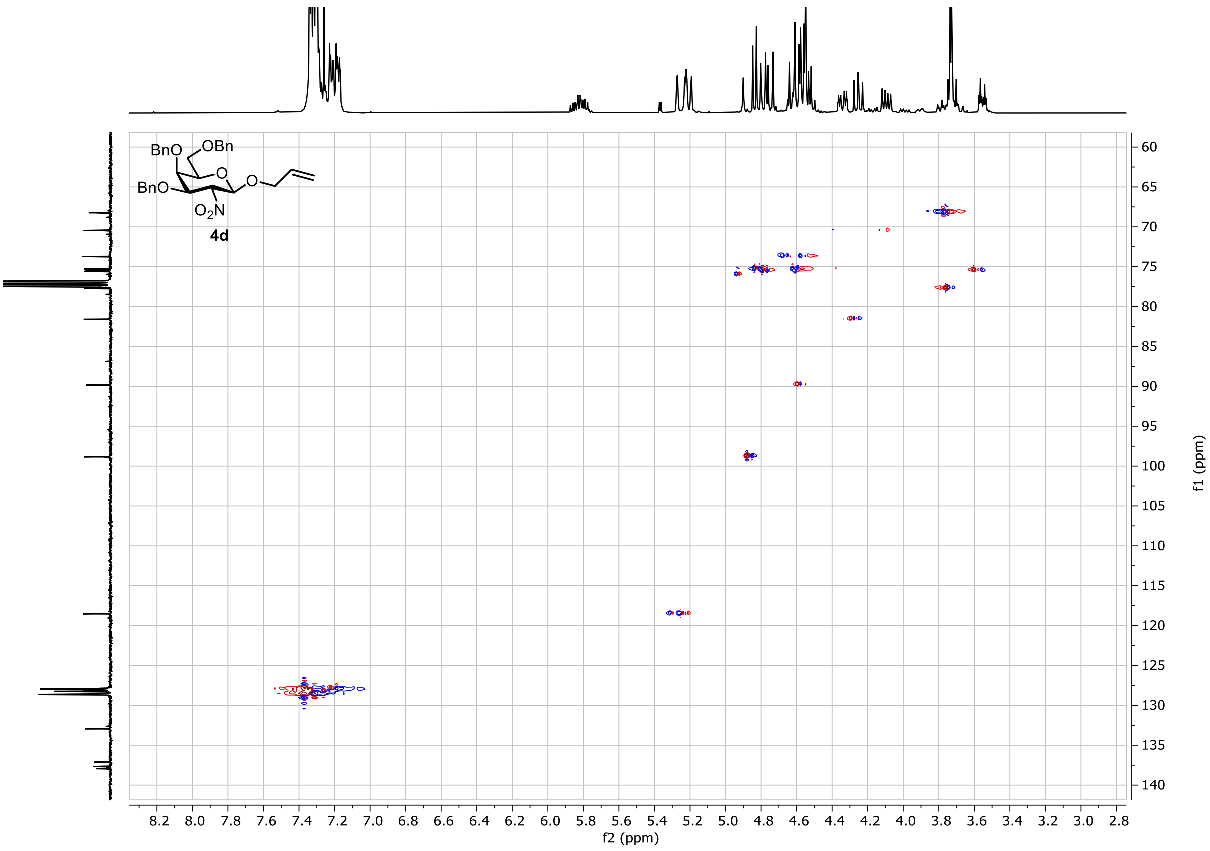

^1H NMR ($CDCl_3$, 400 MHz)

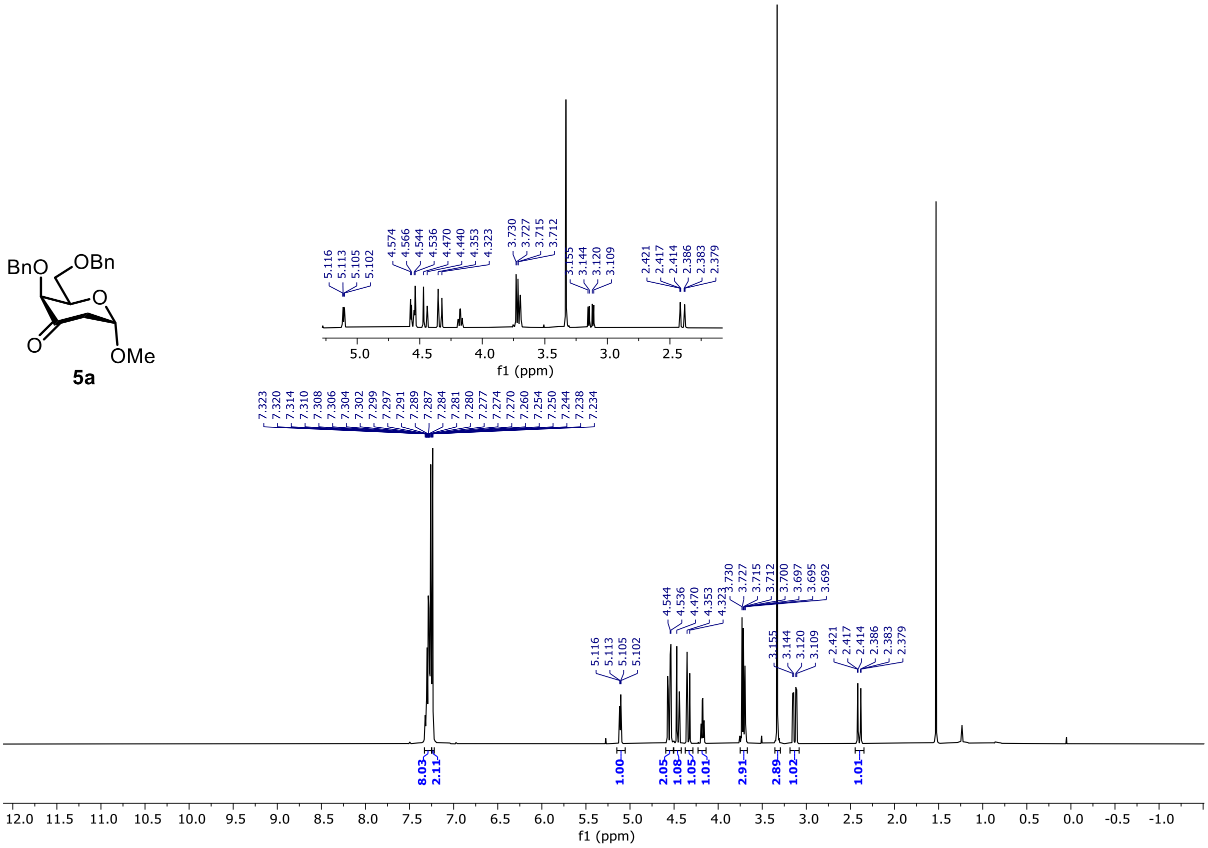

$^{13}C\{^1H\}$ NMR ($CDCl_3$, 101 MHz)

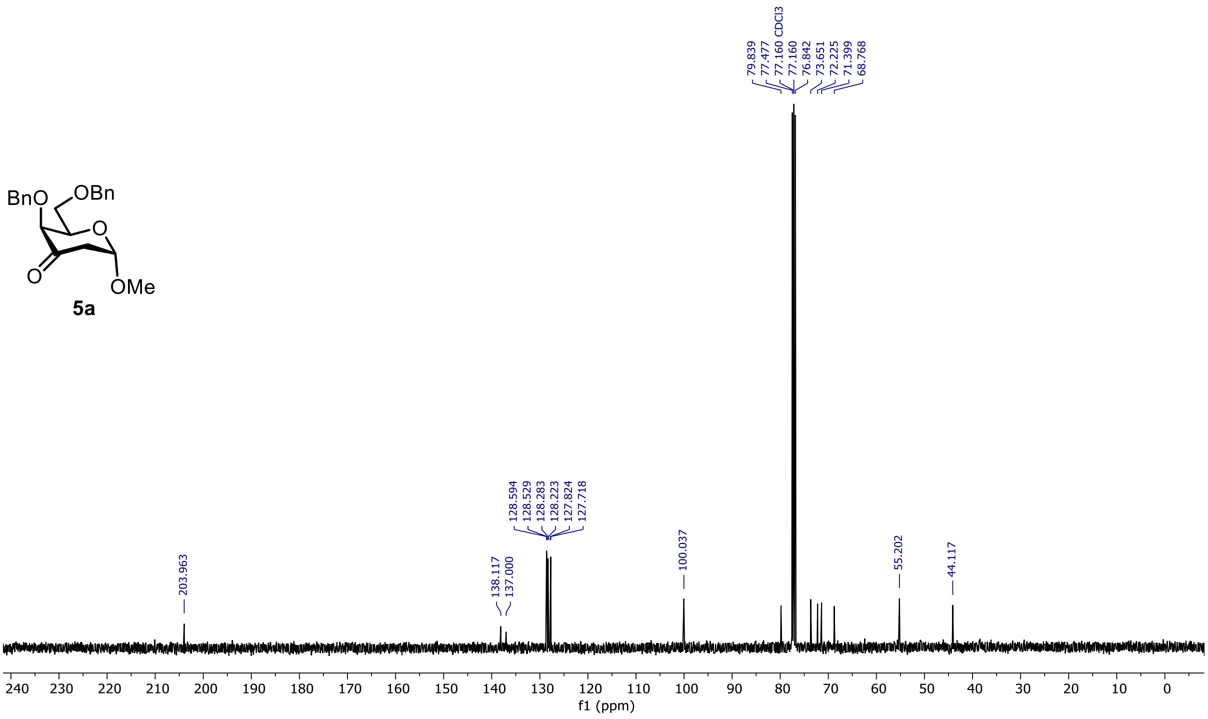

COSY NMR ($CDCl_3$, 400 MHz)

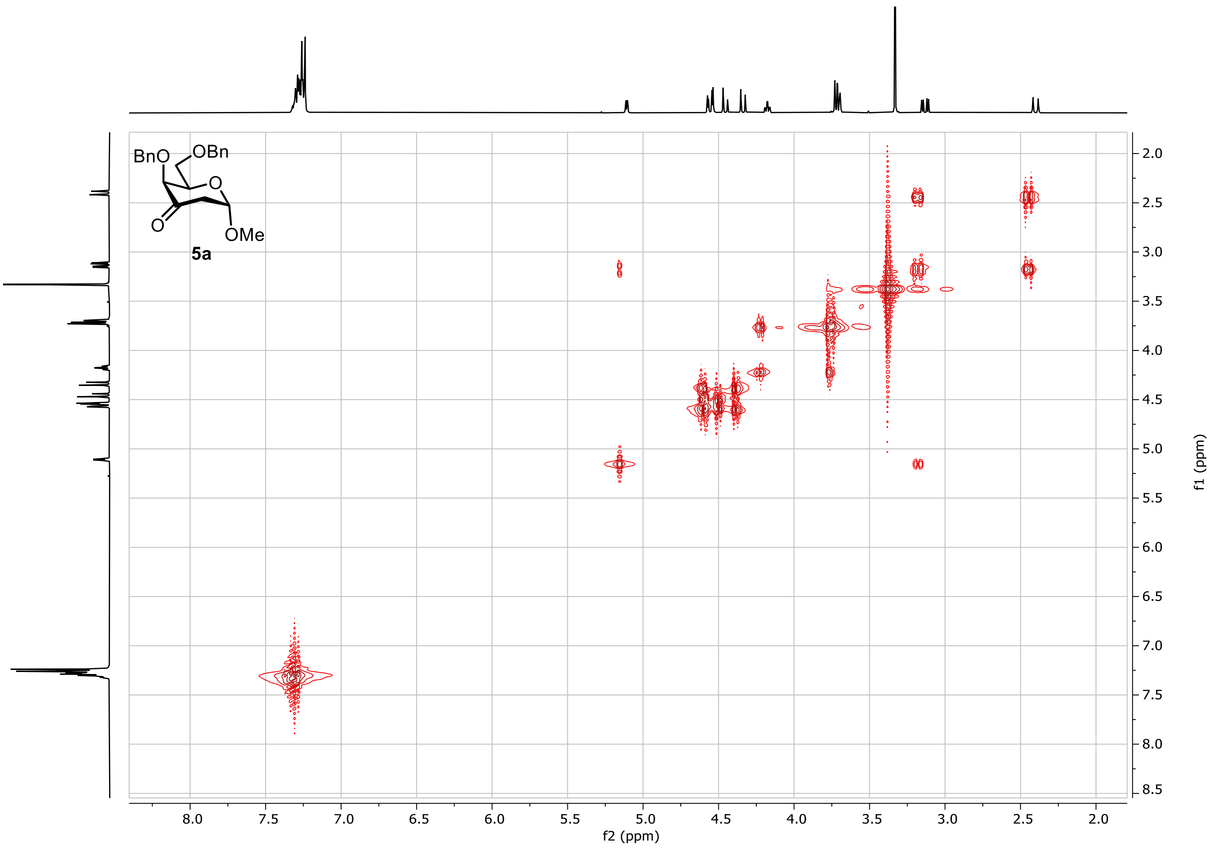

HSQC NMR ($CDCl_3$, 400 MHz)

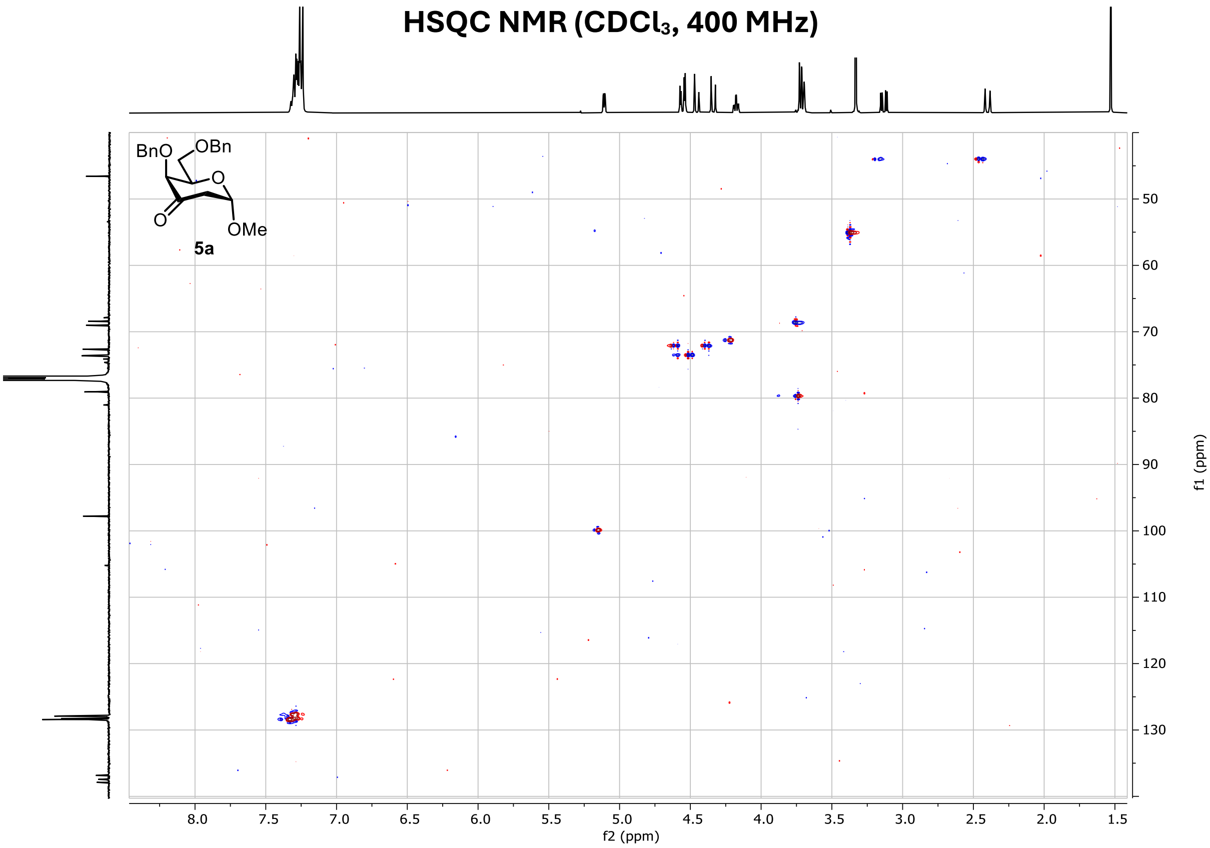

^{1}H NMR ($CDCl_3$, 400 MHz)

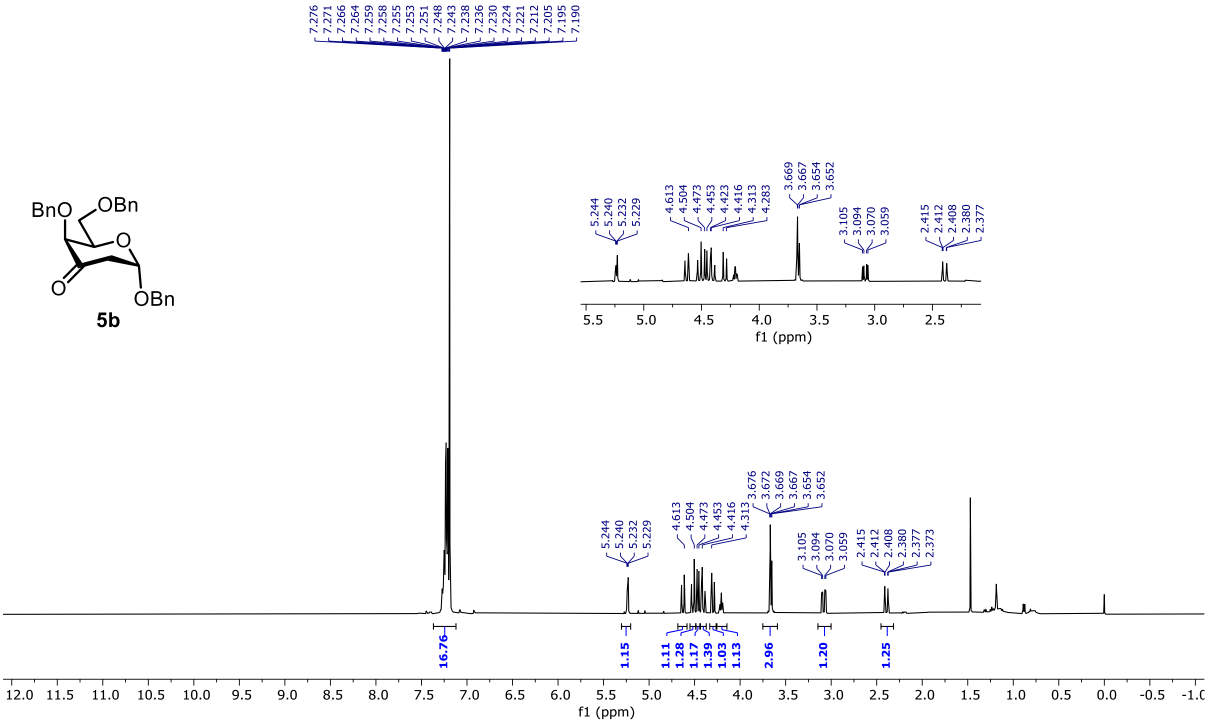

^{13}C{^{1}H} NMR ($CDCl_3$, 101 MHz)

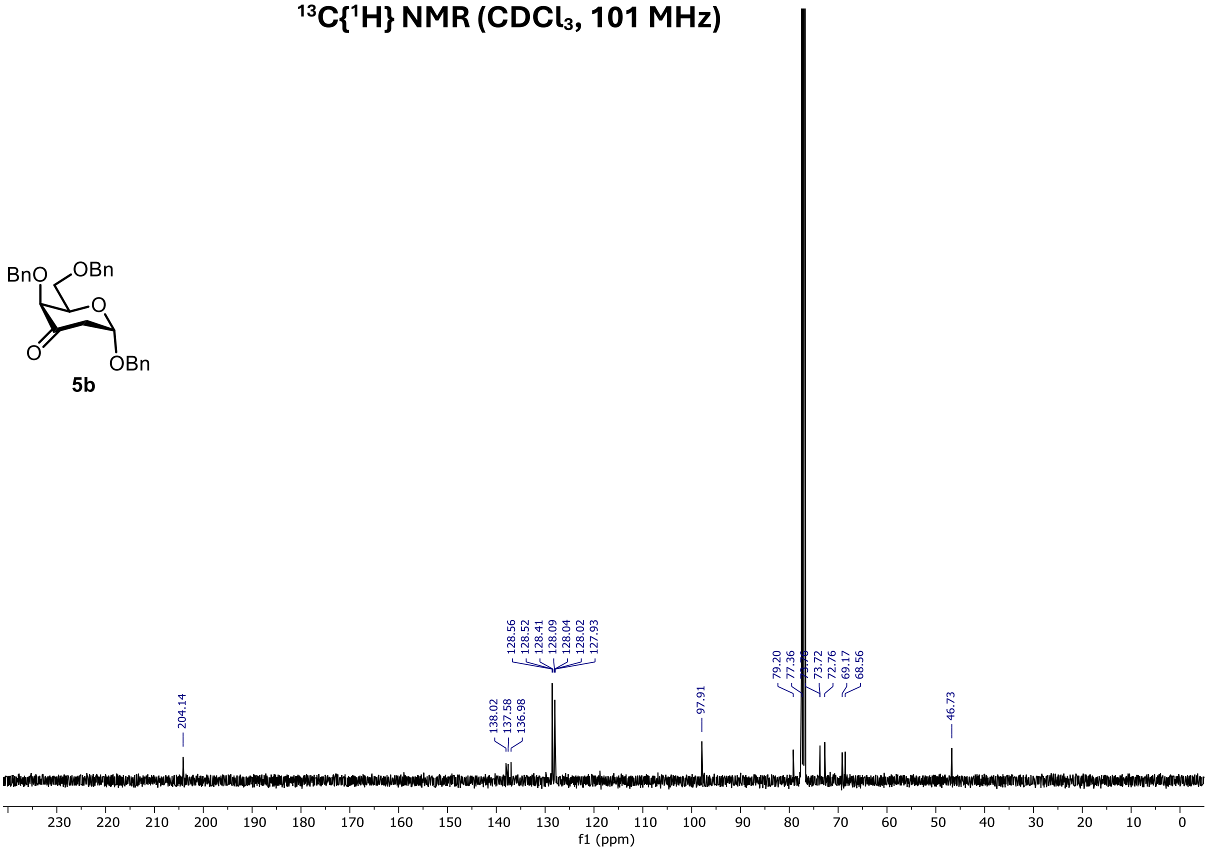

COSY NMR ($CDCl_3$, 400 MHz)

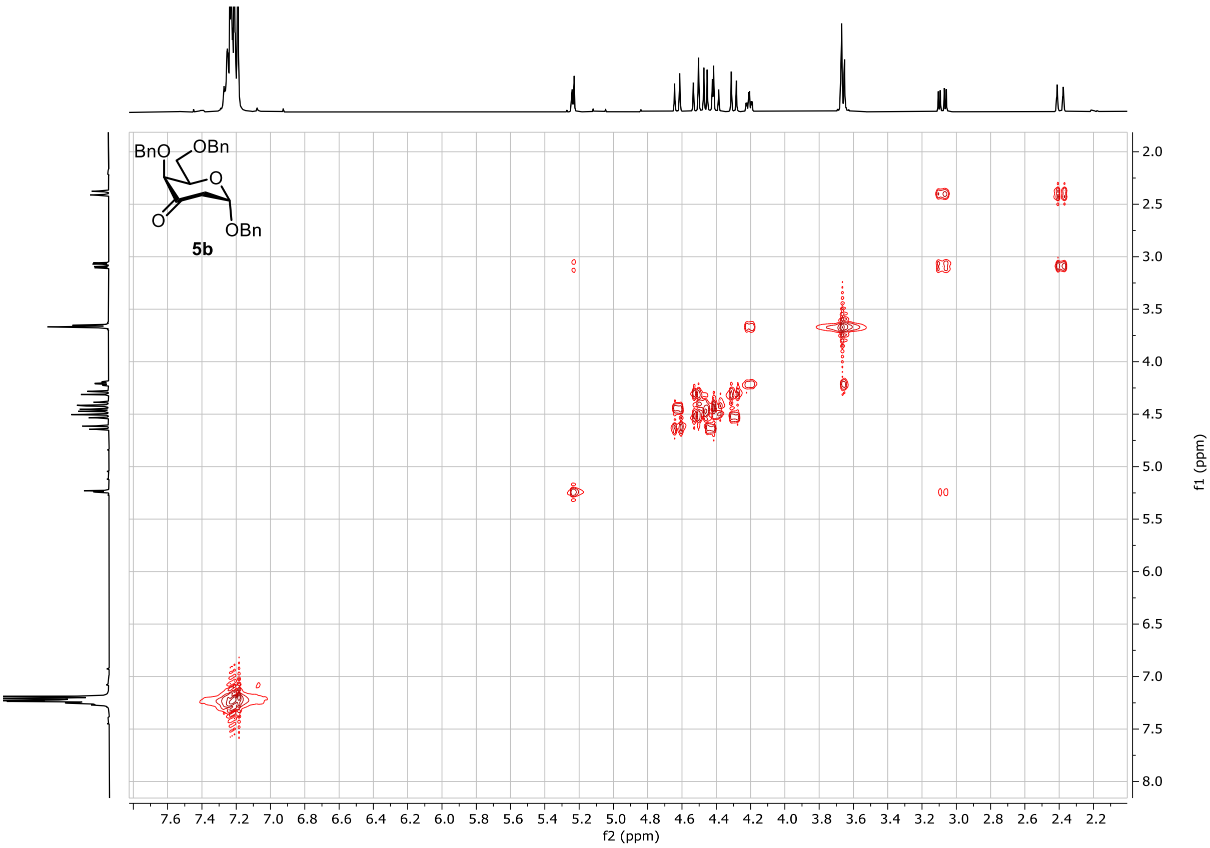

^{1}H NMR ($CDCl_3$, 400 MHz)

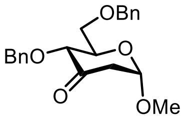

5c

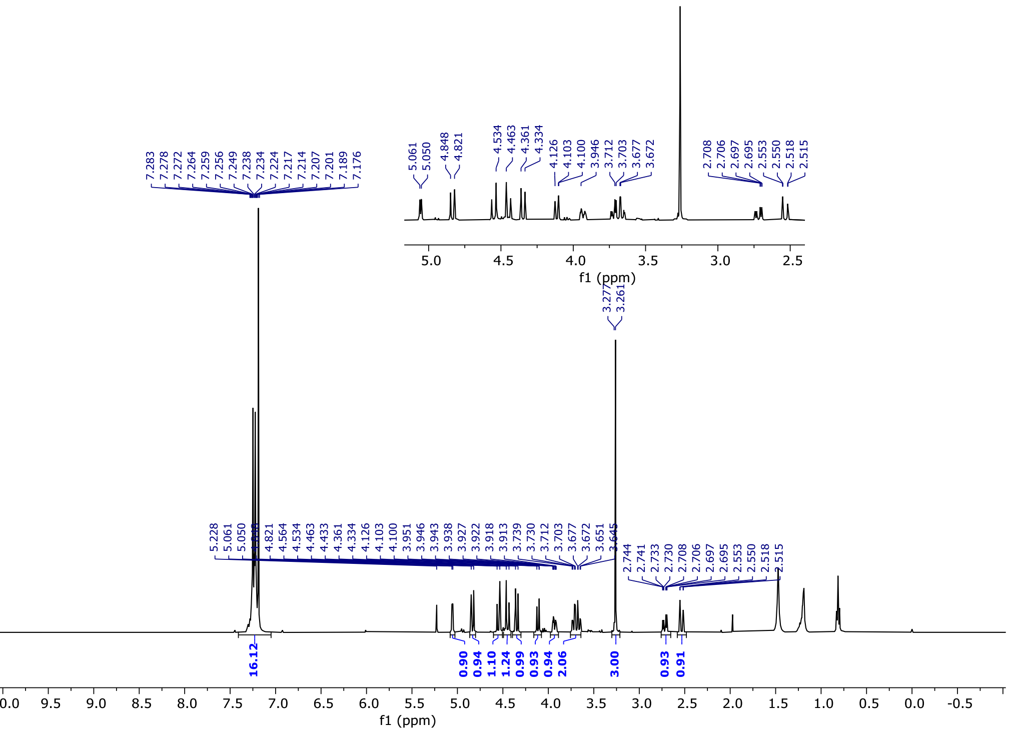

$^{13}C\{^1H\}$ NMR ($CDCl_3$, 101 MHz)

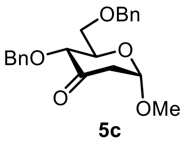

5c

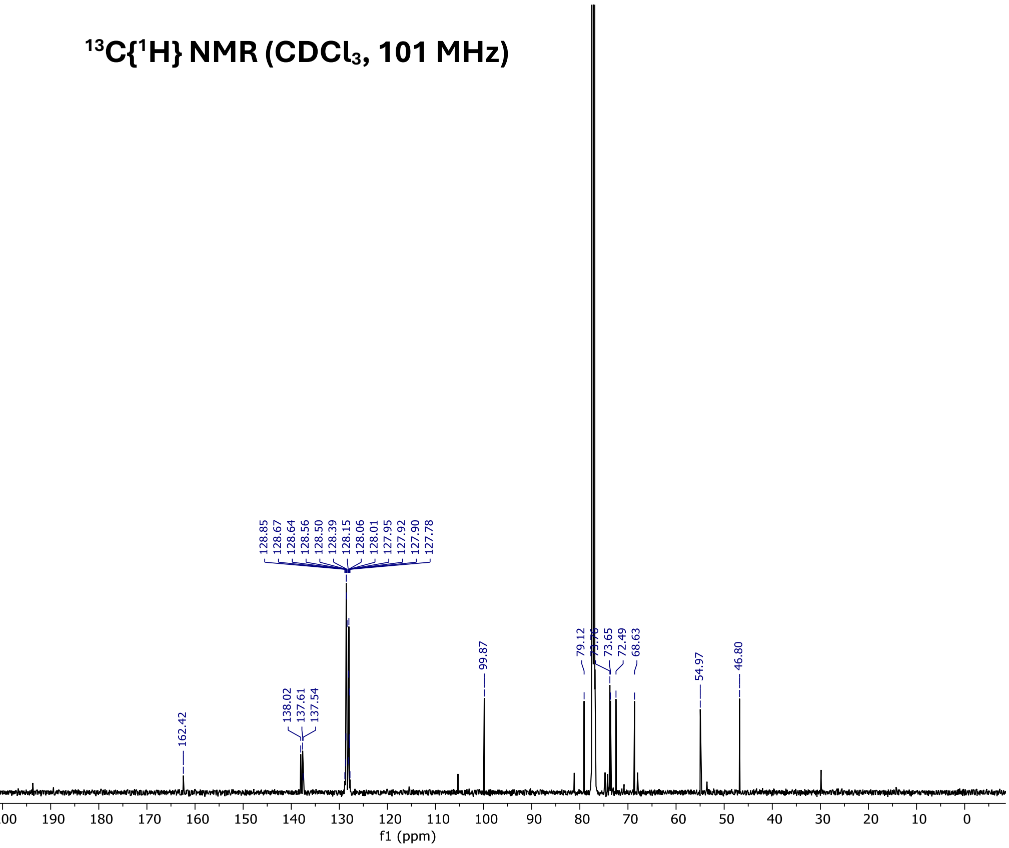

^{1}H NMR (CDCl$_3$, 400 MHz)

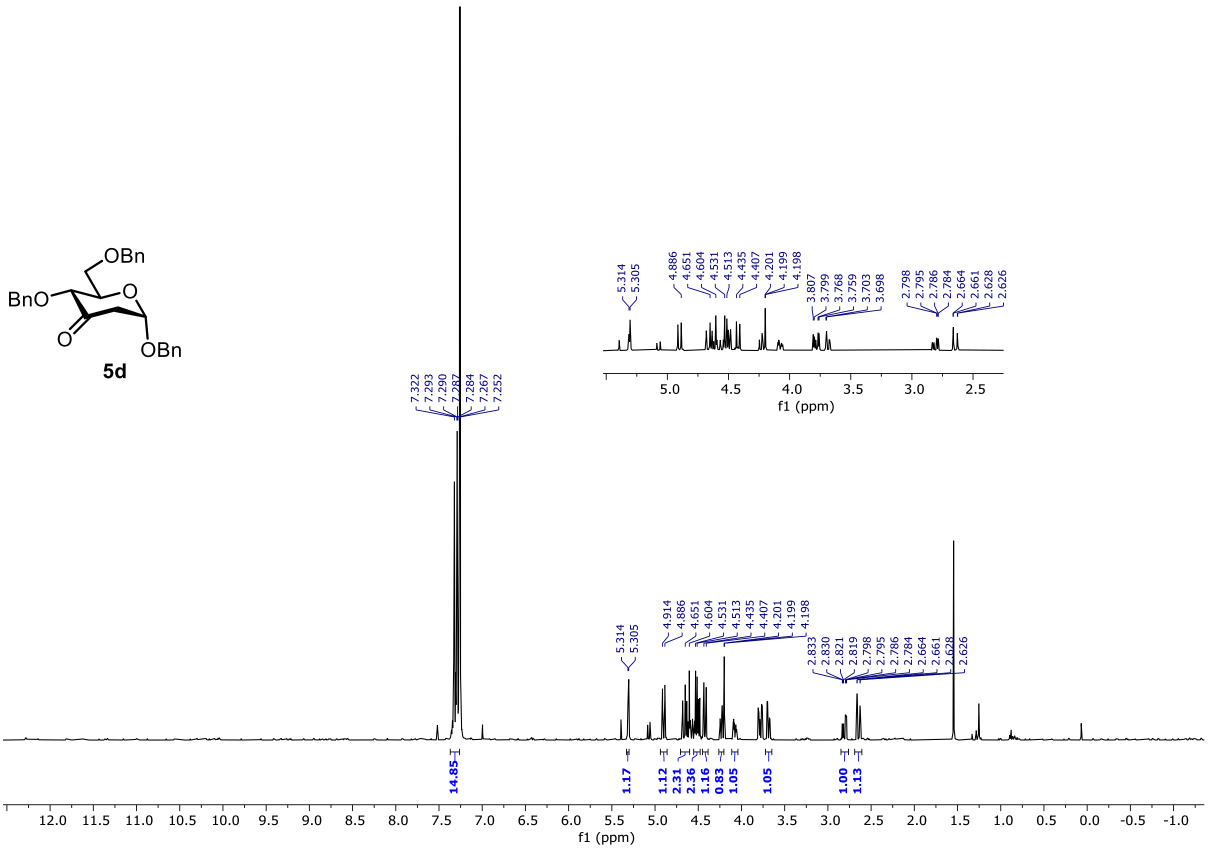

^{13}C{^{1}H} NMR (CDCl$_3$, 101 MHz)

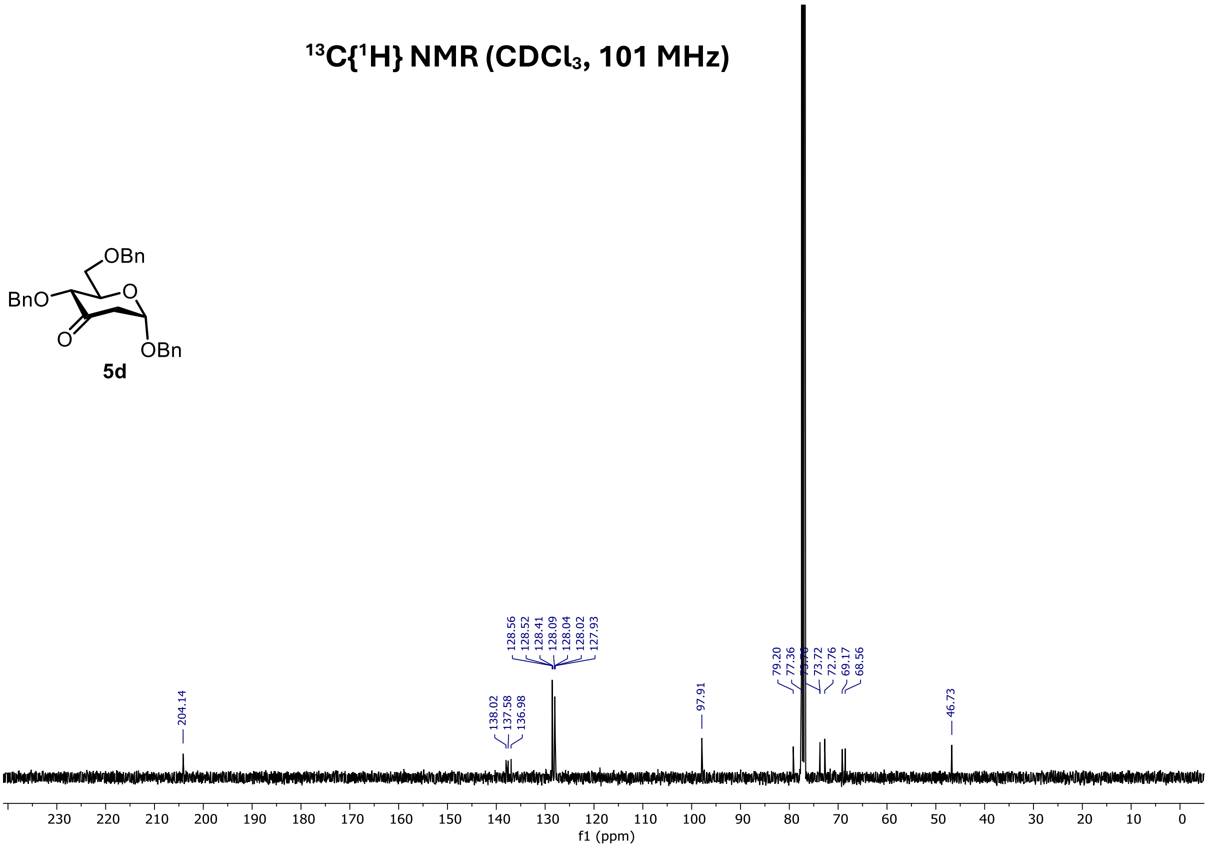

COSY NMR ($CDCl_3$, 400 MHz)

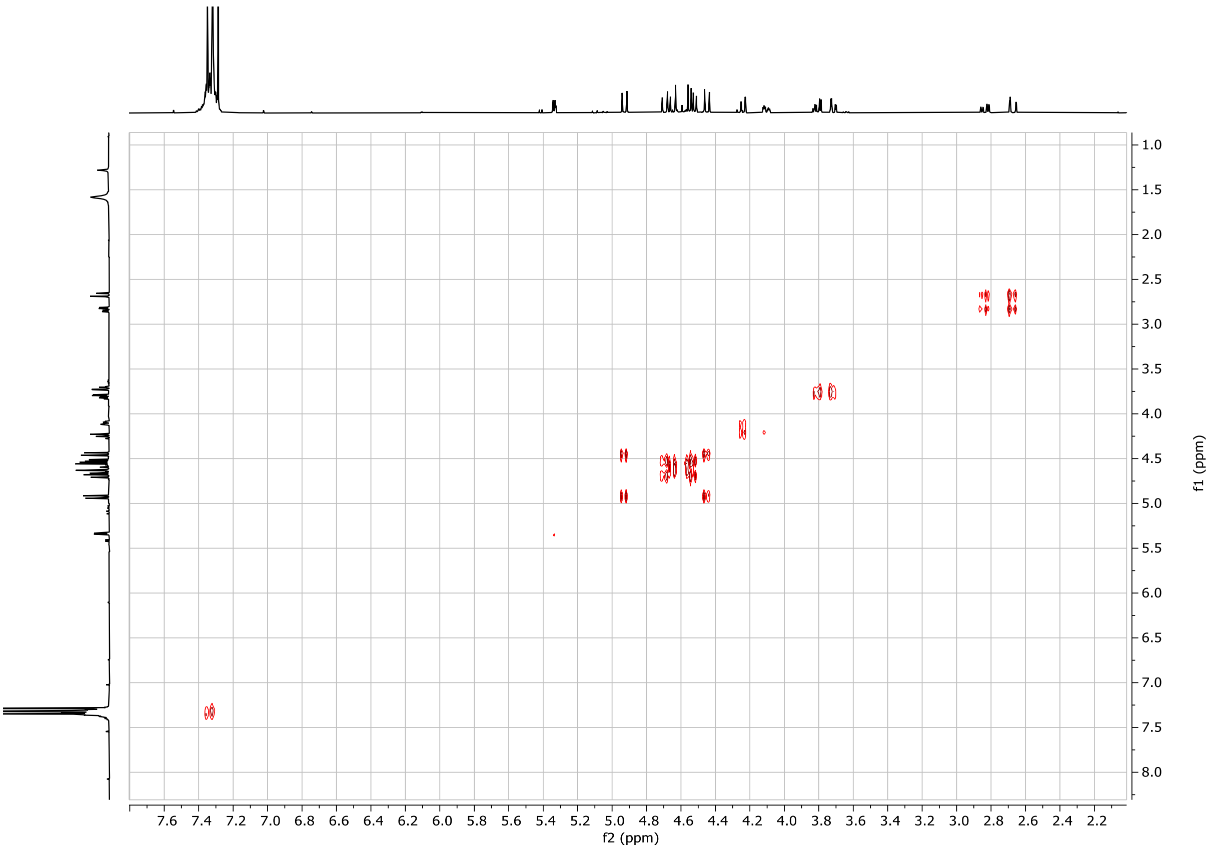

^{1}H NMR ($CDCl_3$, 600 MHz)

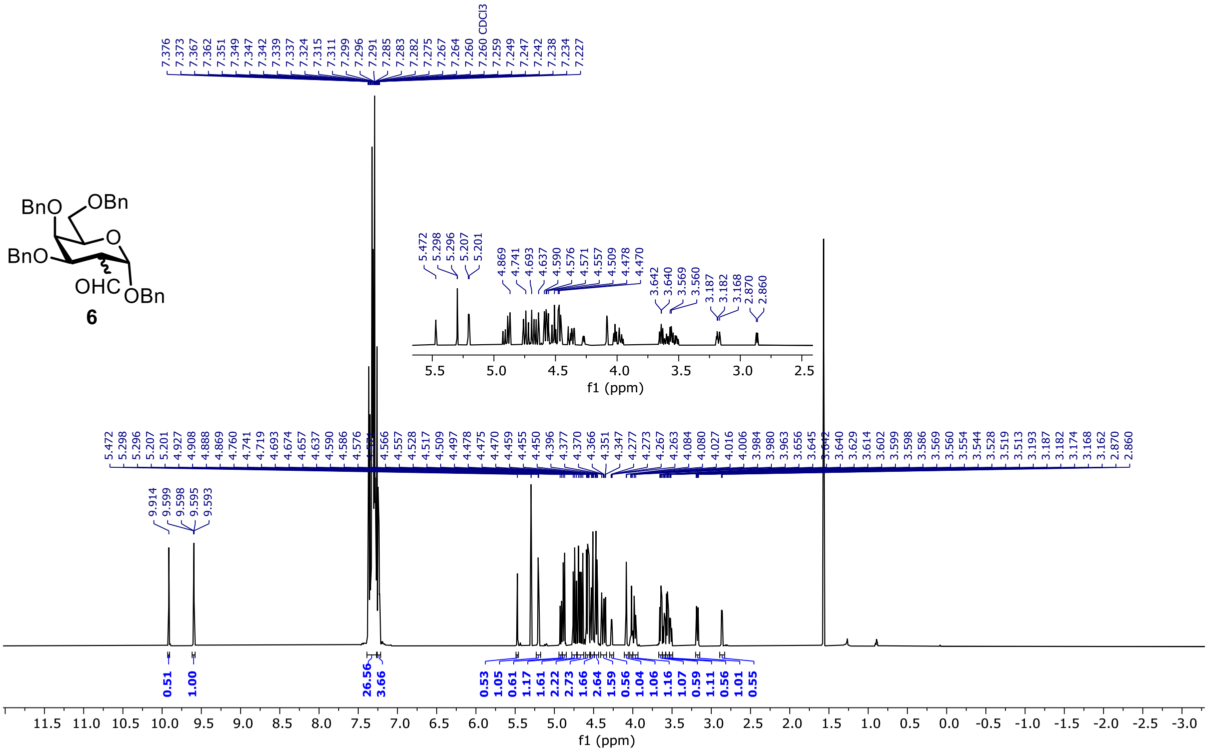

^{13}C{^{1}H} NMR ($CDCl_3$, 151 MHz)

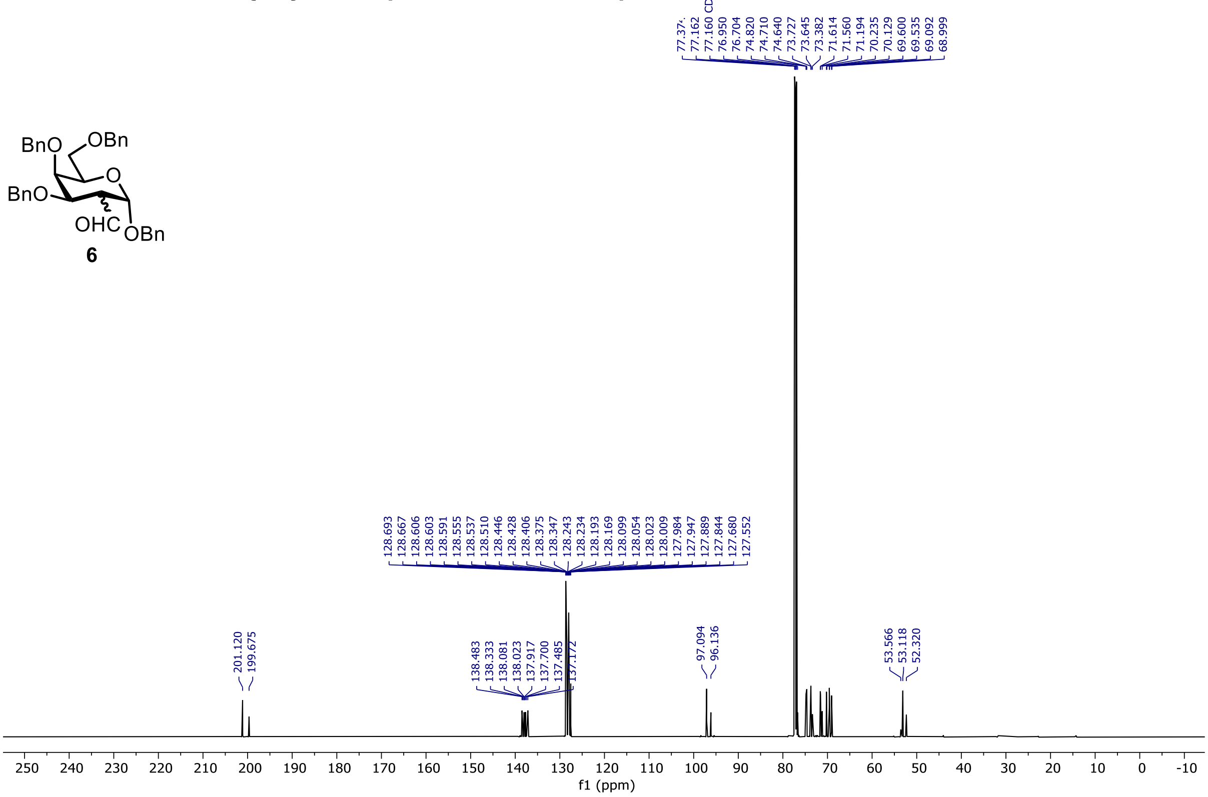

COSY NMR ($CDCl_3$, 600 MHz)

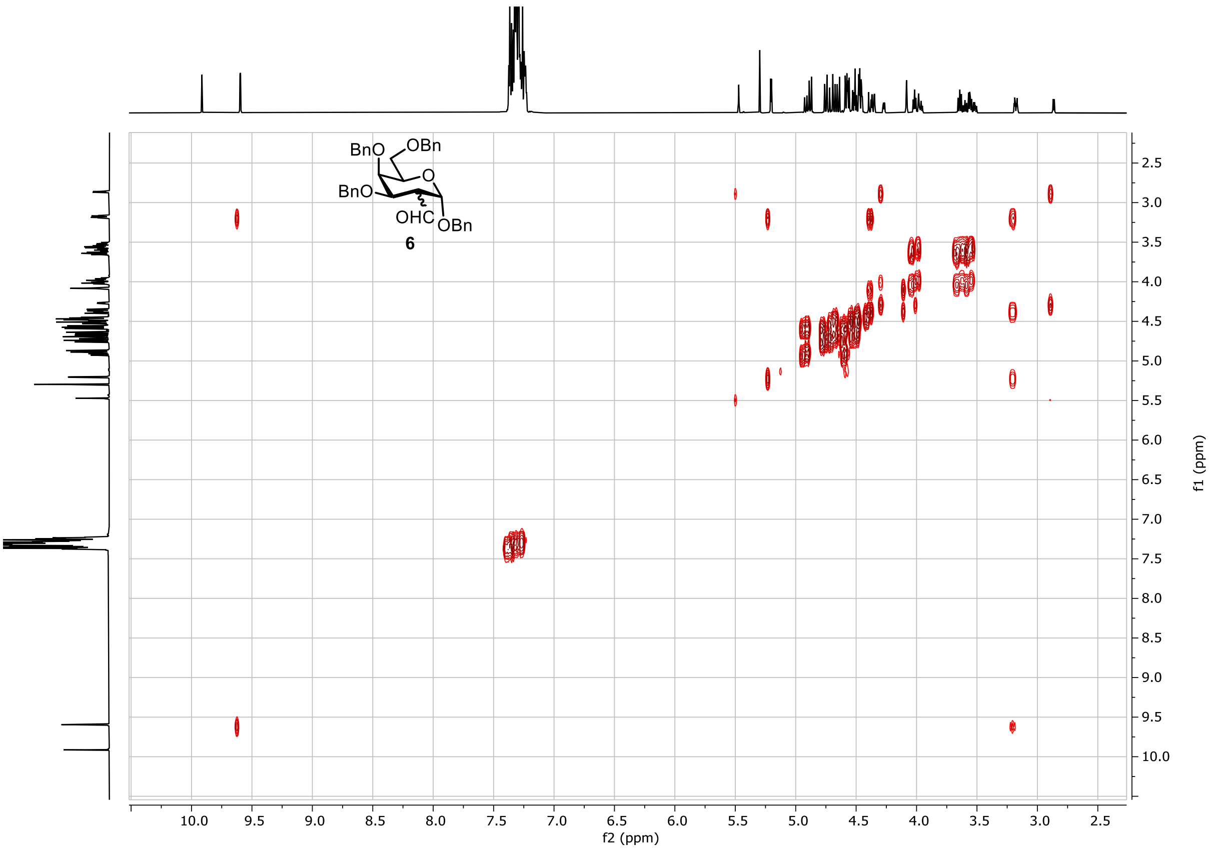

HSQC NMR ($CDCl_3$, 600 MHz)

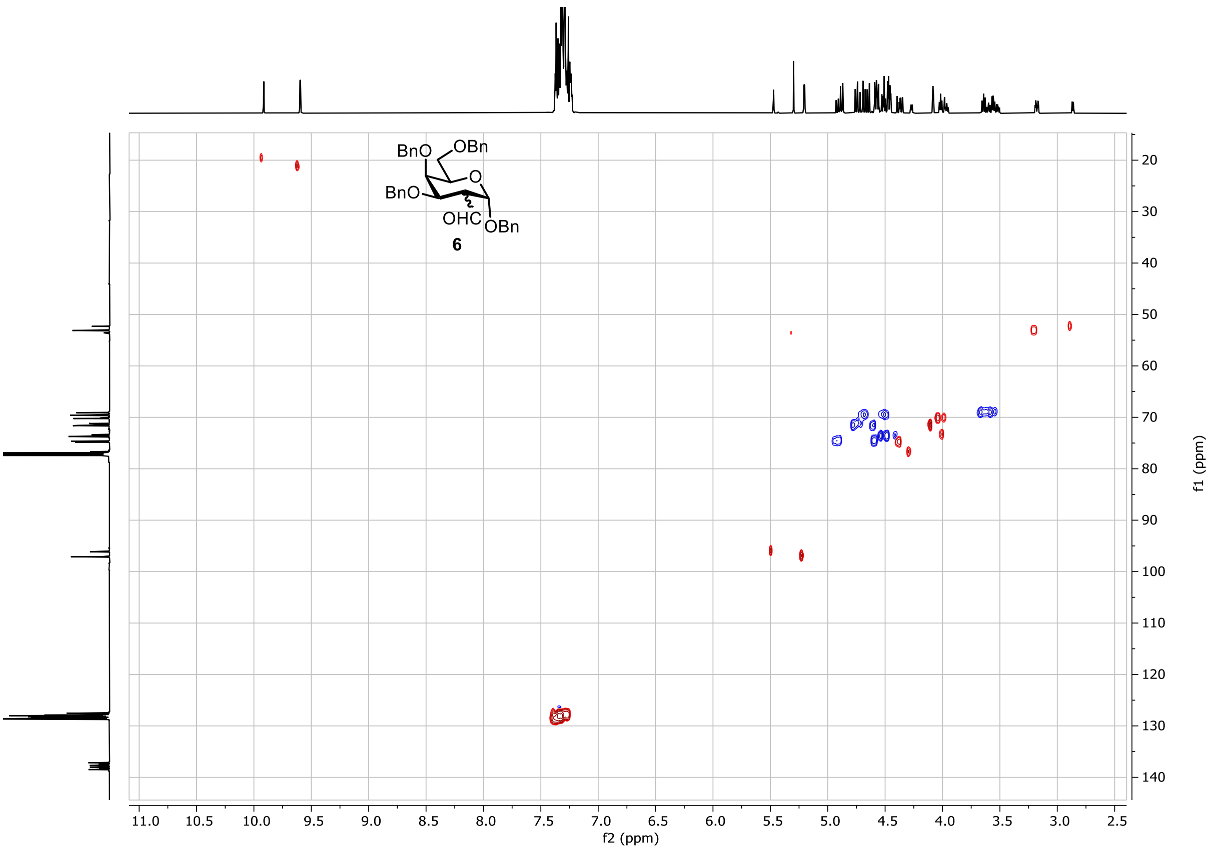

^{1}H NMR ($CDCl_3$, 600 MHz)

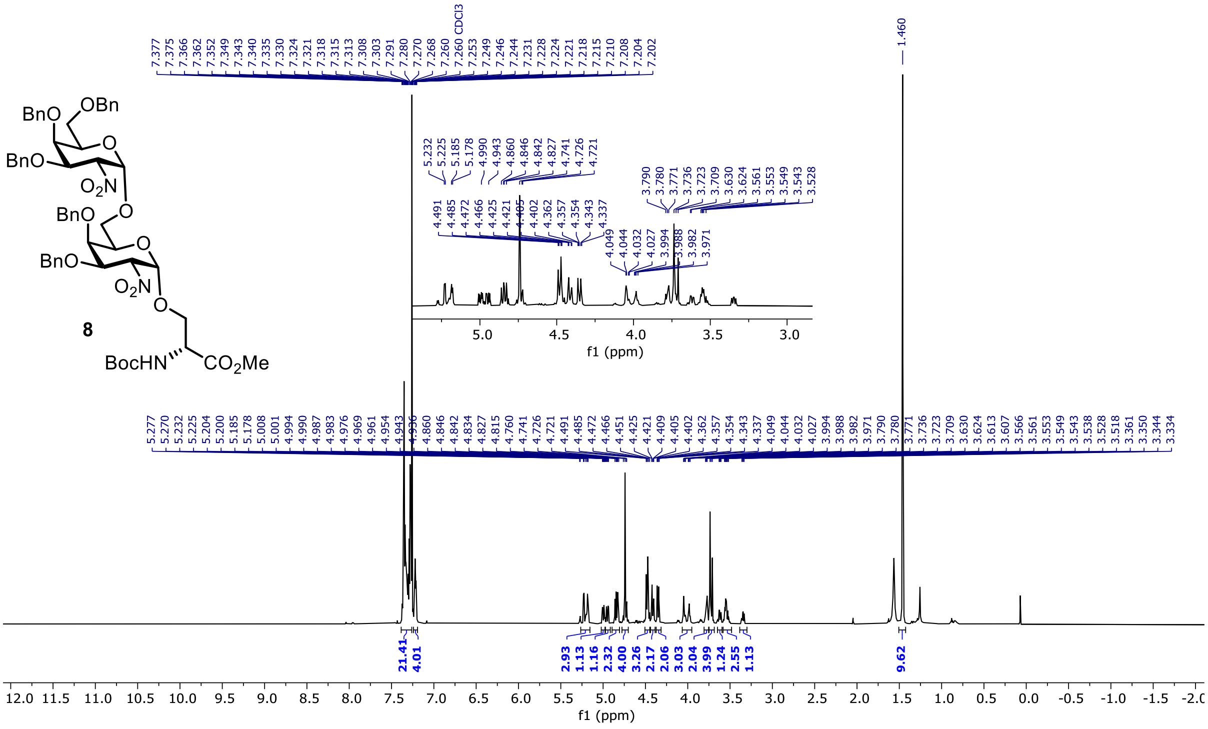

^{13}C{^{1}H} NMR ($CDCl_3$, 151 MHz)

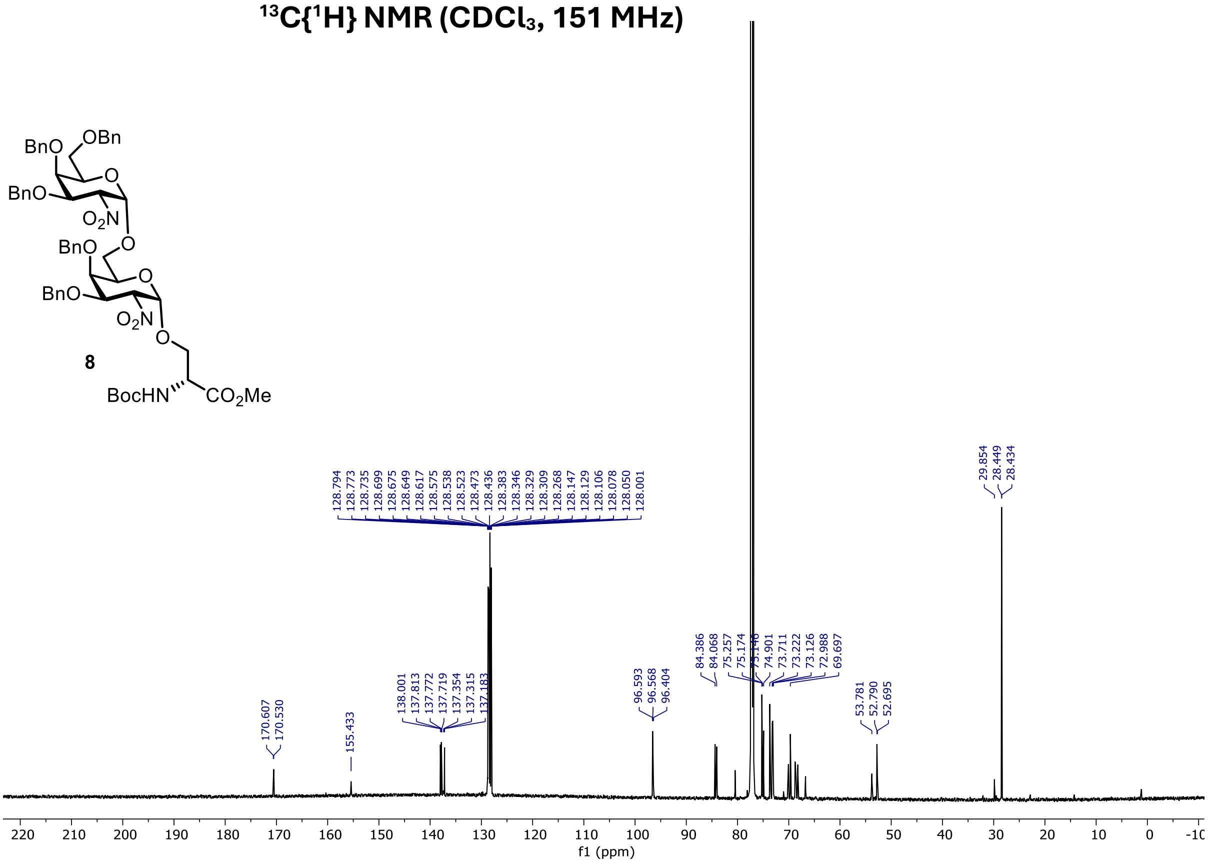

COSY NMR ($CDCl_3$, 600 MHz)

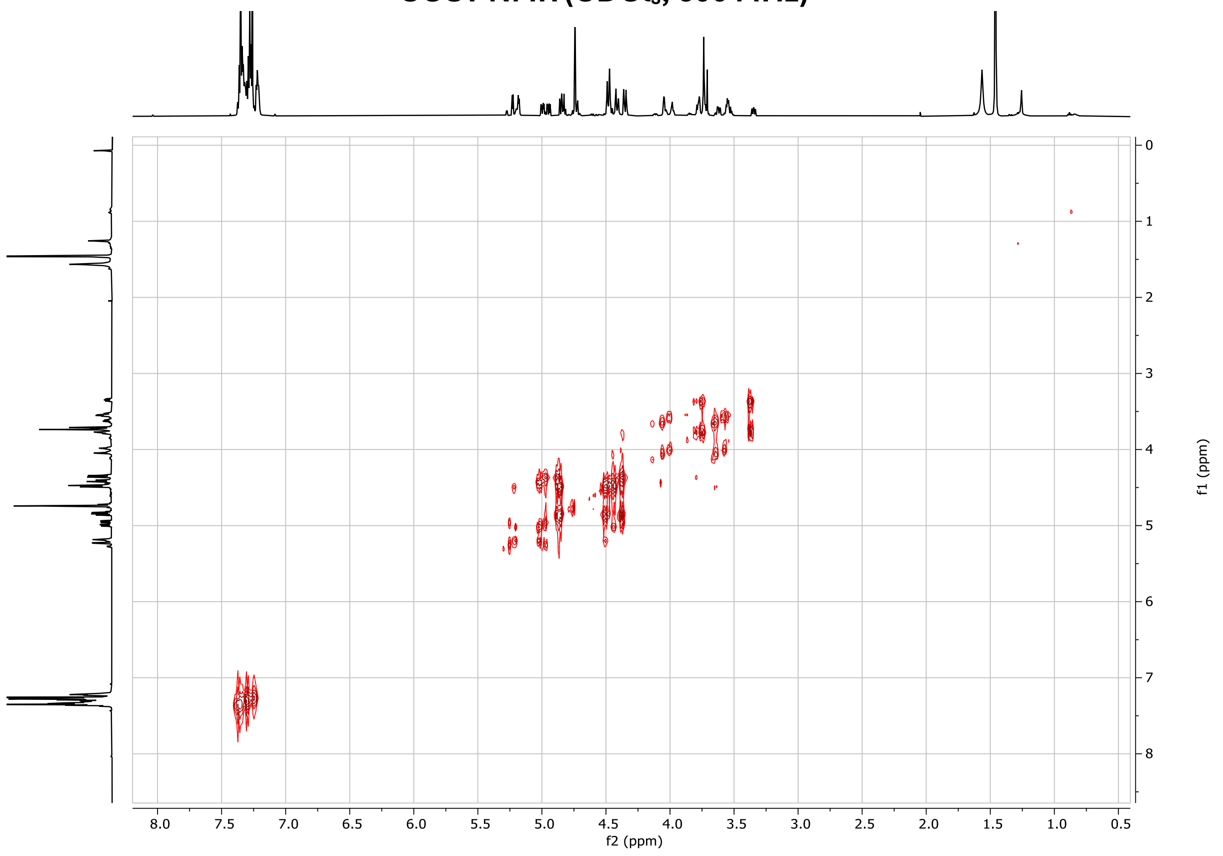

HSQC NMR ($CDCl_3$, 600 MHz)

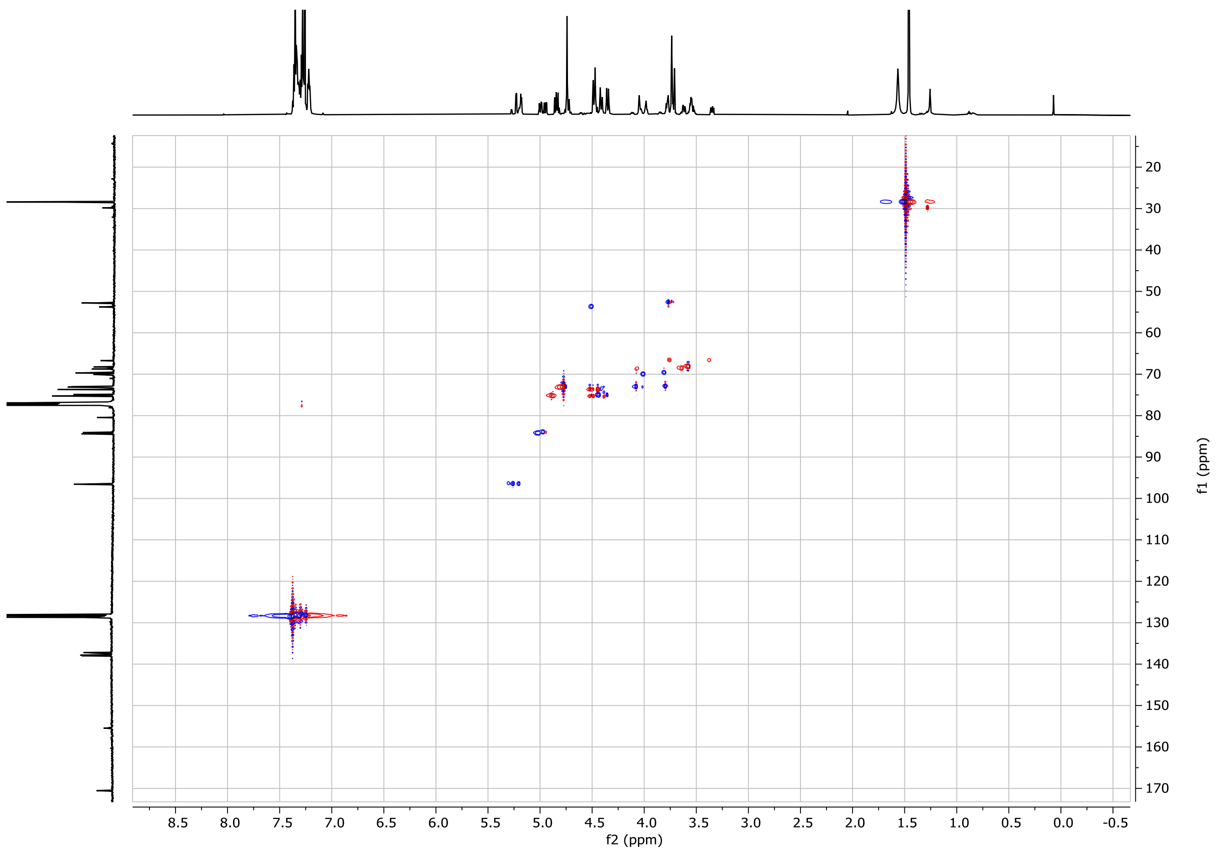

^{1}H NMR (CDCl$_3$, 600 MHz)

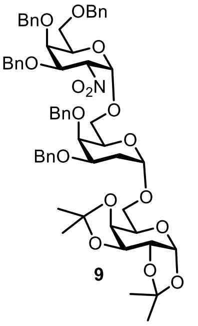

9

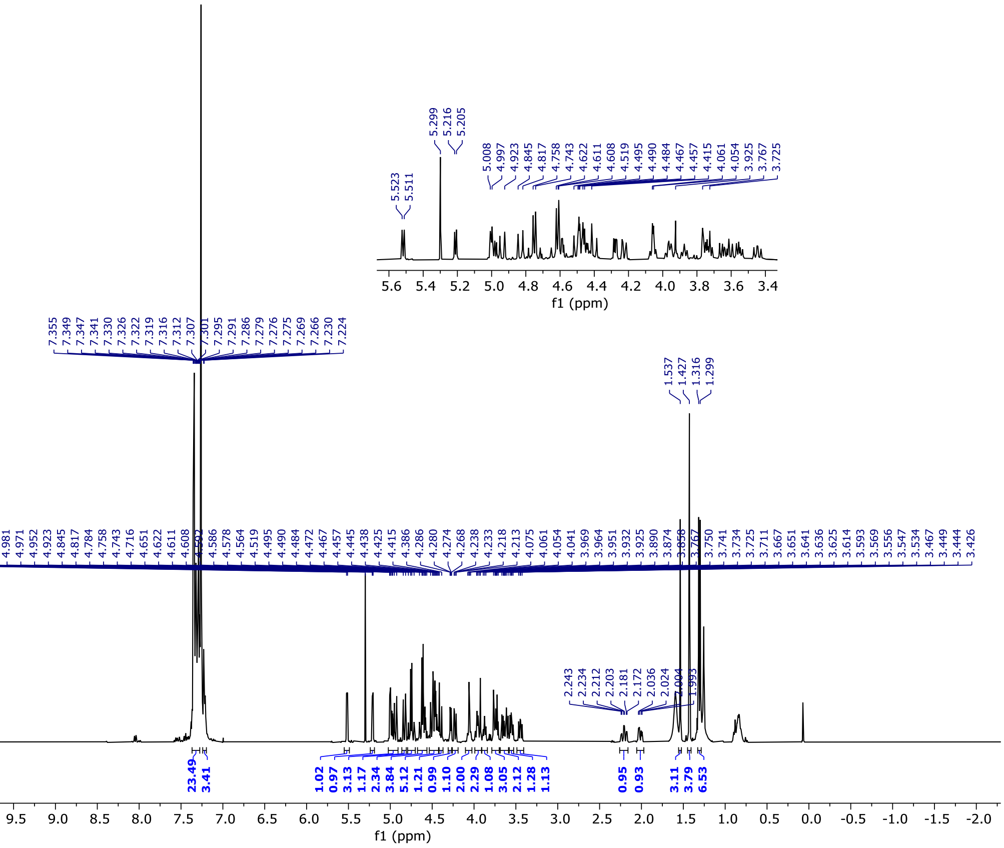

^{13}C{^{1}H} NMR (CDCl$_3$, 151 MHz)

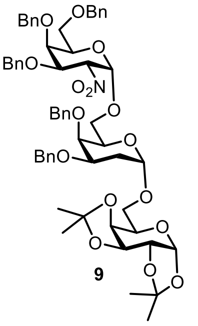

9

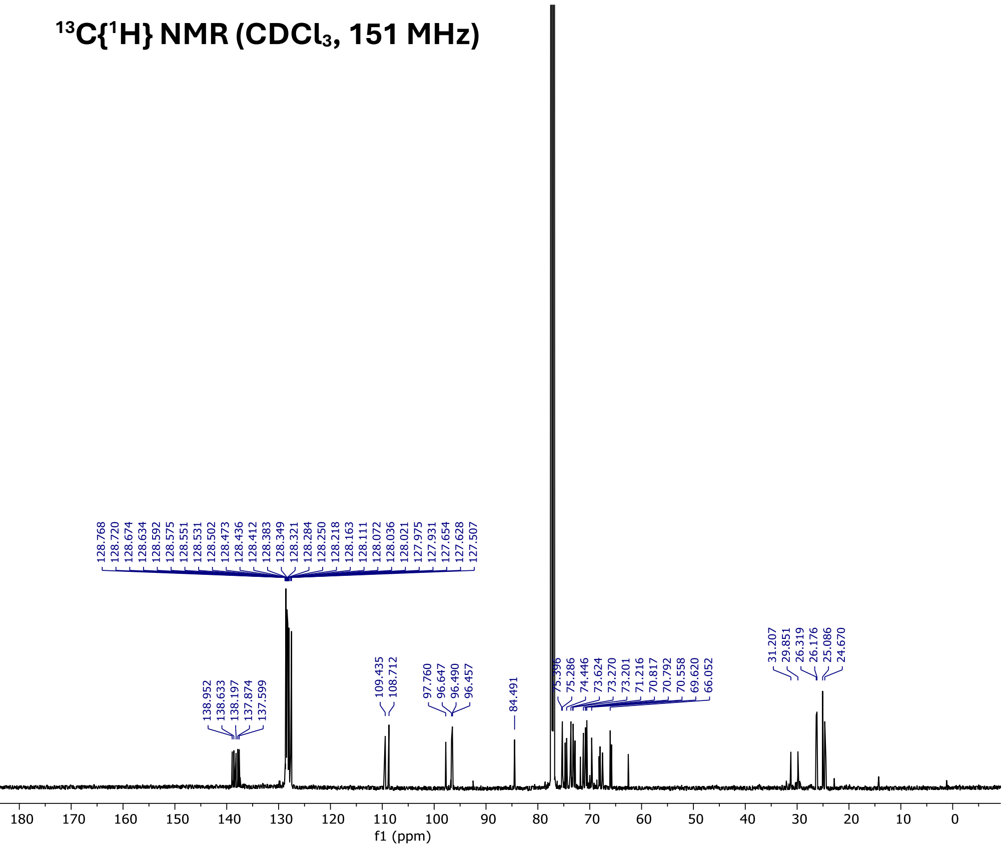

COSY NMR ($CDCl_3$, 600 MHz)

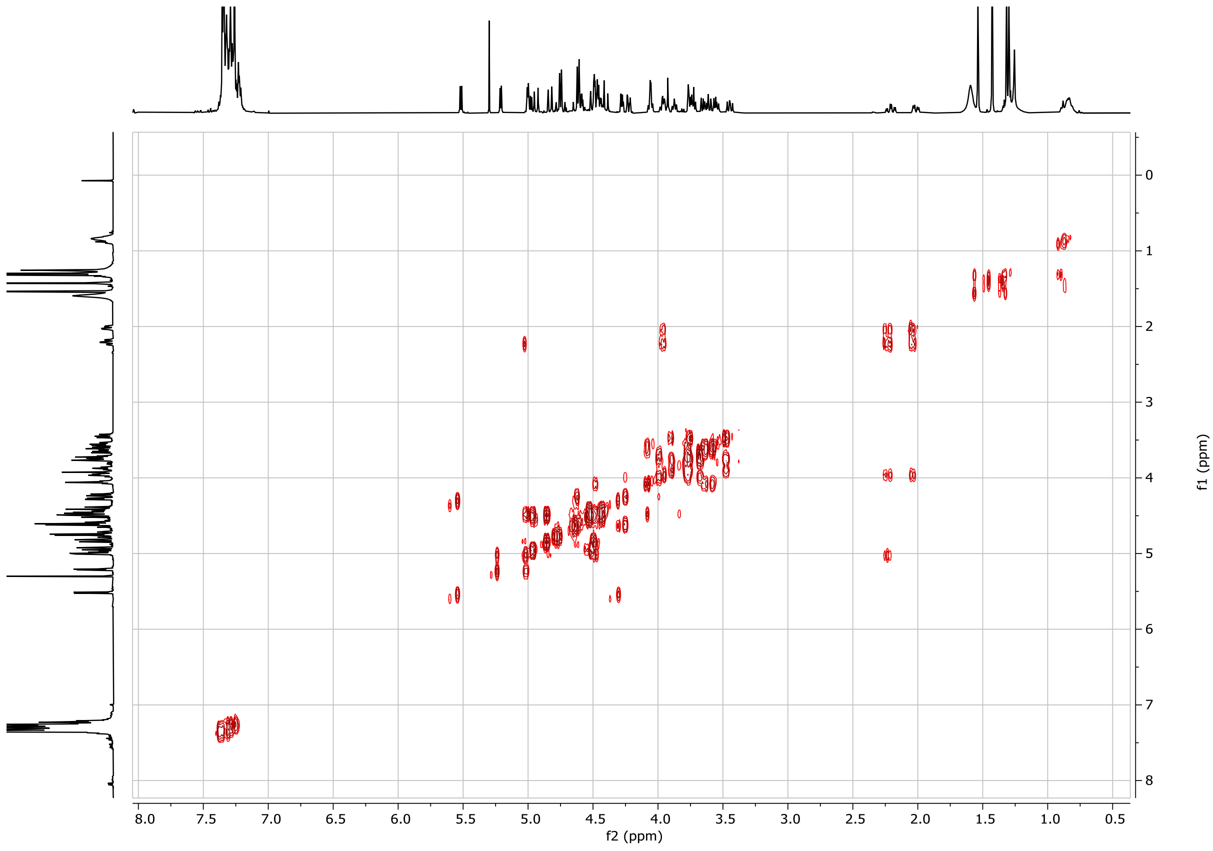

HSQC NMR ($CDCl_3$, 600 MHz)

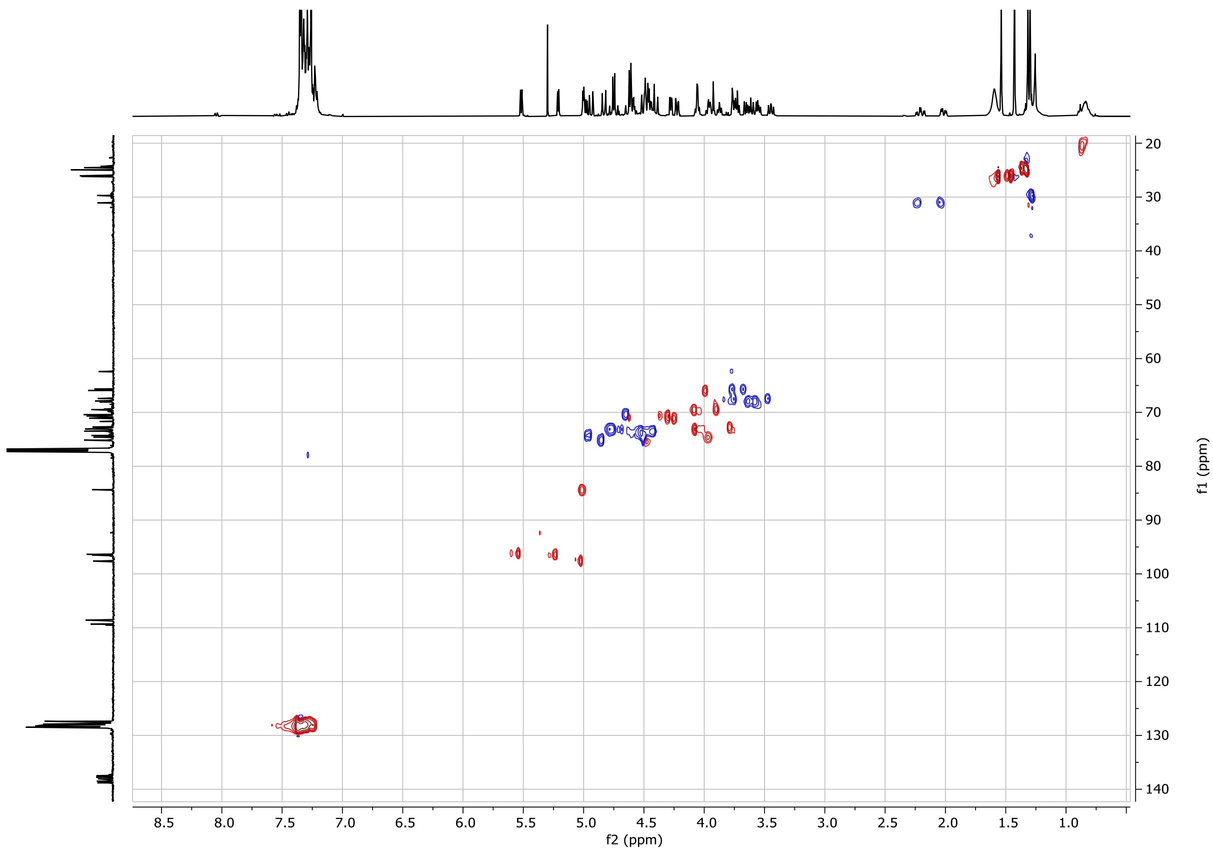

Supplement: Supplementary file 1 [file jo5c00172_si_001.pdf]
